# Molecular determinants for brain targeting by peptides: a meta-analysis approach with experimental validation

**DOI:** 10.1186/s12987-024-00545-5

**Published:** 2024-05-27

**Authors:** Marco Cavaco, Patrícia Fraga, Javier Valle, Ruben D. M. Silva, Lurdes Gano, João D. G. Correia, David Andreu, Miguel A. R. B. Castanho, Vera Neves

**Affiliations:** 1 Instituto de Medicina Molecular João Lobo Antunes, Faculdade de Medicina, Universidade de Lisboa, Av. Prof. Egas Moniz, 1649–028 Lisbon, Portugal; 2 Proteomics and Protein Chemistry Unit, Department of Medicine and Life Sciences, Pompeu Fabra University, Dr. Aiguader 88, Barcelona Biomedical Research Park, 08003 Barcelona, Spain; 3 Centro de Ciências E Tecnologias Nucleares, Instituto Superior Técnico, Universidade de Lisboa, CTN, Estrada Nacional 10 (Km 139,7), 2695–066 Bobadela LRS, Portugal; 4 Departamento de Engenharia E Ciências Nucleares, Instituto Superior Técnico, Universidade de Lisboa, CTN, Estrada Nacional 10 (Km 139,7), 2695–066 Bobadela LRS, Portugal

**Keywords:** Blood–brain barrier (BBB), Blood–brain barrier peptide shuttles (BBBpS), Brain delivery, Cell-penetrating peptides (CPPs), Neurological disorders

## Abstract

Blood–brain barrier (BBB) peptide-shuttles (BBBpS) are able to translocate the BBB and reach the brain. Despite the importance of brain targeting in pharmacology, BBBpS are poorly characterized. Currently, their development relies on the empiric assumption that cell-penetrating peptides (CPPs), with proven ability to traverse lipid membranes, will likewise behave as a BBBpS. The relationship between CPPs/BBBpS remains elusive and, to the best of our knowledge, has not hitherto been subject to thorough experimental scrutiny. In this work, we have identified/quantified the main physicochemical properties of BBBpS and then searched for CPPs with these properties, hence potential BBBpS. The specific features found for BBBpS are: (i) small size, (ii) none or few aromatic residues, (iii) hydrophobic, and (iv) slight cationic nature. Then, we selected the 10 scoring best in an ordinary least squares analysis, and tested them in vitro and in vivo. Overall, we identified the molecular determinants for brain targeting by peptides, devised a methodology that can be used to assist in the design of peptides with potential brain penetration from amino acid residue sequences, and found four new BBBpS within the CPP library.

**Graphical Abstract:**

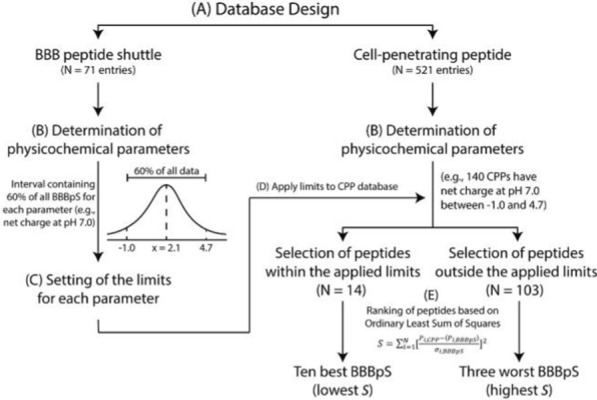

**Supplementary Information:**

The online version contains supplementary material available at 10.1186/s12987-024-00545-5.

## Introduction

Interest in peptide-based biotechnological products is increasing significantly thanks to developments improving their function and/or in vivo stability [1, 2]. Peptides are now routinely used in therapeutic/diagnostic protocols, and are currently gaining a foothold in drug-delivery strategies for their ability to ferry payloads, such as proteins, nucleic acids, small drugs, or nanoparticles into cells and across biological barrier, such as the blood–brain barrier (BBB) [3–5].

A case in point is cell-penetrating peptides (CPPs), which are relatively short peptides that internalize into cells without membrane damage [6]. They have been successfully applied as intracellular carriers for, among others, proteins, nucleic acids, pharmaceuticals (e.g., small molecule drugs and biologics), and nanoparticles. Most CPPs are hydrophobic and positively charged at physiological pH [7, 8]. While their detailed internalization mechanisms remain unclear, even controversial, there is consensus that CPP physicochemical properties and payload, as well as cell type and uptake conditions, have all a bearing on the mode of action [5].

Some CPPs can traverse biological barriers, mostly by receptor-mediated transcytosis (RMT), adsorptive-mediated transcytosis (AMT), or direct diffusion. Among them, those able to traverse the blood–brain barrier (BBB) and access the brain, appropriately named BBB peptide shuttles (BBBpS) [4], are particularly relevant in neuropharmacology. The first BBBpS described was the HIV trans-activator of transduction (TAT) peptide [9]. Subsequently, other peptides, such as SynB, penetratin, Angiopep-2, dNP2, TP10, MiniAp-4, and PepH3 have been investigated and have shown good translocation properties [5, 10–13].

**Fig. 1:**
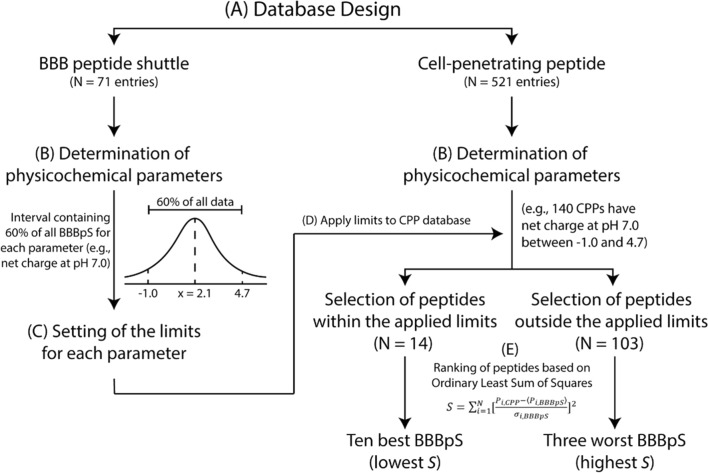
Multi-step strategy in BBBpS selection. The process consisted of five different steps: **A** database design using “peptides”, and/or “cell-penetrating peptides”, “CPP”, “internalization”, “membrane”, “uptake”, and/or “penetration” as keywords for CPP database search engine; and “peptides”, and/or “blood brain barrier”, “BBB”, “BBB peptide shuttle”, “trans-BBB peptide”, “translocation”, “brain”, “endothelial”, and/or “uptake” for BBBpS database search engine as keywords. The search included 33 selected journals and was supplemented by inspection of review papers reporting relevant peptides potentially missed in the engine search; **B** determination of peptide physicochemical parameters; **C** definition of a 60% interval for each parameter; **D** application of defined parameter limits to the CPP database; **E** ordinary least squares ranking to select the ten best and three worst BBBpS (lowest S and highest S, respectively)

Two frequent questions among drug developers are: “Why not all CPPs traverse cellular barriers?” and “What turns a CPP into a BBBpS?”. To shed some light on the issue, we have applied a multi-step methodology to identify molecular hallmarks of BBBpS, followed by a search for CPPs embodying those features (Fig. 1). To this end, we have first created a database with—to our best knowledge—most known BBBpS (71 entries) and, from the amino acid residues sequence of those peptides, defined nine relevant physicochemical parameters and established the central boundaries (i.e., comprising 60% of data) to accommodate the majority of peptides without being affected by outliers for each property. While the value of 60% is defined based on common sense, it results from the dimension of the population of known BBBpS and intrinsic variability of the relevant parameters. The value is a balance between being inclusive (i.e., identifying a pool of potential BBBpS among CPP) and being selective (i.e., only the best matches are included in the pool). In fact, a systematic screen of different values (40–80%) confirms that i) higher values in this range leads to pools composed of so many CPP that their experimental screening is not possible in practice; and ii) lower values in this range lead to too small pools, prone to the exclusion of BBBpS among CPP. Overall, 60% value was set based on the fact that it leads to the pool of CPP showing a dimension of statistical significance and practical enforceable (e.g., experimental tests, and importantly the in vivo biodistribution). Using a second database with most known CPPs (521 entries), we have identified CPPs with physicochemical properties matching the BBBpS hallmarks and applying an ordinary least squares (OLS) method, ranked CPPs for potential BBBpS activity and selected the ten best BBBpS candidates. The choice of an OLS method is based on its simplicity, efficiency, interpretability and flexibility [14, 15]. As one of the most straightforward tools for conducting regression analysis, we find OLS an ideal statistical tool to rank peptides according to their physicochemical properties.

These ten best BBBpS leads were next produced and tested in an in vitro BBB model to validate experimentally the methodology with nine out ten-peptide showing moderate-to-high translocation. We then selected the four best performers and made in vivo biodistribution studies to assess their brain uptake in a mice model. Results confirmed that all peptides had higher brain accumulation (> 0.5% ID/g tissue) when compared to other known BBBpS [5, 10–13]. Taken together, the in vitro and in vivo data showed good correlation with the predicted ranking, confirming the robustness and reliability of our methodology.

## Materials and methods

### Database design and data collection

CPPs and BBBpS are published over a wide variety of journals. We focused our search on 33 journals providing a broad coverage of peptide science areas, from academic scientific research to health applications (Table S1). To identify papers relevant for the BBBpS and CPP databases, we trained the search engine at each journal website for keywords. Specifically, for the CPP database, the keywords “peptides”, “cell-penetrating peptides”, “CPP”, “internalization”, “membrane”, “uptake”, and/or “penetration” were chosen, while for the BBBpS database the keywords were “peptides”, “blood–brain barrier”, “BBB”, “BBB peptide shuttle”, “trans-BBB peptide”, “translocation”, “brain”, “endothelial”, and/or “uptake”. In addition, we examined ten recent reviews (from 2016 on) to complement our databases [4, 5, 12, 16–22]. The information exported to the database was the peptide name (if available), sequence, main cargoes reported, cellular model; pathologies addressed, and proposed internalization/translocation mechanism.

### Identifying CPPs as potential BBBpS

The selection of potential new BBBpS from known CPPs followed a multi-step strategy (Fig. 1). After compiling both peptide databases, nine physicochemical parameters were determined for each entry using a well-established peptide predictor software from bioSYNTHESIS [23] or information from the literature: (a) molecular weight (g mol^−1^), (b) UV–Vis extinction coefficient (M^−1^.cm^−1^), (c) hydrophobic percentage (%), (d) isoelectric point, (e) net charge (pH 7.0), (f) charge, (g) average hydrophobicity, (h) hydrophobicity (pH 7.0), and (i) hydrophilic residues ratio (%). For each of these properties in the BBBpS database, a 60% inclusion range, i.e., an interval reasonably representative of the property value distribution was defined and applied as cutoffs to identify potential BBBpS in the larger CPP database. Peptides with all parameters within the defined cutoffs were then ranked using an OLS method:

1 $$S = \sum _{{i = 1}}^{N} \left[ {\frac{{P_{{i,CPP}} - \langle P_{{i,BBBpS}} \rangle }}{{\sigma _{{i,BBBpS}} }}} \right]^{2}$$

where, *N* is the number of parameters, *P*_*i,CPP*_ is the value of parameter *i* for a specific CPP, and *P*_*i,BBBpS*_ and *σ*_*iBBBpS*_ are respectively the average value and the standard deviation of parameter *i* in the BBBpS database. In our case, the a-i parameters defined above imply N = 9. Using Eq. 1, we can identify the CPPs that are more alike the BBBpS characteristics, given the static intrinsic variability of BBBpS, as they will be associated to a smaller value of sums of squares (S).

The ten CPPs with lowest *S* values were thus selected to be experimentally tested as BBBpS candidates. Likewise, the three CPPs with the highest S values among those outside the 60% intervals were selected as negative controls (non-BBBpS). For simplicity, the BBBpS candidates are hereafter referred to as BBBpS_X (X = 1, 2, …, 10), and the three non-BBBpS as non-BBBpS_Y (Y = 1, 2, 3). Numbering is assigned alphabetically according to peptide names hence does not involve a best/worst BBBpS candidate ranking. An example of the application of the method to the “net charge at pH 7” parameter is shown in Fig. 1.

### Chemicals and materials

Fmoc-protected amino acids, Fmoc-Rink amide (MBHA) resin, 2-(1H-benzotriazol-1-yl)-1, 1,3,3-tetramethyluronium hexafluorophosphate (HBTU), and *N*-hydroxybenzotriazole (HOBt) were from Iris Biotech (Marktredwitz, Germany). HPLC-grade acetonitrile (ACN), and peptide-synthesis grade *N,N*-dimethylformamide (DMF), dichloromethane (DCM), *N,N*-diisopropylethylamine (DIEA), *N,N*-diisopropylcarbodiimide (DIPCI), trifluoroacetic acid (TFA), and triisopropylsilane (TIS) were from Carlo Erba-SDS (Sabadell, Spain). 3,6-dioxa-1,8-octanedithiol (DODT), 5(6)-carboxyfluorescein (CF), and tetramethylrhodamine isothiocyanate-4 KDa dextran (TRITC-Dx4) were from Sigma-Aldrich (Spain).

Dulbecco’s modified Eagle medium (DMEM), DMEM/Ham’s F-12 (DMEM:F12), DMEM:F12 without phenol-red, trypsin–EDTA, attachment factor protein solution (AF), fetal bovine serum (FBS), and penicillin–streptomycin antibiotic solution (Pen/Strep) were from Gibco/Thermo Fischer (USA). Minimum essential medium Eagle (EMEM), and endothelial cell growth supplement (ECGS) were from Sigma-Aldrich (Spain). CellTiter-Blue^®^ cell viability reagent was from Promega (Spain).

### Peptides

The peptide sequences in Table 1 were assembled in a Prelude synthesizer (Gyros Protein Technologies, USA) running Fmoc (FastMoc) solid-phase peptide synthesis (SPPS) protocols at 0.1 mmol scale on a Fmoc-Rink-amide ChemMatrix resin. Non-labeled versions of the peptides were obtained upon acidolytic (TFA) deprotection and cleavage of the respective peptide-resins, followed by semi-preparative reverse phase HPLC purification and analytical documentation by HPLC and MS as described [24]. Fluoro- and/or radiolabeled versions of each peptide (Table 1) were also made as required, by coupling either 5(6)-carboxyfluorescein (CF) [24] or the ^67^Ga chelating unit NODA-Ga(tBu)_3_ [11, 25] at the N-terminus of the corresponding peptide-resin, following optimized protocols. The CF- and NODA-GA labeled peptides were obtained after TFA treatment of the corresponding peptide-resins, and purified and characterized similarly to the free versions above. For radiolabeling with ^67^Ga^3+^, a fraction of ^67^GaCl_3_ (0.5 Ml, 156 MBq) eluted from a Sep-Pak^®^ Classic Silica cartridge (690 mg, 55–105 µm, Waters^™^) was adjusted to pH 5.5 by 0.5 mL of 0.4 M sodium acetate buffer pH 5.5. An aliquot of this solution (190 μL, 28–32 MBq) was added to the purified ^67^Ga-NODA-GA labeled peptide (10 μL, 0.75 mM), and the mixture was incubated for 30 min at r.t. The radiochemical purity and retention time of the ^67^Ga-peptides were evaluated by a separate analytical RP-HPLC coupled to a γ-detector, as described [11, 25]. All peptides were > 90% pure by HPLC, except BBBpS_8 and CF-non-BBBpS_2 (> 85%).

**Table 1:** Sequence and analytical data for the peptides

Peptide	Code	Amino acid sequence	Theoretical mass (Da)^a^	Experimental mass (Da)^b^	HPLC t_R_ (min)	Purity (%)^c^
A	BBBpS_1	KYKGAIIGNIK-amide	1203.57	1203.49	4.99	96.54
CF-A		CF-KYKGAIIGNIK-amide	1561.89	1560.79	7.05	96.02
^67^ Ga-A		^67^ Ga-KYKGAIIGNIK-amide	N.A	N.A	11.75	> 95
B	BBBpS_2	KYRSGAITIGY-amide	1227.49	1227.43	5.65	97.29
CF-B		CF-KYRSGAITIGY-amide	1585.81	1584.73	7.62	94.63
^67^ Ga-B		^67^ Ga-KYRSGAITIGY-amide	N.A	N.A	11.95	> 95
CPP(II)	BBBpS_3	EEGRLYMRYYSPTTRRYG-amide	2297.66	2297.58	5.60	97.06
CF-CPP(II)		CF-EEGRLYMRYYSPTTRRYG-amide	2655.98	2654.88	7.95	90.79
CTP	BBBpS_4	APWHLSSQYSRT-amide	1431.63	1431.57	5.51	95.66
CF-CTP		CF-APWHLSSQYSRT-amide	1789.95	1788.87	8.45	93.68
HAP-2	BBBpS_5	HIQLSPFSQSWR-amide	1484.75	1484.68	7.14	94.53
CF-HAP-2		CF-HIQLSPFSQSWR-amide	1843.07	1841.98	9.50	93.70
^67^ Ga-HAP-2		^67^ Ga-HIQLSPFSQSWR-amide	N.A	N.A	12.97	> 95
hCR(12–32)	BBBpS_6	YTQDFNKFHTFPQTAIGVGAP-amide	2338.72	2338.61	6.78	97.46
CF-hCR(12–32)		CF-YTQDFNKFHTFPQTAIGVGAP-amide	2697.04	2695.91	8.79	94.18
KLA13	BBBpS_7	LKTLTETLKELTKTLTEL-amide	2074.61	207.49	10.05	95.95
CF-KLA13		CF-LKTLTETLKELTKTLTEL-amide	2432.93	2431.79	13.51	98.50
Peptide b3-1	BBBpS_8	YKEATSTFTNITYRGT-amide	1852.11	1852.03	5.87	86.69
CF-Peptide b3-1		CF-YKEATSTFTNITYRGT-amide	2210.43	2209.33	8.63	95.32
Peptide third	BBBpS_9	NRPDSAQFWLHH-amide	1506.71	1506.65	6.13	93.08
CF-Peptide third		CF-NRPDSAQFWLHH-amide	1865.03	1863.95	8.01	96.68
^67^ Ga-Peptide third		^67^ Ga-NRPDSAQFWLHH-amide	N.A	N.A	12.25	> 95
SPA	BBBpS_10	RPKPQQFFGLM-amide	1347.71	1347.65	7.58	95.23
CF-SPA		CF-RPKPQQFFGLM-amide	1706.03	1704.95	9.25	95.05
P1746c27	non-BBBpS_1	KKKKQPPKPKKPKTQEKKKKQPPKPKR-amide	3262.23	3262.08	2.77	98.57
CF-P1746c27		CF-KKKKQPPKPKKPKTQEKKKKQPPKPKR-amide	3620.55	3619.38	4.23	98.04
TI	non-BBBpS _2	KWCFRVCYRGICYRRCR-amide	2267.85	2267.81	6.81	94.63
CF-TI		CF-KWCFRVCYRGICYRRCR-amide	2626.17	2625.11	8.11	87.61
^67^ Ga-TI		^67^ Ga-KWCFRVCYRGICYRRCR-amide	N.A	N.A	12.99	99.0
YDEGE	non-BBBpS _3	YDEEGGGE-amide	853.84	853.80	2.60	95.71
CF-YDEGE		CF-YDEEGGGE-amide	1212.16	1211.10	7.89	98.39

^a^Calculated using GPMAW version 8.10

^b^From the mass spectrum

^c^Estimated by HPLC peak integration of UV chromatogram (free and CF-labelled versions) or radio-chromatogram (^67^ Ga-labelled versions)

CF, 5(6)-carboxyfluorescein

^67^ Ga, ^67^ Ga-NODA-GA Gallium 67 chelate

N.A. not applicable

### Cell culture

Human cerebral microvasculature endothelial cells (HEBC-5i, ATCC^®^ CRL-3245^™^), human fibroblast (Hs68, ATCC^®^ CRL-1635^™^), human epithelial cells (HeLa, ATCC^®^ CCL-2^™^), human breast cancer cells (MDA-MB-231, ATCC^®^ HTB-26^™^), and human embryonic kidney cells (HEK-293, ATCC^®^ CRL-1573^™^) were purchased from American Type Culture Collection (Manassas, VA). Hs68, HeLa, and MDA-MB-231 cells were cultured as a monolayer in DMEM supplemented with 10% FBS, and 1% pen/strep, according to manufacturer’s instructions. HEK-293 cells were cultured as a monolayer in EMEM supplemented with 10% FBS, and 1% pen/strep, according to manufacturer’s instructions. HBEC-5i cells were cultured as a monolayer on AF-coated T-flasks in DMEM:F12 supplemented with 10% FBS, 1% pen/strep, and 40.0 µg/mL ECGS, according to manufacturer’s instructions. All cells were grown in a humidified atmosphere of 5% CO_2_ at 37 ℃ (MCO-18AIC (UV), Sanyo, Japan) with the medium changed every other day.

### Translocation across a human endothelial cell line

The translocation capacity of all CF-peptides was evaluated using an in vitro HBEC-5i cell model, as previously described [24, 26]. Briefly, HBEC-5i cells were carefully harvested with trypsin–EDTA and seeded at 8.000 cells/well in AF pre-coated tissue culture inserts (transparent polyester (PET) membrane with 1.0 µm pores) for 24-well plates (BD Falcon, USA). Throughout 8 days, medium was changed every other day. On the day of the experiment, cells were washed twice with 1X PBS (137.0 mM NaCl, 2.7 mM KCl, 10.0 mM Na_2_HPO_4_, and 1.8 mM KH_2_PO_4_), and once with DMEM:F12 without phenol red. Then, CF-peptides (5.0 µM, in DMEM:F12 without phenol red) were added to the apical side of the in vitro BBB model and incubated for 24 h.

Finally, samples from the apical and basolateral side were collected and fluorescence intensity analyzed using a Varioskan^™^ LUX multimode microplate reader (Thermo Fisher, Spain). The percentage (%) of translocation was calculated using the following equation:

2 $$Translocation \left(\%\right)=\left(\frac{{F}_{i}-{F}_{cells}}{{F}_{peptide}-{F}_{Medium}}\right)\times 100$$

where* F*_*i*_*, F*_*cells*_*, F*_*peptide*_ and *F*_*Medium*_ denote respectively the fluorescence intensity recovered, that of untreated cells, that of total peptide initially added to the transwell apical side, and that of the medium. Retention (%) corresponds to the remaining fluorescence [100—(F_apical_ + F_basolateral_)].

Experiments were performed in triplicates on different days using three independently grown cell cultures.

### In vitro BBB model integrity assay

After the translocation assay, an in vitro BBB integrity assay was performed. Herein, cells were washed twice with 1X PBS and once with DMEM:F12 without phenol red. Then, previously diluted TRITC-Dx4 was added to the apical side and incubated for 2 h. TRITC-Dx4 was diluted in DMEM:F12 without phenol red to an absorbance below 0.1. Finally, samples from the apical and basolateral side were collected and fluorescence intensity analyzed using a Varioskan^™^ LUX multimode microplate reader. The percentage of TRITC-Dx4 recovered was determined using the following equation:

3 $$TRITC\_Dx4 Permeability \left(\%\right)=\left(\frac{{F}_{i}-{F}_{cells}}{{F}_{TRITC-Dx4}-{F}_{Medium}}\right)\times 100$$

where F_i_, F_cells_, F_TRITC-Dx4_ and F_Medium_ are defined as above.

The integrity of the in vitro BBB model is indirectly proportional to the percentage of TRITX-Dx4 recovered and was determined using the following equation:

4 $$Integrity\left(\%\right)=100-TRITC\_Dx4 Permeability (\%)$$

### Cytotoxicity towards a panel of human cell lines

Peptide cytotoxicity was determined using the CellTiter-Blue^®^ cell viability assay, following a described protocol [24, 26]. Briefly, all cell lines were carefully harvested with trypsin–EDTA and seeded 10.000–20.000 cells/100 μL into 96-well clear flat-bottomed polystyrene plates (Corning, USA) for 24 h. After medium removal, cells were washed twice with 1X PBS, and 100 µL of previously diluted peptides (range from 0.05–100.0 µM) in the respective medium were added to cells. Then, after 24 h, cells were washed twice with 1X PBS and 20 µL of CellTiter-Blue^®^ Reagent (diluted in 100 µL of medium) was added to each well and incubated for 3 h in culturing conditions. The fluorescence intensity was measured using Varioskan^™^ LUX multimode microplate reader.

IC_50_ values were determined using GraphPad Prism 7.0 software using a log(inhibitor) *versus* normalized response. Experiments were performed in triplicates on different days using three independently grown cell cultures.

### Internalization across human cell lines

The ability of peptides to cross cellular membranes was evaluated using a previously described protocol with minor alterations [25]. Briefly, cells were harvested with trypsin–EDTA and seeded at 50,000 cells/500 µL into 24-well clear flat-bottomed polystyrene plates (Corning, USA) and incubated in their respective medium for 24 h. After medium removal, cells were washed twice with 1X PBS, and 5.0 µM of CF-peptides predissolved in the respective medium were added. After 24 h, cells were washed twice with 1X PBS, harvested, and washed again twice with 1X PBS. The fluorescence intensity of 10,000 cells, measured with a BD LSRFortessa X-20 flow cytometer (BD Biosciences, USA), defines the ratio between the mean fluorescence of a sample and that of untreated cells.

Experiments were performed in triplicates on different days using three independently grown cell cultures.

### Biodistribution

All animal experiments were performed in compliance with national and EU legislation for good practices on laboratory animal science. The animals were housed in a temperature and humidity-controlled environment with a 12 h light/12 h dark schedule. Biodistribution of radiolabeled peptides was performed on 7 weeks old CD1 female mice.

Animals were intravenously injected into the tail vein with 100 µL of a 25 µM saline solution of the ^67^Ga-peptide with a radioactivity around 100 µCi. The mice were euthanized by cervical dislocation at 2 min and 1 h after injection. The dose administered and the radioactivity in the euthanized animals was measured using a dose calibrator (Carpintec CRC-15W, Ramsey, USA). The difference between the radioactivity in the injected and the euthanized animals was assumed to be due to excretion. Brain and tissues of interest without previous perfusion were dissected, washed, and weighed, and their radioactivity was measured using a γ counter (Hidex AMG, Hidex, Turku, Finland). The uptake in the brain and tissues of interest was calculated and expressed as a percentage of injected radioactivity dose per gram of tissue (% ID/g).

### Statistical analysis

Quantitative data were processed using Excel 2013 (Microsoft, USA) and the GraphPad Prism version 7.0 software (USA). Medians, means, and standard deviations are shown in figures. Pairwise significances were calculated using one-way ANOVA followed by Tukey’s or Dunnett’s multiple comparison test, and nonparametric Mann–Whitney, or unpaired *t*-tests.

## Results and discussion

The use of CPPs to deliver protein, nucleic acid, small drug, nanoparticle, and other payloads across cell membranes has been intensely studied in the last three decades, for its potential biotechnological and medical applications [5]. The main physicochemical properties of this rather diverse class of peptides have been identified, while the actual internalization mechanisms remain nuclear [27]. On the other hand, some peptides are able to traverse physiological barriers, such as the endothelial BBB have been more recently reported. These BBB-crossing peptides do not simply engage in cell membrane interaction, via AMT or RMT, but can achieve transcytosis through other distinct pathways, such as paracellular, direct diffusion or carrier-mediated transport [4, 13, 28–31]. However, as the main physicochemical features that allow BBB transcytosis have not been investigated in sufficient detail, researchers may rely excessively—and controversially—on analogy in assuming that CPPs are likewise able to traverse cell barriers [4, 32, 33].

### Selection of BBBpS and non-BBBpS

We have devised a multi-step methodology to shed light on the physicochemical characteristics enabling BBBpS to traverse cell barriers. To this end, we have generated databases for most published BBBpS (Table S2) and CPPs (Table S3) (71 and 521 entries, respectively). The size difference between both databases suggests that research on BBBpS lags behind that of CPPs. In the former database we have then included physicochemical properties easily evaluated from the peptide sequence [23] and found that, overall, BBBpS have: (i) a small size (average MW of 2046 g mol^−1^), (ii) none or few aromatic residues (average molar absorptivity of 3790 M^−1^.cm^−1^ at 280 nm, corresponding to 1–2 Tyr or 0–1 Trp residues), (iii) a slight hydrophobic nature ($\underline{x}$ =35%—mean content in hydrophobic residues), and (iv) a slightly cationic charge (average net charge of + 2). Cutoff values, defining symmetrical intervals enclosing 60% of each BBBpS parameter (relative to the mean), are shown in Table S4. A 60% value was chosen because it accommodates most peptides without being affected by outliers. Figure 1 shows an example for the “net charge at pH 7.0” criterion. In this case, the average net charge is 2.1 and 60% of BBBpS within the -1.0 to 4.7 interval.

The following step in our work was identifying, within the large CPP family, peptides most likely to perform as BBBpS. To this end, the 521 entries of the CPP database were screened against nine physicochemical parameters for which statistically relevant cutoffs had previously been defined within the BBBpS set (Fig. 1) to identify those qualify as BBBpS. It should be stressed that we made use of statistical tools, therefore reporting trends and tendencies. The result was a subgroup of 14 CPPs with presumable BBBpS potential. This selection involved the exclusion of all other 504 entries (~ 98%) in the CPP database, which strongly suggests that the overlap between CPP and BBBpS families is indeed small. Consequently, brain-targeting drug delivery strategies based on “chemical intuition” assumptions of alleged CPP-BBBpS equivalence are ill-founded and likely to be proven unrealistic. This is a disruptive result in the face of "conventional wisdom" prevalent in the literature of the last 20 years, hence seems to call for a revision of the paradigm.

The peptides selected in both databases were then ranked using the OLS method [34] and the ten best-ranking BBBpS candidates and three non-BBBpS negative controls were synthesized and experimentally tested for their ability to transverse the BBB model (Table 1). Results were compared to PepH3, a well-tested BBBpS standard.

### Translocation across an in vitro BBB model

Seeking experimental support for our hypothesis, we have synthesized and tested in vitro the activity of ten peptides (Table 1), selected among the 14 BBBpS entries by their lowest *S* score in an OLS ranking [34]*.* We used an in vitro model with a HBEC-5i cell monolayer to assess the BBB translocation of the peptides [24, 25]. Low paracellular leakage and expression of tight junctions that validate this model have been previously reported by our group [26]. In this simple, quick and robust molecular screen [11, 24–26, 35], the fluorescence intensity measured on both the apical and basolateral compartments of the device is readily converted into the rate of cellular barrier crossing by the peptide under study. The stability of peptides is kept throughout the experiment, as stability assays using culturing conditions (medium, 37 ℃ and 5% CO2) show no evidence of degradation (Figure S1).

Most BBBpS candidates tested positive in this in vitro BBB model, with four of them, namely, BBBpS_1, BBBpS_2, BBBpS_5, and BBBpS_9 being highly active (Table 2 and Figure S2A), similar to PepH3, a well-characterized and efficacious BBBpS [11, 24–26, 35, 36]. For PepH3 a translocation of 54.0 ± 2.2% was achieved after 24 h incubation. As for the BBBpS studied, we observed translocation levels above 30%, except for BBBpS_7 (20.3 ± 5.1%). Interestingly, some of the BBBpS selected had similar or higher BBB permeability than PepH3, e.g., BBBpS_1 (61.4 ± 2.3%), BBBpS_2 (41.4 ± 3.9%), BBBpS_5 (53.0 ± 4.7%), and BBBpS_9 (46.0 ± 4.3%). Conversely, non-BBBpS displayed poor endothelial cell barrier translocating abilities, e.g., for non-BBBpS_2 gave 2.6 ± 1.2% and for non-BBBpS_3 gave 11.1 ± 2.6%, and for non-BBBpS_1 gave 20.0 ± 3.5%.

**Table 2:** Summary of the cell-based assays

Peptide	Cytotoxicity (µM)^a^					Internalization (relative fluorescence intensity)^b^					Translocation (%)^c^
	HBEC-5i	HeLa	Hs68	MDA-MB-231	HEK-293	HBEC-5i	HeLa	Hs68	MDA-MB-231	HEK293	HBEC-5i
	IC_50_	IC_50_	IC_50_	IC_50_	IC_50_						
BBBpS_1	> 100.0	> 100.0	> 100.0	> 100.0	> 100.0	19.6 ± 0.4	11.9 ± 0.0	10.3 ± 0.7	6.9 ± 1.2	27.5 ± 6.6	61.4 ± 2.3
BBBpS_2	> 100.0	> 100.0	> 100.0	> 100.0	> 100.0	67.5 ± 7.9	18.3 ± 1.5	50.7 ± 6.9	23.5 ± 5.6	35.5 ± 6.5	41.4 ± 3.9
BBBpS_3	> 100.0	> 100.0	> 100.0	> 100.0	> 100.0	18.7 ± 0.7	13.4 ± 0.3	9.8 ± 1.0	8.4 ± 0.9	43.8 ± 10.6	35.7 ± 3.6
BBBpS_4	> 100.0	> 100.0	> 100.0	> 100.0	> 100.0	8.9 ± 0.3	8.9 ± 1.1	6.5 ± 0.5	6.4 ± 0.9	8.1 ± 2.5	33.4 ± 3.5
BBBpS_5	> 100.0	> 100.0	> 100.0	> 100.0	> 100.0	15.4 ± 1.3	23.2 ± 5.4	8.8 ± 0.5	17.3 ± 3.7	24.5 ± 4.2	53.0 ± 4.7
BBBpS_6	> 100.0	> 100.0	> 100.0	> 100.0	> 100.0	8.0 ± 0.9	7.9 ± 0.1	6.7 ± 0.9	5.7 ± 1.2	8.7 ± 4.0	35.6 ± 3.9
BBBpS_7	> 100.0	> 100.0	> 100.0	> 100.0	> 100.0	36.6 ± 4.5	83.5 ± 27.8	32.9 ± 3.3	26.4 ± 4.5	28.0 ± 7.2	20.3 ± 5.0
BBBpS_8	> 100.0	> 100.0	> 100.0	> 100.0	> 100.0	13.0 ± 0.2	21.8 ± 2.7	11.0 ± 1.4	12.4 ± 0.6	13.4 ± 0.7	35.4 ± 8.5
BBBpS_9	> 100.0	> 100.0	> 100.0	> 100.0	> 100.0	13.8 ± 0.5	16.6 ± 0.1	11.6 ± 0.7	6.8 ± 0.7	23.3 ± 3.0	46.0 ± 4.3
BBBpS_10	> 100.0	> 100.0	> 100.0	> 100.0	> 100.0	10.7 ± 0.9	9.4 ± 0.0	6.9 ± 0.7	8.8 ± 0.3	13.5 ± 2.1	38.4 ± 5.3
non-BBBpS _1	> 100.0	> 100.0	> 100.0	> 100.0	> 100.0	1577.7 ± 251.1	174.8 ± 60.3	225.3 ± 17.3	219.8 ± 14.9	895.0 ± 45.6	20.0 ± 3.5
non-BBBpS _2	20.7 ± 1.0	39.4 ± 1.8	26.4 ± 2.9	26.9 ± 3.0	14.7 ± 1.4	656.7 ± 52.6	268.3 ± 9.0	307.1 ± 27.2	237.4 ± 36.9	462.4 ± 65.9	2.6 ± 1.2
non-BBBpS _3	> 100.0	> 100.0	> 100.0	> 100.0	> 100.0	212.8 ± 24.3	187.0 ± 15.1	254.0 ± 29.2	157.0 ± 28.5	269.0 ± 61.8	11.1 ± 2.6

^a^determined using CellTiter-Blue® assay

^b^evaluated by fluorescence intensity using flow cytometry

^c^evaluated by fluorescence intensity using a plate reader

IC_50_, concentration that causes cell death in 50% of cells

None of the peptides increased paracellular permeability in the in vitro BBB model (HBEC-5i integrity > 90.0%—Figure S2B). The difference between all peptides and control was not statistically significant (*p* > 0.05).

**Fig. 2:**
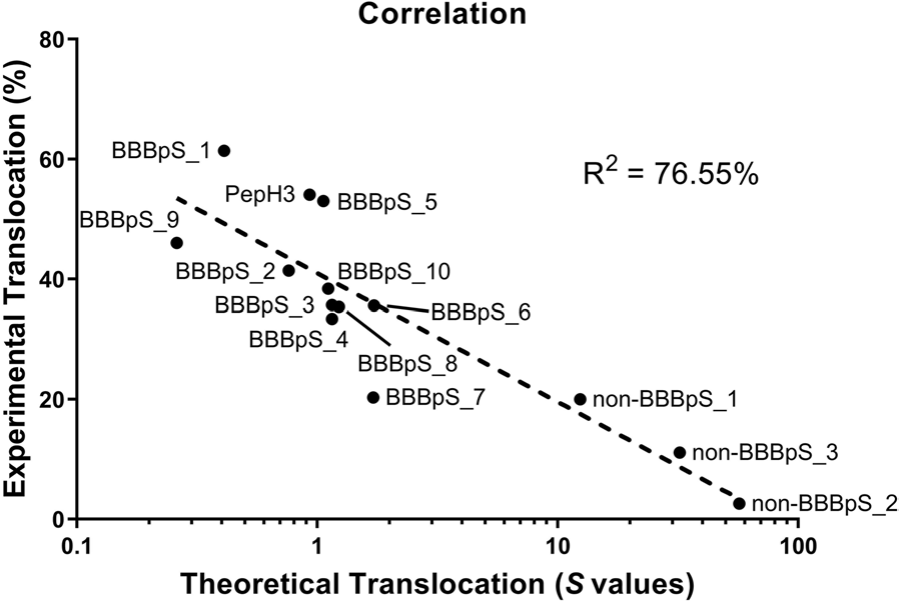
Correlation between theoretical and experimental translocation. The theoretical values of the translocation based on the ordinary Least Squares Method (S) were plotted against the translocation (%) obtained from our in vitro BBB model. Then, a correlation analysis was performed using a linear regression

Importantly, the experimental results show a high correlation with the *S* values obtained in the prior OLS ranking. Peptides with lower *S* scores corresponding to the most active BBBpS and those (non-BBBpS) with high *S* values being unable to effective cross the BBB (Fig. 2). These results altogether corroborate the negative correlation between translocation capacity and *S* (Fig. 2). Finally, it must be noted that the peptide concentration (5.0 μM) used in all translocation assays is well below the IC_50_ toxicity values determined on a panel of human cell lines.

### Toxicity towards a human cell line panel

In vitro peptide cytotoxicity was studied on a panel of cell lines commonly used in preclinical studies to assess translocation (HBEC-5i), toxicity (HeLa, MDA-MB-231, and Hs68), and transfection (HEK-293). The CellTiter-Blue^®^ assay revealed that for most peptides, with the exception of non-BBBpS_2, with a minimum IC_50_ of 14.7 ± 1.4 µM in HEK-293 cells and a maximum of 39.4 ± 1.8 µM in HeLa cells, there was no toxicity up to 100.0 µM (Table 2 and Figure S3).

### Internalization into human cell lines

We also evaluated peptide ability to internalize a cell line panel commonly applied in preclinical toxicity studies (Table 2 and Figure S3). The potential BBBpS candidates revealed in all cases an ability to accumulate inside cells. BBBpS_2, BBBpS_5, and BBBpS_7 showed the highest internalization (RFI > 20.0), with no particular selectivity, while BBBpS_3 had some selectivity towards HEK-293 cells (RFI > 40.0). BBBpS_4, BBBpS_6, and BBBpS_10 had the lowest uptakes (RFI < 10.0). The three negative control peptides were internalized successfully (RFI > 150.0), confirming their CPP nature, non-BBBpS_1 and non-BBBpS_2 being the most efficient and showing selectivity towards HBEC-5i and HEK-293 cells.

Overall, peptides internalize more in HEK-293 than in other lines, which is not surprising as CPPs are commonly designed and applied for transfection [37] and HEK-293 is extensively used for protein expression [38, 39]. In contrast, the lowest internalization was observed in MDA-MB-231 cells, which are triple-negative breast cancer metastatic cells able to colonize the brain. These cells have a membrane with different lipid and protein composition compared to healthy cells [40], which leads to different membrane charge, fluidity, and rigidity, factors that might affect peptide internalization.

Results clearly show the difference between BBBpS and non-BBBpS. For non-BBBpS, high internalization (RFI > 200.0) was obtained regardless of cell type, while BBBpS only moderate (8.0 < RFI < 200.0) internalization is obtained for non-healthy cells and low internalization for HBEC-5i and Hs68 (healthy cells) confirming the CPP nature of non-BBBpS.

### Biodistribution

The most promising BBBpS, namely BBBpS_1, BBBpS_2, BBBpS_5, and BBBpS_9, were selected for in vivo biodistribution studies in healthy mice using ^67^Ga-radiolabed peptide derivatives. A non_BBBpS, non-BBBpS_2, was used as a negative control to validate the capacity to discriminate potential BBBpS from non_BBBpS. The use of a ^67^Ga-NODA-GA moiety as a peptide tracer slightly increases peptide hydrophobicity (Table 1), which might influence the peptides internalization or permeation across the BBB.

The biodistribution profile of ^67^Ga-BBBpS_1, ^67^Ga-BBBpS_2, ^67^Ga-BBBpS_5, ^67^Ga-BBBpS_9, and ^67^Ga-non-BBBpS_2 including brain uptake is shown in Table 3. The brain uptake for ^67^Ga-BBBpS_1 (0.66 ± 0.16% ID/g), ^67^Ga-BBBpS_2 (0.52 ± 0.23% ID/g), ^67^Ga-BBBpS_5 (0.74 ± 0.01% ID/g), and ^67^Ga-BBBpS_9 (0.61 ± 0.11% ID/g) at 2 min post-injection is, respectively, 2.4-, 1.9-, 2.7-, and 2.3-fold higher than the brain accumulation of ^67^Ga-non-BBBpS_2 (0.27 ± 0.04% ID/g) at 2 min post-injection. It is worth stressing that the method selects BBBpS among CPPs, i.e. peptides already prone to cell penetration. In this context, and also bearing in mind that 98% of drugs cannot traverse the BBB [41], a 2 to threefold increase in brain penetration must be deemed quite significant and highlights the relevance of the present work. For all BBBpS, the in vivo data showed a rapid (up to 2 min. after injection) and a brain accumulation above 0.5% ID/g, followed by a quick brain washout (< 0.1% ID/g after 1 h), and clearance from all organs. Our BBBpS demonstrated a potent brain targeting, achieving even higher values than the antibody fragment FC5 (0.5% ID/g), which has been widely used as a reference [11, 42]. Indeed, all four BBBpS overperformed the best brain-targeting molecules in the literature such as Tat, penetratin, Angiopep-2, dNP2, TP10, MiniAp-4, and PepH3, all with brain accumulation levels ranging between 0.25 and 0.50% ID/g [10, 11, 43–48]. In addition, the selected BBBpS present high excretion rate (> 75% ID/g at 1 h post-injection), which is an important feature to avoid toxicity associated to accumulation in the main organs. This characteristic ensures that all BBBpS not only can be used as an active carrier to the brain, but can also be an active shuttle in-and-out the brain. For non-BBBpS_2, our results demonstrate a lower brain accumulation (0.27 ± 0.04% ID/g after 2 min post-injection), followed by a more slowly brain washout (0.13 ± 0.03% ID/g after 1 h), and high accumulation in the liver (22.8 ± 10.1% ID/g after 1 h). These data, as well as the in vitro data for BBB cell retention (calculated as the non-apical and non-basolateral fraction of the total) and for cell internalization sustain that we see brain accumulation. We do not expect the BBBpS are only captured in the endothelium as in vitro data shows that the non-BBBpS present higher retention than any BBBpS (Figure S2) and non-BBBpS internalization in all cells tested is much higher than for any BBBpS.. Interestingly, internalization in brain endothelial cells (HBEC-5i) is much higher for any BBBpS (Figure S4).

**Table 3:** Biodistribution Profiles of the ^67^Ga-labeled Peptides^a^

Organ	^67^ Ga-BBBpS_1		^67^ Ga- BBBpS_2		^67^ Ga- BBBpS_5		^67^ Ga- BBBpS_9		^67^ Ga-non-BBBpS_2	
	2 min	1 h	2 min	1 h	2 min	1 h	2 min	1 h	2 min	1 h
Blood	12.6 ± 2.1	0.5 ± 0.3	13.4 ± 3.8	1.4 ± 0.9	10.7 ± 2.1	1.2 ± 0.3	11.6 ± 1.5	0.8 ± 0.4	9.3 ± 6.3	0.9 ± 0.6
Liver	4.1 ± 0.8	0.3 ± 0.2	3.5 ± 1.2	0.6 ± 0.2	5.2 ± 1.1	1.0 ± 0.4	4.7 ± 0.4	2.6 ± 0.3	19.8 ± 0.7	22.8 ± 10.1
Intestine	1.7 ± 0.3	0.2 ± 0.1	1.5 ± 0.2	0.4 ± 0.0	2.2 ± 0.2	0.4 ± 0.2	1.9 ± 0.2	0.2 ± 0.0	2.7 ± 0.7	1.6 ± 0.5
Spleen	2.0 ± 0.2	0.2 ± 0.1	1.9 ± 0.6	0.5 ± 0.2	2.2 ± 0.1	0.3 ± 0.1	2.6 ± 0.3	0.9 ± 0.2	5.6 ± 0.4	3.7 ± 1.8
Heart	3.4 ± 1.0	0.3 ± 0.1	3.1 ± 0.7	0.8 ± 0.1	3.8 ± 0.8	0.5 ± 0.0	3.3 ± 0.1	0.3 ± 0.0	6.0 ± 0.8	1.7 ± 0.1
Lung	6.1 ± 0.7	2.5 ± 0.3	6.0 ± 1.2	1.7 ± 0.4	7.8 ± 2.8	1.2 ± 0.2	5.4 ± 0.6	0.7 ± 0.1	7.6 ± 1.5	2.4 ± 0.7
Kidney	33.8 ± 3.8	2.4 ± 1.8	18.1 ± 2.1	1.4 ± 0.6	22.0 ± 3.6	1.1 ± 0.6	30.0 ± 3.7	3.1 ± 0.3	19.9 ± 0.4	5.4 ± 0.8
Muscle	2.4 ± 0.2	0.3 ± 0.1	2.0 ± 0.4	0.7 ± 0.1	3.1 ± 0.8	0.5 ± 0.2	2.6 ± 0.3	0.2 ± 0.1	1.8 ± 0.5	1.2 ± 0.4
Bone	3.4 ± 0.3	0.3 ± 0.1	3.1 ± 0.1	0.8 ± 0.5	3.8 ± 0.5	0.6 ± 0.4	3.4 ± 0.3	0.5 ± 0.1	3.7 ± 0.5	1.6 ± 0.1
Stomach	1.4 ± 0.5	0.1 ± 0.0	1.0 ± 0.1	0.2 ± 0.1	2.6 ± 0.0	0.3 ± 0.4	1.5 ± 0.2	0.3 ± 0.1	2.1 ± 0.6	1.7 ± 0.6
Brain	**0.66 ± 0.16**	**0.04 ± 0.02**	**0.52 ± 0.23**	**0.09 ± 0.03**	**0.74 ± 0.01**	**0.05 ± 0.01**	**0.61 ± 0.11**	**0.05 ± 0.01**	**0.27 ± 0.04**	**0.13 ± 0.03**
Excretion (% ID)	–	79.3 ± 3.3	–	77.7 ± 2.6	–	78.3 ± 4.7	–	82.0 ± 6.1	–	13.5 ± 5.1

^a^Tissue distribution of ^67^ Ga-BBB_1, ^67^ Ga-BBB_2, ^67^ Ga-BBB_5, ^67^ Ga-BBB_9, and ^67^ Ga-non-BBBpS_2 at 2 min and 1 h post injection via tail vein in CD1 mice. Results are expressed as the average of percentage of injected dose (ID) per gram of tissue (%ID/g tissue; mean ± SD), n = 3

Taken together, meta-analysis, in vitro and in vivo data suggest that a CPP is not necessarily a good BBBpS. Moreover, all BBBpS have fast clearance from blood primarily through renal excretion (high kidney uptake at 2 min followed by rapid elimination at 1 h timepoint) and rapid brain washout (< 0.1% ID/g after 1 h post-injection), in accordance with fast radioactivity elimination from most organs. In addition, they all present high excretion rate (> 75% at 1 h post-injection). Unlike the ^67^Ga-BBBpS tested, the biodistribution profile of the ^67^Ga- non-BBBpS_2 is different. It has also a fast blood clearance and an important radioactivity fraction is eliminated via the urinary pathway, as suggested by the kidney uptake. However, high liver uptake and retention was found (19.8 ± 0.7 and 22.8 ± 10.1% ID/g at 2 min and 1 h post injection, respectively) indicating an important contribution of the hepatobiliary tract on the elimination of this radiolabeled peptide. In agreement with this finding, the total radioactivity excretion was much lower, 13.5 ± 5.1% ID/g at 1 h after administration.

To sum up, the results suggest that all BBBpS reported here present higher brain accumulation in vivo when compared to other known BBBpS, while non-BBBpS behave rather differently, not only in terms of brain accumulation but also of overall biodistribution. However, to confirm BBB translocation future studies can consider isolation of brain parenchyma, or perfusion tissues before isolation. Among the different characteristics, the small size of peptides (MW < 3000 g.mol^−1^) can be hypothesized as an issue when shuttling large cargoes, since it can restrict the interaction between BBBpS and endothelial cells. The use of linkers, delivery systems with more than one BBBpS, and smaller cargoes are strategies to overcome this possible drawback. Nevertheless, our preclinical results demonstrate that BBBpS are capable of penetrating the brain conjugated to large cargoes [26, 36].

## Conclusions

Although the main physicochemical characteristics underlying BBBpS activity remain elusive, intuitive approaches naïvely tend to assume all CPPs as potential BBBpS, in a perspective where BBB translocation is viewed as a sequential crossing of cellular membranes. In this study, we devised and applied a quantitative methodology that combines meta-analysis and statistical reasoning, and eloquently demonstrate that, based on intrinsic structural features, i.e., small size, few aromatic residues, and slightly hydrophobic and slight cationic nature, very few CPPs are indeed BBBpS, in practice demonstrating that both peptide families should better be separately viewed. In addition, our work has identified four BBBpS with high translocation abilities in vitro and higher brain accumulation in vivo compared to other known BBBpS.

The internalization capacity of CPPs can be exploited to shuttle payloads across membranes into the cytoplasm or the nucleus while, in using BBBpS for brain delivery, researchers may expect endothelial barrier translocation. Thus, instead of cargo delivery to a primary target cell, a BBBpS can be expected to transcytose an endothelial cell and deliver a given payload at a target site in the brain parenchyma. These complementary behaviors will no doubt continue to be further explored by researchers aiming at more efficient delivery systems for therapeutic purposes.

### Supplementary Information

Supplementary Material 1.

## Data Availability

The datasets generated during the current study are available in the supplementary material.

## Abbreviations

ACN: Acetonitrile
AF: Attachment factor protein solution
BBB: Blood–brain barrier
BBBpS: BBB peptide shuttles
CF: 5(6)-Carboxyfluorescein
CPPs: Cell-penetrating peptides
DCM: Dichloromethane
DIEA: *N,N*-Diisopropylethylamine
DIPCI: *N,N*-Diisopropylcarbodiimide
DMEM: Dulbecco’s modified Eagle medium
DMF: *N,N*-Dimethylformamide
DODT: 3,6-Dioxa-1,8-octanedithiol
ECGS: Endothelial cell growth supplement
EMEM: Minimum essential medium Eagle
FBS: Fetal bovine serum
HBTU: 1,3,3-Tetramethyluronium hexafluorophosphate
HOBt: *N*-Hydroxybenzotriazole
MBHA: Fmoc-Rink amide
OLS: Ordinary least squares
SPPS: Solid-phase peptide synthesis
TAT: Transactivator of transduction
TFA: Trifluoroacetic acid
TIS: Triisopropylsilane
TRITC-Dx4: Tetramethylrhodamine isothiocyanate-4 KDa dextran
