# Supplementary material for: Molecular determinants for brain targeting by peptides: a meta-analysis approach with experimental validation
Source: Fluids Barriers CNS. 2024 May 27;21:45. doi: 10.1186/s12987-024-00545-5 (PMC11131246; doi:10.1186/s12987-024-00545-5)
Supplement: Supplementary file 1 — Supplementary Material 1. [file 12987_2024_545_MOESM1_ESM.docx]

**Table of Contents**

[**Table S1. List of the journals selected for the meta-analysis.** 2](#_Toc153780902)

[**Table S2. Features of published BBB peptide shuttles.** 3](#_Toc153780903)

[**Table S3. Features of published cell-penetrating peptides.** 7](#_Toc153780904)

[**Table S4. Limits selected for each parameter using the BBB peptide shuttle database.** 40](#_Toc153780905)

[**Figure S1. Stability of peptides in culturing conditions.** 500 µM 5(6)-CF-peptides solution in DMEM:F12 without phenol-red (Gibco/Thermo Fischer (USA) were incubated at 37°C and 5% CO2. Then, 150 µl aliquots were taken at different timepoints and analyzed by analytical RP-HPLC using linear gratients of solvent B (0.1% TFA in ACN) into solvent A (0.1% TFA in H2O). The detection was made using a (**A**) SPD-M40 PDA detection (λ 220 nm) and (**B**) RF-20Axs Spectrofluorometric detector (λ_ex_ 495/λ_em_ 520 nm) (Shimadzu, Japan). Percent of intact peptide was calculated by peak integration, expressed as percent of the amount at t0. 41](file:///E:\3.%20Papers\BBBpS%20vs%20CPPS\Submission\7.%20Fluids%20and%20Barriers%20of%20the%20CNS\Comments\Supplementary%20information_track%20changes.docx#_Toc160187124)

[**Figure S2.** **Translocation of 5(6)-peptides across an in vitro BBB model and the TRITX-Dx4 permeability study. (A)** Percentage of translocation of 5(6)-CF-peptides (5.0 µM). **(B)** Fluorescence intensity of TRITC-Dx4 as a measured of BBB integrity after peptide exposure after translocation assay. The values were obtained from triplicates of three independent experiments. PepH3 was used as a BBBpS control. Error bars, S.D. 42](file:///E:\3.%20Papers\BBBpS%20vs%20CPPS\Submission\7.%20Fluids%20and%20Barriers%20of%20the%20CNS\Comments\Supplementary%20information_track%20changes.docx#_Toc160187125)

[**Figure S3.** **in vitro cytotoxicity of all peptides towards a panel of different cell lines**. HBEC-5i, Hs68, HeLa, MDA-MB-231, and HEK-293 cells treated with different concentrations of peptides (0.05 – 100.0 µM) for 24h. The percentage of viable cells was determined using CellTiter-Blue® reagent assay. The values were obtained from triplicates of three independent experiments. Error bars, S.D. 43](file:///E:\3.%20Papers\BBBpS%20vs%20CPPS\Submission\7.%20Fluids%20and%20Barriers%20of%20the%20CNS\Comments\Supplementary%20information_track%20changes.docx#_Toc160187126)

[**Figure S4.** **in vitro internalization of all 5(6)-peptides towards a panel of different cell lines.** HBEC-5i, Hs68, HeLa, MDA-MB-231, and HEK-293 cells exposed to 5(6)-CF-peptides (5.0 µM) for 24h. The relative fluorescence intensity (RFI) was determined using flow cytometry. The values were obtained from triplicates of three independent experiments. Error bars, S.D. 44](file:///E:\3.%20Papers\BBBpS%20vs%20CPPS\Submission\7.%20Fluids%20and%20Barriers%20of%20the%20CNS\Comments\Supplementary%20information_track%20changes.docx#_Toc160187127)

**Table S1. . List of the journals selected for the meta-analysis.**

| Antimicrob Agents Chemother | Biochem J | FEBS J | J Phys Chem | Nat Chem Biol |
| --- | --- | --- | --- | --- |
| ACS Chem Biology | Biochemistry | Front Microbiol | J Virol | Peptides |
| ACS Chem Neurosci | Biomater | Front Pharmacol | J Am Chem Soc | PLoS One |
| AIDS | Biophys J | J Bio Chem | Langmuir | PLoS Pathog |
| Amino Acids | Biopolymers Pept Sci | J Control Release | Mol Oncol | Sci Rep |
| BBA Mol Cell Biol | ChemBioChem | J Med Chem | Mol Pharm |  |
| BBA-Biomembranes | ChemMedChem | J Peptide Sci | Nanoscale |  |

**Table S2. Features of published BBB peptide shuttles.**

| **Peptide** | **Typical sequence** | **Main cargoes** | **BBB model** | **Pathology** | **Proposed translocation** | **Physicochemical properties** | | | | | | | | | **Ref.** |
| --- | --- | --- | --- | --- | --- | --- | --- | --- | --- | --- | --- | --- | --- | --- | --- |
|  |  |  |  |  |  | **Molecular weight**  **(g/mol)** | **Extinction coefficient**  **(M^-1^.cm^-1^)** | **Hydrophobic**  **(%)** | **Isoelectric point** | **Net charge**  **(pH 7)** | **Charge** | **Average hydrophobicity** | **Hydrophobicity**  **(pH 7)** | **Ratio hydrophilic residues**  **(%)** |  |
| - | GLHTSATNLYLH |  | - rat PBCECs |  |  | 1325,57 | 1280 | 41,67 | 10,03 | -0,78 | 2 | -0,8 | 33,33 | 17 | [1] |
| - | VAARTGEIYVPW |  | - rat PBCECs |  |  | 1360,64 | 6970 | 58,33 | 10,02 | -1 | 0 | -0,5 | 34,58 | 17 | [1] |
| - | LMWP | NPs | - bEnd.3s  - mice | - Brain tumors |  | 544,74 | 5690 | 75 | 13,8 | -1 | 0 | -1,6 | 56,25 | 0 | [2] |
| #2077 | RLSSVDSDLSGC | NPs | - Rats |  |  | 1237,4 | 120 | 33,33 | 6,05 | -2,09 | -1 | 0,3 | 15,08 | 58 | [3] |
| AGBBB015F | CGGKTFFYGGSRGKRNNFKTEEY | NPs | - BBMVECs  - rats | - Brain tumors |  | 2646,05 | 2680 | 26,09 | 10,31 | 1,91 | 3 | 0,3 | 10,7 | 43 | [4] |
| Angiopep-2 | TFFYGGSRGKRNNFKTEEY | Proteins, peptides, NPs, small drugs, nucleic acids | - bEnd.3s  - BBCEC  - PBCECs  - BMVEs  - BCECs  - Mice  - Rats  - Humans | - Brain tumors  - Lysosomal storage disorders  - Neurodegenerative disorders  - Epilepsy | LRP1 | 2300,61 | 2560 | 26,32 | 10,47 | 1 | 2 | 0,3 | 11,58 | 47 | [5-34] |
| D-Angiopep-2 | cyeetkfnnrkGrsGGyfft | Small drugs | - bEnd.3s  - Mice | - Brain tumors | LRP1 | 2300,61 | 2560 | 26,32 | 10,47 | 1 | 2 | 0,3 | 11,58 | 47 | [35] |
| Apamin | CNCKAPETALCARRCQQH | NPs | - BBB-RTU  - Mice | - Spinal cord injury |  | 2032,43 | 480 | 44,44 | 8 | 1,75 | 3 | 0,2 | 13,94 | 39 | [36,37] |
| ApoB | SVIDALQYKLEGTTRLTRKRGLKLATALSLSNKFVEGS | Proteins | - PBCECs  - Mice | - Lysosomal storage disorders | LDLR | 4165,08 | 1280 | 39,47 | 11,07 | 3 | 4 | 0,2 | 25,37 | 42 | [10,38,39] |
| ApoE-I | TEELRVRLASHLRKLRKRLLRDA | Proteins | - PBCECs  - Mice | - Lysosomal storage disorders | LRP1 | 2829,52 | 0 | 39,13 | 12,5 | 4,11 | 6 | 0,8 | 22,91 | 52 | [10] |
| ApoE-II | LRKLRKRLLLRKLRKRLL | Proteins, NPs | - PBCECs  - BBCECs  - RBE4s  - hCMEC/D3  - Mice | - Lysosomal storage disorders  - Neurodegenerative disorders | LRP1 | 2372,25 | 0 | 44,44 | 13,34 | 10 | 10 | 0,9 | 34,67 | 56 | [10,40-42] |
| ApoE-III | LRKLRKRLLLR | NPs | - Mice |  | LRP1 | 1464.98 | 0 | 45.45 | 12.97 | 6 | 6 | 0.8 | 36.18 | 55 | [43] |
| ApoO | CNCKAPETALCA[Orn][Orn]CQQH |  | - BBB-RTU |  |  | 2176,65 | 480 | 50 | 6,68 | -0,25 | 1 |  | 17,44 |  | [36] |
| K16-ApoE | KKKKKKKKKKKKKKKKLRVRLASHLRKLRKRLLRDA | Small drugs | - Rats | - Brain tumors |  | 4521,05 | 0 | 25 | 13,36 | 22,11 | 24 | 1,7 | 5,75 | 72 | [44] |
| B6 | CGHKAKGPRK | NPs | - bEnd.3s | - Neurodegenerative disorders | TfR1 | 1080,37 | 120 | 20 | 11,86 | 3,02 | 5 | 1 | -3,1 | 40 | [45,46] |
| AchR, Acetylcholine receptor; AMT, Adsorptive-mediated transport; BBB, Blood-brain barrier; BBBCM, cell-based BBB model; BBB-RTU, Blood-brain barrier ready-to-use device; BBCECs, Bovine brain capillary endothelial cells; BBMVECs, Bovine brain microvascular endothelial cells; BCECs, Brain capillary endothelial cells; bEnd.3, Brain endothelial cells; BMEC, Brain microvascular endothelial cells; HBEC-5i, Human brain endothelial cell line; hCMECs, Human cerebral microvascular endothelial cells; LDLR, Low density lipoprotein receptor; LRP, Lipoprotein receptor-related protein; MSC, Mesenchymal stem cells; NPs, Nanoparticles; NRP-1, Neuropilin-1; PBCECs, Porcine brain capillary endothelial cells; RBE4s, Rat brain endothelial cells; RMT, Receptor-mediated transport; TfR, Transferrin receptor; | | | | | | | | | | | | | | | |

**Table S2. Features of published BBB peptide shuttles (cont.).**

| **Peptide** | **Typical sequence** | **Main cargoes** | **BBB model** | **Pathology** | **Proposed translocation** | **Physicochemical properties** | | | | | | | | | **Ref.** |
| --- | --- | --- | --- | --- | --- | --- | --- | --- | --- | --- | --- | --- | --- | --- | --- |
|  |  |  |  |  |  | **Molecular weight**  **(g/mol)** | **Extinction coefficient**  **(M^-1^.cm^-1^)** | **Hydrophobic**  **(%)** | **Isoelectric point** | **Net charge**  **(pH 7)** | **Charge** | **Average hydrophobicity** | **Hydrophobicity**  **(pH 7)** | **Ratio hydrophilic residues**  **(%)** |  |
| B6-LGS | G-GHKAKGPRK-LGS | Adenovirus | - hCMECs |  | TfR1 | 1748,2 | 0 | 15,38 | 11,86 | 4,11 | 5 | 0,7 | 1,15 | 38 | [47] |
| C2-9r | CDIFTNSRGKRAGGGGrrrrrrrrr | Nucleic acids | - Mice |  | AchR | 3457,16 | 120 | 16 | 13,25 | 9,91 | 11 | 1,3 | 0,84 | 60 | [48] |
| RI-C2-9r | (botin)K-KGRSNTFIDC | Nucleic acids | - Mice |  | AchR | 1478,74 | 120 | 30 | 9,22 | 0,91 | 1 | 0,4 | 12 | 50 | [48] |
| CAGALCY | CAGALCY | NPs | - Mice |  |  | 1155,48 | 1520 | 85,71 | 7,98 | -1,18 | 0 | -1 | 49 | 0 | [49,50] |
| CDX | FKESWREARGTRIERG | NPs | - rat PBCECs  - Rats  - Mice | - Brain tumors  - Neurodegenerative disorders | nAChRs | 5690 | 25 | 12,03 | 1 | 2 | 1 | 10,62 | 56 | 56 | [51-53] |
| D-CDX | GreirtGraerwsekf | NPs | - BCECs | - Brain tumors | nAChRs | 5690 | 25 | 12,03 | 1 | 2 | 1 | 10,62 | 56 | 56 | [54] |
| CRT | CRTIGPSVC | NPs, small drugs | - bEnd.3s  - BCECs  - Mice | - Brain Tumors  - Neurodegenerative disorders | TfR | 1391,76 | 240 | 44,44 | 7,99 | 0,82 | 1 | -0,3 | 24,56 | 22 | [55-57] |
| dNP2 | KIKKVKKKGRK | CtCTLA-4 | - Mice | - Neurodegenerative disorders |  | 1796,45 | 0 | 18,18 | 12 | 8 | 8 | 1,9 | 0 | 73 | [58] |
| G23 | HLNILSTLWKYRC | NPs | - Mice |  | Ganglioside GM1 | 2102,67 | 7090 | 53,85 | 10,58 | 1,02 | 3 | -0,6 | 42 | 31 | [59] |
| GHo | NHQQQNPHQPPM | Nucleic acids, small drugs | - bEnd.3s | - Brain tumors |  | 1454,67 | 0 | 8,33 | 13,8 | 3 | 2 | -0,6 | -14,17 | 31 | [11,60] |
| GLA | GLAHSFSDFARDFVA |  | - hCMEC/D3 |  | RMT | 1638,86 | 0 | 53,33 | 8,02 | -1,89 | 0 | -0,2 | 30,93 | 33 | [61] |
| GSH | GSH | NPs | - Mice | - Brain tumors  - Neurodegenerative disorders  - Brain inflammation | GSH Transporter | 299,3 | 0 | 0 | 13,8 | 1 | 1 | -0,1 | 1 | 33 | [62-67] |
| GYR | GYRPVHNIRGHWAPG |  | - hCMEC/D3 |  | RMT | 1716,03 | 6970 | 33,33 | 12,5 | 1,22 | 4 | -0,3 | 15,4 | 20 | [61] |
| H102 | HKQLPFFEED | NPs | - Rats | - Neurodegenerative disorders |  | 1288,48 | 0 | 30 | 5,34 | -2,89 | -1 | 0,5 | 10,6 | 50 | [68] |
| HAV6 | SHAVSS | Small drugs | - Rats |  |  | 585,64 | 0 | 33,33 | 13,8 | 1,1 | 1 | -0,3 | 18,33 | 50 | [69] |
| Leptin [1-33] | METDTLLLWVLLLWVPGSTGDYPYDVPDYAGGSGIQKVQDDTKTLIKTIVTNINDISHTQSVSAK |  | - Rats |  | LRP | 7123,45 | 15220 | 40 | 4,5 | -4,89 | -3 | -0,2 | 23,37 | 34 | [70] |
| Leptin [12-32] | LLWVPGSTGDYPYDVPDYAGGSGIQKVQDDTKTLIKTIVTNINDISHTQSVSA |  | - Rats |  | LRP | 5679,6 | 9530 | 35,85 | 4,7 | -3,89 | -2 | -0,1 | 18,02 | 36 | [71] |
| Leptin [61-90} | YQQVLTSLPSQNVLQIANDLENLRDLLHLLC | NPs | - BCECs  - Mice  - Rats |  | LRP | 3564,33 | 1400 | 48,39 | 5,28 | -2,98 | -1 | -0,4 | 30,68 | 42 | [72,73] |
| M1 | TFYGGRPKRNNFLRGIR | Small drugs | - Mice | - Brain tumors | LRP | 2051,48 | 1280 | 29,41 | 12,97 | 4 | 5 | 0,2 | 15,41 | 41 | [74] |
| mHph2 | YARVRRRGPRRHHHHHHHHHHC | NPs | - Mice | - Brain tumors |  | 2916,39 | 1400 | 18,18 | 12,81 | 6,03 | 16 | 0,3 | 8,14 | 27 | [75] |
| AchR, Acetylcholine receptor; AMT, Adsorptive-mediated transport; BBB, Blood-brain barrier; BBBCM, cell-based BBB model; BBB-RTU, Blood-brain barrier ready-to-use device; BBCECs, Bovine brain capillary endothelial cells; BBMVECs, Bovine brain microvascular endothelial cells; BCECs, Brain capillary endothelial cells; bEnd.3, Brain endothelial cells; BMEC, Brain microvascular endothelial cells; HBEC-5i, Human brain endothelial cell line; hCMECs, Human cerebral microvascular endothelial cells; LDLR, Low density lipoprotein receptor; LRP, Lipoprotein receptor-related protein; MSC, Mesenchymal stem cells; NPs, Nanoparticles; NRP-1, Neuropilin-1; PBCECs, Porcine brain capillary endothelial cells; RBE4s, Rat brain endothelial cells; RMT, Receptor-mediated transport; TfR, Transferrin receptor; | | | | | | | | | | | | | | | |

**Table S2. Features of published BBB peptide shuttles (cont.).**

| **Peptide** | **Typical sequence** | **Main cargoes** | **BBB model** | **Pathology** | **Proposed translocation** | **Physicochemical properties** | | | | | | | | | **Ref.** |
| --- | --- | --- | --- | --- | --- | --- | --- | --- | --- | --- | --- | --- | --- | --- | --- |
|  |  |  |  |  |  | **Molecular weight**  **(g/mol)** | **Extinction coefficient**  **(M^-1^.cm^-1^)** | **Hydrophobic**  **(%)** | **Isoelectric point** | **Net charge**  **(pH 7)** | **Charge** | **Average hydrophobicity** | **Hydrophobicity**  **(pH 7)** | **Ratio hydrophilic residues**  **(%)** |  |
| MiniAp-4 | H-[Dap]KAPETALD-NH2 |  | - BBBCMs  - Mice |  |  | 842,98 | 0 | 37,5 | 7,04 | -2 | -1 | 0,7 | 5 | 38 | [76] |
| NGR | NGR | NPs | - BCECs  - Rats | - Brain tumors |  | 345,37 | 0 | 0 | 11,18 | 1 | 1 | 1,1 | -18,33 | 67 | [77] |
| NoLS | KKRTLRKNDRKKRC | NPs | - BCECs  - Mice | - Brain tumors | RMT | 1829,31 | 120 | 14,29 | 12,53 | 6,91 | 8 | 1,9 | -7,5 | 79 | [78] |
| Penetratin | RQIKIWFQNRRMKWKK | NPs | - bEnd.3s  - Mice | - Neurodegenerative disorders |  | 2246,85 | 11380 | 37,5 | 12,83 | 7 | 7 | 0,5 | 23 | 63 | [79] |
| D-penetratin | rqikiwfqnrrmkwkk | Small drugs | - Rats | - Brain tumors |  | 2246,85 | 11380 | 37,5 | 12,83 | 7 | 7 | 0,5 | 23 | 63 | [80] |
| PepC7 | CTSTSAPYC |  | - Mice |  | RMT | 931,07 | 1520 | 44,44 | 7,98 | 1,18 | 0 | -0,6 | 19,11 | 22 | [81] |
| PepH3 | AGILKRW |  | - bEnd.3s  - HBEC-5is  - Rats |  | AMT | 842,1 | 5690 | 57,14 | 11,6 | 2 | 2 | -0,2 | 42,86 | 29 | [82-84] |
| D-PepH3 | aGilkrw |  | - HBEC-5is |  | AMT | 842,1 | 5690 | 57,14 | 11,6 | 2 | 2 | -0,2 | 42,86 | 29 | [84] |
| PepNeg | SGTQEEY |  | - bEnd.3s |  | AMT | 811,84 | 1280 | 14,29 | 4,1 | -3 | -2 | 0,5 | -0,14 | 57 | [83] |
| Peptide 1* | GFtGFL-S | NPs | - Rats |  |  | 639,79 | 0 | 50 | 13,8 | -1 | 0 | -1,2 | 51,17 | 0 | [85] |
| Peptide 2* | GFtGFL-S[Glucose] | NPs | - Rats |  |  | 639,79 | 0 | 50 | 13,8 | -1 | 0 | -1,2 | 51,17 | 0 | [85] |
| Peptide 3* | GFtGFL-S[Galactose] | NPs | - Rats |  |  | 639,79 | 0 | 50 | 13,8 | -1 | 0 | -1,2 | 51,17 | 0 | [85] |
| Peptide 4* | GFtGFL-S[Xylose] | NPs | - Rats |  |  | 639,79 | 0 | 50 | 13,8 | -1 | 0 | -1,2 | 51,17 | 0 | [85] |
| Peptide 5* | GFtGFL-S[Lactose] | NPs | - Rats |  |  | 639,79 | 0 | 50 | 13,8 | -1 | 0 | -1,2 | 51,17 | 0 | [85] |
| Peptide-22 | [cMPRLRGC]c | NPs | - BCECs  - Mice  - Rats | - Brain tumors | LDLR | 934,24 | 240 | 50 | 11,05 | 0,82 | 2 | 0,1 | 24,75 | 25 | [86-88] |
| RGE | RGERPPR | NPs |  | - Brain tumors | NRP-1 | 866,02 | 0 | 0 | 12,81 | 1 | 2 | 1,7 | -23,57 | 57 | [89] |
| RVG | YTIWMPENPRPGTPCDIFTNSRGKRASNG | NPs, nucleic acids | - bEnd.3s + astrocytes  - BCECs  - hCMEC/D3  - Mice | - Brain tumors  - Brain inflammation  - Neurodegenerative disorders | AchR | 3265.81 | 7090 | 27,59 | 10,55 | 0,91 | 2 | 0,1 | 6,55 | 38 | [48,90-98] |
| RVG-9R | YTIWMPENPRPGTPCDIFTNSRGKRASNG-RRRRRRRRR | Nucleic acids | - Mice |  | AchR | 3265,81 | 7090 | 27,59 | 10,55 | 0,91 | 2 | 0,1 | 6,55 | 38 | [99] |
| RVGN7 | CDIFTNSRGKRASN-K(biotin) | Nucleic acids | - Mice |  | AchR | 1923,26 | 120 | 26,67 | 11,56 | 1,91 | 3 | 0,1 | 5,2 | 38 | [48] |
| SAP | VRLPPPVRLPPPVRLPPP |  | - bEnd.3s + MSCs |  |  | 1996,61 | 0 | 33,33 | 12,81 | 3 | 3 | 0 | 4 | 17 | [14] |
| SGV | SGVYKVAYDWQH |  | - BCECs + pericytes |  |  | 1451,67 | 8250 | 50 | 9,52 | -0,89 | 1 | -0,5 | 27,58 | 33 | [100] |
| AchR, Acetylcholine receptor; AMT, Adsorptive-mediated transport; BBB, Blood-brain barrier; BBBCM, cell-based BBB model; BBB-RTU, Blood-brain barrier ready-to-use device; BBCECs, Bovine brain capillary endothelial cells; BBMVECs, Bovine brain microvascular endothelial cells; BCECs, Brain capillary endothelial cells; bEnd.3, Brain endothelial cells; BMEC, Brain microvascular endothelial cells; HBEC-5i, Human brain endothelial cell line; hCMECs, Human cerebral microvascular endothelial cells; LDLR, Low density lipoprotein receptor; LRP, Lipoprotein receptor-related protein; MSC, Mesenchymal stem cells; NPs, Nanoparticles; NRP-1, Neuropilin-1; PBCECs, Porcine brain capillary endothelial cells; RBE4s, Rat brain endothelial cells; RMT, Receptor-mediated transport; TfR, Transferrin receptor; | | | | | | | | | | | | | | | |

**Table S2. Features of published BBB peptide shuttles (cont.).**

| **Peptide** | **Typical sequence** | **Main cargoes** | **BBB model** | **Pathology** | **Proposed translocation** | **Physicochemical properties** | | | | | | | | | **Ref.** |
| --- | --- | --- | --- | --- | --- | --- | --- | --- | --- | --- | --- | --- | --- | --- | --- |
|  |  |  |  |  |  | **Molecular weight**  **(g/mol)** | **Extinction coefficient**  **(M^-1^.cm^-1^)** | **Hydrophobic**  **(%)** | **Isoelectric point** | **Net charge**  **(pH 7)** | **Charge** | **Average hydrophobicity** | **Hydrophobicity**  **(pH 7)** | **Ratio hydrophilic residues**  **(%)** |  |
| SxTSSTx | SYTSSTM |  | - Rats |  | RMT | 775,85 | 1280 | 28,57 | 6 | 0 | 0 | -0,5 | 21,14 | 43 | [101] |
| SynB1 | RGGRLSYSRRRFSTSTGR | Small drugs | - Rats | - Brain tumors |  | 2099,42 | 1280 | 16,67 | 13,33 | 5 | 6 | 0,7 | 10,11 | 56 | [80] |
| T7 | HAIYPRH | NPs | - bEnd.3s + MSC  - BCECs + pericytes | - Brain tumors | TfR | 892,07 | 1280 | 42,86 | 11,41 | 0,22 | 3 | -0,4 | 22,71 | 14 | [14,102-106] |
| TAT | YGRKKRRQRRR | Proteins, NPs, small drugs | - bEnd.3s  - BCECs  -hCMEC/D3  - hCMECs  - PBCECs  - Mice  - Rats  - Rabbits | - Brain tumors  - Neurodegenerative disorders  - Brain inflammation  - Brain infection  - Lysosomal storage disorders | AMT | 1396,71 | 0 | 0 | 13,33 | 8 | 8 | 2,4 | -14 | 90 | [10,107-122] |
| RI-OR2-TAT | rGffvlkGrrrrqrrkkrGy | NPs | - hCMEC/D3  - Mice | - Neurodegenerative disorders | AMT | 2662,3 | 1280 | 25 | 13,54 | 11 | 11 | 1,1 | 12,1 | 60 | [123] |
| TfRL1 | THRPPMWSPVWP |  | - bEnd.3s + MSC |  | TfR | 1490,8 | 11380 | 33,33 | 11,18 | 1,11 | 2 | -0,6 | 13,5 | 17 | [14] |
| TGN | TGNYKALHPHNG | NPs | - bEnd.3s  - Mice | - Brain tumors  - Neurodegenerative disorders | RMT | 1307,51 | 1280 | 25 | 10,59 | 0,22 | 3 | -0,2 | 6,83 | 25 | [124-130] |
| THR | THRPPMWSPVWP | NPs | - bEnd.3s  - BMBECs + astrocytes  - hCMEC/D3  - Mice  - Rats | - Neurodegenerative disorders | TfR | 1490,8 | 11380 | 33,33 | 11,18 | 1,11 | 2 | -0,6 | 13,5 | 17 | [106,131-135] |
| THR enan | Thrppmwspvwp |  | - BMBECs + astrocytes |  | TfR | 1490,8 | 11380 | 33,33 | 11,18 | 1,11 | 2 | -0,6 | 13,5 | 17 | [132] |
| THR retro enan | Pwvpswmpprht |  | - BMBECs + astrocytes |  | TfR | 1490,8 | 11380 | 33,33 | 11,18 | 1,11 | 2 | -0,6 | 13,5 | 17 | [132] |
| THR_met | T(NMe)H(NMe)RPPM(NMe)WSPVWP |  | - BMBECs + astrocytes |  | TfR | 1490,8 | 11380 | 33,33 | 11,18 | 1,11 | 2 | -0,6 | 13,5 | 17 | [132] |
| THRre_2c | (pwvpswmpprht-o2oC)_2_KKKC |  | - MBECs |  | TfR | 3450,29 | 22880 | 32,14 | 12,54 | 4,13 | 7 | -0,2 | 10,86 | 25 | [136] |
| THRre_2f | (pwvpswmpprht)_2_KKGK(CF)G |  | - MBECs |  | TfR | 3794,55 | 22760 | 27,59 | 12,54 | 5,22 | 7 | -0,2 | 8,79 | 24 | [136] |
| THRre_2m | (pwvpswmpprht)_2_KKGK(mAL)G |  | - MBECs |  | TfR | 3794,55 | 22760 | 27,59 | 12,54 | 5,22 | 7 | -0,2 | 8,79 | 24 | [136] |
| TPL | TGNYKALHPHNG-GGGG-HLNILSTLWKYR-C | NPs | - bEnd.3s  - Rats | - Neurodegenerative disorders |  | 3164,79 | 8370 | 34,48 | 10,48 | 2,24 | 6 | -0,4 | 21,66 | 24 | [137] |
| THR retro enan | Pwvpswmpprht |  | - BMBECs + astrocytes |  | TfR | 1490,8 | 11380 | 33,33 | 11,18 | 1,11 | 2 | -0,6 | 13,5 | 17 | [132] |
| AchR, Acetylcholine receptor; AMT, Adsorptive-mediated transport; BBB, Blood-brain barrier; BBBCM, cell-based BBB model; BBB-RTU, Blood-brain barrier ready-to-use device; BBCECs, Bovine brain capillary endothelial cells; BBMVECs, Bovine brain microvascular endothelial cells; BCECs, Brain capillary endothelial cells; bEnd.3, Brain endothelial cells; BMEC, Brain microvascular endothelial cells; HBEC-5i, Human brain endothelial cell line; hCMECs, Human cerebral microvascular endothelial cells; LDLR, Low density lipoprotein receptor; LRP, Lipoprotein receptor-related protein; MSC, Mesenchymal stem cells; NPs, Nanoparticles; NRP-1, Neuropilin-1; PBCECs, Porcine brain capillary endothelial cells; RBE4s, Rat brain endothelial cells; RMT, Receptor-mediated transport; TfR, Transferrin receptor; | | | | | | | | | | | | | | | |

**Table S3. Features of published cell-penetrating peptides.**

| **Peptide** | **Typical sequence** | **Main cargoes** | **Cell model** | **Diseases** | **Proposed translocation** | **Physicochemical properties** | | | | | | | | | **Ref.** |
| --- | --- | --- | --- | --- | --- | --- | --- | --- | --- | --- | --- | --- | --- | --- | --- |
|  |  |  |  |  |  | **Molecular weight**  **(g/mol)** | **Extinction coefficient**  **(M^-1^.cm^-1^)** | **Hydrophobic**  **(%)** | **Isoelectric point** | **Net charge**  **(pH 7)** | **Charge** | **Average hydrophobicity** | **Hydrophobicity**  **(pH 7)** | **Ratio hydrophilic residues**  **(%)** |  |
| - | CWWRRRRRRRRCRRRRRRRRCRRRRRRRRC | Proteins | - DC2.4 - Mice |  |  | 4550,59 | 11860 | 20 | 13,33 | 22,64 | 24 | 2 | 1,8 | 80 | [138] |
| - | KYKGAIIGNIK | Nucleic acids | - HEK293T - Bacteria | Infectious Diseases |  | 1203,57 | 1280 | 45,45 | 10,98 | 2 | 3 | 0,1 | 26,45 | 36 | [139] |
| - | KYRSGAITIGY | Nucleic acids | - HEK293T - Bacteria | Infectious Diseases |  | 1227,49 | 2560 | 45,45 | 10,74 | 1 | 2 | -0,3 | 30,55 | 27 | [139] |
| - | EPRNEEK | NPs | - BCECs - Mice | Cancer |  | 899,99 | 0 | 0 | 7,14 | -2 | -1 | 2,2 | -31 | 86 | [140] |
| (F4S-Y13S)Cti | KWCSRVCYRGICSRRCRG | Proteins | - HeLa, MCF-7, MDA-MB-435S, HaCaT, MM96L, HT144, WM164 - Lipid vesicles | Cancer |  | 2189,69 | 7450 | 44,44 | 10,54 | 6,63 | 7 | 0,3 | 23,78 | 44 | [141] |
| (Y8S-I11S)Cti | KWCFRVCSRGSCYRRCRG | Proteins | - HeLa, MCF-7, MDA-MB-435S, HaCaT, MM96L, HT144, WM164 - Lipid vesicles | Cancer |  | 2223,7 | 7450 | 44,44 | 10,54 | 5,63 | 6 | 0,3 | 23,67 | 44 | [141] |
| AA3H | MASIWVGHRG | Proteins | - HeLa, RAW264.7 - Mice |  | Endocytosis | 1113,36 | 5690 | 50 | 11,18 | 2,11 | 3 | -0,6 | 37,6 | 20 | [142] |
| ACCP | CGehGehGehGehGGrrrrrrrrGC | NPs | - HT-1080, bEnd.3, MCF-7, A549  - Mice | Cancer | Macropinocytosis | 2938,31 | 240 | 8 | 11,46 | 5,27 | 9 | 1,3 | -4,24 | 48 | [143,144] |
| Acpp  (AVB-620) | EEEEEEEEPLGLAGRRRRRRRR | Proteins, NPs | - BMEC - Mice  - Humans (Phase I) | Cancer |  | 2965,34 | 0 | 13,04 | 10,69 | 2,01 | 2 | 2 | -7,78 | 74 | [27,145,146] |
| D-Acpp | eeeeeeeePLGLAGrrrrrrrrr |  | - HT1080, MDA-MB-231 - Mice | Cancer |  | 2965,34 | 0 | 13,04 | 10,69 | 2,01 | 2 | 2 | -7,78 | 74 | [147,148] |
| AIP6 | RLRWR |  | - RAW 264.7 - Mice | Inflammatory disorders |  | 785,97 | 5690 | 40 | 12,81 | 4 | 4 | 0,8 | 31 | 60 | [149] |
| AM-111 (Brimapitide) | DQSRPVQPFLNLTTPRKPRPPRRRQRRKKRG |  | - Humans (Phase II) | Cutaneous disorders |  | 3823,62 | 0 | 12,9 | 13,06 | 12 | 12 | 1 | -6,55 | 58 | [150] |
| Antp (2) | (BMB)RRRRFFCSASASCK | Proteins | - HeLa | Cancer | Endocytosis | 1598,89 | 240 | 42,86 | 12,03 | 5,82 | 6 | 0,7 | 16 | 57 | [151] |
| Antp (4) | (BMB)RRRRFFCALDWSWLQC | Proteins | - HeLa | Cancer | Endocytosis | 2067,48 | 11620 | 56,25 | 10,92 | 3,82 | 4 | 0 | 34,06 | 44 | [151] |
| Antp (5) | (BMB)RRRRFFCALDASALQC | Proteins | - HeLa | Cancer | Endocytosis | 1837,2 | 240 | 56,25 | 10,92 | 3,82 | 4 | 0,3 | 27,06 | 44 | [151] |
| Antp-HD | HALCLTERQIKIWFQNRRMKWKKEN | Nucleic acids, proteins | - Rat pituitary cells - Rats |  | Inverted micelles | 3258,02 | 11500 | 40 | 11,01 | 6,02 | 7 | 0,3 | 23,04 | 52 | [152] |
| Antp-HD 40P2 | HALCPPERQIKIWFQNRRMKWKKEN | Nucleic acids, proteins | - Rat pituitary cells - Rats |  | Inverted micelles | 3237,98 | 11500 | 36 | 11,01 | 6,02 | 7 | 0,4 | 14,84 | 52 | [152] |
| Antp-HD 50A | AYALCLTERQIKIWFANRRMKWKKEN | Nucleic acids, proteins | - Rat pituitary cells - Rats |  | Inverted micelles | 3298,07 | 12780 | 50 | 10,71 | 5,91 | 6 | 0,2 | 27,81 | 46 | [152] |
| Aca, Aminocaproic acid; AEC, Aortic endothelial cells; Ahx, 6-aminohexanoic acid; Aib, 2-aminoisobutyric acid; B-ALL, B acute lymphoblastic leukemia; BBMVEC, Bovine brain microvascular endothelial cells; BCECs, Brain capillary endothelial cells; BHK, Baby hamster kidney; BMB, 3,5-bis(mercaptomethyl)benzoyl; BMDCs, Bone marrow-derived dendritic cells; BMEC, Brain microvascular endothelial cells; Cha, Cyclohexylalanine; CHO, Chinese hamster ovary; Dap, Diaminopimelic acid; DC2.4, Mouse dendritic cells; DMECs, Dermal microvessel endothelial cells; Dmt, *N,N*-Dimethyltryptamine; ES, Embryonic stem cells; F2, Diphenyl; GICs, Glioma-initiating cells; HASMCs, primary human aortic smooth muscle cells; HCE, Human corneal epithelial; HCEC, Human corneal epithelial cells; HCM, Human cardiac myocytes; HEK, Human embryonic kidney; HEL, Human erythroleukemia cell; Hex, Hexyl; hfRPE, Human fetal retinal pigment epithelial cells; hMSCs, Human mesenchymal stem cells; HMVEC, Human lung microvascular endothelial cells; HUVECs, Human umbilical vein endothelial cells; MDCK, Madin-Darby canine kidney; Met, Methylation; Nap, Naphthylalanine; NHC, Human conjunctival epithelial cells; NHDF, Normal human dermal fibroblasts; NPs, Nanoparticles; RAECM, Rat alveolar epithelial cell monolayers; SDHCEC, Spontaneously derived human corneal epithelial cells; Sip, Silaproline; X, Cyclohexylalanine; , Naphthylalanone; - No name attributed; * Different peptides with similar designations; | | | | | | | | | | | | | | | |

**Table S3. Features of published cell-penetrating peptides (cont.).**

| **Peptide** | **Typical sequence** | **Main cargoes** | **Cell model** | **Diseases** | **Proposed translocation** | **Physicochemical properties** | | | | | | | | | **Ref.** |
| --- | --- | --- | --- | --- | --- | --- | --- | --- | --- | --- | --- | --- | --- | --- | --- |
|  |  |  |  |  |  | **Molecular weight**  **(g/mol)** | **Extinction coefficient**  **(M^-1^.cm^-1^)** | **Hydrophobic**  **(%)** | **Isoelectric point** | **Net charge**  **(pH 7)** | **Charge** | **Average hydrophobicity** | **Hydrophobicity**  **(pH 7)** | **Ratio hydrophilic residues**  **(%)** |  |
| aPP5R1 | GPSQPTYPGDDAPVRDLIRFYRDLRRYLNVVTRHRY | Proteins | - HeLa |  | Endocytosis | 4364,08 | 5120 | 36,11 | 10,27 | 4,11 | 5 | 0,2 | 13,69 | 39 | [153] |
| aPP6R1 | GPSQPTYPGDDAPVRDLRRFYRDLRRYLNVVTRHRY | Proteins | - HeLa |  | Endocytosis | 4407,1 | 5120 | 33,33 | 10,69 | 5,11 | 6 | 0,2 | 10,56 | 39 | [153] |
| ARF (1-22) | MVRRFLVTLRIRRACGPPRVRV | Nucleic acids | - MCF-7, MDA-MB-231, A2780, 4T1 - Mice | Cancer | Endocytosis | 2652,42 | 120 | 50 | 12,9 | 7,91 | 8 | 0,2 | 31,23 | 32 | [154] |
| ARF (19–31) | RVRVFVVHIPRLT | Nucleic acids | - MCF-7, MDA-MB-231, A2780, 4T1 - Mice | Cancer |  | 1592,04 | 0 | 53,85 | 12,81 | 4,11 | 5 | -0,3 | 41 | 23 | [154] |
| Aurein 2.2 | GLFDIVKKVVGAL |  | - Bacteria - Lipid vesicles | Infectious Diseases |  | 1358,77 | 0 | 61,54 | 10,02 | 2 | 2 | -0,3 | 43,38 | 23 | [155] |
| AVN | KCPSRRPKR | Proteins | - EBTr |  |  | 1127,4 | 120 | 11,11 | 12,21 | 5,91 | 6 | 1,6 | -15,11 | 67 | [156] |
| AZX100 | YARAAARQARAWLRRASAPLPGLK |  | - Fibroblasts - Hamsters | Cutaneous disorders |  | 2651,21 | 6970 | 54,17 | 12,69 | 7 | 7 | 0,1 | 24,5 | 33 | [157] |
| BA27 | GRFKRFRKKFKKLFKKLSKKWKMRRNQFWVKVQRG | Proteins | - L02, HUVEC, ECV304, HeLa, MDA-MB-435, MDA-MB-231, HCM, MCF-7, HK-2, A375 | Cancer |  | 4600,88 | 11380 | 34,29 | 13,35 | 18 | 18 | 0,7 | 20,06 | 60 | [158] |
| BA28 | GGLRSLGRKILRAWKKYGGKKWKMRRNQFWVKVQRG | Proteins | - L02, HUVEC, ECV304, HeLa, MDA-MB-435, MDA-MB-231, HCM, MCF-7, HK-2, A375 | Cancer |  | 4374,5 | 18350 | 36,11 | 12,83 | 14 | 14 | 0,4 | 22,39 | 47 | [158] |
| Bac | RRIRPRPPRLPRPRPRPLPFPRP | Proteins | - Pan-1, MiaPaca-2, S2013 | Cancer |  | 2881,61 | 0 | 17,39 | 13,6 | 10 | 10 | 0,8 | -8,26 | 39 | [159] |
| Bac 7 | RRIRPRPPRLPRPRPRPLPFPRPGPRPIPRPLPFPRPGPRPIPRPLPFPRPGPRPIPRP |  | - RAW 264.7 - Bacteria | Infectious Diseases |  | 6910,68 | 0 | 18,64 | 13,86 | 18 | 18 | 0,5 | -7,44 | 29 | [159] |
| Bac 7 (1-7) | RRIRPRP |  | - RAW 264.7 - Bacteria | Infectious Diseases |  | 950,18 | 0 | 14,29 | 12,97 | 5 | 5 | 1,5 | -7 | 57 | [159] |
| Bac-7 (1-15) | RRIRPRPPRLPRPRP |  | - RAW 264.7 - Bacteria | Infectious Diseases |  | 1920,4 | 0 | 13,33 | 13,45 | 8 | 8 | 1,2 | -11,67 | 47 | [159] |
| Bac 7 (1-17) | RRIRPRPPRLPRPRPRP |  | - RAW 264.7 - Bacteria | Infectious Diseases |  | 2173,71 | 0 | 11,76 | 13,53 | 9 | 9 | 1,2 | -13,82 | 47 | [159] |
| Bac-7 (1-24) | RRIRPRPPRLPRPRPRPLPFPRPGPRPIPRPLPFPRPGPRPIPRPLPFPRPGPRPIPRP |  | - RAW 264.7 - Bacteria | Infectious Diseases |  | 6910,68 | 0 | 18,64 | 13,86 | 18 | 18 | 0,5 | -7,44 | 29 | [159] |
| Bac 7 (7-24) | PPRLPRPRPRPLPFPRPG |  | - RAW 264.7 - Bacteria | Infectious Diseases |  | 2103,62 | 0 | 16,67 | 13,16 | 6 | 6 | 0,5 | -10,39 | 28 | [159] |
| Bac 7 ( 9-24) | RLPRPRPRPLPFPRPG |  | - RAW 264.7 - Bacteria | Infectious Diseases |  | 1909,38 | 0 | 18,75 | 13,16 | 6 | 6 | 0,6 | -5,94 | 31 | [159] |
| Bac 7 (11-24) | PRPRPRPLPFPRPG |  | - RAW 264.7 - Bacteria | Infectious Diseases |  | 1640,02 | 0 | 14,29 | 12,97 | 5 | 5 | 0,6 | -12,93 | 29 | [159] |
| Aca, Aminocaproic acid; AEC, Aortic endothelial cells; Ahx, 6-aminohexanoic acid; Aib, 2-aminoisobutyric acid; B-ALL, B acute lymphoblastic leukemia; BBMVEC, Bovine brain microvascular endothelial cells; BCECs, Brain capillary endothelial cells; BHK, Baby hamster kidney; BMB, 3,5-bis(mercaptomethyl)benzoyl; BMDCs, Bone marrow-derived dendritic cells; BMEC, Brain microvascular endothelial cells; Cha, Cyclohexylalanine; CHO, Chinese hamster ovary; Dap, Diaminopimelic acid; DC2.4, Mouse dendritic cells; DMECs, Dermal microvessel endothelial cells; Dmt, *N,N*-Dimethyltryptamine; ES, Embryonic stem cells; F2, Diphenyl; GICs, Glioma-initiating cells; HASMCs, primary human aortic smooth muscle cells; HCE, Human corneal epithelial; HCEC, Human corneal epithelial cells; HCM, Human cardiac myocytes; HEK, Human embryonic kidney; HEL, Human erythroleukemia cell; Hex, Hexyl; hfRPE, Human fetal retinal pigment epithelial cells; hMSCs, Human mesenchymal stem cells; HMVEC, Human lung microvascular endothelial cells; HUVECs, Human umbilical vein endothelial cells; MDCK, Madin-Darby canine kidney; Met, Methylation; Nap, Naphthylalanine; NHC, Human conjunctival epithelial cells; NHDF, Normal human dermal fibroblasts; NPs, Nanoparticles; RAECM, Rat alveolar epithelial cell monolayers; SDHCEC, Spontaneously derived human corneal epithelial cells; Sip, Silaproline; X, Cyclohexylalanine; , Naphthylalanone; - No name attributed; * Different peptides with similar designations; | | | | | | | | | | | | | | | |

**Table S3. Features of published cell-penetrating peptides (cont.).**

| **Peptide** | **Typical sequence** | **Main cargoes** | **Cell model** | **Diseases** | **Proposed translocation** | **Physicochemical properties** | | | | | | | | | **Ref.** |
| --- | --- | --- | --- | --- | --- | --- | --- | --- | --- | --- | --- | --- | --- | --- | --- |
|  |  |  |  |  |  | **Molecular weight**  **(g/mol)** | **Extinction coefficient**  **(M^-1^.cm^-1^)** | **Hydrophobic**  **(%)** | **Isoelectric point** | **Net charge**  **(pH 7)** | **Charge** | **Average hydrophobicity** | **Hydrophobicity**  **(pH 7)** | **Ratio hydrophilic residues**  **(%)** |  |
| Bac 7 (13-24) | PRPRPLPFPRPG |  | - RAW 264.7 - Bacteria | Infectious Diseases |  | 1386,71 | 0 | 16,67 | 12,81 | 4 | 4 | 0,4 | -10,08 | 25 | [159] |
| Bac 7 (15-24) | PRPLPFPRPG |  | - RAW 264.7 - Bacteria | Infectious Diseases |  | 1133,4 | 0 | 20 | 12,49 | 3 | 3 | 0,2 | -6,1 | 20 | [159] |
| BIM BH3 | IWIAQELRRIGDEFNAYYARR |  | - B-ALL, Jurkat T - Lipid vesicles |  |  | 2641,09 | 8250 | 52,38 | 9,62 | 2 | 2 | 0,1 | 29,33 | 43 | [160] |
| BIM BH3 (4) | IWIAQELARIGDEFNAYYARR |  | - B-ALL, Jurkat T - Lipid vesicles |  |  | 2555,98 | 8250 | 57,14 | 7,02 | 1 | 1 | -0,1 | 31,95 | 38 | [160] |
| BIM BH3 (5) | IWIAQELRAIGDEFNAYYARR |  | - B-ALL, Jurkat T - Lipid vesicles |  |  | 2555,98 | 8250 | 57,14 | 7,02 | 1 | 1 | -0,1 | 31,95 | 38 | [160] |
| BIM BH3 (9) | IWIAQELRAIGDAFNAYYARR |  | - B-ALL, Jurkat T - Lipid vesicles |  |  | 2497,94 | 8250 | 61,9 | 9,63 | 2 | 2 | -0,2 | 35,38 | 33 | [160] |
| BIM BH3 (15) | IWIAQELRRIGDEFAAYYARR |  | - B-ALL, Jurkat T - Lipid vesicles |  |  | 2598,06 | 8250 | 57,14 | 9,62 | 2 | 2 | 0,1 | 33,24 | 38 | [160] |
| BIM SAHBA1 | IWIAQELRRIGDEFNAYYARR |  | - B-ALL, Jurkat T - Lipid vesicles |  |  | 2641,09 | 8250 | 52,38 | 9,62 | 2 | 2 | 0,1 | 29,33 | 43 | [160] |
| BIM SAHBA1 (1) | IWIAQELRAIGDAFNAYYARR |  | - B-ALL, Jurkat T - Lipid vesicles |  |  | 2497,94 | 8250 | 61,9 | 9,63 | 2 | 2 | -0,2 | 35,38 | 33 | [160] |
| BIM SAHBA1 (13) | IWIAQELDAIGDAFNAYYARR |  | - B-ALL, Jurkat T - Lipid vesicles |  |  | 2456,84 | 8250 | 61,9 | 4,35 | 0 | 0 | -0,2 | 33,43 | 33 | [160] |
| BIM SAHBA1 (16) | IRIAQELRAIGDAFNAYYARR |  | - B-ALL, Jurkat T - Lipid vesicles |  |  | 2467,91 | 2560 | 57,14 | 10,4 | 3 | 3 | 0,1 | 30,1 | 38 | [160] |
| BIM SAHBA1 (17) | IWIAQELRAIGDAFNEYYARR |  | - B-ALL, Jurkat T - Lipid vesicles |  |  | 2555,98 | 8250 | 57,14 | 7,02 | 1 | 1 | -0,1 | 31,95 | 38 | [160] |
| BIM SAHBA1 (18) | IWIAQELRAIGDAFNATYARR |  | - B-ALL, Jurkat T - Lipid vesicles |  |  | 2435,87 | 6970 | 57,14 | 10,01 | 2 | 2 | -0,1 | 33 | 33 | [160] |
| BIM SAHBA1 (19) | IWIAQELRAIEDAFNAYYARR |  | - B-ALL, Jurkat T - Lipid vesicles |  |  | 2570 | 8250 | 61,9 | 7,02 | 1 | 1 | -0,1 | 33,9 | 38 | [160] |
| BMV Gag | KMTRAQRRAAARRNRWTAR | NPs | - CHO-K1, HeLa, Jurkat T, Caco-2  - Lipid vesicles |  | Macropinocytosis | 2356,81 | 5690 | 36,84 | 13,45 | 9 | 9 | 0,9 | 12,11 | 53 | [161-163] |
| BP16 | KKLFKKILKKL | Proteins, small drugs | - MCF-7, CAPAN-1, NIH3T3 | Cancer |  | 1386,95 | 0 | 45,45 | 11,44 | 7 | 7 | 0,8 | 32,55 | 55 | [164] |
| BP100 | KKLFKKILKYL | Nucleic acids | - Plants |  |  | 1421,95 | 1280 | 54,55 | 10,85 | 6 | 6 | 0,3 | 40,36 | 45 | [165] |
| BP100-CH7 | KKLFKKILKYLHHCRGHTVHSHHHCIR | NPs | - Plants |  |  | 3358,22 | 1520 | 37,04 | 10,83 | 8,6 | 15 | 0 | 27,089 | 30 | [166] |
| BP100-KH9 | KKLFKKILKYLKHKHKHKHKHKHKHKHKH | NPs, Nucleic acids | - Plants |  |  | 3809,92 | 1280 | 20,69 | 11,54 | 16 | 24 | 0,9 | 10,66 | 48 | [165,166] |
| Aca, Aminocaproic acid; AEC, Aortic endothelial cells; Ahx, 6-aminohexanoic acid; Aib, 2-aminoisobutyric acid; B-ALL, B acute lymphoblastic leukemia; BBMVEC, Bovine brain microvascular endothelial cells; BCECs, Brain capillary endothelial cells; BHK, Baby hamster kidney; BMB, 3,5-bis(mercaptomethyl)benzoyl; BMDCs, Bone marrow-derived dendritic cells; BMEC, Brain microvascular endothelial cells; Cha, Cyclohexylalanine; CHO, Chinese hamster ovary; Dap, Diaminopimelic acid; DC2.4, Mouse dendritic cells; DMECs, Dermal microvessel endothelial cells; Dmt, *N,N*-Dimethyltryptamine; ES, Embryonic stem cells; F2, Diphenyl; GICs, Glioma-initiating cells; HASMCs, primary human aortic smooth muscle cells; HCE, Human corneal epithelial; HCEC, Human corneal epithelial cells; HCM, Human cardiac myocytes; HEK, Human embryonic kidney; HEL, Human erythroleukemia cell; Hex, Hexyl; hfRPE, Human fetal retinal pigment epithelial cells; hMSCs, Human mesenchymal stem cells; HMVEC, Human lung microvascular endothelial cells; HUVECs, Human umbilical vein endothelial cells; MDCK, Madin-Darby canine kidney; Met, Methylation; Nap, Naphthylalanine; NHC, Human conjunctival epithelial cells; NHDF, Normal human dermal fibroblasts; NPs, Nanoparticles; RAECM, Rat alveolar epithelial cell monolayers; SDHCEC, Spontaneously derived human corneal epithelial cells; Sip, Silaproline; X, Cyclohexylalanine; , Naphthylalanone; - No name attributed; * Different peptides with similar designations; | | | | | | | | | | | | | | | |

**Table S3. Features of published cell-penetrating peptides (cont.).**

| **Peptide** | **Typical sequence** | **Main cargoes** | **Cell model** | **Diseases** | **Proposed translocation** | **Physicochemical properties** | | | | | | | | | **Ref.** |
| --- | --- | --- | --- | --- | --- | --- | --- | --- | --- | --- | --- | --- | --- | --- | --- |
|  |  |  |  |  |  | **Molecular weight**  **(g/mol)** | **Extinction coefficient**  **(M^-1^.cm^-1^)** | **Hydrophobic**  **(%)** | **Isoelectric point** | **Net charge**  **(pH 7)** | **Charge** | **Average hydrophobicity** | **Hydrophobicity**  **(pH 7)** | **Ratio hydrophilic residues**  **(%)** |  |
| KH9-BP100 | KHKHKHKHKHKHKHKHKHKKLFKKILKYL | Nucleic acids | - Plants |  |  | 3809,92 | 1280 | 20,69 | 11,54 | 16 | 24 | 0,9 | 10,66 | 48 | [165] |
| bPrPp | MVKSKIGSWILVLFVAMWSDVGLCKKRPKP | Proteins, Nucleic acid | - CHO, HeLa, HepG2 |  | Endocytosis, Macropinocytosis | 3418,44 | 11500 | 53,33 | 10,78 | 5,91 | 6 | -0,2 | 34,67 | 33 | [167,168] |
| BR2 | RAGLPFQVGRLLRRLLR | Proteins, Nucleic acids | - HCT116, HeLa, B16/F10, NIH3T3, HaCat, BJ | Cancer | Lipid-mediated macropinocytosis | 2021,59 | 0 | 47,06 | 13,16 | 6 | 6 | 0,1 | 34,59 | 35 | [169,170] |
| Buforin 2 | TRSSRAGLQWPVGRVHRLLRK |  | - Bacteria  - Lipid vesicles | Infectious Diseases |  | 2474,03 | 5690 | 33,33 | 13,17 | 7,11 | 8 | 0,3 | 21,52 | 43 | [171] |
| C105Y | CSIPPEVKFNKPFVYLI | Proteins | - RBL-2H3, HuH7, Swiss 3T3, Bovine spermatozoa, Human semen | Reproductive disorders | Endocytosis-independent | 1994,51 | 1400 | 52,94 | 8,77 | 1,91 | 2 | -0,4 | 29,12 | 29 | [172-174] |
| cAFFR4 | (AFΦRRRRQ)c | Proteins | - MCF-7, A549 | Cancer |  | 1136,35 | 0 | 37,5 | 12,97 | 5 | 5 | 0,8 | 21,12 | 63 | [175] |
| cA2FFR4 | (AAFΦRRRRQ)c | Proteins | - MCF-7, A549 | Cancer |  | 1060,25 | 0 | 37,5 | 12,97 | 5 | 5 | 1,1 | 14,12 | 63 | [175] |
| cA3FFR4 | (AAAFΦRRRRQ)c | Proteins | - MCF-7, A549 | Cancer |  | 1131,33 | 0 | 44,44 | 12,97 | 5 | 5 | 0,9 | 17,11 | 56 | [175] |
| cA4FFR4 | (AAAAFΦRRRRQ)c | Proteins | - MCF-7, A549 | Cancer |  | 1202,41 | 0 | 50 | 12,97 | 5 | 5 | 0,8 | 19,5 | 50 | [175] |
| cA5FFR4 | (AAAAAFΦRRRRQ)c | Proteins | - MCF-7, A549 | Cancer |  | 1273,48 | 0 | 54,55 | 12,97 | 5 | 5 | 0,7 | 21,45 | 45 | [175] |
| cA6FFR4 | (AAAAAAFΦRRRRQ)c | Proteins | - MCF-7, A549 | Cancer |  | 1344,57 | 0 | 58,33 | 12,97 | 5 | 0,6 | 0,6 | 23,08 | 42 | [175] |
| cF2R4 | (FFRRRRQ)c | Proteins | - MCF-7, A549 | Cancer |  | 1065,27 | 0 | 28,57 | 12,97 | 5 | 5 | 1 | 18,29 | 71 | [175] |
| cFΦR4  (CPP1) | (FΦRRRRQ)c | Proteins | - HeLa, MCF-7, A549 |  |  | 918,09 | 0 | 16,67 | 12,97 | 5 | 5 | 1,6 | 5,17 | 83 | [175-178] |
| CPP2 | (RRΦFRRQ)c | Proteins | - HeLa, NIH3T3, A549, H1299 - Lipid vesicles |  | Endocytosis | 1065,27 | 0 | 28,57 | 12,97 | 5 | 5 | 1 | 18,29 | 71 | [177] |
| CPP3 | (RRFRΦRQ)c | Proteins | - HeLa, NIH3T3, A549, H1299 - Lipid vesicles |  | Endocytosis | 1065,27 | 0 | 28,57 | 12,97 | 5 | 5 | 1 | 19,29 | 71 | [177] |
| CPP4 | (FRRRRΦQ)c | Proteins | - HeLa, NIH3T3, A549, H1299 - Lipid vesicles |  | Endocytosis | 1065,27 | 0 | 28,57 | 12,97 | 5 | 5 | 1 | 18,29 | 71 | [177] |
| CPP5 | (FΦRRRQ)c | Proteins | - HeLa, NIH3T3, A549, H1299 - Lipid vesicles |  | Endocytosis | 909,08 | 0 | 33,33 | 12,81 | 4 | 4 | 0,7 | 23,67 | 67 | [177] |
| CPP6 | (FΦRRRRRQ)c | Proteins | - HeLa, NIH3T3, A549, H1299 - Lipid vesicles |  | Endocytosis | 1221,46 | 0 | 25 | 13,16 | 6 | 6 | 1,3 | 14,25 | 75 | [177] |
| Aca, Aminocaproic acid; AEC, Aortic endothelial cells; Ahx, 6-aminohexanoic acid; Aib, 2-aminoisobutyric acid; B-ALL, B acute lymphoblastic leukemia; BBMVEC, Bovine brain microvascular endothelial cells; BCECs, Brain capillary endothelial cells; BHK, Baby hamster kidney; BMB, 3,5-bis(mercaptomethyl)benzoyl; BMDCs, Bone marrow-derived dendritic cells; BMEC, Brain microvascular endothelial cells; Cha, Cyclohexylalanine; CHO, Chinese hamster ovary; Dap, Diaminopimelic acid; DC2.4, Mouse dendritic cells; DMECs, Dermal microvessel endothelial cells; Dmt, *N,N*-Dimethyltryptamine; ES, Embryonic stem cells; F2, Diphenyl; GICs, Glioma-initiating cells; HASMCs, primary human aortic smooth muscle cells; HCE, Human corneal epithelial; HCEC, Human corneal epithelial cells; HCM, Human cardiac myocytes; HEK, Human embryonic kidney; HEL, Human erythroleukemia cell; Hex, Hexyl; hfRPE, Human fetal retinal pigment epithelial cells; hMSCs, Human mesenchymal stem cells; HMVEC, Human lung microvascular endothelial cells; HUVECs, Human umbilical vein endothelial cells; MDCK, Madin-Darby canine kidney; Met, Methylation; Nap, Naphthylalanine; NHC, Human conjunctival epithelial cells; NHDF, Normal human dermal fibroblasts; NPs, Nanoparticles; RAECM, Rat alveolar epithelial cell monolayers; SDHCEC, Spontaneously derived human corneal epithelial cells; Sip, Silaproline; X, Cyclohexylalanine; , Naphthylalanone; - No name attributed; * Different peptides with similar designations; | | | | | | | | | | | | | | | |

**Table S3. Features of published cell-penetrating peptides (cont.).**

| **Peptide** | **Typical sequence** | **Main cargoes** | **Cell model** | **Diseases** | **Proposed translocation** | **Physicochemical properties** | | | | | | | | | **Ref.** |
| --- | --- | --- | --- | --- | --- | --- | --- | --- | --- | --- | --- | --- | --- | --- | --- |
|  |  |  |  |  |  | **Molecular weight**  **(g/mol)** | **Extinction coefficient**  **(M^-1^.cm^-1^)** | **Hydrophobic**  **(%)** | **Isoelectric point** | **Net charge**  **(pH 7)** | **Charge** | **Average hydrophobicity** | **Hydrophobicity**  **(pH 7)** | **Ratio hydrophilic residues**  **(%)** |  |
| CPP7 | (FFΦRRRRQ)c | Proteins | - HeLa, NIH3T3, A549, H1299 - Lipid vesicles |  | Endocytosis | 1212,45 | 0 | 37,5 | 12,97 | 5 | 5 | 0,6 | 28,12 | 63 | [177] |
| CPP8 | (RFRFRΦRQ)c | Proteins | - HeLa, NIH3T3, A549, H1299 - Lipid vesicles |  | Endocytosis | 1212,45 | 0 | 37,5 | 12,97 | 5 | 5 | 0,6 | 28,12 | 63 | [177] |
| CPP9 | (fΦRrRrQ)c | Proteins | - HeLa, NIH3T3, A549, H1299 - Lipid vesicles |  | Endocytosis | 1065,27 | 0 | 28,57 | 12,97 | 5 | 5 | 1 | 18,29 | 71 | [177] |
| CPP10 | (rRFRΦRQ)c | Proteins | - HeLa, NIH3T3, A549, H1299 - Lipid vesicles |  | Endocytosis | 1065,27 | 0 | 28,57 | 12,97 | 5 | 5 | 1 | 18,29 | 71 | [177] |
| CPP11 | (fΦRrRrRQ)c | Proteins | - HeLa, NIH3T3, A549, H1299 - Lipid vesicles |  | Endocytosis | 1221,46 | 0 | 25 | 13,16 | 6 | 6 | 1,3 | 14,25 | 75 | [177] |
| CPP12 | (FfΦRrRrQ)c | Proteins | - HeLa, NIH3T3, A549, H1299 - Lipid vesicles |  | Endocytosis | 1212,45 | 0 | 37,5 | 12,97 | 5 | 5 | 0,6 | 28,12 | 63 | [177,179] |
| CPP12 (15-NF) | ((Dap)RRRRQ)c |  | - HeLa |  |  | 841,99 | 0 | 16,67 | 12,97 | 5 | 5 | 1,9 | -4,17 | 83 | [179] |
| CPP12 (16-NF) | (KRRRRQ)c |  | - HeLa |  |  | 899,09 | 0 | 0 | 12,97 | 6 | 6 | 2,5 | -14,83 | 100 | [179] |
| CPP12 (17-NF) | (DRRRRQ)c |  | - HeLa |  |  | 886,00 | 0 | 0 | 12,49 | 4 | 4 | 2,5 | -20,17 | 100 | [179] |
| CPP13 | (FϕrRrRQ)c | Proteins | - HeLa, NIH3T3, A549, H1299 - Lipid vesicles |  | Endocytosis | 1065,27 | 0 | 28,57 | 12,97 | 5 | 5 | 51 | 18,29 | 71 | [177] |
| CPP14 | (FWRRRRQ)c | Proteins | - HeLa, NIH3T3, A549, H1299 - Lipid vesicles |  | Endocytosis | 1104,31 | 5690 | 28,57 | 12,97 | 5 | 5 | 0,9 | 18,29 | 71 | [177] |
| CPP15 | (YΦRRRRQ)c | Proteins | - HeLa, NIH3T3, A549, H1299 - Lipid vesicles |  | Endocytosis | 1081,27 | 1280 | 28,57 | 12,49 | 5 | 5 | 1,1 | 13,43 | 71 | [177] |
| CPP16 | (HΦRRRRQ)c | Proteins | - HeLa, NIH3T3, A549, H1299 - Lipid vesicles |  | Endocytosis | 1055,24 | 0 | 14,29 | 12,97 | 5,11 | 6 | 1,3 | 5,57 | 71 | [177] |
| CPP17 | (Phg)ΦRRRRQ)c | Proteins | - HeLa, NIH3T3, A549, H1299 - Lipid vesicles |  | Endocytosis | 1051,24 | 0 | 16,67 | 12,97 | 5 | 5 | 1,4 | 5,17 | 71 | [177] |
| CPP18 | (FϕrRrRq)c | Proteins | - HeLa, NIH3T3, A549, H1299 - Lipid vesicles |  | Endocytosis | 1065,27 | 0 | 28,57 | 12,97 | 5 | 5 | 1 | 18,29 | 71 | [177] |
| CPP33 | RLWMRWYSPRTRAYGC | NPs | - A549 - Mice | Cancer | Endocytosis | 2102,53 | 14060 | 50 | 11,15 | 4,91 | 5 | -0,3 | 30,62 | 31 | [180,181] |
| CPP(I) | LAGrrrrrrrrrk |  | - Mice | Cancer |  | 1793,21 | 0 | 15,38 | 13,6 | 11 | 11 | 2,1 | -0,62 | 77 | [148] |
| Aca, Aminocaproic acid; AEC, Aortic endothelial cells; Ahx, 6-aminohexanoic acid; Aib, 2-aminoisobutyric acid; B-ALL, B acute lymphoblastic leukemia; BBMVEC, Bovine brain microvascular endothelial cells; BCECs, Brain capillary endothelial cells; BHK, Baby hamster kidney; BMB, 3,5-bis(mercaptomethyl)benzoyl; BMDCs, Bone marrow-derived dendritic cells; BMEC, Brain microvascular endothelial cells; Cha, Cyclohexylalanine; CHO, Chinese hamster ovary; Dap, Diaminopimelic acid; DC2.4, Mouse dendritic cells; DMECs, Dermal microvessel endothelial cells; Dmt, *N,N*-Dimethyltryptamine; ES, Embryonic stem cells; F2, Diphenyl; GICs, Glioma-initiating cells; HASMCs, primary human aortic smooth muscle cells; HCE, Human corneal epithelial; HCEC, Human corneal epithelial cells; HCM, Human cardiac myocytes; HEK, Human embryonic kidney; HEL, Human erythroleukemia cell; Hex, Hexyl; hfRPE, Human fetal retinal pigment epithelial cells; hMSCs, Human mesenchymal stem cells; HMVEC, Human lung microvascular endothelial cells; HUVECs, Human umbilical vein endothelial cells; MDCK, Madin-Darby canine kidney; Met, Methylation; Nap, Naphthylalanine; NHC, Human conjunctival epithelial cells; NHDF, Normal human dermal fibroblasts; NPs, Nanoparticles; RAECM, Rat alveolar epithelial cell monolayers; SDHCEC, Spontaneously derived human corneal epithelial cells; Sip, Silaproline; X, Cyclohexylalanine; , Naphthylalanone; - No name attributed; * Different peptides with similar designations; | | | | | | | | | | | | | | | |

**Table S3. Features of published cell-penetrating peptides (cont.).**

| **Peptide** | **Typical sequence** | **Main cargoes** | **Cell model** | **Diseases** | **Proposed translocation** | **Physicochemical properties** | | | | | | | | | **Ref.** |
| --- | --- | --- | --- | --- | --- | --- | --- | --- | --- | --- | --- | --- | --- | --- | --- |
|  |  |  |  |  |  | **Molecular weight**  **(g/mol)** | **Extinction coefficient**  **(M^-1^.cm^-1^)** | **Hydrophobic**  **(%)** | **Isoelectric point** | **Net charge**  **(pH 7)** | **Charge** | **Average hydrophobicity** | **Hydrophobicity**  **(pH 7)** | **Ratio hydrophilic residues**  **(%)** |  |
| CPP(II) | EEGRLYMRYYSPTTRRYG | Small drugs | - HepG2 | Cancer |  | 2298,64 | 5120 | 33,33 | 9,81 | 3 | 3 | 0,3 | 15,72 | 39 | [182] |
| CPP(III) | GAFPHR | NPs | - Bacteria | Infectious Diseases |  | 683,79 | 0 | 33,33 | 11,18 | 2,11 | 3 | -0,1 | 14,33 | 17 | [183] |
| CPP(IV) | RRRRRRGGRRRRG | NPs | - B16F10 |  |  | 1751,09 | 0 | 0 | 13,66 | 11 | 11 | 2,3 | -10,77 | 77 | [184] |
| CPP-C | PIEVCMYREP | Proteins | - IEC6 - Mice |  |  | 1236,51 | 1400 | 50 | 4,12 | 0,09 | 0 | 0,1 | 19,3 | 30 | [185] |
| CRHC | CRRRRRHHHHHHHRRRRC | Nucleic acids | - ARPE-19, hfRPE | Ocular disorders |  | 2590,05 | 240 | 11,11 | 12,81 | 10,6 | 17 | 1,2 | 1,56 | 50 | [186] |
| cHLH | GARELRRLERELRRLE | Proteins | - MCF-10A, MM96L, MDA-MB-231, MDA-MB-435S, HFF-1 - Lipid vesicles | Cancer |  | 2052,45 | 0 | 31,25 | 11,65 | 3 | 3 | 1,4 | 14,56 | 63 | [187] |
| CIGB-552 | HARIKPTFRRLKWKYKGKFW |  | - H-460, HT-29 - Mice | Cancer |  | 2647,32 | 12660 | 40 | 12,26 | 9,11 | 10 | 0,2 | 25,45 | 40 | [188] |
| CK30 | KKKKKKKKKKKKKKKKKKKKKKKKKKKKKKKKKKKKKKKK | Proteins | - HER - Mice | Ocular disorders |  | 5145,21 | 0 | 0 | 12,28 | 40 | 40 | 3 | -23 | 100 | [189] |
| CLIP6 | KVRVRVRVpPTRVRERVK | Proteins, nucleic acids | - U87, BMDCs, DC2.4 - Mice | Cancer | Endocytosis-independent, direct internalization | 2133,7 | 0 | 35,29 | 12,82 | 8 | 8 | 1 | 15,41 | 53 | [190,191] |
| CNT | CGRKKRRQRRR | NPs | - A431 | Cancer |  | 1499,85 | 120 | 9,09 | 12,82 | 8,91 | 9 | 2,1 | -8,27 | 82 | [192] |
| Con.P | ARPLEHGSDKAT |  | - HIG-82, Clu-3, CHO, 293, PC-3 - Mice | Inflammatory disorders |  | 1281,44 | 0 | 25 | 7,99 | 1,11 | 2 | 0,7 | 2,42 | 42 | [193] |
| Crotamine | YKQCHKKGGHCFPKEKICLPPSSDFGKMDCRWRWKCCKKGS |  | - Mouse ES, Human primary fibroblasts, Lymphoblasts  - Lipid vesicles |  |  | 4832,95 | 13380 | 34,15 | 9,69 | 8,68 | 11 | 0,4 | 12 | 44 | [194,195] |
| CTP | APWHLSSQYSRT | Protein | - Vero E6, U-2OS, CHO, H9C2, NIH3T3, C57/BL6, HeLa, HK-2 - Mice | Cardiovascular Diseases |  | 1432,61 | 6970 | 33,33 | 10,02 | 2,11 | 3 | -0,4 | 19,75 | 42 | [196-198] |
| CVP1 | MARRARRPRGRFYAFRRGR | Proteins | - 293T, HCT-116, NIH3T3, MDCK, MSB1 |  | Caveolae-mediated endocytosis | 2436,94 | 1280 | 36,84 | 13,05 | 10 | 10 | 0,9 | 14,84 | 47 | [199] |
| CVP1 (Mut) | MAAGAGAPAGAFYAFGAGA | Proteins | - 293T, HCT-116, NIH3T3, MDCK, MSB1 |  | Caveolae-mediated endocytosis | 1628,89 | 1280 | 68,42 | 6 | 1 | 1 | -0,7 | 34,42 | 0 | [199] |
| CyLoP-1 | CRWRWKCCKK | NPs, nucleic acids | - HASMCs, NIH3T3, C6, PANC-1, HeLa, CCL-11 | Cardiovascular Diseases |  | 1396,79 | 11740 | 50 | 10,41 | 5,73 | 6 | 0,5 | 24,4 | 50 | [200,201] |
| Aca, Aminocaproic acid; AEC, Aortic endothelial cells; Ahx, 6-aminohexanoic acid; Aib, 2-aminoisobutyric acid; B-ALL, B acute lymphoblastic leukemia; BBMVEC, Bovine brain microvascular endothelial cells; BCECs, Brain capillary endothelial cells; BHK, Baby hamster kidney; BMB, 3,5-bis(mercaptomethyl)benzoyl; BMDCs, Bone marrow-derived dendritic cells; BMEC, Brain microvascular endothelial cells; Cha, Cyclohexylalanine; CHO, Chinese hamster ovary; Dap, Diaminopimelic acid; DC2.4, Mouse dendritic cells; DMECs, Dermal microvessel endothelial cells; Dmt, *N,N*-Dimethyltryptamine; ES, Embryonic stem cells; F2, Diphenyl; GICs, Glioma-initiating cells; HASMCs, primary human aortic smooth muscle cells; HCE, Human corneal epithelial; HCEC, Human corneal epithelial cells; HCM, Human cardiac myocytes; HEK, Human embryonic kidney; HEL, Human erythroleukemia cell; Hex, Hexyl; hfRPE, Human fetal retinal pigment epithelial cells; hMSCs, Human mesenchymal stem cells; HMVEC, Human lung microvascular endothelial cells; HUVECs, Human umbilical vein endothelial cells; MDCK, Madin-Darby canine kidney; Met, Methylation; Nap, Naphthylalanine; NHC, Human conjunctival epithelial cells; NHDF, Normal human dermal fibroblasts; NPs, Nanoparticles; RAECM, Rat alveolar epithelial cell monolayers; SDHCEC, Spontaneously derived human corneal epithelial cells; Sip, Silaproline; X, Cyclohexylalanine; , Naphthylalanone; - No name attributed; * Different peptides with similar designations; | | | | | | | | | | | | | | | |

**Table S3. Features of published cell-penetrating peptides (cont.).**

| **Peptide** | **Typical sequence** | **Main cargoes** | **Cell model** | **Diseases** | **Proposed translocation** | **Physicochemical properties** | | | | | | | | | **Ref.** |
| --- | --- | --- | --- | --- | --- | --- | --- | --- | --- | --- | --- | --- | --- | --- | --- |
|  |  |  |  |  |  | **Molecular weight**  **(g/mol)** | **Extinction coefficient**  **(M^-1^.cm^-1^)** | **Hydrophobic**  **(%)** | **Isoelectric point** | **Net charge**  **(pH 7)** | **Charge** | **Average hydrophobicity** | **Hydrophobicity**  **(pH 7)** | **Ratio hydrophilic residues**  **(%)** |  |
| Cyt c5-13 | KGKKIFIMK |  | - RBL-2H3 |  |  | 1092,51 | 0 | 44,44 | 11,11 | 5 | 5 | 0,5 | 30,78 | 44 | [173] |
| CAAKA | CAAKA | NPs | - A375, MSTO, rat 9L, U87, LN18 | Cancer | Endocytosis | 462,57 | 120 | 80 | 8,93 | 1,91 | 2 | 0,1 | 29,8 | 20 | [202] |
| CADY | GLWRALWRLLRSLWRLLWRA | Nucleic acid | - Hs68, U2OS, THP1, 3T3C, HUVECs  - Lipid vesicles  - Mice | Cancer, inflammatory disorders |  | 2622,33 | 22760 | 65 | 13,16 | 6 | 6 | -0,6 | 54,75 | 30 | [203-208] |
| CADY-K | GLWRALWRLLRSLWRLLWK | NPs | - Lipid vesicles |  |  | 2523,24 | 22760 | 63,16 | 12,97 | 6 | 6 | -0,6 | 55 | 32 | [209] |
| D-CADY-K | glwralwrllrslwrllwk | NPs | - Lipid vesicles |  |  | 2523,24 | 22760 | 63,16 | 12,97 | 6 | 6 | -0,6 | 55 | 32 | [209] |
| CADY 2 | GLWWRLWWRLRSWFRLWFRA | Proteins, nucleic acids | - HEK-293T, Hs68 | Cancer |  | 2878,54 | 34140 | 65 | 13,16 | 6 | 6 | -0,9 | 57,1 | 30 | [203,210] |
| CC12 | EMFTPPSMIERLK | Protein | - ARPE-19, HUVECs - Mice | Ocular disorders |  | 1578,97 | 0 | 30,77 | 7,07 | 1 | 1 | 0,2 | 20,08 | 38 | [211] |
| CCMV Gag | KLTRAQRRAAARKNKRNTR |  | - Lipid vesicles |  |  | 2295,76 | 0 | 26,32 | 13,33 | 10 | 10 | 1,2 | 2,37 | 63 | [162] |
| CCP2 | DSLKSYWYLQKFSWR | Proteins | - Jurkat T, CHO, HeLa, Lovo, A549, MCF-7, U2OS, HepG2, K562, NHDF - Rats | Cancer | Dynamin-dependent endocytosis | 2007,35 | 13940 | 46,67 | 10,05 | 3 | 3 | -0,3 | 31,8 | 53 | [212] |
| CCP44 | KRPTMRFRYTWNPMK | Proteins | - Jurkat T, CHO, HeLa, Lovo, A549, MCF-7, U2OS, HepG2, K562, NHDF - Rats | Cancer | Dynamin-dependent endocytosis | 2012,49 | 6970 | 33,33 | 12,21 | 6 | 6 | 0,2 | 14 | 40 | [212] |
| CF-14 | RIVELTLPRVSVRL |  | - Bacteria | Infectious Diseases |  | 1651,11 | 0 | 50 | 12,17 | 3 | 3 | 0 | 36,86 | 36 | [213] |
| DPT-sh1 | VKKKKIKREIKI | Small drugs | - Virus | Infectious Diseases |  | 1511,05 | 0 | 33,33 | 11,36 | 7 | 7 | 1,4 | 15,83 | 67 | [214] |
| DPT-sh2 | RQKRLIRQKRLIRQKRLI | Small drugs | - Virus | Infectious Diseases |  | 2403,13 | 0 | 33,33 | 13,33 | 10 | 10 | 0,9 | 23 | 67 | [214] |
| DPV3  (Vectocell 3) | RKKRRRESRKKRRRES | Proteins | - L02, HUVEC, ECV304, HeLa, MDA-MB-435, DA-MB-231, HCM, MCF-7, HK-2, A375 | Cancer | Direct internalization | 2212,65 | 0 | 0 | 12,74 | 11 | 11 | 2,7 | -17,25 | 100 | [158,215] |
| DQ 65–79 | NIAVLKHNLNIVIKR |  | - CTL | Cancer |  | 1745,25 | 0 | 53,33 | 11,77 | 4,11 | 5 | -0,2 | 34,33 | 40 | [216] |
| DRIM | QQRKRKIWSILAPLGTTLVKLVAGIG | Proteins | - HEK293, HeLa - Bacteria | Infectious Diseases |  | 2847,66 | 5690 | 46,15 | 12,54 | 6 | 6 | -0,2 | 34,08 | 31 | [217] |
| EB | RRKKAAVALLPAVLLALLAP |  | - HK320, THK320, Vero, HeLa | Infectious Diseases |  | 2084,77 | 0 | 70 | 12,53 | 5 | 5 | -0,2 | 41,6 | 20 | [218] |
| Aca, Aminocaproic acid; AEC, Aortic endothelial cells; Ahx, 6-aminohexanoic acid; Aib, 2-aminoisobutyric acid; B-ALL, B acute lymphoblastic leukemia; BBMVEC, Bovine brain microvascular endothelial cells; BCECs, Brain capillary endothelial cells; BHK, Baby hamster kidney; BMB, 3,5-bis(mercaptomethyl)benzoyl; BMDCs, Bone marrow-derived dendritic cells; BMEC, Brain microvascular endothelial cells; Cha, Cyclohexylalanine; CHO, Chinese hamster ovary; Dap, Diaminopimelic acid; DC2.4, Mouse dendritic cells; DMECs, Dermal microvessel endothelial cells; Dmt, *N,N*-Dimethyltryptamine; ES, Embryonic stem cells; F2, Diphenyl; GICs, Glioma-initiating cells; HASMCs, primary human aortic smooth muscle cells; HCE, Human corneal epithelial; HCEC, Human corneal epithelial cells; HCM, Human cardiac myocytes; HEK, Human embryonic kidney; HEL, Human erythroleukemia cell; Hex, Hexyl; hfRPE, Human fetal retinal pigment epithelial cells; hMSCs, Human mesenchymal stem cells; HMVEC, Human lung microvascular endothelial cells; HUVECs, Human umbilical vein endothelial cells; MDCK, Madin-Darby canine kidney; Met, Methylation; Nap, Naphthylalanine; NHC, Human conjunctival epithelial cells; NHDF, Normal human dermal fibroblasts; NPs, Nanoparticles; RAECM, Rat alveolar epithelial cell monolayers; SDHCEC, Spontaneously derived human corneal epithelial cells; Sip, Silaproline; X, Cyclohexylalanine; , Naphthylalanone; - No name attributed; * Different peptides with similar designations; | | | | | | | | | | | | | | | |

**Table S3. Features of published cell-penetrating peptides (cont.).**

| **Peptide** | **Typical sequence** | **Main cargoes** | **Cell model** | **Diseases** | **Proposed translocation** | **Physicochemical properties** | | | | | | | | | **Ref.** |
| --- | --- | --- | --- | --- | --- | --- | --- | --- | --- | --- | --- | --- | --- | --- | --- |
|  |  |  |  |  |  | **Molecular weight**  **(g/mol)** | **Extinction coefficient**  **(M^-1^.cm^-1^)** | **Hydrophobic**  **(%)** | **Isoelectric point** | **Net charge**  **(pH 7)** | **Charge** | **Average hydrophobicity** | **Hydrophobicity**  **(pH 7)** | **Ratio hydrophilic residues**  **(%)** |  |
| EB1 | LIRLWSHLIHIWFQNRRLKWKKK | Nucleic acids | - HeLa, HepG2  - Lipid vesicles |  | Endocytosis | 3100,96 | 17070 | 47,83 | 12,83 | 8,22 | 10 | -0,2 | 39,61 | 43 | [168,204] |
| Erns | RQGAARVTSWLGLQLRIGK | Proteins | - Rats | Metabolic disorders |  | 2110,61 | 5690 | 42,11 | 12,82 | 5 | 5 | 0 | 30,37 | 37 | [219] |
| F2Pal10 | KIHKKGMIKS |  | - HL-60 |  | G-protein coupled receptors | 1169,56 | 0 | 30 | 11,11 | 5,11 | 6 | 0,7 | 18,3 | 50 | [220] |
| F2pal10K5 → R | KIHKRGMIKS |  | - HL-60 |  | G-protein coupled receptors | 1197,57 | 0 | 30 | 11,86 | 5,11 | 6 | 0,7 | 19,2 | 50 | [220] |
| F2Pal16 | KIHKKGMIKSSRPLRV |  | - HL-60 |  | G-protein coupled receptors | 1878,45 | 0 | 31,25 | 12,56 | 7,11 | 8 | 0,6 | 17,5 | 50 | [220] |
| F3 | KDEPQRRSARLSAKPAPPKPEPKPKKAPAKKC | NPs | - MDA-MB-231, HUVECs - Mice | Cancer |  | 3536,32 | 120 | 21,88 | 11,01 | 8,91 | 9 | 1,2 | -11,78 | 53 | [221] |
| FAKCPP | FAKLAARLYR | Proteins | - THP-1 human monocytes | Inflammatory disorders |  | 1208,51 | 1280 | 70 | 11,46 | 4 | 4 | -0,1 | 43,2 | 30 | [222] |
| FAM-1 | ARR(Aib)RR(Aib)RR(Aib) | Nucleic acids | - HeLa |  | Endocytosis | 1196,47 | 0 | 14,29 | 13,33 | 7 | 7 | 2,5 | -6,14 | 86 | [223] |
| ent-FAM-1 | Arr(Aib)rr(Aib)rr(Aib) | Nucleic acids | - HeLa |  | Endocytosis | 1196,47 | 0 | 14,29 | 13,33 | 7 | 7 | 2,5 | -6,14 | 86 | [223] |
| FAM-2 | ARr(Aib)Rr(Aib)Rr(Aib) | Nucleic acids | - HeLa |  | Endocytosis | 1196,47 | 0 | 14,29 | 13,33 | 7 | 7 | 2,5 | -6,14 | 86 | [223] |
| FHV | RTRRNRRRVR | Proteins | - EBTr |  |  | 1425,7 | 0 | 10 | 13,45 | 8 | 8 | 1,9 | -5 | 80 | [156] |
| FHV coat | RRRRNRTRRNRRRVR | Proteins, NPs, nucleic acids | - COS-7, CHO-K1, HeLa, Jurkat T, mouse ES, GICs  - Lipid vesicles - Mice | Cancer | Macropinocytosis | 2164,57 | 0 | 6,67 | 13,7 | 12 | 12 | 2,1 | -9,8 | 87 | [161-163,224,225] |
| FHV-TA | NRARRNRRRVR | Proteins | - EBTr |  |  | 1509,78 | 0 | 18,18 | 13,45 | 8 | 8 | 1,8 | -5,73 | 82 | [156] |
| FL20 | GIGAILKVLATGLPTLISWI | Nucleic acids | - 293FT, B16F10, CHO-K1 |  |  | 2036,67 | 5690 | 60 | 10,28 | 2 | 2 | -0,9 | 50,15 | 10 | [226] |
| FT2 | VRLPPPVRLPPPVK(fatty acid)LPPP |  | - HeLa |  |  | 1969,58 | 0 | 33,33 | 12,51 | 4 | 4 | 0 | 3,5 | 17 | [227] |
| FUSO | CGLFEALLELLESLWELLLEA | NPs | - MPyV virus | Infectious Diseases |  | 2404,98 | 5810 | 66,67 | 2,76 | -4,09 | -4 | -0,4 | 50,71 | 29 | [228] |
| GALA | WEAALAEALAEALAEHLAEALAEALEALAA | NPs, nucleic acid | - HMVEC-L - Lipid vesicles  - Mice | Cancer | Endocytosis | 3032,53 | 5690 | 73,33 | 3,23 | -5,88 | -5 | -0,1 | 38,73 | 23 | [229-231] |
| Aca, Aminocaproic acid; AEC, Aortic endothelial cells; Ahx, 6-aminohexanoic acid; Aib, 2-aminoisobutyric acid; B-ALL, B acute lymphoblastic leukemia; BBMVEC, Bovine brain microvascular endothelial cells; BCECs, Brain capillary endothelial cells; BHK, Baby hamster kidney; BMB, 3,5-bis(mercaptomethyl)benzoyl; BMDCs, Bone marrow-derived dendritic cells; BMEC, Brain microvascular endothelial cells; Cha, Cyclohexylalanine; CHO, Chinese hamster ovary; Dap, Diaminopimelic acid; DC2.4, Mouse dendritic cells; DMECs, Dermal microvessel endothelial cells; Dmt, *N,N*-Dimethyltryptamine; ES, Embryonic stem cells; F2, Diphenyl; GICs, Glioma-initiating cells; HASMCs, primary human aortic smooth muscle cells; HCE, Human corneal epithelial; HCEC, Human corneal epithelial cells; HCM, Human cardiac myocytes; HEK, Human embryonic kidney; HEL, Human erythroleukemia cell; Hex, Hexyl; hfRPE, Human fetal retinal pigment epithelial cells; hMSCs, Human mesenchymal stem cells; HMVEC, Human lung microvascular endothelial cells; HUVECs, Human umbilical vein endothelial cells; MDCK, Madin-Darby canine kidney; Met, Methylation; Nap, Naphthylalanine; NHC, Human conjunctival epithelial cells; NHDF, Normal human dermal fibroblasts; NPs, Nanoparticles; RAECM, Rat alveolar epithelial cell monolayers; SDHCEC, Spontaneously derived human corneal epithelial cells; Sip, Silaproline; X, Cyclohexylalanine; , Naphthylalanone; - No name attributed; * Different peptides with similar designations; | | | | | | | | | | | | | | | |

**Table S3. Features of published cell-penetrating peptides (cont.).**

| **Peptide** | **Typical sequence** | **Main cargoes** | **Cell model** | **Diseases** | **Proposed translocation** | **Physicochemical properties** | | | | | | | | | **Ref.** |
| --- | --- | --- | --- | --- | --- | --- | --- | --- | --- | --- | --- | --- | --- | --- | --- |
|  |  |  |  |  |  | **Molecular weight**  **(g/mol)** | **Extinction coefficient**  **(M^-1^.cm^-1^)** | **Hydrophobic**  **(%)** | **Isoelectric point** | **Net charge**  **(pH 7)** | **Charge** | **Average hydrophobicity** | **Hydrophobicity**  **(pH 7)** | **Ratio hydrophilic residues**  **(%)** |  |
| GET | TYRSRKYTSWYVALKRKLLKLLLKLLLKLLKRRRRRRRR | Nucleic acid | - hMSCs, MC3T3, Human articular chondrocytes, C2C12, Human dermal fibroblasts, HUVECs, HL-1 cardiac , Ne4C - Rats |  |  | 5100,59 | 9530 | 43,59 | 12,77 | 18,99 | 19 | 0,6 | 30,87 | 51 | [232] |
| gH625 | HGLASTLTRWAHYNALIRAF | NPs, nucleic acids | - HeLa, MDA-MB-231  - Lipid vesicles | Cancer | Endocytosis, caveolae-mediated endocytosis | 2298,74 | 6970 | 55 | 11,21 | 3,22 | 5 | -0,6 | 39,4 | 20 | [233-241] |
| gp41 | GALFLGWLGAAGSTMGA | NPs | - A375, MSTO, rat 9L, U87, LN18 | Cancer | Endocytosis | 1579,93 | 5690 | 58,82 | 13,8 | 1 | 1 | -0,9 | 43,53 | 6 | [202] |
| H16 | HHHHHHHHHHHHHHHH | NPs | - HT1080 |  | Endocytosis | 2212,41 | 0 | 0 | 13,8 | 2,79 | 17 | -0,5 | 8 | 0 | [242] |
| H3K(+H)4b | K(KHHHKHHHKHHHHKHHHK)2(K(KHHHKHHHKHHHHKHHHK)2 | Nucleic acids | - MDA-MB-435, SVRbag4, HUVEC | Cancer |  | 9969,77 | 0 | 0 | 11,95 | 28,81 | 75 | 0,5 | -1,22 | 30 | [243] |
| H3K8b | K((HHHKHHHKHHHKHHHK)2KHHHHNHHHHK)(K(HHHHNHHHHK(HHHKHHHKHHHKHHHK2))2 | Nucleic acids | - MDA-MB-435, SVRbag4, HUVEC | Cancer |  | 17372,14 | 0 | 0 | 12 | 41,72 | 127 | 0,3 | -0,35 | 26 | [243] |
| HAP-1 | SFHQFARATLAS | Proteins | - HIG-82, Clu-3, CHO, 293, PC-3 - Mice | Inflammatory disorders |  | 1335,53 | 0 | 50 | 11,18 | 2 | 3 | -0,4 | 33,67 | 33 | [193] |
| HAP-2 | HIQLSPFSQSWR |  | - HIG-82, Clu-3, CHO, 293, PC-3 - Mice | Inflammatory disorders |  | 1485,73 | 5690 | 33,33 | 11,18 | 2,11 | 3 | -0,5 | 25,5 | 50 | [193] |
| hBD3-3 | GKCSTRGRKCCRRKK |  | - RAW 264.7 - Mice | Inflammatory disorders | Endocytosis | 1767,22 | 360 | 20 | 11,54 | 8,72 | 9 | 1,4 | 0,47 | 60 | [244] |
| HBHAc | KKAAPAKKAAAKKAPAKKAAAKK | Proteins | - HeLa, MDA-MB-231, PC3 - Mice | Cancer |  | 2275,93 | 0 | 47,83 | 11,71 | 11 | 11 | 1,1 | 5,61 | 43 | [245] |
| hCT(9-32) | LGTYTQDFNKFHTFPQTAIGVGAP | NPs | - MDCK, HeLa, A375, MSTO, rat 9L, U87, LN18, Bovine nasal mucosa | Cancer | Endocytosis | 2611,04 | 1280 | 37,5 | 7,92 | 1,11 | 2 | -0,5 | 22,5 | 21 | [202,246,247] |
| hCR(12-32) | YTQDFNKFHTFPQTAIGVGAP |  | - MDCK, HeLa |  | Endocytosis | 2339,7 | 1280 | 38,1 | 7,92 | 1,11 | 2 | -0,4 | 20,33 | 24 | [247] |
| hCT(18-32)-K7 | K(KKRKAPKKKRKFA)FHTFPQTAIGVGAP | Proteins, nucleic acids | - HeLa, MCF-7, HEK293, HT-29, FaDu - Mice | Cancer | Endocytosis | 3166,99 | 0 | 32,14 | 12,61 | 11,11 | 12 | 0,6 | 10,86 | 39 | [248,249] |
| hLactoferrin | KCFQWQRNMRKVRGPPVSCIKR |  | - HeLa, IEC-8 |  | Direct internalization | 2718,41 | 5930 | 36,36 | 12,06 | 7,82 | 8 | 0,4 | 15,18 | 50 | [250] |
| HP4 | RRRRPRRRTTRRRR | Proteins, nucleic acids | - MSCs, Dendritic cells, C6Bu1, CT26, B16F10, HeLa, U-87, K549, K562, A375 - Mice | Cancer |  | 2035,44 | 0 | 0 | 13,7 | 12 | 12 | 2,3 | -12,43 | 79 | [251,252] |
| Aca, Aminocaproic acid; AEC, Aortic endothelial cells; Ahx, 6-aminohexanoic acid; Aib, 2-aminoisobutyric acid; B-ALL, B acute lymphoblastic leukemia; BBMVEC, Bovine brain microvascular endothelial cells; BCECs, Brain capillary endothelial cells; BHK, Baby hamster kidney; BMB, 3,5-bis(mercaptomethyl)benzoyl; BMDCs, Bone marrow-derived dendritic cells; BMEC, Brain microvascular endothelial cells; Cha, Cyclohexylalanine; CHO, Chinese hamster ovary; Dap, Diaminopimelic acid; DC2.4, Mouse dendritic cells; DMECs, Dermal microvessel endothelial cells; Dmt, *N,N*-Dimethyltryptamine; ES, Embryonic stem cells; F2, Diphenyl; GICs, Glioma-initiating cells; HASMCs, primary human aortic smooth muscle cells; HCE, Human corneal epithelial; HCEC, Human corneal epithelial cells; HCM, Human cardiac myocytes; HEK, Human embryonic kidney; HEL, Human erythroleukemia cell; Hex, Hexyl; hfRPE, Human fetal retinal pigment epithelial cells; hMSCs, Human mesenchymal stem cells; HMVEC, Human lung microvascular endothelial cells; HUVECs, Human umbilical vein endothelial cells; MDCK, Madin-Darby canine kidney; Met, Methylation; Nap, Naphthylalanine; NHC, Human conjunctival epithelial cells; NHDF, Normal human dermal fibroblasts; NPs, Nanoparticles; RAECM, Rat alveolar epithelial cell monolayers; SDHCEC, Spontaneously derived human corneal epithelial cells; Sip, Silaproline; X, Cyclohexylalanine; , Naphthylalanone; - No name attributed; * Different peptides with similar designations; | | | | | | | | | | | | | | | |

**Table S3. Features of published cell-penetrating peptides (cont.).**

| **Peptide** | **Typical sequence** | **Main cargoes** | **Cell model** | **Diseases** | **Proposed translocation** | **Physicochemical properties** | | | | | | | | | **Ref.** |
| --- | --- | --- | --- | --- | --- | --- | --- | --- | --- | --- | --- | --- | --- | --- | --- |
|  |  |  |  |  |  | **Molecular weight**  **(g/mol)** | **Extinction coefficient**  **(M^-1^.cm^-1^)** | **Hydrophobic**  **(%)** | **Isoelectric point** | **Net charge**  **(pH 7)** | **Charge** | **Average hydrophobicity** | **Hydrophobicity**  **(pH 7)** | **Ratio hydrophilic residues**  **(%)** |  |
| Hph1 | YARVRRRGPRR | Proteins, nucleic acids | - HeLa, Dendritic cells, C6Bu1, CT26, B16F10, U-87, K549, K562, A375, MSCs, RAW 264.7, HCT116 - Rats, Mice | Cancer, cardiovascular Diseases |  | 1442,73 | 1280 | 27,27 | 12,81 | 7 | 7 | 1,2 | 4,55 | 55 | [251-255] |
| hPP 10 | KIPLPRFKLKCIFCKKRRKR | Proteins | - HEK-293T | Cancer |  | 2559,41 | 240 | 40 | 12,1 | 10,82 | 11 | 0,8 | 20,2 | 50 | [210] |
| hPP3 | KPKRKRRKKKGHGWSR | Proteins | - ECV304, HepG2, PC3, HeLa |  | Clathrin-mediated endocytosis | 2033,54 | 5690 | 6,25 | 12,98 | 11,11 | 12 | 1,6 | -8,75 | 69 | [256] |
| HR9 | CHHHHHHRRRRRRRRRHHHHHHC | NPs, nucleic acids | - HEK-293T, SK-OV-3 | Metabolic disorders |  | 3275,8 | 240 | 8,7 | 12,81 | 11,16 | 22 | 0,8 | 2,96 | 39 | [257,258] |
| HTLV-II Rex | TRRQRTRRARRNR | NPs | - CHO-K1, Caco-2, HeLa, Jurkat T  - Lipid vesicles |  | Macropinocytosis | 1783,08 | 0 | 7,69 | 13,53 | 9 | 9 | 1,8 | -7,38 | 77 | [161-163] |
| Human cFos | KRRIRRERNKMAAAKSRNRRRELTDT |  | - CHO-K1, HeLa, Jurkat T |  | Macropinocytosis | 3269,89 | 0 | 23,08 | 12,61 | 10 | 10 | 1,5 | 0,88 | 69 | [161] |
| Human cJun | RIKAERKRMRNRIAASKSRKRKLERIAR |  | - CHO-K1, HeLa, Jurkat T |  | Macropinocytosis | 3479,33 | 0 | 32,14 | 12,82 | 13 | 13 | 1,4 | 10,04 | 68 | [161] |
| I-6 | RI(Npg)(Dap)RLLQ | Proteins | - MCF-7, MDA-MB-231, A2780, 4T1 - Mice | Cancer |  | 855,1 | 0 | 42,86 | 12,49 | 3 | 3 | 0,1 | 37,29 | 43 | [259] |
| IDR-1018 | VRLIVAVRIWRR | Small drugs | - Virus | Infectious Diseases |  | 1537 | 5690 | 66,67 | 12,97 | 5 | 5 | -0,1 | 50,67 | 33 | [214] |
| Iduna | RRRKIKR | Proteins | - HeLa - Mice |  | Lipid-raft mediated endocytosis | 1012,3 | 0 | 14,29 | 12,97 | 7 | 7 | 2,3 | -0,43 | 86 | [255] |
| IMT-P8 | RRWRRWNRFNRRRCR | Proteins | - HeLa, PC3, MDA-MB-231 - Mice | Cancer |  | 2274,7 | 11500 | 26,67 | 13,05 | 9,91 | 10 | 1,1 | 8,8 | 73 | [260] |
| INF7-SGSC | GLFEAIEGFIENGWEGMINGWYGSGSC | Nucleic acids | - 293 |  |  | 2924,37 | 12780 | 44,44 | 3,8 | -3,08 | -3 | -0,4 | 29,48 | 30 | [261] |
| Integrin b3 | VTVLALGALAGVGVG | NPs | - A375, MSTO, rat 9L, U87, LN18 | Cancer | Endocytosis | 1296,67 | 0 | 66,67 | 13,8 | 1 | 1 | -0,9 | 49,33 | 0 | [202] |
| iRGD | RGDKGPDC | Proteins, NPs, small drugs | - HEK 293, A549, MIA PaCa-2, B16F10, HUVECs, U251, U373, H460, MCF-7, CT26, 4T1, HeLa, HepG2, PPC1, MDA-MB-435S, MCF-10A, BGC-823, DU145, LNCap, PC-3, U87, KYSE-150, K562, MDA-MB-231, BCPAP  - Mice | Cancer, ocular disorders | Endocytosis, raft-mediated endocytosis | 846,94 | 120 | 12,5 | 6,05 | 1,09 | 1 | 1,4 | -18 | 50 | [262-277] |
| K4 | KKKK | Proteins | - CHO K1, HeLa, A549, Jurkat T |  |  | 530,73 | 0 | 0 | 11,11 | 5 | 5 | 3 | -23 | 100 | [278] |
| Aca, Aminocaproic acid; AEC, Aortic endothelial cells; Ahx, 6-aminohexanoic acid; Aib, 2-aminoisobutyric acid; B-ALL, B acute lymphoblastic leukemia; BBMVEC, Bovine brain microvascular endothelial cells; BCECs, Brain capillary endothelial cells; BHK, Baby hamster kidney; BMB, 3,5-bis(mercaptomethyl)benzoyl; BMDCs, Bone marrow-derived dendritic cells; BMEC, Brain microvascular endothelial cells; Cha, Cyclohexylalanine; CHO, Chinese hamster ovary; Dap, Diaminopimelic acid; DC2.4, Mouse dendritic cells; DMECs, Dermal microvessel endothelial cells; Dmt, *N,N*-Dimethyltryptamine; ES, Embryonic stem cells; F2, Diphenyl; GICs, Glioma-initiating cells; HASMCs, primary human aortic smooth muscle cells; HCE, Human corneal epithelial; HCEC, Human corneal epithelial cells; HCM, Human cardiac myocytes; HEK, Human embryonic kidney; HEL, Human erythroleukemia cell; Hex, Hexyl; hfRPE, Human fetal retinal pigment epithelial cells; hMSCs, Human mesenchymal stem cells; HMVEC, Human lung microvascular endothelial cells; HUVECs, Human umbilical vein endothelial cells; MDCK, Madin-Darby canine kidney; Met, Methylation; Nap, Naphthylalanine; NHC, Human conjunctival epithelial cells; NHDF, Normal human dermal fibroblasts; NPs, Nanoparticles; RAECM, Rat alveolar epithelial cell monolayers; SDHCEC, Spontaneously derived human corneal epithelial cells; Sip, Silaproline; X, Cyclohexylalanine; , Naphthylalanone; - No name attributed; * Different peptides with similar designations; | | | | | | | | | | | | | | | |

**Table S3. Features of published cell-penetrating peptides (cont.).**

| **Peptide** | **Typical sequence** | **Main cargoes** | **Cell model** | **Diseases** | **Proposed translocation** | **Physicochemical properties** | | | | | | | | | **Ref.** |
| --- | --- | --- | --- | --- | --- | --- | --- | --- | --- | --- | --- | --- | --- | --- | --- |
|  |  |  |  |  |  | **Molecular weight**  **(g/mol)** | **Extinction coefficient**  **(M^-1^.cm^-1^)** | **Hydrophobic**  **(%)** | **Isoelectric point** | **Net charge**  **(pH 7)** | **Charge** | **Average hydrophobicity** | **Hydrophobicity**  **(pH 7)** | **Ratio hydrophilic residues**  **(%)** |  |
| D-K4 | Kkkk | Nucleic acids | - Virus - Ducks | Infectious Diseases |  | 530,73 | 0 | 0 | 11,11 | 5 | 5 | 3 | -23 | 100 | [279] |
| K5(QW)6K5 | KKKKKQWQWQWQWQWQWKKKKK | Proteins | - HeLa,HEK293 |  |  | 3185,97 | 34140 | 27,27 | 11,71 | 11 | 11 | 0,5 | 13,27 | 73 | [280] |
| K6 | KKKKKK | Proteins | - CHO K1, HeLa, A549, Jurkat T |  |  | 787,09 | 0 | 0 | 11,44 | 7 | 7 | 3 | -23 | 100 | [278] |
| D-K6L9 | LKLLKKLLKKLLKLL | Nucleic acids | - CHO, Cos-7 |  |  | 1805,62 | 0 | 60 | 11,44 | 7 | 7 | 0,1 | 50,8 | 40 | [281] |
| K8 | KKKKKKKK | Proteins, nucleic acids | - CHO K1, HeLa, A549, T  - Bacteria | Infectious Diseases |  | 1043,45 | 0 | 0 | 11,61 | 9 | 9 | 3 | -23 | 100 | [278,282] |
| K9 | KKKKKKKKK | Proteins | - HeLa |  |  | 1171,63 | 0 | 0 | 11,67 | 10 | 10 | 3 | -23 | 100 | [283] |
| D-K9 | Kkkkkkkkk | Proteins | - RAECM, HepG2 - Rats | Metabolic disorders |  | 1171,63 | 0 | 0 | 11,67 | 10 | 10 | 3 | -23 | 100 | [284] |
| K10 | KKKKKKKKKK | Proteins | - CHO K1, HeLa, A549, Jurkat T |  |  | 1299,81 | 0 | 0 | 11,71 | 11 | 11 | 3 | -23 | 100 | [278] |
| K10(QW)6 | KKKKKKKKKKQWQWQWQWQWQW | Proteins | - HeLa, HEK293 |  |  | 3185,97 | 34140 | 27,27 | 11,71 | 11 | 11 | 0,5 | 13,27 | 73 | [280] |
| K12 | KKKKKKKKKKKK | Proteins | - CHO K1, HeLa, A549, Jurkat T |  |  | 1556,17 | 0 | 0 | 11,79 | 13 | 13 | 3 | -23 | 100 | [278] |
| KAFAK | KAFAKLAARLYRKALARQLGVAA | NPs | - THP-1, Macrophage - Mice | Inflammatory disorders |  | 2487,14 | 1280 | 65,22 | 12,23 | 7 | 7 | 0 | 36,65 | 30 | [285,286] |
| KAFCPP | KAFAKLAARLYR | Proteins | - THP-1 human monocytes | Inflammatory disorders |  | 1407,77 | 1280 | 66,67 | 11,61 | 5 | 5 | 0,1 | 37,5 | 33 | [287] |
| KALA | WEAKLAKALAKALAKHLAKALAKALKACEA | Nucleic acids | - CV-1, C2C12, K562, UCSF, CaCo-2 |  | Receptor- mediated endocytosis | 3132 | 5810 | 66,67 | 10,47 | 6,02 | 7 | 0,2 | 34,1 | 30 | [288] |
| KFF | KFFKFFKFFK | Nucleic acids | - HeLa |  |  | 1413,81 | 0 | 60 | 11,11 | 5 | 5 | -0,3 | 49 | 40 | [289] |
| KGA | KLAKLAKKLAKLAKGGKKWKMRRNQFWVKVQRG | Proteins | - L02, HUVEC, ECV304, HeLa, MDA-MB-435, MDA-MB-231, HCM, MCF-7, HK-2, A375 | Cancer |  | 3896,05 | 11380 | 42,42 | 12,86 | 14 | 14 | 0,5 | 22,67 | 48 | [158] |
| KH27K | KHHHHHHHHHHHHHHHHHHHHHHHHHHHK | NPs | -MPyV virus | Infectious Diseases |  | 3977,42 | 0 | 0 | 10,72 | 6,02 | 30 | -0,3 | 5,86 | 7 | [228] |
| KLA | KLAKLAKKLAKLAK | Proteins, small drugs | - A549, HeLa, PC3, MDA-MB-231, HIG-82, Clu-3, CHO, 293  - Mice | Cancer, inflammatory disorders, nasal disorders |  | 1524,09 | 0 | 57,14 | 11,44 | 7 | 7 | 0,6 | 30,43 | 43 | [193,260,290] |
| KLA1 | KLALKALKAALKLA |  | - HeLa, Neuron |  |  | 1451,98 | 0 | 71,43 | 11,11 | 5 | 5 | 0 | 43,79 | 29 | [291] |
| Aca, Aminocaproic acid; AEC, Aortic endothelial cells; Ahx, 6-aminohexanoic acid; Aib, 2-aminoisobutyric acid; B-ALL, B acute lymphoblastic leukemia; BBMVEC, Bovine brain microvascular endothelial cells; BCECs, Brain capillary endothelial cells; BHK, Baby hamster kidney; BMB, 3,5-bis(mercaptomethyl)benzoyl; BMDCs, Bone marrow-derived dendritic cells; BMEC, Brain microvascular endothelial cells; Cha, Cyclohexylalanine; CHO, Chinese hamster ovary; Dap, Diaminopimelic acid; DC2.4, Mouse dendritic cells; DMECs, Dermal microvessel endothelial cells; Dmt, *N,N*-Dimethyltryptamine; ES, Embryonic stem cells; F2, Diphenyl; GICs, Glioma-initiating cells; HASMCs, primary human aortic smooth muscle cells; HCE, Human corneal epithelial; HCEC, Human corneal epithelial cells; HCM, Human cardiac myocytes; HEK, Human embryonic kidney; HEL, Human erythroleukemia cell; Hex, Hexyl; hfRPE, Human fetal retinal pigment epithelial cells; hMSCs, Human mesenchymal stem cells; HMVEC, Human lung microvascular endothelial cells; HUVECs, Human umbilical vein endothelial cells; MDCK, Madin-Darby canine kidney; Met, Methylation; Nap, Naphthylalanine; NHC, Human conjunctival epithelial cells; NHDF, Normal human dermal fibroblasts; NPs, Nanoparticles; RAECM, Rat alveolar epithelial cell monolayers; SDHCEC, Spontaneously derived human corneal epithelial cells; Sip, Silaproline; X, Cyclohexylalanine; , Naphthylalanone; - No name attributed; * Different peptides with similar designations; | | | | | | | | | | | | | | | |

**Table S3. Features of published cell-penetrating peptides (cont.).**

| **Peptide** | **Typical sequence** | **Main cargoes** | **Cell model** | **Diseases** | **Proposed translocation** | **Physicochemical properties** | | | | | | | | | **Ref.** |
| --- | --- | --- | --- | --- | --- | --- | --- | --- | --- | --- | --- | --- | --- | --- | --- |
|  |  |  |  |  |  | **Molecular weight**  **(g/mol)** | **Extinction coefficient**  **(M^-1^.cm^-1^)** | **Hydrophobic**  **(%)** | **Isoelectric point** | **Net charge**  **(pH 7)** | **Charge** | **Average hydrophobicity** | **Hydrophobicity**  **(pH 7)** | **Ratio hydrophilic residues**  **(%)** |  |
| KLA2 | KALAALLKKLAKLLAALK |  | - AEC |  |  | 1877,58 | 0 | 72,22 | 11,3 | 6 | 6 | 0 | 46,17 | 28 | [292] |
| KLA3 | KLALKLALKALKAALK |  | - AEC |  |  | 1693,33 | 0 | 68,75 | 11,3 | 6 | 6 | 0,1 | 43,12 | 31 | [292] |
| KLA8 | KLALQLALQALQAALQLA |  | - AEC |  |  | 1877,42 | 0 | 72,22 | 10,28 | 2 | 2 | -0,7 | 49,06 | 28 | [292] |
| KLA9 | QLALQLALQALQAALQLA |  | - AEC |  |  | 1877,38 | 0 | 72,22 | 13,8 | 1 | 1 | -0,8 | 49,78 | 28 | [292] |
| KLA10 | ELALELALEALEAALELA |  | - AEC |  |  | 1882,28 | 0 | 72,22 | 3,6 | -4 | -4 | 0 | 4394 | 28 | [292] |
| KLA11  (Peptide IV) | LKTLATALTKLAKTLTTL |  | - AEC | Cardiovascular Diseases |  | 1901,47 | 0 | 50 | 10,92 | 4 | 4 | -0,3 | 40,67 | 17 | [292,293] |
| KLA13 | LKTLTETLKELTKTLTEL |  | - AEC |  |  | 2075,59 | 0 | 33,33 | 9,8 | 1 | 1 | 0,3 | 28,67 | 33 | [292] |
| KLA15 | RQIKIWFQNRRMKWKK |  | - AEC |  |  | 2246,85 | 11380 | 37,5 | 12,83 | 8 | 8 | 0,5 | 23 | 63 | [292] |
| KLD12 | KLDLKLDLKLDL | NPs | - SK-OV-3 | Cancer |  | 1426,84 | 0 | 50 | 9,8 | 1 | 1 | 0,6 | 30,5 | 50 | [294] |
| Kno | KQINNWFINQRKRHWK |  | - Lipid vesicles |  |  | 2196,65 | 11380 | 31,25 | 12,54 | 6,11 | 7 | 0,2 | 16,06 | 63 | [295] |
| KT2 | NGVQPKYKWWKWWKKWW |  | - HCT116, Vero |  | Clathrin-mediated endocytosis, caveolae-mediated endocytosis | 2434,98 | 35420 | 47,06 | 10,85 | 6 | 6 | -0,5 | 29,94 | 41 | [296] |
| Ku-70 | PMLKE | NPs | - A375, MSTO, rat 9L, U87, LN18 | Cancer | Endocytosis | 616,8 | 0 | 40 | 10,1 | 1 | 1 | 0,6 | 14,8 | 40 | [202] |
| L17E | IWLTALKFLGKHAAKHEAKQQLSKL | Proteins | - HeLa |  | Endocytosis | 2860,6 | 5690 | 48 | 10,79 | 5,22 | 7 | -0,1 | 32,6 | 36 | [297] |
| Lactoferrin (22) | KCFQWQRNVRKVRGPPVSCIKR | NPs | - A549, Beas-2B |  | Clathrin-mediated endocytosis | 2686,35 | 5930 | 36,36 | 12,06 | 7,82 | 8 | 0,3 | 15,27 | 50 | [298] |
| LAH4 (Vectofusin-1) | KKALLALALHHLAHLALHLALALKKAGC | NPs, nucleic acids | - HEK293T, hPBMCs, HSPCs, HepG2, HCT116  -MPyV virus | Infectious Diseases, inflammatory disorders |  | 2882,81 | 120 | 70,37 | 10,64 | 4,35 | 8 | -0,5 | 48,78 | 15 | [228,299-302] |
| LAH4-L1 | KKALLAHALHLLALLALHLAHALKKA |  | - HCT116 | Cancer |  | 2779,67 | 0 | 69,23 | 11,11 | 5,44 | 9 | -0,5 | 48,77 | 15 | [303] |
| Lambda-N | QTRRRERRAEKQAQW | Proteins | - EBTr |  |  | 1999,29 | 5690 | 20 | 12,31 | 5 | 5 | 1,3 | 0,47 | 73 | [156] |
| LH | LHHLLHHLHHLLHH | Small drugs | - MDA-MB-231, HeLa | Cancer |  | 1794,23 | 0 | 42,86 | 13,8 | 1,89 | 9 | -1,1 | 47,43 | 0 | [304] |
| Aca, Aminocaproic acid; AEC, Aortic endothelial cells; Ahx, 6-aminohexanoic acid; Aib, 2-aminoisobutyric acid; B-ALL, B acute lymphoblastic leukemia; BBMVEC, Bovine brain microvascular endothelial cells; BCECs, Brain capillary endothelial cells; BHK, Baby hamster kidney; BMB, 3,5-bis(mercaptomethyl)benzoyl; BMDCs, Bone marrow-derived dendritic cells; BMEC, Brain microvascular endothelial cells; Cha, Cyclohexylalanine; CHO, Chinese hamster ovary; Dap, Diaminopimelic acid; DC2.4, Mouse dendritic cells; DMECs, Dermal microvessel endothelial cells; Dmt, *N,N*-Dimethyltryptamine; ES, Embryonic stem cells; F2, Diphenyl; GICs, Glioma-initiating cells; HASMCs, primary human aortic smooth muscle cells; HCE, Human corneal epithelial; HCEC, Human corneal epithelial cells; HCM, Human cardiac myocytes; HEK, Human embryonic kidney; HEL, Human erythroleukemia cell; Hex, Hexyl; hfRPE, Human fetal retinal pigment epithelial cells; hMSCs, Human mesenchymal stem cells; HMVEC, Human lung microvascular endothelial cells; HUVECs, Human umbilical vein endothelial cells; MDCK, Madin-Darby canine kidney; Met, Methylation; Nap, Naphthylalanine; NHC, Human conjunctival epithelial cells; NHDF, Normal human dermal fibroblasts; NPs, Nanoparticles; RAECM, Rat alveolar epithelial cell monolayers; SDHCEC, Spontaneously derived human corneal epithelial cells; Sip, Silaproline; X, Cyclohexylalanine; , Naphthylalanone; - No name attributed; * Different peptides with similar designations; | | | | | | | | | | | | | | | |

**Table S3. Features of published cell-penetrating peptides (cont.).**

| **Peptide** | **Typical sequence** | **Main cargoes** | **Cell model** | **Diseases** | **Proposed translocation** | **Physicochemical properties** | | | | | | | | | **Ref.** |
| --- | --- | --- | --- | --- | --- | --- | --- | --- | --- | --- | --- | --- | --- | --- | --- |
|  |  |  |  |  |  | **Molecular weight**  **(g/mol)** | **Extinction coefficient**  **(M^-1^.cm^-1^)** | **Hydrophobic**  **(%)** | **Isoelectric point** | **Net charge**  **(pH 7)** | **Charge** | **Average hydrophobicity** | **Hydrophobicity**  **(pH 7)** | **Ratio hydrophilic residues**  **(%)** |  |
| LinTT1 | AKRGARSTA | NPs | - Rats | Cancer |  | 917,06 | 0 | 33,33 | 12,51 | 4 | 4 | 0,8 | 8,89 | 44 | [305] |
| LK | LKKLLKLLKKLLKLAG | Small drugs | - A549, MDA-MB-231, HeLa  - Mice | Cancer, nasal disorders | Endocytosis | 1820,59 | 0 | 56,25 | 11,44 | 7 | 7 | 0,2 | 43,94 | 38 | [304,306] |
| LK-2 | LKKLCKLLKKLCKAG | Small drugs | - HeLa | Ocular disorders |  | 1687,36 | 240 | 53,33 | 10,61 | 6,82 | 7 | 0,4 | 33,4 | 40 | [307] |
| LK-3 | LKKLCKLLKKLCKAGLKKLCKLLKKLCKAG | Small drugs | - HeLa | Ocular disorders |  | 3356,71 | 480 | 53,33 | 10,72 | 12,63 | 13 | 0,4 | 33,4 | 40 | [307] |
| LPIN3 | RRKRRRRRK | Proteins | - Jurkat T - Mice | Cancer | Lipid rafts mediated endocytosis | 1367,7 | 0 | 0 | 13,45 | 10 | 10 | 3 | -16 | 100 | [308] |
| Lp-PPRP | PPRP | NPs | - HeLa, A549, MDA-MB-231 | Cancer |  | 456,56 | 0 | 0 | 11,18 | 2 | 2 | 0,8 | -38 | 25 | [309] |
| LSP-L1 | YQRLC | NPs | - CHO, CCL-61 |  |  | 681,83 | 1400 | 60 | 8,85 | 1,91 | 2 | -0,4 | 37,6 | 40 | [310] |
| LSP-L2 | CNPGY | NPs | - CHO, CCL-61 |  |  | 552,62 | 1400 | 40 | 8,9 | 1,09 | 1 | -0,6 | 5 | 20 | [310] |
| M918 | MVTVLFRRLRIRRASGPPRVRV | Proteins, nucleic acids | - HeLa, CHO  - Lipid vesicles |  | Endocytosis, macropinocytosis | 2636,36 | 0 | 45,45 | 13,45 | 8 | 8 | 0,2 | 28,77 | 36 | [204,311,312] |
| MAP  (PN159) | KLALKLALKALKAALKLA | Proteins, nucleic acids | - Hela, Caco-2, AEC, A549, NIH3T3, SEZ, ECV 304, SK-N-SH, HepG2  - Lipid vesicles | Gastrointestinal disorders | Endocytosis, pore formation, paracellular pathway | 1877,58 | 0 | 72,22 | 11,3 | 6 | 6 | -1 | 46,17 | 28 | [153,204,206,293,313-316] |
| MAP(Aib) | KL(Aib)LKL(Aib)LK(Aib)LKA(Aib)LKL(Aib) | Nucleic acids | - A549, NIH3T3 |  |  | 1947,78 | 0 | 61,54 | 11,3 | 6 | 6 | 0,1 | 48,15 | 38 | [315] |
| MAP-1 | [KLALKALKALKAALKLA]c | Proteins | - HEK293, HeLa - Bacteria | Infectious Diseases |  | 1764,41 | 0 | 70,59 | 11,3 | 6 | 6 | 0,1 | 43 | 29 | [217] |
| sMAP-1 | KLALKALKALKAALKLA | Proteins | - HEK293, HeLa - Bacteria | Infectious Diseases |  | 1764,41 | 0 | 70,59 | 11,3 | 6 | 6 | 0,1 | 43 | 29 | [217] |
| Mca | GDCLPHLKLCKENKDCCSKKCKRRGTNIEKRCR |  | - Mice |  |  | 3864,77 | 720 | 30,3 | 9,54 | 7,57 | 9 | 1 | 5,82 | 55 | [317] |
| D-MCa | GDCLPHLKLCKENKDCCSKKCKRRGTNIEKRCR | Proteins | - U87, SVGp12 | Cancer |  | 3864,77 | 720 | 30,3 | 9,54 | 7,57 | 9 | 1 | 5,82 | 55 | [318] |
| MGPE-9 | CRRLRHLRHHYRRRWHRFRC | Nucleic acids | - ARPE-19, hfRPE | Ocular disorders |  | 2901,52 | 7210 | 35 | 12,6 | 10,26 | 14 | 0,6 | 23,05 | 45 | [186] |
| MitP | INLKKLAKL(Aib)KKIL | Proteins | - Swiss 3T3, Bovine spermatozoa, Human semen | Reproductive disorders |  | 1608,24 | 0 | 53,85 | 11,3 | 6 | 6 | 0,3 | 37,15 | 46 | [174] |
| iMitP | inlkklakl(Aib)kkil | Proteins | - Swiss 3T3, Bovine spermatozoa, Human semen | Reproductive disorders |  | 1608,24 | 0 | 53,85 | 11,3 | 6 | 6 | 0,3 | 37,15 | 46 | [174] |
| Aca, Aminocaproic acid; AEC, Aortic endothelial cells; Ahx, 6-aminohexanoic acid; Aib, 2-aminoisobutyric acid; B-ALL, B acute lymphoblastic leukemia; BBMVEC, Bovine brain microvascular endothelial cells; BCECs, Brain capillary endothelial cells; BHK, Baby hamster kidney; BMB, 3,5-bis(mercaptomethyl)benzoyl; BMDCs, Bone marrow-derived dendritic cells; BMEC, Brain microvascular endothelial cells; Cha, Cyclohexylalanine; CHO, Chinese hamster ovary; Dap, Diaminopimelic acid; DC2.4, Mouse dendritic cells; DMECs, Dermal microvessel endothelial cells; Dmt, *N,N*-Dimethyltryptamine; ES, Embryonic stem cells; F2, Diphenyl; GICs, Glioma-initiating cells; HASMCs, primary human aortic smooth muscle cells; HCE, Human corneal epithelial; HCEC, Human corneal epithelial cells; HCM, Human cardiac myocytes; HEK, Human embryonic kidney; HEL, Human erythroleukemia cell; Hex, Hexyl; hfRPE, Human fetal retinal pigment epithelial cells; hMSCs, Human mesenchymal stem cells; HMVEC, Human lung microvascular endothelial cells; HUVECs, Human umbilical vein endothelial cells; MDCK, Madin-Darby canine kidney; Met, Methylation; Nap, Naphthylalanine; NHC, Human conjunctival epithelial cells; NHDF, Normal human dermal fibroblasts; NPs, Nanoparticles; RAECM, Rat alveolar epithelial cell monolayers; SDHCEC, Spontaneously derived human corneal epithelial cells; Sip, Silaproline; X, Cyclohexylalanine; , Naphthylalanone; - No name attributed; * Different peptides with similar designations; | | | | | | | | | | | | | | | |

**Table S3. Features of published cell-penetrating peptides (cont.).**

| **Peptide** | **Typical sequence** | **Main cargoes** | **Cell model** | **Diseases** | **Proposed translocation** | **Physicochemical properties** | | | | | | | | | **Ref.** |
| --- | --- | --- | --- | --- | --- | --- | --- | --- | --- | --- | --- | --- | --- | --- | --- |
|  |  |  |  |  |  | **Molecular weight**  **(g/mol)** | **Extinction coefficient**  **(M^-1^.cm^-1^)** | **Hydrophobic**  **(%)** | **Isoelectric point** | **Net charge**  **(pH 7)** | **Charge** | **Average hydrophobicity** | **Hydrophobicity**  **(pH 7)** | **Ratio hydrophilic residues**  **(%)** |  |
| iMP | Inlkalaalakkil | Proteins | - Swiss 3T3, Bovine spermatozoa, Human semen | Reproductive disorders |  | 1480 | 0 | 71,43 | 10,92 | 4 | 4 | -0,3 | 46,57 | 29 | [174] |
| MPG | GALFLGFLGAAGSTMGAWSQPKKKRKV | Proteins, nucleic acids | - AN3CA, HeLa, HS68, NIH3T3, bv2, HepG2, Cos-7, PC3, MCF-7, SCK3, HEK-293T  - Lipid vesicles | Cancer | Endocytosis | 2807,5 | 5690 | 44,44 | 11,91 | 6 | 6 | -0,1 | 27,63 | 30 | [168,204,210,319-324] |
| MPG-8 | AFLGWLGAWGTMGWSPKKKRK | Nucleic acids | - Hs68, HeLa, PC3, MCF-7, SCK3 |  |  | 2406,01 | 17070 | 42,86 | 11,91 | 6 | 6 | -0,2 | 28,57 | 29 | [319] |
| MPG-a | GALFLAFLAAALSLMGLWSQPKKKRKV |  | - Lipid vesicles |  |  | 2945,8 | 5690 | 59,26 | 11,91 | 6 | 6 | -0,3 | 39,78 | 30 | [204] |
| msr(W/ R) | RRWWRRWRR |  | - CHO-K1 |  | Direct internalization | 1513,81 | 17070 | 33,33 | 13,33 | 7 | 7 | 0,9 | 23 | 67 | [325] |
| MT20 | GIGAVLKVLTTGLPALISWI | Nucleic acids | - 293FT, B16F10, CHO-K1 |  |  | 2022,64 | 5690 | 60 | 10,28 | 2 | 2 | -0,9 | 49 | 10 | [226] |
| MTat2 | AKKRRQRRRAKKRRQRRR | NPs | - HeLa, HCE - Fungus | Infectious Diseases, ocular disorders | Endocytosis | 2491,07 | 0 | 11,11 | 13,66 | 15 | 15 | 2,3 | -9,44 | 89 | [326] |
| mtCPP-1 | r(Dmt)(Orn)F |  | - HeLa, U87, bEnd.3 |  |  | 621,75 | 5690 | 66,67 | 11,18 | 2 | 2 | 0 | 60 | 50 | [327] |
| mtCPP-8 | r(Dmt)RF |  | - HeLa, U87, bEnd.3 |  |  | 663,78 | 5690 | 50 | 12,49 | 3 | 3 | 0 | 41,5 | 50 | [327] |
| mtCPP-9 | r(Dmt)rF |  | - HeLa, U87, bEnd.3 |  |  | 663,78 | 5690 | 50 | 12,49 | 3 | 3 | 0 | 41,5 | 50 | [327] |
| MTD103 | LALPVLLLA | Proteins | - RAW 264.7, HCT116 - Mice | Cancer |  | 922,28 | 0 | 88,89 | 13,8 | 1 | 1 | -1,3 | 68 | 0 | [253] |
| MTS  (KFGF) | AAVLLPVLLAAP | Proteins, NPs | - A431, SK-BR-3, RAW 264.7, HCT116, HEK293, HeLa, A375, MSTO, rat 9L, U87, LN18  - Bacteria  - Mice | Cancer, infectious Diseases, inflammatory disorders | Endocytosis | 1147,53 | 0 | 83,33 | 13,8 | 1 | 1 | -1 | 52 | 0 | [202,217,253,290,328] |
| MTS-1 | IEGRGIAAVLLPVLLAAPGIPGNSS | Proteins | - NIH 3T3, SAA, BHK-1, LE-II - Mice |  |  | 2385,98 | 0 | 52 | 11,1 | 1 | 1 | -0,4 | 31,16 | 20 | [329] |
| MTS-2 | IEGRGIPAAVLLPVLLAAP | Proteins | - NIH 3T3, SAA, BHK-1, LE-II - Mice |  |  | 1870,42 | 0 | 63,16 | 9 | 1 | 1 | -0,5 | 38,47 | 11 | [329] |
| NBP | CAKVKDEPQRRSARLSAKPAPPKPEPKPKKAPAKK | Proteins, nucleic acids | - Mice | Ocular disorders |  | 3834,72 | 120 | 25,71 | 11,1 | 9,9 | 10 | 1,1 | -8,09 | 51 | [330] |
| Aca, Aminocaproic acid; AEC, Aortic endothelial cells; Ahx, 6-aminohexanoic acid; Aib, 2-aminoisobutyric acid; B-ALL, B acute lymphoblastic leukemia; BBMVEC, Bovine brain microvascular endothelial cells; BCECs, Brain capillary endothelial cells; BHK, Baby hamster kidney; BMB, 3,5-bis(mercaptomethyl)benzoyl; BMDCs, Bone marrow-derived dendritic cells; BMEC, Brain microvascular endothelial cells; Cha, Cyclohexylalanine; CHO, Chinese hamster ovary; Dap, Diaminopimelic acid; DC2.4, Mouse dendritic cells; DMECs, Dermal microvessel endothelial cells; Dmt, *N,N*-Dimethyltryptamine; ES, Embryonic stem cells; F2, Diphenyl; GICs, Glioma-initiating cells; HASMCs, primary human aortic smooth muscle cells; HCE, Human corneal epithelial; HCEC, Human corneal epithelial cells; HCM, Human cardiac myocytes; HEK, Human embryonic kidney; HEL, Human erythroleukemia cell; Hex, Hexyl; hfRPE, Human fetal retinal pigment epithelial cells; hMSCs, Human mesenchymal stem cells; HMVEC, Human lung microvascular endothelial cells; HUVECs, Human umbilical vein endothelial cells; MDCK, Madin-Darby canine kidney; Met, Methylation; Nap, Naphthylalanine; NHC, Human conjunctival epithelial cells; NHDF, Normal human dermal fibroblasts; NPs, Nanoparticles; RAECM, Rat alveolar epithelial cell monolayers; SDHCEC, Spontaneously derived human corneal epithelial cells; Sip, Silaproline; X, Cyclohexylalanine; , Naphthylalanone; - No name attributed; * Different peptides with similar designations; | | | | | | | | | | | | | | | |

**Table S3. Features of published cell-penetrating peptides (cont.).**

| **Peptide** | **Typical sequence** | **Main cargoes** | **Cell model** | **Diseases** | **Proposed translocation** | **Physicochemical properties** | | | | | | | | | **Ref.** |
| --- | --- | --- | --- | --- | --- | --- | --- | --- | --- | --- | --- | --- | --- | --- | --- |
|  |  |  |  |  |  | **Molecular weight**  **(g/mol)** | **Extinction coefficient**  **(M^-1^.cm^-1^)** | **Hydrophobic**  **(%)** | **Isoelectric point** | **Net charge**  **(pH 7)** | **Charge** | **Average hydrophobicity** | **Hydrophobicity**  **(pH 7)** | **Ratio hydrophilic residues**  **(%)** |  |
| NF-B | VQRKRQKLMP |  | - MCF-7, HT29, PC3, KB, MIAOACA2 |  | Endocytosis | 1283,66 | 0 | 30 | 12,53 | 5 | 5 | 0,8 | 11 | 60 | [331] |
| NGR | CNGRCG | Proteins, NPs, small drugs | - TS/A, B16F1, WEHI-164, DMECs, GI-ME-N, HUVEC, SLK, HT-1080, GI-LI-N, HTLA-230, IMR-32, SH-SY5Y, KS1767, NXS2, Mouse RMA-T lymphoma cells,  - Mice  - Humans (Phase I) | Cancer |  | 608,71 | 240 | 33,33 | 7,99 | 1,82 | 2 | 0,2 | 7,17 | 33 | [273,332-340] |
| NLS motif | RRMKWKK | Protein | - MIN6 |  |  | 1032,35 | 5690 | 28,57 | 12,54 | 6 | 6 | 1,5 | 10,57 | 71 | [341] |
| NrTP1 | YKQCHKKGGKKGSG |  | - Lipid vesicles |  |  | 1505,84 | 1400 | 14,29 | 10,55 | 6,02 | 7 | 0,8 | -0,71 | 50 | [194] |
| NrTP6 | YKQSHKKGGKKGSG | Proteins | - HeLa, BHK21 |  |  | 1489,78 | 1280 | 7,14 | 10,85 | 6,11 | 7 | 0,9 | -4,57 | 57 | [342,343] |
| NrTP8 | WKQSHKKGGKKGSG |  | - Lipid vesicles |  |  | 1512,82 | 5690 | 7,14 | 11,3 | 6,11 | 7 | 0,9 | -2,14 | 57 | [194] |
| Nucleoplasmin | KRPAATKKAGQAKKKL | Proteins, nucleic acids | - PC-3M - Mice | Cancer |  | 1724,2 | 0 | 31,25 | 11,98 | 8 | 8 | 1,1 | 4,31 | 50 | [344] |
| OCT-6 | GRKRKKRT |  | - MCF-7, HT29, PC3, KB, MIAOACA2 |  | Endocytosis | 1029,29 | 0 | 0 | 12,83 | 7 | 7 | 2,2 | -12,25 | 75 | [331] |
| Glu-Oct6 | EEEAAGRKRKKRT | Nucleic acids | - DU-145, LNCaP, PANC-1 | Cancer |  | 1558,81 | 0 | 15,38 | 10,88 | 4 | 4 | 2 | -8,38 | 69 | [345] |
| oxmCPP | CRRRRRRRR | Small drugs | - MCF-7, HT-29 | Cancer |  | 1370,67 | 120 | 11,11 | 12,97 | 8,91 | 9 | 2,6 | -7 | 89 | [346] |
| oxmCPPamph | CGGWVELPPPVELPPPVELPPP | Small drugs | - MCF-7, HT-29 | Cancer |  | 2319,86 | 5810 | 36,36 | 2,93 | -2,09 | -2 | -0,2 | 7,59 | 14 | [346] |
| (PRR)4 | PRRPRRPRRPRR | Proteins | - HeLa |  |  | 1655,03 | 0 | 0 | 13,53 | 8 | 9 | 2 | -24,67 | 67 | [347] |
| (PRR)5 | PRRPRRPRRPRRPRR | Proteins | - HeLa |  |  | 2065,51 | 0 | 0 | 13,66 | 10 | 11 | 2 | -24,67 | 67 | [347] |
| (PRR)6 | PRRPRRPRRPRRPRRPRR | Proteins | - HeLa |  |  | 2475,01 | 0 | 0 | 13,74 | 12 | 12 | 2 | -24,67 | 67 | [347] |
| P0084 | RKQKSLQTKLAENPPVPRKKRQSRPRWKQWLQK | Proteins, nucleic acids | - HEK-293, A431, CHO-K1 |  | RMT | 4126,07 | 11380 | 21,21 | 12,71 | 12 | 12 | 0,8 | 2,33 | 64 | [348] |
| P1 | FFFF | Small drug | - A549, HepG2, GM5657T | Cancer |  | 606,73 | 0 | 100 | 13,8 | 1 | 1 | -2,5 | 97 | 0 | [349] |
| P1 (pal-12) | RCLSSSAVANRS |  | - Mice | Cardiovascular Diseases |  | 1250,43 | 120 | 41,67 | 11,05 | 2,91 | 3 | 0,2 | 18,17 | 58 | [350] |
| Aca, Aminocaproic acid; AEC, Aortic endothelial cells; Ahx, 6-aminohexanoic acid; Aib, 2-aminoisobutyric acid; B-ALL, B acute lymphoblastic leukemia; BBMVEC, Bovine brain microvascular endothelial cells; BCECs, Brain capillary endothelial cells; BHK, Baby hamster kidney; BMB, 3,5-bis(mercaptomethyl)benzoyl; BMDCs, Bone marrow-derived dendritic cells; BMEC, Brain microvascular endothelial cells; Cha, Cyclohexylalanine; CHO, Chinese hamster ovary; Dap, Diaminopimelic acid; DC2.4, Mouse dendritic cells; DMECs, Dermal microvessel endothelial cells; Dmt, *N,N*-Dimethyltryptamine; ES, Embryonic stem cells; F2, Diphenyl; GICs, Glioma-initiating cells; HASMCs, primary human aortic smooth muscle cells; HCE, Human corneal epithelial; HCEC, Human corneal epithelial cells; HCM, Human cardiac myocytes; HEK, Human embryonic kidney; HEL, Human erythroleukemia cell; Hex, Hexyl; hfRPE, Human fetal retinal pigment epithelial cells; hMSCs, Human mesenchymal stem cells; HMVEC, Human lung microvascular endothelial cells; HUVECs, Human umbilical vein endothelial cells; MDCK, Madin-Darby canine kidney; Met, Methylation; Nap, Naphthylalanine; NHC, Human conjunctival epithelial cells; NHDF, Normal human dermal fibroblasts; NPs, Nanoparticles; RAECM, Rat alveolar epithelial cell monolayers; SDHCEC, Spontaneously derived human corneal epithelial cells; Sip, Silaproline; X, Cyclohexylalanine; , Naphthylalanone; - No name attributed; * Different peptides with similar designations; | | | | | | | | | | | | | | | |

**Table S3. Features of published cell-penetrating peptides (cont.).**

| **Peptide** | **Typical sequence** | **Main cargoes** | **Cell model** | **Diseases** | **Proposed translocation** | **Physicochemical properties** | | | | | | | | | **Ref.** |
| --- | --- | --- | --- | --- | --- | --- | --- | --- | --- | --- | --- | --- | --- | --- | --- |
|  |  |  |  |  |  | **Molecular weight**  **(g/mol)** | **Extinction coefficient**  **(M^-1^.cm^-1^)** | **Hydrophobic**  **(%)** | **Isoelectric point** | **Net charge**  **(pH 7)** | **Charge** | **Average hydrophobicity** | **Hydrophobicity**  **(pH 7)** | **Ratio hydrophilic residues**  **(%)** |  |
| P2 | RRRRRRRRRGAL | Small drug | - A549, HepG2, GM5657T | Cancer |  | 1665,03 | 0 | 16,67 | 13,6 | 10 | 10 | 2,1 | 1,25 | 75 | [349] |
| P3 | FFFFRRRRRRRRRGAL | Small drug | - A549, HepG2, GM5657T | Cancer |  | 2253,75 | 0 | 37,5 | 13,6 | 10 | 10 | 0,9 | 25,19 | 56 | [349] |
| P4 | LGAQSNF | Nucleic acids | - Myoblasts - Mice | Cardiovascular Diseases |  | 735,83 | 0 | 42,86 | 13,8 | 1 | 1 | -0,6 | 26 | 43 | [351] |
| P4 (pal-10) | SGRRYGHALR |  | - Mice | Cardiovascular Diseases |  | 1172,36 | 1280 | 30 | 12,18 | 4,11 | 5 | 0,2 | 16,5 | 40 | [350] |
| P7 | GLRRALLRLLRSLRRLLLRA |  | - Bacteria - Mice | Infectious Diseases |  | 2416,17 | 0 | 55 | 13,45 | 8 | 8 | 0,2 | 43,95 | 40 | [352] |
| P11LRR | PPPPPPPPPPLRR |  | - MCF-7 | Cancer |  | 1414,76 | 0 | 7,69 | 12,49 | 3 | 3 | 0,3 | -29,85 | 15 | [353] |
| P14LRR | PPPPLRR | Small drugs | - Bacteria | Infectious Diseases |  | 832,04 | 0 | 14,29 | 12,49 | 3 | 3 | 0,6 | -16 | 29 | [354] |
| P14SH | PPPPC | Small drugs | - Bacteria | Infectious Diseases |  | 509,63 | 120 | 20 | 8 | 1,09 | 1 | -0,2 | -27 | 0 | [354] |
| P16 | RKRRKR | proteins | - HeLa-S3, 293TT, 293FT, HaCaT - Mice | Infectious Diseases |  | 899,13 | 0 | 0 | 12,97 | 7 | 7 | 3 | -17 | 100 | [355] |
| p18 | LSTAADMQGVVTDGMASG |  | - A549, NCI-H23, CCD-13Lu, DU145, LN-CAP, CRL11611, MCF-7, MCF-10A, HCT116, CCD33Co, HT1080, SKOV3 | Cancer |  | 1710,98 | 0 | 44,44 | 2,92 | -1 | -1 | -0,2 | 23,28 | 28 | [356] |
| P22 | NAKTRRHERRRKLAIER | Proteins, NPs, nucleic acids, small drugs | - Lipid vesicles |  |  | 2190,62 | 0 | 23,53 | 12,5 | 7,11 | 8 | 1,5 | 4,06 | 65 | [162] |
| p28 | LSTAADMQGVVTDGMASGLDKDYLKPDD | Proteins, NPs, nucleic acids, small drugs | - A549, H69AR, NCI-H23, CCD-13Lu, CRL-1611, HK-2, HepG2, THLE-2, DU145, LNCaP, PC-3, CRL1161, MCF-7, MDA-MB-231, T47D, ZR-75-1, MCF-10A, BE, Colo205, HCT116, HT29, SW620, W1Dr, CCD33Co, CCF-STTG1, U87, LN229, IMR-32, SK-N-BE, HT1080, RD, TE85, HTB88, ES-2, CAOV-3, SKOV3, PA-1, BLD-1, BCA1, BCA2, CCa9, ccA12, es3, uiso-gct-1, uiso-pr-1, mmd2, hose6-3, uiso-mEL-2, LN-CAP, CRL11611, HVECs, Human fibroblasts, MCF-7, T47D, HTB-88, TE85, LN229, MIA-Paca2  - Humans (Phase I) | Cancer | Caveolae-mediated endocytosis | 2914,34 | 1280 | 39,29 | 3,5 | -3 | -3 | 0,3 | 13,21 | 39 | [210,356-362] |
| Aca, Aminocaproic acid; AEC, Aortic endothelial cells; Ahx, 6-aminohexanoic acid; Aib, 2-aminoisobutyric acid; B-ALL, B acute lymphoblastic leukemia; BBMVEC, Bovine brain microvascular endothelial cells; BCECs, Brain capillary endothelial cells; BHK, Baby hamster kidney; BMB, 3,5-bis(mercaptomethyl)benzoyl; BMDCs, Bone marrow-derived dendritic cells; BMEC, Brain microvascular endothelial cells; Cha, Cyclohexylalanine; CHO, Chinese hamster ovary; Dap, Diaminopimelic acid; DC2.4, Mouse dendritic cells; DMECs, Dermal microvessel endothelial cells; Dmt, *N,N*-Dimethyltryptamine; ES, Embryonic stem cells; F2, Diphenyl; GICs, Glioma-initiating cells; HASMCs, primary human aortic smooth muscle cells; HCE, Human corneal epithelial; HCEC, Human corneal epithelial cells; HCM, Human cardiac myocytes; HEK, Human embryonic kidney; HEL, Human erythroleukemia cell; Hex, Hexyl; hfRPE, Human fetal retinal pigment epithelial cells; hMSCs, Human mesenchymal stem cells; HMVEC, Human lung microvascular endothelial cells; HUVECs, Human umbilical vein endothelial cells; MDCK, Madin-Darby canine kidney; Met, Methylation; Nap, Naphthylalanine; NHC, Human conjunctival epithelial cells; NHDF, Normal human dermal fibroblasts; NPs, Nanoparticles; RAECM, Rat alveolar epithelial cell monolayers; SDHCEC, Spontaneously derived human corneal epithelial cells; Sip, Silaproline; X, Cyclohexylalanine; , Naphthylalanone; - No name attributed; * Different peptides with similar designations; | | | | | | | | | | | | | | | |

**Table S3. Features of published cell-penetrating peptides (cont.).**

| **Peptide** | **Typical sequence** | **Main cargoes** | **Cell model** | **Diseases** | **Proposed translocation** | **Physicochemical properties** | | | | | | | | | **Ref.** |
| --- | --- | --- | --- | --- | --- | --- | --- | --- | --- | --- | --- | --- | --- | --- | --- |
|  |  |  |  |  |  | **Molecular weight**  **(g/mol)** | **Extinction coefficient**  **(M^-1^.cm^-1^)** | **Hydrophobic**  **(%)** | **Isoelectric point** | **Net charge**  **(pH 7)** | **Charge** | **Average hydrophobicity** | **Hydrophobicity**  **(pH 7)** | **Ratio hydrophilic residues**  **(%)** |  |
| P31 | KRRRKR | Proteins | - HeLa-S3, 293TT, 293FT, HaCaT - Mice | Infectious Diseases |  | 899,13 | 0 | 0 | 12,97 | 7 | 7 | 3 | -17 | 100 | [355] |
| P1267 | PKQPPKPKKPKTQEKKKKQPAKPKPGKRQRMALKLEADRLFDVKNEDGDVIGHALDMKA | Proteins, nucleic acids | - HEK-293, A431, CHO-K1 |  | RMT | 6714,27 | 0 | 25,42 | 10,74 | 11,11 | 12 | 1 | -1,39 | 53 | [348] |
| P1746 | PLKPKKPKTQEKKKKQPPKPKKPKTQEKKKKQPPKPKR | Proteins, nucleic acids | - HEK-293, A431, CHO-K1 |  | RMT | 4538,83 | 0 | 2,63 | 11,62 | 18 | 18 | 1,6 | -22,74 | 66 | [348] |
| P1746c27 | KKKKQPPKPKKPKTQEKKKKQPPKPKR | Proteins, nucleic acids | - HEK-293, A431, CHO-K1 |  | RMT | 3263,21 | 0 | 0 | 11,77 | 15 | 15 | 1,8 | -26,15 | 70 | [348] |
| P1869 | PPHPRPLPAPAQSRKKQKGRAGRGHEKTGASVLRGPQKPHPLPAQLR | Proteins, nucleic acids | - HEK-293, A431, CHO-K1 |  | RMT | 5088,17 | 0 | 21,28 | 12,82 | 11,33 | 13 | 0,5 | -0,47 | 38 | [348] |
| PA | PPPPRRRR | NPs | - MCF-7 - Lipid vesicles |  |  | 1031,25 | 0 | 0 | 12,97 | 5 | 5 | 1,5 | -30 | 50 | [363] |
| PAF102 | GHRKKWFWAGPARRKKWFWAGPAWRKKWFW | Proteins | - Fungus | Infectious Diseases |  | 3973,82 | 39830 | 46,67 | 12,98 | 11,11 | 12 | -0,1 | 28,53 | 33 | [364] |
| PCM | WLSEAGPVVTVRALRGTGSW | NPs | - Rat MCs - Mice | Cardiovascular Diseases |  | 2142,55 | 11380 | 45 | 11,04 | 2 | 2 | -0,4 | 30,75 | 25 | [365] |
| PD3 | PPLSQETFSDLWKLLRKKRRRESRKKRRRES | Proteins | - L02, HUVEC, ECV304, HeLa, MDA-MB-435, MDA-MB-231, HCM, MCF-7, HK-2, A375 | Cancer |  | 3968,77 | 5690 | 19,35 | 12,28 | 10 | 10 | 1,3 | 3,55 | 71 | [158] |
| PDX-1 | RHIKIWFQNRRMKWKK | Proteins | - HeLa |  | Endocytosis | 2255,86 | 11380 | 37,5 | 12,83 | 8,11 | 9 | 0,4 | 24,12 | 56 | [366] |
| Penetratin | RQIKIWFQNRRMKWKK | Proteins, NPs, nucleic acids, small drugs | - A375, AEC, ARPE-19, Astrocytes, B16F10, BAEC, bEnd.3, BHK21, Bowes, C6Bu1, Caco-2, CCL-61, SAOS-2, CT26, Dendritic cells, ECV304, EOMA, HaCaT, HBCEC, HCE, HCM, HepG2, Huh7, Hig-82, HK-2, h-TERT-BJ1, HUVEC, HT1080, HT29, K549, K562, L02, L929, MC57, MDA-MB-435, MCF-7, US-OS, SKLU-1, H4IIE, BNL, RT101, MDCK, MSCs, Neuron, PC3M, RBL-2H3, SDHCEC, SK-BR3, U-87, MDA-MB-231, PC12, T-36274, HeLa, KLN 205, Jurkat –T, P388D.1, RAW264.7, COS-1  - Lipid vesicles  - Drosophila, Virus  - Rats, Rabbits, Mini Pigs  - Humans (Phase I) | Cancer, infectious Diseases, metabolic disorders, ocular disorders, reproductive disorders | Endocytosis, macropinocytosis, clathrin-mediated endocytosis, caveolae-mediated endocytosis, | 2246,85 | 11380 | 37,5 | 12,83 | 8 | 8 | 0,5 | 23 | 63 | [151,153,158,168,173,201,204,206,211,214,218,219,247,250-253,289-291,295,301,310-312,320,344,367-423] |
| Aca, Aminocaproic acid; AEC, Aortic endothelial cells; Ahx, 6-aminohexanoic acid; Aib, 2-aminoisobutyric acid; B-ALL, B acute lymphoblastic leukemia; BBMVEC, Bovine brain microvascular endothelial cells; BCECs, Brain capillary endothelial cells; BHK, Baby hamster kidney; BMB, 3,5-bis(mercaptomethyl)benzoyl; BMDCs, Bone marrow-derived dendritic cells; BMEC, Brain microvascular endothelial cells; Cha, Cyclohexylalanine; CHO, Chinese hamster ovary; Dap, Diaminopimelic acid; DC2.4, Mouse dendritic cells; DMECs, Dermal microvessel endothelial cells; Dmt, *N,N*-Dimethyltryptamine; ES, Embryonic stem cells; F2, Diphenyl; GICs, Glioma-initiating cells; HASMCs, primary human aortic smooth muscle cells; HCE, Human corneal epithelial; HCEC, Human corneal epithelial cells; HCM, Human cardiac myocytes; HEK, Human embryonic kidney; HEL, Human erythroleukemia cell; Hex, Hexyl; hfRPE, Human fetal retinal pigment epithelial cells; hMSCs, Human mesenchymal stem cells; HMVEC, Human lung microvascular endothelial cells; HUVECs, Human umbilical vein endothelial cells; MDCK, Madin-Darby canine kidney; Met, Methylation; Nap, Naphthylalanine; NHC, Human conjunctival epithelial cells; NHDF, Normal human dermal fibroblasts; NPs, Nanoparticles; RAECM, Rat alveolar epithelial cell monolayers; SDHCEC, Spontaneously derived human corneal epithelial cells; Sip, Silaproline; X, Cyclohexylalanine; , Naphthylalanone; - No name attributed; * Different peptides with similar designations; | | | | | | | | | | | | | | | |

**Table S3. Features of published cell-penetrating peptides (cont.).**

| **Peptide** | **Typical sequence** | **Main cargoes** | **Cell model** | **Diseases** | **Proposed translocation** | **Physicochemical properties** | | | | | | | | | **Ref.** |
| --- | --- | --- | --- | --- | --- | --- | --- | --- | --- | --- | --- | --- | --- | --- | --- |
|  |  |  |  |  |  | **Molecular weight**  **(g/mol)** | **Extinction coefficient**  **(M^-1^.cm^-1^)** | **Hydrophobic**  **(%)** | **Isoelectric point** | **Net charge**  **(pH 7)** | **Charge** | **Average hydrophobicity** | **Hydrophobicity**  **(pH 7)** | **Ratio hydrophilic residues**  **(%)** |  |
| D-Penetratin | RQIKIWFQNRRMKWKK | Proteins, NPs | - Rats, Mice | Metabolic disorders |  | 2246,85 | 11380 | 37,5 | 12,83 | 8 | 8 | 0,5 | 23 | 63 | [219,371,376,380,397,410] |
| Penetratin 2W2F | RQIKIFFQNRRMKFKK | NPs | - Lipid vesicles |  |  | 2168,77 | 0 | 37,5 | 12,83 | 0,6 | 8 | 8 | 23 | 63 | [387] |
| Penetratin  289-W | RWIKIWFWWRRMKWKK | Proteins | - HCEC, NHC - Mice | Ocular disorders |  | 2435,12 | 28450 | 56,25 | 12,83 | 8 | 8 | -0,2 | 45 | 44 | [374] |
| Penetratin  28-W | RWIKIWFWNRRMKWKK | Proteins | - HCEC, NHC - Mice | Ocular disorders |  | 2363,01 | 22760 | 50 | 12,83 | 8 | 8 | 0 | 36,38 | 50 | [374] |
| Penetratin  2-W | RWIKIWFQNRRMKWKK | Proteins | - HCEC, NHC - Mice | Ocular disorders |  | 2304,93 | 17070 | 43,75 | 12,83 | 8 | 8 | 0,2 | 29,69 | 56 | [374] |
| Penetratin Sample5 | KWFKIQMQIRRWKNRK | Proteins | - Rats | Metabolic disorders |  | 2246,85 | 11380 | 37,5 | 12,83 | 8 | 8 | 0,5 | 23 | 63 | [401] |
| Penetratin Sample6 | KWFKIQMQIRRWKNKR | Proteins | - Rats | Metabolic disorders |  | 2246,85 | 11380 | 37,5 | 12,83 | 8 | 8 | 0,5 | 23 | 63 | [401] |
| Penetratin Shuffle  (RK fix)-2 | RWFKIQMQIRRWKNKK | Proteins | - Rats | Metabolic disorders |  | 2246,85 | 11380 | 37,5 | 12,83 | 8 | 8 | 0,5 | 23 | 63 | [401,402] |
| Penetratin (Arg) | RQIRIWFQNRRMRWRR | Nucleic acid | - HeLa - Lipid vesicles |  | Endocytosis | 2358,89 | 11380 | 37,5 | 13,45 | 8 | 8 | 0,5 | 25,25 | 63 | [312] |
| PenetraMax | KWFKIQMQIRRWKNKR | proteins | - Mice | Metabolic disorders |  | 2246,85 | 11380 | 37,5 | 12,83 | 8 | 8 | 0,5 | 23 | 63 | [380] |
| D-PenetraMax | Kwfkiqmqirrwknkr | proteins | - Mice | Metabolic disorders |  | 2246,85 | 11380 | 37,5 | 12,83 | 8 | 8 | 0,5 | 23 | 63 | [380] |
| Pep 2a | (cha)r(cha)K | Small drugs | - H9c2, A2780 - Mice |  | Endocytosis | 608,82 | 0 | 0 | 11,6 | 3 | 3 |  | -18,5 |  | [424] |
| Pep 2b | FrFK | Small drugs | - H9c2, A2780 - Mice |  | Endocytosis | 596,74 | 0 | 50 | 11,6 | 3 | 3 | 0,3 | 39,25 | 50 | [424] |
| Pep 2c | Fr(cha)K | Small drugs | - H9c2, A2780 - Mice |  | Endocytosis | 602,78 | 0 | 33,33 | 11,6 | 3 | 3 |  | 20 |  | [424] |
| Pep 2d | FrFK | Small drugs | - H9c2, A2780 - Mice |  | Endocytosis | 596,74 | 0 | 50 | 11,6 | 3 | 3 | 0,3 | 39,25 | 50 | [424] |
| Pep 2e | Fr(Nap)K | Small drugs | - H9c2, A2780 - Mice |  | Endocytosis | 577,74 | 0 | 25 | 11,77 | 4 | 4 | 1,6 | 9,25 | 75 | [424] |
| Pep 2f | Fr(Hex)K | Small drugs | - H9c2, A2780 - Mice |  | Endocytosis | 506,62 | 0 | 25 | 11,6 | 3 | 3 | 0,9 | 15 | 50 | [424] |
| Pep 2g | FrY(Met)K | Small drugs | - H9c2, A2780 - Mice |  | Endocytosis | 612,74 | 1280 | 50 | 10,58 | 3 | 3 | 0,3 | 30,75 | 50 | [424] |
| Pep 2h | Fr(cha)K | Small drugs | - H9c2, A2780 - Mice |  | Endocytosis | 602,78 | 0 | 33,33 | 11,6 | 3 | 3 |  | 20 |  | [424] |
| Aca, Aminocaproic acid; AEC, Aortic endothelial cells; Ahx, 6-aminohexanoic acid; Aib, 2-aminoisobutyric acid; B-ALL, B acute lymphoblastic leukemia; BBMVEC, Bovine brain microvascular endothelial cells; BCECs, Brain capillary endothelial cells; BHK, Baby hamster kidney; BMB, 3,5-bis(mercaptomethyl)benzoyl; BMDCs, Bone marrow-derived dendritic cells; BMEC, Brain microvascular endothelial cells; Cha, Cyclohexylalanine; CHO, Chinese hamster ovary; Dap, Diaminopimelic acid; DC2.4, Mouse dendritic cells; DMECs, Dermal microvessel endothelial cells; Dmt, *N,N*-Dimethyltryptamine; ES, Embryonic stem cells; F2, Diphenyl; GICs, Glioma-initiating cells; HASMCs, primary human aortic smooth muscle cells; HCE, Human corneal epithelial; HCEC, Human corneal epithelial cells; HCM, Human cardiac myocytes; HEK, Human embryonic kidney; HEL, Human erythroleukemia cell; Hex, Hexyl; hfRPE, Human fetal retinal pigment epithelial cells; hMSCs, Human mesenchymal stem cells; HMVEC, Human lung microvascular endothelial cells; HUVECs, Human umbilical vein endothelial cells; MDCK, Madin-Darby canine kidney; Met, Methylation; Nap, Naphthylalanine; NHC, Human conjunctival epithelial cells; NHDF, Normal human dermal fibroblasts; NPs, Nanoparticles; RAECM, Rat alveolar epithelial cell monolayers; SDHCEC, Spontaneously derived human corneal epithelial cells; Sip, Silaproline; X, Cyclohexylalanine; , Naphthylalanone; - No name attributed; * Different peptides with similar designations; | | | | | | | | | | | | | | | |

**Table S3. Features of published cell-penetrating peptides (cont.).**

| **Peptide** | **Typical sequence** | **Main cargoes** | **Cell model** | **Diseases** | **Proposed translocation** | **Physicochemical properties** | | | | | | | | | **Ref.** |
| --- | --- | --- | --- | --- | --- | --- | --- | --- | --- | --- | --- | --- | --- | --- | --- |
|  |  |  |  |  |  | **Molecular weight**  **(g/mol)** | **Extinction coefficient**  **(M^-1^.cm^-1^)** | **Hydrophobic**  **(%)** | **Isoelectric point** | **Net charge**  **(pH 7)** | **Charge** | **Average hydrophobicity** | **Hydrophobicity**  **(pH 7)** | **Ratio hydrophilic residues**  **(%)** |  |
| Pep 2i | FrYK | Small drugs | - H9c2, A2780 - Mice |  | Endocytosis | 612,74 | 1280 | 50 | 10,58 | 3 | 3 | 0,3 | 30,75 | 50 | [424] |
| Pep 2j | YrYK | Small drugs | - H9c2, A2780 - Mice |  | Endocytosis | 628,74 | 2560 | 50 | 10,19 | 3 | 3 | 0,4 | 22,25 | 50 | [424] |
| PEP-1* | KETWWETWWWEWSQPKKKRKV | Protein | - Lipid vesicles |  | Pore formation | 2933,48 | 34140 | 33,33 | 10,45 | 4 | 4 | 0,2 | 19,1 | 52 | [425,426] |
| Pep-1 | KETWWETWWTEWSQPKKKRKV | Proteins, NPs, nucleic acids | - Hs68, HEK 293T, H9c2, SK-OV-3, BV2, SH-SY5Y, NIH3T3, Porcine corneas  - Rats, Mice  - Lipid vesicles | Cancer, cardiovascular Diseases, ocular disorders, metabolic disorders, inflammatory disorders | Endocytosis, pore formation | 2848,37 | 28450 | 28,57 | 10,45 | 4 | 4 | 0,4 | 15,1 | 52 | [203,204,206,210,258,404,411,427-433] |
| Pep2* | GCKKYRRFRWKFKGKFWFWG | Proteins | - THP-1, KG-1 | Cancer |  | 2712,36 | 18470 | 45 | 11,71 | 8,91 | 9 | 0 | 31,7 | 40 | [434] |
| Pep-2 | KETWFETWFTEWSQPKKKRKV | Nucleic acids | - HS-68, HUVEC, HeLa, MCF-7 | Cancer | Endocytosis-independent | 2770,29 | 17070 | 28,57 | 10,45 | 4 | 4 | 0,5 | 15,1 | 52 | [435,436] |
| Pep-21 | KATWFETWFTEWSQPKKKRKV | Nucleic acids | - HeLa, HUVEC, MCF-7 | Cancer |  | 2712,25 | 17070 | 33,33 | 10,78 | 5 | 5 | 0,3 | 18,52 | 48 | [435] |
| Pep-29 | KETWFETWFAEWSQPKKKRKV | Nucleic acids | - HeLa, HUVEC, MCF-7 | Cancer |  | 2740,26 | 17070 | 33,33 | 10,45 | 4 | 4 | 0,5 | 16,43 | 52 | [435] |
| Pep-3 | KWFETWFTEWPKKRK | Nucleic acids | - HeLa, HUVEC, MCF-7 | Cancer |  | 2097,52 | 17070 | 33,33 | 10,63 | 4 | 4 | 0,3 | 19,8 | 47 | [435] |
| Pep-30 | KETWFETWFTAWSQPKKKRKV | Nucleic acids | - HeLa, HUVEC, MCF-7 | Cancer |  | 2712,25 | 17070 | 33,33 | 10,78 | 5 | 5 | 0,3 | 18,52 | 48 | [435] |
| Pep-32 | KETWFETWFTEWAQPKKKRKV | Nucleic acids | - HeLa, HUVEC, MCF-7 | Cancer |  | 2754,29 | 17070 | 33,33 | 10,45 | 4 | 4 | 0,4 | 17,29 | 48 | [435] |
| Pep-33 | KETWFETWFTEWSAPKKKRKV | Nucleic acids | - HeLa, HUVEC, MCF-7 | Cancer |  | 2713,23 | 17070 | 33,33 | 10,45 | 4 | 4 | 0,4 | 17,52 | 48 | [435] |
| Pep-40 | KETWFETWFTEWSQPKKKRKA | Nucleic acids | - HeLa, HUVEC, MCF-7 | Cancer |  | 2742,23 | 17070 | 28,57 | 10,45 | 4 | 4 | 0,5 | 13,43 | 52 | [435] |
| Pep-43 | KETWFETWFTEWSQPKKKRKV | Nucleic acids | - HeLa, HUVEC, MCF-7 | Cancer |  | 2770,29 | 17070 | 28,57 | 10,45 | 4 | 4 | 0,5 | 15,1 | 52 | [435] |
| pep7* | SDLWEMMMVSLACQY | Proteins | - WI-L2 |  |  | 1807,22 | 7090 | 66,67 | 2,98 | -1,09 | -1 | -0,6 | 33 | 42,8 | [437] |
| pep-7 | GGKKKRKV | Proteins | - Porcine corneas | Ocular disorders | Endocytosis | 900,18 | 0 | 12,5 | 11,91 | 6 | 6 | 1,7 | -3,75 | 63 | [428] |
| Pep-A | RGTKALTEVIPLTEEAELELAENREILKEPVH | Proteins | - Lipid vesicles |  | Pore formation | 3629,31 | 0 | 37,5 | 4,38 | -2,88 | -2 | 0,5 | 17,66 | 41 | [429] |
| Pep-B | MEFSLKDQEAKVSRSGLYRSPSMPENLNRPRLKQVEKFKDNTIPDKKKC | Proteins | - Lipid vesicles |  | Pore formation | 5768,91 | 1400 | 28,57 | 10,24 | 5,91 | 6 | 0,7 | 5,67 | 59 | [429] |
| Aca, Aminocaproic acid; AEC, Aortic endothelial cells; Ahx, 6-aminohexanoic acid; Aib, 2-aminoisobutyric acid; B-ALL, B acute lymphoblastic leukemia; BBMVEC, Bovine brain microvascular endothelial cells; BCECs, Brain capillary endothelial cells; BHK, Baby hamster kidney; BMB, 3,5-bis(mercaptomethyl)benzoyl; BMDCs, Bone marrow-derived dendritic cells; BMEC, Brain microvascular endothelial cells; Cha, Cyclohexylalanine; CHO, Chinese hamster ovary; Dap, Diaminopimelic acid; DC2.4, Mouse dendritic cells; DMECs, Dermal microvessel endothelial cells; Dmt, *N,N*-Dimethyltryptamine; ES, Embryonic stem cells; F2, Diphenyl; GICs, Glioma-initiating cells; HASMCs, primary human aortic smooth muscle cells; HCE, Human corneal epithelial; HCEC, Human corneal epithelial cells; HCM, Human cardiac myocytes; HEK, Human embryonic kidney; HEL, Human erythroleukemia cell; Hex, Hexyl; hfRPE, Human fetal retinal pigment epithelial cells; hMSCs, Human mesenchymal stem cells; HMVEC, Human lung microvascular endothelial cells; HUVECs, Human umbilical vein endothelial cells; MDCK, Madin-Darby canine kidney; Met, Methylation; Nap, Naphthylalanine; NHC, Human conjunctival epithelial cells; NHDF, Normal human dermal fibroblasts; NPs, Nanoparticles; RAECM, Rat alveolar epithelial cell monolayers; SDHCEC, Spontaneously derived human corneal epithelial cells; Sip, Silaproline; X, Cyclohexylalanine; , Naphthylalanone; - No name attributed; * Different peptides with similar designations; | | | | | | | | | | | | | | | |

**Table S3. Features of published cell-penetrating peptides (cont.).**

| **Peptide** | **Typical sequence** | **Main cargoes** | **Cell model** | **Diseases** | **Proposed translocation** | **Physicochemical properties** | | | | | | | | | **Ref.** |
| --- | --- | --- | --- | --- | --- | --- | --- | --- | --- | --- | --- | --- | --- | --- | --- |
|  |  |  |  |  |  | **Molecular weight**  **(g/mol)** | **Extinction coefficient**  **(M^-1^.cm^-1^)** | **Hydrophobic**  **(%)** | **Isoelectric point** | **Net charge**  **(pH 7)** | **Charge** | **Average hydrophobicity** | **Hydrophobicity**  **(pH 7)** | **Ratio hydrophilic residues**  **(%)** |  |
| PepM | KLFMALVAFLRFLTIPPTAGILKRWGTI | Nucleic acids | - HEK293, HepG2, BHK, HEK  - Bacteria | Infectious Diseases | Endocytosis-independent | 3175,14 | 5690 | 60,71 | 12,53 | 5 | 5 | -0,7 | 47,54 | 14 | [438,439] |
| PEP-NJSM | RYAKMKRRRRRVARRHRRR |  | - Bacteria | Infectious Diseases |  | 2665,29 | 1280 | 26,32 | 13,26 | 14,11 | 15 | 1,7 | 5,42 | 68 | [440] |
| PepFect14 | AGYLLGKLLQQLAAAALQQLL | NPs, nucleic acid | - CHO, HeLa, U2OS, U87  - Mice | Cancer, inflammatory disorders |  | 1960,81 | 1280 | 66,67 | 9,8 | 2 | 2 | -0,7 | 47,86 | 24 | [205,441-444] |
| PepFect28 | AGYLLGKLLQQLAAAALQQLLCSSCTFFYGGSRGKRNNFKTEEY | Nucleic acids | - U88 | Cancer |  | 4860,83 | 4080 | 47,73 | 9,33 | 3,82 | 4 | -0,3 | 29,84 | 36 | [444] |
| PepNu | RLRRKRRKRRKRRKRK | Nucleic acids | - Mice | Infectious Diseases |  | 2333,98 | 0 | 6,25 | 13,66 | 16 | 16 | 2,7 | -9,69 | 94 | [445] |
| PepR | LKRWGTIKKSKAINVLRGFRKEIGRMLNILNRRRR | Nucleic acids | - HEK293, HepG2, BHK, HEK |  | Endocytosis | 4279,43 | 5690 | 37,14 | 12,98 | 13 | 13 | 0,5 | 23,09 | 51 | [438,439] |
| Peptide 0 | ARDIL(Dap)RLLQ |  | - HEK293T, MCF-7 | Cancer |  | 1097,38 | 0 | 55,56 | 11,04 | 2 | 2 | 0,2 | 38,56 | 44 | [446] |
| Peptide 3 Gu | ARRPRRPRRPGGG |  | - HeLa, A549, Jurkat T, CHO-K1 |  |  | 1488,77 | 0 | 7,69 | 13,33 | 7 | 7 | 1,3 | -13,92 | 46 | [447] |
| Peptide 3-F | ARDIF(Dap)RFFQ |  | - HEK293T, MCF-7 | Cancer |  | 1199,41 | 0 | 55,56 | 11,04 | 2 | 2 | -0,1 | 37,56 | 44 | [446] |
| Peptide 3-W | ARIW(Dap)RWWQ |  | - HEK293T, MCF-7 | Cancer |  | 1201,44 | 17070 | 62,5 | 12,49 | 3 | 3 | -0,8 | 49,12 | 38 | [446] |
| Peptide b3-1 747-762 | YKEATSTFTNITYRGT | Proteins | - HEL | Cardiovascular Diseases |  | 1853,09 | 2560 | 31,25 | 9,52 | 2 | 2 | -0,1 | 19,62 | 31 | [448] |
| PEPTIDE II* | Klaklalkalkaalkla |  | - AEC | Cardiovascular Diseases |  | 1764,41 | 0 | 70,59 | 11,3 | 6 | 6 | 0,1 | 43 | 29 | [293] |
| Peptide II | GCKLGLKLGLKGLKGGLKLG | Nucleic acids | - AEC | Cardiovascular Diseases |  | 1953,66 | 120 | 40 | 10,79 | 5,91 | 6 | 0,1 | 31,7 | 25 | [314] |
| Peptide III | GCKALKLKAALALLAKLKLA | Nucleic acids | - AEC | Cardiovascular Diseases |  | 2037,78 | 120 | 70 | 10,79 | 5,91 | 6 | -0,1 | 44 | 25 | [314] |
| Peptide IV | GCKGLKLKGGLGLLGKLKLG | Nucleic acids | - AEC | Cardiovascular Diseases |  | 1953,66 | 120 | 40 | 10,79 | 5,91 | 6 | 0,1 | 31,7 | 25 | [314] |
| Peptide V | GCRQIKIWFQNRRMKWKK | Nucleic acids | - AEC | Cardiovascular Diseases |  | 2407,05 | 11500 | 38,89 | 12,24 | 7,91 | 8 | 0,4 | 23,17 | 56 | [314] |
| Peptide third | NRPDSAQFWLHH | NPs | - A431 | Cutaneous disorders | Clathrin-mediated endocytosis | 1507,69 | 5690 | 33,33 | 8,13 | 1,22 | 3 | -0,2 | 15 | 42 | [449] |
| Peptide 73 | RLWDIVRRWVGWL |  | - Bacteria - Lipid vesicles | - Infectious Diseases |  | 1755,18 | 17070 | 61,54 | 12,17 | 3 | 3 | -0,5 | 49,62 | 31 | [155] |
| Peptide 77 | RLWDIVRRVWGWL |  | - Bacteria - Lipid vesicles | - Infectious Diseases |  | 1755,18 | 17070 | 61,54 | 12,17 | 3 | 3 | -0,5 | 49,62 | 31 | [155] |
| Aca, Aminocaproic acid; AEC, Aortic endothelial cells; Ahx, 6-aminohexanoic acid; Aib, 2-aminoisobutyric acid; B-ALL, B acute lymphoblastic leukemia; BBMVEC, Bovine brain microvascular endothelial cells; BCECs, Brain capillary endothelial cells; BHK, Baby hamster kidney; BMB, 3,5-bis(mercaptomethyl)benzoyl; BMDCs, Bone marrow-derived dendritic cells; BMEC, Brain microvascular endothelial cells; Cha, Cyclohexylalanine; CHO, Chinese hamster ovary; Dap, Diaminopimelic acid; DC2.4, Mouse dendritic cells; DMECs, Dermal microvessel endothelial cells; Dmt, *N,N*-Dimethyltryptamine; ES, Embryonic stem cells; F2, Diphenyl; GICs, Glioma-initiating cells; HASMCs, primary human aortic smooth muscle cells; HCE, Human corneal epithelial; HCEC, Human corneal epithelial cells; HCM, Human cardiac myocytes; HEK, Human embryonic kidney; HEL, Human erythroleukemia cell; Hex, Hexyl; hfRPE, Human fetal retinal pigment epithelial cells; hMSCs, Human mesenchymal stem cells; HMVEC, Human lung microvascular endothelial cells; HUVECs, Human umbilical vein endothelial cells; MDCK, Madin-Darby canine kidney; Met, Methylation; Nap, Naphthylalanine; NHC, Human conjunctival epithelial cells; NHDF, Normal human dermal fibroblasts; NPs, Nanoparticles; RAECM, Rat alveolar epithelial cell monolayers; SDHCEC, Spontaneously derived human corneal epithelial cells; Sip, Silaproline; X, Cyclohexylalanine; , Naphthylalanone; - No name attributed; * Different peptides with similar designations; | | | | | | | | | | | | | | | |

**Table S3. Features of published cell-penetrating peptides (cont.).**

| **Peptide** | **Typical sequence** | **Main cargoes** | **Cell model** | **Diseases** | **Proposed translocation** | **Physicochemical properties** | | | | | | | | | **Ref.** |
| --- | --- | --- | --- | --- | --- | --- | --- | --- | --- | --- | --- | --- | --- | --- | --- |
|  |  |  |  |  |  | **Molecular weight**  **(g/mol)** | **Extinction coefficient**  **(M^-1^.cm^-1^)** | **Hydrophobic**  **(%)** | **Isoelectric point** | **Net charge**  **(pH 7)** | **Charge** | **Average hydrophobicity** | **Hydrophobicity**  **(pH 7)** | **Ratio hydrophilic residues**  **(%)** |  |
| PFV | PFVYLI | NPs | - HeLa, MCF-7, MDA-MB-435S, 4T1 - Mice | Cancer | Lipid Raft, clathrin-mediated endocytosis | 750,97 | 1280 | 83,33 | 6 | 1 | 1 | -1,7 | 64,83 | 0 | [413] |
| PI | CASPSGALRSC | Proteins | - MDA-MB-231 - Mice | Cancer | Caveolae-mediated endocytosis | 1051,23 | 240 | 45,45 | 7,99 | 1,82 | 2 | -0,1 | 18,64 | 36 | [450] |
| PIsI | RVIRVWFQNKRCKDKK | Proteins | - Bowes melanoma  - Lipid vesicles |  |  | 2104,63 | 5810 | 37,5 | 11,63 | 6,91 | 7 | 0,8 | 15,88 | 63 | [375,403] |
| Pip2a | R(Ahx)RRARR(Ahx)RYQFLIR(Ahx)RAR(Ahx)RA | Nucleic acids | - Mice | Muscular disorders |  | 2910,59 | 1280 | 26,67 | 13,16 | 11 | 11 | 1,1 | 13,93 | 61 | [451] |
| Pip2b | R(Ahx)RR(Ahx)RR(Ahx)RIHILFQNrRMKWHK | Proteins | - C2C12, Neonatal cardiac cells - Mice | Cardiovascular Diseases | Endocytosis | 3184 | 5690 | 30 | 13,54 | 11,22 | 13 | 0,8 | 18,65 | 60 | [452] |
| Pip5e | R(Ahx)RRARR(Ahx)RILFQYR(Ahx)RAR(Ahx)RA | Small drugs, nucleic acids | - Myotubes  - Mice | Cardiovascular Diseases, muscular disorders |  | 2910,59 | 1280 | 26,67 | 13,16 | 11 | 11 | 1,1 | 13,93 | 61 | [453,454] |
| Pip6a | R(Ahx)RRARR(Ahx)RYQFLIR(Ahx)RAR(Ahx)RA | Nucleic acids | - Murine H2k, SH-SY5Y, Myotubes - Mice | Muscular disorders | Endocytosis, clathrin-mediated endocytosis | 3661,55 | 1280 | 26,67 | 13,16 | 11 | 11 | 1,1 | 13,93 | 61 | [454-457] |
| Pip6f | R(Ahx)RRARR(Ahx)RFQILYR(Ahx)RAR(Ahx)RA | Nucleic acids | - Mice | Cardiovascular Diseases |  | 2995,71 | 1280 | 26,67 | 13,16 | 11 | 11 | 1,1 | 13,93 | 61 | [458] |
| PNC27 | PPLSQETFSDLWKLLKKWKMRRNQFWVKVQRG | Proteins | - L02, HUVEC, ECV304, HeLa, MDA-MB-435, MDA-MB-231, HCM, MCF-7, HK-2, A375 | Cancer |  | 4031,97 | 17070 | 37,5 | 11,69 | 7 | 7 | 0,1 | 22,12 | 50 | [158] |
| POD | ARKKAAKAARKKAAKAARKKAAKAARKKAAKA | Protein, nucleic acids | - A549, HER | Ocular disorders |  | 3318,21 | 0 | 50 | 12,99 | 17 | 17 | 1,3 | 10,12 | 50 | [459,460] |
| PPMO | YARVRRRGPRGYARVRRRGPRRC | Proteins | - Bacteria  - Mice | Cardiovascular Diseases, infectious Diseases |  | 2871,46 | 2680 | 30,43 | 12,75 | 11,91 | 12 | 1 | 7,09 | 48 | [461,462] |
| PR9 | PPLSQETFSDLWKLLRRRRRRRRR | Proteins | - L02, HUVEC, ECV304, HeLa, MDA-MB-435, MDA-MB-231, HCM, MCF-7, HK-2, A375 | Cancer |  | 3179,84 | 5690 | 25 | 12,81 | 9 | 9 | 1 | 10,83 | 63 | [158] |
| PreS2-TLM | PLSSIFSRIGDP | Proteins | - A431, SK-BR-3, HepG2, 293, HeLa - Rats, Mice | Cancer | RMT | 1288,52 | 0 | 33,33 | 6,95 | 1 | 1 | -0,1 | 18,25 | 42 | [290,463] |
| PRL4 | PRLPRLPRLPRL | Proteins | - Rats | Metabolic disorders |  | 1483,93 | 0 | 33,33 | 12,97 | 5 | 5 | 0,4 | 13,33 | 33 | [219] |
| Protamine 1 | PRRRRSSSRPVRRRRRPRVSRRRRRRGGRRRR | NPs | - Caco-2, HeLa |  |  | 4250,08 | 0 | 6,25 | 13,91 | 22 | 22 | 1,9 | -9,38 | 78 | [163] |
| Protamine 2 | PRRRRSSSRPIRRRRPRRASRRRRRRGGRRRR | NPs | - Caco-2, HeLa |  |  | 4236,05 | 0 | 6,25 | 13,91 | 22 | 22 | 1,9 | -9,75 | 78 | [163] |
| Aca, Aminocaproic acid; AEC, Aortic endothelial cells; Ahx, 6-aminohexanoic acid; Aib, 2-aminoisobutyric acid; B-ALL, B acute lymphoblastic leukemia; BBMVEC, Bovine brain microvascular endothelial cells; BCECs, Brain capillary endothelial cells; BHK, Baby hamster kidney; BMB, 3,5-bis(mercaptomethyl)benzoyl; BMDCs, Bone marrow-derived dendritic cells; BMEC, Brain microvascular endothelial cells; Cha, Cyclohexylalanine; CHO, Chinese hamster ovary; Dap, Diaminopimelic acid; DC2.4, Mouse dendritic cells; DMECs, Dermal microvessel endothelial cells; Dmt, *N,N*-Dimethyltryptamine; ES, Embryonic stem cells; F2, Diphenyl; GICs, Glioma-initiating cells; HASMCs, primary human aortic smooth muscle cells; HCE, Human corneal epithelial; HCEC, Human corneal epithelial cells; HCM, Human cardiac myocytes; HEK, Human embryonic kidney; HEL, Human erythroleukemia cell; Hex, Hexyl; hfRPE, Human fetal retinal pigment epithelial cells; hMSCs, Human mesenchymal stem cells; HMVEC, Human lung microvascular endothelial cells; HUVECs, Human umbilical vein endothelial cells; MDCK, Madin-Darby canine kidney; Met, Methylation; Nap, Naphthylalanine; NHC, Human conjunctival epithelial cells; NHDF, Normal human dermal fibroblasts; NPs, Nanoparticles; RAECM, Rat alveolar epithelial cell monolayers; SDHCEC, Spontaneously derived human corneal epithelial cells; Sip, Silaproline; X, Cyclohexylalanine; , Naphthylalanone; - No name attributed; * Different peptides with similar designations; | | | | | | | | | | | | | | | |

**Table S3. Features of published cell-penetrating peptides (cont.).**

| **Peptide** | **Typical sequence** | **Main cargoes** | **Cell model** | **Diseases** | **Proposed translocation** | **Physicochemical properties** | | | | | | | | | **Ref.** |
| --- | --- | --- | --- | --- | --- | --- | --- | --- | --- | --- | --- | --- | --- | --- | --- |
|  |  |  |  |  |  | **Molecular weight**  **(g/mol)** | **Extinction coefficient**  **(M^-1^.cm^-1^)** | **Hydrophobic**  **(%)** | **Isoelectric point** | **Net charge**  **(pH 7)** | **Charge** | **Average hydrophobicity** | **Hydrophobicity**  **(pH 7)** | **Ratio hydrophilic residues**  **(%)** |  |
| Protamine  (LMWP) | VSRRRRRRGGRRRR | NPs, small drugs, nucleic acids | - Cos-7, HT-1080, bEnd.3, A549, MDA-MB-231, HCT 116, HCT-29, LS 147T, NCI-H460, CEC, NHC  - Mice, Dogs | Cancer, ocular disorders | Endocytosis, clathrin-mediated endocytosis | 1880,25 | 0 | 7,14 | 13,66 | 11 | 11 | 2,1 | -4,93 | 79 | [409,464-469] |
| PT | PPLSQETFSDLWKLLYGRKKRRQRRR | Proteins | - L02, HUVEC, ECV304, HeLa, MDA-MB-435, MDA-MB-231, HCM, MCF-7, HK-2, A375 | Cancer |  | 3316,01 | 6970 | 26,92 | 12,2 | 8 | 8 | 0,7 | 11,88 | 58 | [158] |
| PTD3 | YARKARRQARR | Proteins, small drugs, nucleic acids | - Jurkat T, T98G - Mice | Cancer | Macropinocytosis | 1431,7 | 1280 | 36,36 | 12,69 | 7 | 7 | 1,3 | 7,55 | 64 | [470,471] |
| PTD4 | YARAAARQARA | Proteins, NPs, small drugs, nucleic acids | - Jurkat T, Hig-82, HEL299, HeLa, HEK293, OS143B, EC-9706, MDA-MB-231, S180, H22, Fibroblasts - Hamsters, Rabbits, Mice | Cancer, cutaneous disorders |  | 1204,38 | 1280 | 63,64 | 12,18 | 4 | 4 | 0,4 | 23,36 | 36 | [157,388,413,414,470,472,473] |
| D-PTD4 | Araqraaray |  | - C2C12 |  |  | 1133,3 | 1280 | 60 | 12,18 | 4 | 4 | 0,4 | 21,6 | 40 | [474] |
| PTD5 | RRQRRTSKLMKR | Proteins, small drugs, nucleic acids | - HIG-82, Clu-3, CHO K1, HeLa, A549, Jurkat T, 293, PC-3, Hig-82,  - Rabbits, Mice | Cancer, inflammatory disorders |  | 1616,02 | 0 | 16,67 | 13,17 | 8 | 8 | 1,5 | 4,67 | 75 | [193,278,414,470] |
| pVEC | LLIILRRRIRKQAHAHSK | Proteins, small drugs, nucleic acids | - Bowes melanoma, HeLa, A431, SK-BR3, CHO, RAW264.7, bEnd.3, AEC, HBCEC  - Bacteria  - Rats, Mice  - Lipid vesicles | Cancer, metabolic disorders, infectious Diseases | Endocytosis | 2209,83 | 0 | 44,44 | 12,97 | 7,22 | 9 | 0,3 | 32,11 | 44 | [204,219,290,312,390,391,394,475-477] |
| D-pVEC | LLIILRRRIRKQAHAHSK | Proteins | - Bowes melanoma  - Rats | Metabolic disorders | Endocytosis | 2209,83 | 0 | 44,44 | 12,97 | 7,22 | 9 | 0,3 | 32,11 | 44 | [219,475] |
| pVEC (del5) | RRRIRKQAHAHSK |  | - Bacteria - Lipid vesicles | Infectious Diseases |  | 1643,98 | 0 | 23,08 | 12,97 | 7,22 | 9 | 1,1 | 6,15 | 62 | [477] |
| pVEC (a1) | LLIILRRRWRKQARARSK |  | - RAW264,7 - Bacteria | Infectious Diseases |  | 2320,96 | 5690 | 44,44 | 13,33 | 9 | 9 | 0,6 | 29,56 | 56 | [476] |
| pVEC (a2) | LLIILRRRWRKQAKAKSK |  | - RAW264,7 - Bacteria | Infectious Diseases |  | 2264,94 | 5690 | 44,44 | 12,98 | 9 | 9 | 0,6 | 28,56 | 56 | [476] |
| pVEC (a3) | LLIILRRRWRRQARARSR |  | - RAW264,7 - Bacteria | Infectious Diseases |  | 2376,98 | 5690 | 44,44 | 13,53 | 9 | 9 | 0,6 | 30,56 | 56 | [476] |
| pVEC (a4) | LLIILKKKWKKQAKAKSK |  | - RAW264,7 - Bacteria | Infectious Diseases |  | 2152,9 | 5690 | 44,44 | 11,61 | 9 | 9 | 0,6 | 26,56 | 56 | [476] |
| pVEC (Ala10) | LLIILRRRIaKQAHAHSK |  | - Bowes melanoma |  | Endocytosis | 2124,72 | 0 | 50 | 12,82 | 6,22 | 8 | 0,1 | 35,17 | 39 | [475] |
| pVEC (Ala11) | LLIILRRRIRaQAHAHSK |  | - Bowes melanoma |  | Endocytosis | 2152,73 | 0 | 50 | 12,97 | 6,22 | 8 | 0,1 | 35,67 | 39 | [475] |
| Aca, Aminocaproic acid; AEC, Aortic endothelial cells; Ahx, 6-aminohexanoic acid; Aib, 2-aminoisobutyric acid; B-ALL, B acute lymphoblastic leukemia; BBMVEC, Bovine brain microvascular endothelial cells; BCECs, Brain capillary endothelial cells; BHK, Baby hamster kidney; BMB, 3,5-bis(mercaptomethyl)benzoyl; BMDCs, Bone marrow-derived dendritic cells; BMEC, Brain microvascular endothelial cells; Cha, Cyclohexylalanine; CHO, Chinese hamster ovary; Dap, Diaminopimelic acid; DC2.4, Mouse dendritic cells; DMECs, Dermal microvessel endothelial cells; Dmt, *N,N*-Dimethyltryptamine; ES, Embryonic stem cells; F2, Diphenyl; GICs, Glioma-initiating cells; HASMCs, primary human aortic smooth muscle cells; HCE, Human corneal epithelial; HCEC, Human corneal epithelial cells; HCM, Human cardiac myocytes; HEK, Human embryonic kidney; HEL, Human erythroleukemia cell; Hex, Hexyl; hfRPE, Human fetal retinal pigment epithelial cells; hMSCs, Human mesenchymal stem cells; HMVEC, Human lung microvascular endothelial cells; HUVECs, Human umbilical vein endothelial cells; MDCK, Madin-Darby canine kidney; Met, Methylation; Nap, Naphthylalanine; NHC, Human conjunctival epithelial cells; NHDF, Normal human dermal fibroblasts; NPs, Nanoparticles; RAECM, Rat alveolar epithelial cell monolayers; SDHCEC, Spontaneously derived human corneal epithelial cells; Sip, Silaproline; X, Cyclohexylalanine; , Naphthylalanone; - No name attributed; * Different peptides with similar designations; | | | | | | | | | | | | | | | |

**Table S3. Features of published cell-penetrating peptides (cont.).**

| **Peptide** | **Typical sequence** | **Main cargoes** | **Cell model** | **Diseases** | **Proposed translocation** | **Physicochemical properties** | | | | | | | | | **Ref.** |
| --- | --- | --- | --- | --- | --- | --- | --- | --- | --- | --- | --- | --- | --- | --- | --- |
|  |  |  |  |  |  | **Molecular weight**  **(g/mol)** | **Extinction coefficient**  **(M^-1^.cm^-1^)** | **Hydrophobic**  **(%)** | **Isoelectric point** | **Net charge**  **(pH 7)** | **Charge** | **Average hydrophobicity** | **Hydrophobicity**  **(pH 7)** | **Ratio hydrophilic residues**  **(%)** |  |
| pVEC (Ala12) | LLIILRRRIRKaAHAHSK |  | - Bowes melanoma |  | Endocytosis | 2152,77 | 0 | 50 | 12,97 | 7,22 | 9 | 0,3 | 34,94 | 39 | [475] |
| pVEC (Ala16) | LLIILRRRIRKQAHAaSK |  | - Bowes melanoma |  | Endocytosis | 2143,76 | 0 | 50 | 12,97 | 7,11 | 9 | 0,3 | 33,94 | 44 | [475] |
| pVEC (Ala17) | LLIILRRRIRKQAHAHaK |  | - Bowes melanoma |  | Endocytosis | 2193,83 | 0 | 50 | 12,97 | 7,22 | 9 | 0,3 | 34,67 | 39 | [475] |
| pVEC (Ala18) | LLIILRRRIRKQAHAHSa |  | - Bowes melanoma |  | Endocytosis | 2152,73 | 0 | 50 | 12,97 | 6,22 | 8 | 0,1 | 35,67 | 39 | [475] |
| pVEC (Ala6) | LLIILaRRIRKQAHAHSK |  | - Bowes melanoma |  | Endocytosis | 2124,72 | 0 | 50 | 12,82 | 6,22 | 8 | 0,1 | 35,17 | 39 | [475] |
| pVEC (Ala7) | LLIILRaRIRKQAHAHSK |  | - Bowes melanoma |  | Endocytosis | 2124,72 | 0 | 50 | 12,82 | 6,22 | 8 | 0,1 | 35,17 | 39 | [475] |
| pVEC (Ala8) | LLIILRRaIRKQAHAHSK |  | - Bowes melanoma |  | Endocytosis | 2124,72 | 0 | 50 | 12,82 | 6,22 | 8 | 0,1 | 35,17 | 39 | [475] |
| pVEC (D-Ala13) | LLIILRRRIRKQaHAHSK |  | - Bowes melanoma |  | Endocytosis | 2209,83 | 0 | 44,44 | 12,97 | 7,22 | 9 | 0,3 | 32,11 | 44 | [475] |
| pVEC (D-Ala15) | LLIILRRRIRKQAHaHSK |  | - Bowes melanoma |  | Endocytosis | 2209,83 | 0 | 44,44 | 12,97 | 7,22 | 9 | 0,3 | 32,11 | 44 | [475] |
| pVEC (retro) | KSHAHAQKRIRRRLIILL |  | - Lipid vesicles |  |  | 2209,83 | 0 | 44,44 | 12,97 | 7,22 | 8 | 0,3 | 32,11 | 44 | [204] |
| PZ-128 | KKSRALF |  | - Humans (Phase II) | Cardiovascular Diseases |  | 849,07 | 0 | 42,86 | 11,77 | 4 | 4 | 0,6 | 24,71 | 57 | [478] |
| (R6/W3)S-S | RRWWRRWRR | Proteins | - CHO | Cancer |  | 1513,81 | 17070 | 33,33 | 13,33 | 6 | 6 | 0,9 | 23 | 67 | [393] |
| (RFF)3R | RFFRFFRFFR | Nucleic acids | - Bacteria - Mice | Infectious Diseases |  | 1525,85 | 0 | 60 | 12,97 | 4 | 4 | -0,3 | 52,6 | 40 | [479] |
| (RG)5 | RGRGRGRGRG | NPs | - U87, A549, bEnd.3, C8-D1A - Mice | Cancer | Endocytosis | 1084,26 | 0 | 0 | 13,16 | 5 | 5 | 1,5 | -7 | 50 | [480] |
| (RVRR)2 | RVRRRVRR | Proteins | - MDA-MB-231 | Cancer | Endocytosis | 1153,43 | 0 | 25 | 13,33 | 6 | 6 | 1,9 | 8,5 | 75 | [481] |
| (RVRR)3 | RVRRRVRRRVRR | Proteins | - MDA-MB-231 | Cancer | Endocytosis | 1721,14 | 0 | 25 | 13,6 | 9 | 9 | 1,9 | 8,5 | 75 | [481] |
| (RW)3 | RWRWRW | NPs | - MCF-7 | Cancer |  | 2418,99 | 17070 | 50 | 12,81 | 3 | 3 | -0,2 | 41,5 | 50 | [482] |
| (RW)4 | RWRWRWRW | NPs, small drugs | - CCRF-CEM, SK-OV-3, HCT-116, MDA-MB-468, MCF-7 | Cancer |  | 1387,65 | 22760 | 50 | 12,97 | 4 | 4 | -0,2 | 41,5 | 50 | [482,483] |
| (RW)5 | RWRWRWRWRW | NPs | - MCF-7 | Cancer |  | 3103,81 | 28450 | 50 | 13,16 | 5 | 5 | -0,2 | 41,5 | 50 | [482] |
| Aca, Aminocaproic acid; AEC, Aortic endothelial cells; Ahx, 6-aminohexanoic acid; Aib, 2-aminoisobutyric acid; B-ALL, B acute lymphoblastic leukemia; BBMVEC, Bovine brain microvascular endothelial cells; BCECs, Brain capillary endothelial cells; BHK, Baby hamster kidney; BMB, 3,5-bis(mercaptomethyl)benzoyl; BMDCs, Bone marrow-derived dendritic cells; BMEC, Brain microvascular endothelial cells; Cha, Cyclohexylalanine; CHO, Chinese hamster ovary; Dap, Diaminopimelic acid; DC2.4, Mouse dendritic cells; DMECs, Dermal microvessel endothelial cells; Dmt, *N,N*-Dimethyltryptamine; ES, Embryonic stem cells; F2, Diphenyl; GICs, Glioma-initiating cells; HASMCs, primary human aortic smooth muscle cells; HCE, Human corneal epithelial; HCEC, Human corneal epithelial cells; HCM, Human cardiac myocytes; HEK, Human embryonic kidney; HEL, Human erythroleukemia cell; Hex, Hexyl; hfRPE, Human fetal retinal pigment epithelial cells; hMSCs, Human mesenchymal stem cells; HMVEC, Human lung microvascular endothelial cells; HUVECs, Human umbilical vein endothelial cells; MDCK, Madin-Darby canine kidney; Met, Methylation; Nap, Naphthylalanine; NHC, Human conjunctival epithelial cells; NHDF, Normal human dermal fibroblasts; NPs, Nanoparticles; RAECM, Rat alveolar epithelial cell monolayers; SDHCEC, Spontaneously derived human corneal epithelial cells; Sip, Silaproline; X, Cyclohexylalanine; , Naphthylalanone; - No name attributed; * Different peptides with similar designations; | | | | | | | | | | | | | | | |

**Table S3. Features of published cell-penetrating peptides (cont.).**

| **Peptide** | **Typical sequence** | **Main cargoes** | **Cell model** | **Diseases** | **Proposed translocation** | **Physicochemical properties** | | | | | | | | | **Ref.** |
| --- | --- | --- | --- | --- | --- | --- | --- | --- | --- | --- | --- | --- | --- | --- | --- |
|  |  |  |  |  |  | **Molecular weight**  **(g/mol)** | **Extinction coefficient**  **(M^-1^.cm^-1^)** | **Hydrophobic**  **(%)** | **Isoelectric point** | **Net charge**  **(pH 7)** | **Charge** | **Average hydrophobicity** | **Hydrophobicity**  **(pH 7)** | **Ratio hydrophilic residues**  **(%)** |  |
| (RW)6 | RWRWRWRWRWRW | NPs | - MCF-7 | Cancer |  | 3446,22 | 34140 | 50 | 13,33 | 6 | 6 | -0,2 | 41,5 | 50 | [482] |
| (RX)4B | R(Ahx)R(Ahx)R(Ahx)R(Ahx)A | Nucleic acids | - DBT, Vero-E6 - Virus | Infectious Diseases |  | 1166,45 | 0 | 0 | 12,97 | 4 | 4 | 2,3 | -14 | 80 | [234] |
| (RXR)4  (AVI-5126) | R(Ahx)RR(Ahx)RR(Ahx)RR(Ahx)R | Proteins, nucleic acids | - C2C12, Neonatal cardiac cells, DBT, Vero-E6 - Virus, Bacteria  - Rats, Mice | Infectious Diseases, cardiovascular disorders, ocular disorders | Endocytosis | 1720,13 | 0 | 0 | 13,53 | 8 | 8 | 2,3 | -14 | 80 | [452,479,484-486] |
| (RXR)4XB  (Peptide K or P007) | R(Ahx)RR(Ahx)RR(Ahx)RR(Ahx)R(Ahx)A | Small drugs, nucleic acids | - A549, Muscle  - Bacteria, Virus  - Mice | Infectious Diseases, muscular disorders, cardiovascular disorders |  | 1904,36 | 0 | 0 | 13,53 | 8 | 8 | 2,6 | -14 | 89 | [282,451,452,487-490] |
| (RXRRBR)2  (Eteplirsen or (AVI-4658) | R(Ahx)RRARR(Ahx)RRAR | Nucleic acids | Humans (Phase I and II) | Muscular disorders |  | 1635,99 | 0 | 0 | 13,53 | 9 | 9 | 2,3 | -14 | 80 | [491,492] |
| (RXRRBR)2XB  (B peptide) | R(Ahx)RRARR(Ahx)RRAR(Ahx)A | Proteins, nucleic acids | - C2C12, Neonatal cardiac cells, Muscle  - Mice | Cardiovascular Diseases, muscular disorders | Endocytosis | 1820,22 | 0 | 0 | 13,53 | 8 | 8 | 2 | -14 | 73 | [493]41, 243-248 |
| R3 | RRR | Protein | - U251-MG, HeLa, HEK293, K562, Primary rat fibroblasts | Cancer |  | 486,58 | 0 | 0 | 12,81 | 4 | 4 | 3 | -14 | 100 | [494] |
| R4 | RRRR | Proteins | - RAW 264.7, CHO K1, HeLa, A549, Jurkat T |  |  | 642,77 | 0 | 0 | 12,97 | 5 | 5 | 3 | -14 | 100 | [278,495] |
| R5 | RRRRR | Proteins, nucleic acids | - A549, HeLa | Cancer | Endocytosis | 798,96 | 0 | 0 | 13,16 | 6 | 6 | 3 | -14 | 100 | [496,497] |
| R5F2C | RRRRRFFC | Nucleic acids | - HeLa, NIH3T3 |  |  | 1196,46 | 120 | 337,5 | 12,68 | 5,91 | 6 | 1,1 | 21,62 | 63 | [388] |
| R5Q | RRQRR | Proteins | - CHO K1, HeLa, A549, Jurkat T |  |  | 770,91 | 0 | 0 | 12,97 | 5 | 5 | 2,4 | -13,2 | 100 | [278] |
| R5W3R4 | RRRRRWWWRRRR | NPs | - MCF-7 | Cancer |  | 1982,38 | 17070 | 25 | 13,6 | 10 | 10 | 1,4 | 13,75 | 75 | [482] |
| R6 | RRRRRR | Proteins, NPs, small drugs | - CHO K1, HeLa, A549, Jurkat T, NIH-3T3  - Rats, Mice, Pigs | Metabolic disorders, ocular disorders |  | 955,15 | 0 | 0 | 13,33 | 7 | 7 | 3 | -14 | 100 | [278,495,498-500] |
| D-R6 | Rrrrrr | Proteins | - Rats | Metabolic disorders |  | 955,15 | 0 | 0 | 13,33 | 7 | 7 | 3 | -14 | 100 | [499] |
| R6EW | RRRRRREW | Proteins | - Caco-2 - Rats | Metabolic disorders | Endocytosis | 1270,49 | 5690 | 12,5 | 12,81 | 6 | 6 | 2,2 | -2,25 | 88 | [501] |
| R6F2C | RRRRRRFFC | Nucleic acids | - HeLa, NIH3T3 |  |  | 1352,65 | 120 | 33,33 | 12,81 | 6,91 | 7 | 1,3 | 17,67 | 67 | [388] |
| R6H4 | RRRRRRHHHH | NPs | - HepG2, A549 - Mice |  |  | 1503,75 | 0 | 0 | 13,33 | 7,45 | 11 | 1,6 | -5,2 | 60 | [502] |
| Aca, Aminocaproic acid; AEC, Aortic endothelial cells; Ahx, 6-aminohexanoic acid; Aib, 2-aminoisobutyric acid; B-ALL, B acute lymphoblastic leukemia; BBMVEC, Bovine brain microvascular endothelial cells; BCECs, Brain capillary endothelial cells; BHK, Baby hamster kidney; BMB, 3,5-bis(mercaptomethyl)benzoyl; BMDCs, Bone marrow-derived dendritic cells; BMEC, Brain microvascular endothelial cells; Cha, Cyclohexylalanine; CHO, Chinese hamster ovary; Dap, Diaminopimelic acid; DC2.4, Mouse dendritic cells; DMECs, Dermal microvessel endothelial cells; Dmt, *N,N*-Dimethyltryptamine; ES, Embryonic stem cells; F2, Diphenyl; GICs, Glioma-initiating cells; HASMCs, primary human aortic smooth muscle cells; HCE, Human corneal epithelial; HCEC, Human corneal epithelial cells; HCM, Human cardiac myocytes; HEK, Human embryonic kidney; HEL, Human erythroleukemia cell; Hex, Hexyl; hfRPE, Human fetal retinal pigment epithelial cells; hMSCs, Human mesenchymal stem cells; HMVEC, Human lung microvascular endothelial cells; HUVECs, Human umbilical vein endothelial cells; MDCK, Madin-Darby canine kidney; Met, Methylation; Nap, Naphthylalanine; NHC, Human conjunctival epithelial cells; NHDF, Normal human dermal fibroblasts; NPs, Nanoparticles; RAECM, Rat alveolar epithelial cell monolayers; SDHCEC, Spontaneously derived human corneal epithelial cells; Sip, Silaproline; X, Cyclohexylalanine; , Naphthylalanone; - No name attributed; * Different peptides with similar designations; | | | | | | | | | | | | | | | |

**Table S3. Features of published cell-penetrating peptides (cont.).**

| **Peptide** | **Typical sequence** | **Main cargoes** | **Cell model** | **Diseases** | **Proposed translocation** | **Physicochemical properties** | | | | | | | | | **Ref.** |
| --- | --- | --- | --- | --- | --- | --- | --- | --- | --- | --- | --- | --- | --- | --- | --- |
|  |  |  |  |  |  | **Molecular weight**  **(g/mol)** | **Extinction coefficient**  **(M^-1^.cm^-1^)** | **Hydrophobic**  **(%)** | **Isoelectric point** | **Net charge**  **(pH 7)** | **Charge** | **Average hydrophobicity** | **Hydrophobicity**  **(pH 7)** | **Ratio hydrophilic residues**  **(%)** |  |
| R6L3 | RRLLRRLRR |  | - CHO-K1, CHO-pgsA745 |  | Endocytosis | 1294,66 | 0 | 33,33 | 13,33 | 7 | 7 | 1,4 | 24 | 67 | [389] |
| R6W3 | RRWWRRWRR |  | - CHO-K1, CHO-pgsA745  - Lipid vesicles |  | Endocytosis | 1513,81 | 17070 | 33,33 | 13,33 | 7 | 7 | 0,9 | 23 | 67 | [389,430] |
| R7 | RRRRRRR | Proteins, small drugs, nucleic acids | - U251-MG, HeLa, HEK293, K562, Primary rat fibroblasts, Jurkat T, MDA-MB-231, MCF-7, A549  - Mice | Cancer, inflammatory disorders |  | 1111,34 | 0 | 0 | 13,45 | 8 | 8 | 3 | -14 | 100 | [279,289,494,503,504] |
| R7W | RRRRRRRW | NPs | - Lipid vesicles |  |  | 1297,56 | 5690 | 12,5 | 13,45 | 8 | 8 | 2,2 | -0,12 | 88 | [387] |
| R8 | RRRRRRRR | Proteins, NPs, small drugs, nucleic acids | - COS-7, K562, SDC, Caco-2, MARC-145, HeLa, CHO K1, A-745, HEK293, RAW264.7, EL4, U87MG, Neuron, MCF-7, A2780, NIH-3T3, CCD 27 SK, CT26, B16BL6, HVECs, Human fibroblasts, UISO-Mel2, UISO-Mel-6, BHK21  - Virus  - Piglets, Rats, Mice  - Lipid vesicles | Cancer, infectious Diseases, metabolic disorders, ocular disorders | Endocytosis, macropinocytosis, caveolae-mediated endocytosis, direct internalization | 1267,53 | 0 | 0 | 13,53 | 8 | 8 | 3 | -14 | 100 | [219,224,228,278,289,291,309,347,359,369,371-373,395,404,409,416,418,495,499,501,505-520] |
| D-R8 | Rrrrrrrr | Proteins, small drugs, nucleic acids | - HeLa, CHO K1, A-745, Caco-2, HEK293, RAW264.7, EL4, NIH-3T3, C6, PANC-1, CCL-14  - Virus - Ducks, Rats | Cancer, metabolic disorders | Direct internalization | 1267,53 | 0 | 0 | 13,53 | 8 | 8 | 3 | -14 | 100 | [201,219,279,371,372,499,508,521-524] |
| (aca)R8 | (aca)rrrrrrrr |  | - Jurkat T |  |  | 1368,64 | 0 | 0 | 13,53 | 9 | 9 | 2.6 | -11 | 89 | [525] |
| R8 (r2(rR)3) | rrrRrRrR |  | - HeLa, HEK293, RAW264.7, EL4 - Mice |  | Direct internalization | 1267,53 | 0 | 0 | 13,53 | 8 | 8 | 3 | -14 | 100 | [507] |
| R8 (rR)2R4 | rRrRRRRR |  | - HeLa, HEK293, RAW264.7, EL4 - Mice |  | Direct internalization | 1267,53 | 0 | 0 | 13,53 | 8 | 8 | 3 | -14 | 100 | [507] |
| R8 (rR)3R2 | rRrRrRRR |  | - HeLa, HEK293, RAW264.7, EL4 - Mice |  | Direct internalization | 1267,53 | 0 | 0 | 13,53 | 8 | 8 | 3 | -14 | 100 | [507] |
| R8 (rR)4 | rRrRrRrR |  | - HeLa, HEK293, RAW264.7, EL4 - Mice |  | Direct internalization | 1267,53 | 0 | 0 | 13,53 | 8 | 8 | 3 | -14 | 100 | [507] |
| R8H3 | RRRRRRRRHHH | NPs | - Mice | Cancer |  | 1678,98 | 0 | 0 | 13,53 | 9,33 | 12 | 2 | -8 | 73 | [526] |
| R8Q | RRQRRQRR | Proteins | - CHO K1, HeLa, A549, Jurkat T |  |  | 1211,43 | 0 | 0 | 13,33 | 7 | 7 | 2,3 | -13 | 100 | [278] |
| Aca, Aminocaproic acid; AEC, Aortic endothelial cells; Ahx, 6-aminohexanoic acid; Aib, 2-aminoisobutyric acid; B-ALL, B acute lymphoblastic leukemia; BBMVEC, Bovine brain microvascular endothelial cells; BCECs, Brain capillary endothelial cells; BHK, Baby hamster kidney; BMB, 3,5-bis(mercaptomethyl)benzoyl; BMDCs, Bone marrow-derived dendritic cells; BMEC, Brain microvascular endothelial cells; Cha, Cyclohexylalanine; CHO, Chinese hamster ovary; Dap, Diaminopimelic acid; DC2.4, Mouse dendritic cells; DMECs, Dermal microvessel endothelial cells; Dmt, *N,N*-Dimethyltryptamine; ES, Embryonic stem cells; F2, Diphenyl; GICs, Glioma-initiating cells; HASMCs, primary human aortic smooth muscle cells; HCE, Human corneal epithelial; HCEC, Human corneal epithelial cells; HCM, Human cardiac myocytes; HEK, Human embryonic kidney; HEL, Human erythroleukemia cell; Hex, Hexyl; hfRPE, Human fetal retinal pigment epithelial cells; hMSCs, Human mesenchymal stem cells; HMVEC, Human lung microvascular endothelial cells; HUVECs, Human umbilical vein endothelial cells; MDCK, Madin-Darby canine kidney; Met, Methylation; Nap, Naphthylalanine; NHC, Human conjunctival epithelial cells; NHDF, Normal human dermal fibroblasts; NPs, Nanoparticles; RAECM, Rat alveolar epithelial cell monolayers; SDHCEC, Spontaneously derived human corneal epithelial cells; Sip, Silaproline; X, Cyclohexylalanine; , Naphthylalanone; - No name attributed; * Different peptides with similar designations; | | | | | | | | | | | | | | | |

**Table S3. Features of published cell-penetrating peptides (cont.).**

| **Peptide** | **Typical sequence** | **Main cargoes** | **Cell model** | **Diseases** | **Proposed translocation** | **Physicochemical properties** | | | | | | | | | **Ref.** |
| --- | --- | --- | --- | --- | --- | --- | --- | --- | --- | --- | --- | --- | --- | --- | --- |
|  |  |  |  |  |  | **Molecular weight**  **(g/mol)** | **Extinction coefficient**  **(M^-1^.cm^-1^)** | **Hydrophobic**  **(%)** | **Isoelectric point** | **Net charge**  **(pH 7)** | **Charge** | **Average hydrophobicity** | **Hydrophobicity**  **(pH 7)** | **Ratio hydrophilic residues**  **(%)** |  |
| R8Y | RRRRRRRRY | Proteins | - HeLa |  | Endocytosis | 1430,71 | 1280 | 11,11 | 12,97 | 9 | 9 | 2,4 | -5,44 | 89 | [153] |
| D-R9 | Rrrrrrrrr | Proteins, nucleic acids | - Jurkat T, CH27, hPBL, SCC-25, RAECM, HepG2, MDCKII, Bj-hTERT, C2C12, HT1080 - Rats | Cancer, metabolic disorders |  | 1423,72 | 0 | 0 | 13,6 | 10 | 10 | 3 | -14 | 100 | [147,284,527-529] |
| R9F2 | RRRRRRRRRFFC | Nucleic acids | - HeLa, NIH3T3, DBT, Vero-E6 - Virus | Infectious Diseases |  | 1821,22 | 120 | 25 | 13,05 | 9,91 | 10 | 1,8 | 9,75 | 75 | [388,484] |
| R9-LK15 | RRRRRRRRRGGGKLLKLLLKLLLKLLK | Nucleic acids | - BMSCs | Metabolic disorders |  | 3367,5 | 0 | 37,04 | 13,61 | 15 | 15 | 0,9 | 28,11 | 52 | [530] |
| R9 | RRRRRRRRR | Proteins, NPs, small drugs, nucleic acids | - A431, SK-BR-3, A549, B16F1, HepG2, MDA-MB-231, U87MG, BEAS 2B, CAL 27, SCC-15, CHO-K1, HEC, THP-1, HEK293, NIH-373, HeLa, Huh7, HT1080, HT29, SAOS-2, MCF-7, US-OS, SKLU-1, H4IIE, BNL, RT101, T-36274, KLN 205, Jurkat –T, P388D.1, RAW264.7, COS-1, 293, hPBL, IEC-6, L02, HUVEC, ECV304, MDA-MB-435, HK-2, A375, MDCKII, Bj-hTERT, Neuro2a, Neurons, SJSA-1, SW480 - Bacteria, Virus  - Mice - Lipid vesicles | Cancer, metabolic disorders, infectious Diseases, cardiovascular disorders, inflammatory disorders | Endocytosis, macropinocytosis, clathrin-mediated endocytosis, caveolae-mediated endocytosis, direct internalization | 1423,72 | 0 | 0 | 13,6 | 10 | 10 | 3 | -14 | 100 | [158,169,175,177,204,206,214,250,289,290,312,320,367,389,399,400,405,411,430,447,482,497,527,529-556] |
| R10 | RRRRRRRRRR | Proteins | - CHO K1, HeLa, Jurkat T, RAW 264.7  - Rats | Metabolic disorders |  | 1579,91 | 0 | 0 | 13,66 | 11 | 11 | 3 | -14 | 100 | [278,347,495,499] |
| D-R10 | Rrrrrrrrrr | Proteins | - C2C12  - Rats | Metabolic disorders |  | 1579,91 | 0 | 0 | 13,66 | 11 | 11 | 3 | -14 | 100 | [474,499] |
| R11 | RRRRRRRRRRR | Proteins, NPs, small drugs, nucleic acids | - U251-MG, HeLa, HEK293, K562, Primary rat fibroblasts, B16, T24, SV-HUC, U87  - Mice | Cancer, cutaneous disorders |  | 1736,1 | 0 | 0 | 13,7 | 12 | 12 | 3 | -14 | 100 | [494,497,557-559] |
| R11Q | RRQRRQRRQRR | Proteins | - CHO K1, HeLa, A549, Jurkat T |  |  | 1651,95 | 0 | 0 | 13,53 | 9 | 9 | 2,2 | -12,91 | 100 | [278] |
| R12 | RRRRRRRRRRRR | Proteins, small drugs | - HeLa, CHO K1, A-745, RAW 264.7, A549, Jurkat T  - Rats, Mice | Cancer, infectious Diseases, metabolic disorders | Macropinocytosis | 1892,29 | 0 | 0 | 13,74 | 13 | 13 | 3 | -14 | 100 | [219,278,372,495,511] |
| D-R12 | Rrrrrrrrrrrr | Proteins, small drugs | - HeLa, CHO K1, A-745 - Mice | Cancer |  | 1892,29 | 0 | 0 | 13,74 | 13 | 13 | 3 | -14 | 100 | [372,560] |
| Aca, Aminocaproic acid; AEC, Aortic endothelial cells; Ahx, 6-aminohexanoic acid; Aib, 2-aminoisobutyric acid; B-ALL, B acute lymphoblastic leukemia; BBMVEC, Bovine brain microvascular endothelial cells; BCECs, Brain capillary endothelial cells; BHK, Baby hamster kidney; BMB, 3,5-bis(mercaptomethyl)benzoyl; BMDCs, Bone marrow-derived dendritic cells; BMEC, Brain microvascular endothelial cells; Cha, Cyclohexylalanine; CHO, Chinese hamster ovary; Dap, Diaminopimelic acid; DC2.4, Mouse dendritic cells; DMECs, Dermal microvessel endothelial cells; Dmt, *N,N*-Dimethyltryptamine; ES, Embryonic stem cells; F2, Diphenyl; GICs, Glioma-initiating cells; HASMCs, primary human aortic smooth muscle cells; HCE, Human corneal epithelial; HCEC, Human corneal epithelial cells; HCM, Human cardiac myocytes; HEK, Human embryonic kidney; HEL, Human erythroleukemia cell; Hex, Hexyl; hfRPE, Human fetal retinal pigment epithelial cells; hMSCs, Human mesenchymal stem cells; HMVEC, Human lung microvascular endothelial cells; HUVECs, Human umbilical vein endothelial cells; MDCK, Madin-Darby canine kidney; Met, Methylation; Nap, Naphthylalanine; NHC, Human conjunctival epithelial cells; NHDF, Normal human dermal fibroblasts; NPs, Nanoparticles; RAECM, Rat alveolar epithelial cell monolayers; SDHCEC, Spontaneously derived human corneal epithelial cells; Sip, Silaproline; X, Cyclohexylalanine; , Naphthylalanone; - No name attributed; * Different peptides with similar designations; | | | | | | | | | | | | | | | |

**Table S3. Features of published cell-penetrating peptides (cont.).**

| **Peptide** | **Typical sequence** | **Main cargoes** | **Cell model** | **Diseases** | **Proposed translocation** | **Physicochemical properties** | | | | | | | | | **Ref.** |
| --- | --- | --- | --- | --- | --- | --- | --- | --- | --- | --- | --- | --- | --- | --- | --- |
|  |  |  |  |  |  | **Molecular weight**  **(g/mol)** | **Extinction coefficient**  **(M^-1^.cm^-1^)** | **Hydrophobic**  **(%)** | **Isoelectric point** | **Net charge**  **(pH 7)** | **Charge** | **Average hydrophobicity** | **Hydrophobicity**  **(pH 7)** | **Ratio hydrophilic residues**  **(%)** |  |
| R15 | RRRRRRRRRRRRRRR | NPs | - HeLa |  | Clathrin-mediated endocytosis | 2360,86 | 0 | 0 | 13,82 | 16 | 16 | 3 | -14 | 100 | [561] |
| R16 | RRRRRRRRRRRRRRRR | Proteins, NPs, small drugs | - HeLa, CHO-K1, CHO-A745, RAW 264.7 - Mice | Cancer | Macropinocytosis | 2517,05 | 0 | 0 | 13,84 | 17 | 17 | 3 | -14 | 100 | [372,495,562] |
| D-R16 | Rrrrrrrrrrrrrrrr | Small drugs | - HeLa, CHO-K1, CHO-A745, RAW 264.7 - Mice | Cancer |  | 2517,05 | 0 | 0 | 13,84 | 17 | 17 | 3 | -14 | 100 | [372] |
| Rev | TRQARRNRRRRWRERQR | Proteins, NPs, nucleic acids | - Hela, EBTr, CHO-K1, Jurkat T, COS-7  - Rats  - Lipid vesicles | Metabolic disorders | Macropinocytosis | 2437,83 | 5690 | 11,76 | 13,16 | 10 | 10 | 1,7 | -4,76 | 82 | [156,161-163,219,224,418] |
| RG | RRGRRG | Proteins | - NIH-3T3, Jurkat T |  |  | 756,89 | 0 | 0 | 12,97 | 5 | 5 | 2 | -9,33 | 67 | [498] |
| RGD | ACDCRGDCFCG | Proteins | - LM, B16F1, RMA - Mice | Cancer |  | 1149,32 | 480 | 54,55 | 3,88 | -0,36 | 0 | 0,2 | 19,09 | 27 | [340] |
| RH9 | RRHHRRHRR | NPs | - HeLa, MDA-MB-231 - Lipid vesicles | Cancer | Endocytosis | 1366,6 | 0 | 0 | 13,33 | 7,33 | 10 | 1,8 | -6,67 | 67 | [534] |
| RICK | Kwllrwlsrllrwlarwlg | NPs | - Lipid vesicles |  |  | 2523,24 | 22760 | 63,16 | 12,97 | 6 | 6 | -0,2 | 55 | 32 | [209] |
| RIPL | IPLVVPLRRRRRRRRC | NPs | - SK-OV-3, MCF-7, DU145, PC-3 | Cancer |  | 2102,7 | 120 | 37,5 | 12,97 | 8,91 | 9 | 0,9 | 18,5 | 50 | [563] |
| RL2 | MNQKQPACHENDERPFYQKTAPYVPMYYVPNSYPYYGTNLYQRRPAIAINNPYVPRTYYANPAVVRPHAQIPQRQYLPNSHPPTVVRRPNLHPSFIAIPPKKIQDKIIIPTIGGSHHHHHH |  | - MCF-7, MDA-MB-231 | Cancer |  | 14139,8 | 15480 | 37,19 | 10,1 | 11,02 | 20 | -0,2 | 13,67 | 31 | [564] |
| D-RLA | Rlarlarrlarlar | Small drugs | - C6 | Cancer |  | 1692,15 | 0 | 57,14 | 13,33 | 7 | 7 | 0,6 | 34,29 | 43 | [522] |
| RLW | RLW | NPs | - A549, U87, HUVECs - Mice | Cancer |  | 473,59 | 5690 | 66,67 | 11,18 | 2 | 2 | -0,7 | 61 | 33 | [533] |
| rR7 | rRRRRRRR |  | - HeLa, HEK293, RAW264.7, EL4 - Mice |  | Direct internalization | 1267,53 | 0 | 0 | 13,53 | 9 | 9 | 3 | -14 | 100 | [507] |
| RRL helix | RRLRRLLRRLRRLLRRLR | Proteins | - Rats | Metabolic disorders |  | 2528,29 | 0 | 38,89 | 13,7 | 12 | 12 | 1,1 | 30,33 | 61 | [219] |
| D-RRL Helix | RRLRRLLRRLRRLLRRLR | Proteins | - Rats | Metabolic disorders |  | 2528,29 | 0 | 38,89 | 13,7 | 12 | 12 | 1,1 | 30,33 | 61 | [219] |
| RSG-1.2 | DRRRRGSRPSGAERRRRRAAAA | Proteins | - EBTr |  |  | 2564,92 | 0 | 22,73 | 12,87 | 9 | 9 | 1,5 | -3,5 | 64 | [156] |
| RVRR | RVRR | Proteins | - MDA-MB-231 | Cancer | Endocytosis | 585,72 | 0 | 25 | 12,81 | 4 | 4 | 1,9 | 8,5 | 75 | [481] |
| RWR | RRRRWWWWRRRR |  | - CHO-K1 - Lipid vesicles |  | Endocytosis | 2012,41 | 22760 | 33,33 | 13,53 | 9 | 9 | 0,9 | 23 | 67 | [565] |
| Aca, Aminocaproic acid; AEC, Aortic endothelial cells; Ahx, 6-aminohexanoic acid; Aib, 2-aminoisobutyric acid; B-ALL, B acute lymphoblastic leukemia; BBMVEC, Bovine brain microvascular endothelial cells; BCECs, Brain capillary endothelial cells; BHK, Baby hamster kidney; BMB, 3,5-bis(mercaptomethyl)benzoyl; BMDCs, Bone marrow-derived dendritic cells; BMEC, Brain microvascular endothelial cells; Cha, Cyclohexylalanine; CHO, Chinese hamster ovary; Dap, Diaminopimelic acid; DC2.4, Mouse dendritic cells; DMECs, Dermal microvessel endothelial cells; Dmt, *N,N*-Dimethyltryptamine; ES, Embryonic stem cells; F2, Diphenyl; GICs, Glioma-initiating cells; HASMCs, primary human aortic smooth muscle cells; HCE, Human corneal epithelial; HCEC, Human corneal epithelial cells; HCM, Human cardiac myocytes; HEK, Human embryonic kidney; HEL, Human erythroleukemia cell; Hex, Hexyl; hfRPE, Human fetal retinal pigment epithelial cells; hMSCs, Human mesenchymal stem cells; HMVEC, Human lung microvascular endothelial cells; HUVECs, Human umbilical vein endothelial cells; MDCK, Madin-Darby canine kidney; Met, Methylation; Nap, Naphthylalanine; NHC, Human conjunctival epithelial cells; NHDF, Normal human dermal fibroblasts; NPs, Nanoparticles; RAECM, Rat alveolar epithelial cell monolayers; SDHCEC, Spontaneously derived human corneal epithelial cells; Sip, Silaproline; X, Cyclohexylalanine; , Naphthylalanone; - No name attributed; * Different peptides with similar designations; | | | | | | | | | | | | | | | |

**Table S3. Features of published cell-penetrating peptides (cont.).**

| **Peptide** | **Typical sequence** | **Main cargoes** | **Cell model** | **Diseases** | **Proposed translocation** | **Physicochemical properties** | | | | | | | | | **Ref.** |
| --- | --- | --- | --- | --- | --- | --- | --- | --- | --- | --- | --- | --- | --- | --- | --- |
|  |  |  |  |  |  | **Molecular weight**  **(g/mol)** | **Extinction coefficient**  **(M^-1^.cm^-1^)** | **Hydrophobic**  **(%)** | **Isoelectric point** | **Net charge**  **(pH 7)** | **Charge** | **Average hydrophobicity** | **Hydrophobicity**  **(pH 7)** | **Ratio hydrophilic residues**  **(%)** |  |
| RW9 | RRWWRRWRR |  | - CHO-K1, Bacteria, HeLa, MDA-MB-231, HT29  - Bacteria - Lipid vesicles | Cancer, infectious Diseases, inflammatory disorders | Endocytosis | 1513,81 | 17070 | 33,33 | 13,33 | 7 | 7 | 0,9 | 23 | 67 | [531,534,554] |
| RW16 | RRWRRWWRRWWRRWRR |  | - Lipid vesicles | Infectious Diseases Cancer |  | 2697,23 | 34140 | 37,5 | 13,66 | 11 | 11 | 0,6 | 27,62 | 63 | [396] |
| RY | RYIRS | NPs | - HeLa | Cancer |  | 693,82 | 1280 | 40 | 11,21 | 3 | 3 | 0,4 | 25,8 | 60 | [413] |
| S19 | PFVIGAGVLGALGTGIGGI | Protein | - HeLa, A431, HepG2, SK-N-SH |  |  | 1669,13 | 0 | 52,63 | 12,8 | 1 | 1 | -0,8 | 41,84 | 0 | [566] |
| S413-PV | ALWKTLLKKVLKAPKKKRKVC |  | - HeLa, CHO-K1, pgs A-745 |  | Direct internalization | 2480,35 | 5810 | 47,62 | 11,53 | 9,91 | 10 | 0,5 | 26,14 | 43 | [567] |
| SAP  (FT3) | VRLPPPVRLPPPVRLPPP | Proteins | - HeLa, CHO, BJ, HT, BBMVEC, A549  - Mice | Cancer | Endocytosis | 1997,59 | 0 | 33,33 | 12,81 | 4 | 4 | 0 | 4 | 17 | [227,391,568-570] |
| D-SAP | Vrlpppvrlpppvrlppp |  | - Mice |  |  | 1997,59 | 0 | 33,33 | 12,81 | 4 | 4 | 0 | 4 | 17 | [569] |
| SAP(SIP)  (FT3(SIP)) | VRLPP(Sip)VRLPPPVRLPPP |  | - HeLa |  |  | 1900.44 | 0 | 35.29 | 12.81 | 4 | 4 | -0.1 | 6.94 | 18 | [568] |
| SAP(E) | CGGWVELPPPVELPPPVELPPP |  | - Hela, BJ, HT, BBMVEC, A549  - Lipid vesicles | Cancer | Endocytosis | 2319,86 | 5810 | 36,36 | 2,93 | -2,09 | -2 | -0,2 | 7,59 | 14 | [570,571] |
| SAP10 | RRWKFFPWRR | Nucleic acids | - Lipid vesicles |  |  | 1534,87 | 11380 | 40 | 12,97 | 6 | 6 | 0,3 | 26,3 | 50 | [535] |
| SAPr | PPPLRVPPPLRVPPPLRV | Proteins | - HeLa, CHO |  | Endocytosis | 1997,59 | 0 | 33,33 | 12,81 | 44 | 4 | 0 | 4 | 17 | [391] |
| SAR6EW | SARRRRRREW | Proteins, NPs | - Caco-2, Rats | Metabolic disorders | Endocytosis | 1428,65 | 5690 | 20 | 12,81 | 6 | 6 | 1,7 | 1,8 | 80 | [501,572] |
| sC18 | GLRKRLRKFRNKIKEK | Proteins, NPs, nucleic acids | - HeLa, MCF-7, HT-29, HCT-15, FaDu, HEK-293 - Mice  - Lipid vesicles | Cancer | Endocytosis | 2070,65 | 0 | 25 | 12,53 | 9 | 9 | 1,4 | 9,56 | 69 | [248,249,573,574] |
| SIGYPLP | SIGYPLP | Protein | - HUVECs |  |  | 745,91 | 1280 | 42,86 | 8 | 1 | 1 | -0,8 | 23,57 | 14 | 229 |
| SN50 | AAVALLPAVLLALLAPVQRKRQKLMP |  | - NIH 3T3 | Cancer |  | 2781,69 | 0 | 65,38 | 12,53 | 5 | 5 | -0,3 | 39,08 | 23 | [575] |
| SP50 (SPACE) | ACTGSTQHQCG | Proteins, small drugs, nucleic acids | - Pig skin, HUVEC - Rats, Mice | Cutaneous disorders | Endocytosis | 1092,22 | 240 | 27,27 | 6,89 | 1,07 | 1 | -0,3 | 13,45 | 27 | [576,577] |
| SPA | RPKPQQFFGLM | Nucleic acids | - CHO | Cancer |  | 1348,69 | 0 | 36,36 | 11,6 | 3 | 3 | -0,2 | 19,91 | 36 | [578] |
| SS-31 | r(Dmt)KF |  | - HeLa, U87, bEnd.3 |  |  | 635,78 | 5680 | 50 | 11,6 | 3 | 3 | 0 | 39,25 | 50 | [327] |
| Aca, Aminocaproic acid; AEC, Aortic endothelial cells; Ahx, 6-aminohexanoic acid; Aib, 2-aminoisobutyric acid; B-ALL, B acute lymphoblastic leukemia; BBMVEC, Bovine brain microvascular endothelial cells; BCECs, Brain capillary endothelial cells; BHK, Baby hamster kidney; BMB, 3,5-bis(mercaptomethyl)benzoyl; BMDCs, Bone marrow-derived dendritic cells; BMEC, Brain microvascular endothelial cells; Cha, Cyclohexylalanine; CHO, Chinese hamster ovary; Dap, Diaminopimelic acid; DC2.4, Mouse dendritic cells; DMECs, Dermal microvessel endothelial cells; Dmt, *N,N*-Dimethyltryptamine; ES, Embryonic stem cells; F2, Diphenyl; GICs, Glioma-initiating cells; HASMCs, primary human aortic smooth muscle cells; HCE, Human corneal epithelial; HCEC, Human corneal epithelial cells; HCM, Human cardiac myocytes; HEK, Human embryonic kidney; HEL, Human erythroleukemia cell; Hex, Hexyl; hfRPE, Human fetal retinal pigment epithelial cells; hMSCs, Human mesenchymal stem cells; HMVEC, Human lung microvascular endothelial cells; HUVECs, Human umbilical vein endothelial cells; MDCK, Madin-Darby canine kidney; Met, Methylation; Nap, Naphthylalanine; NHC, Human conjunctival epithelial cells; NHDF, Normal human dermal fibroblasts; NPs, Nanoparticles; RAECM, Rat alveolar epithelial cell monolayers; SDHCEC, Spontaneously derived human corneal epithelial cells; Sip, Silaproline; X, Cyclohexylalanine; , Naphthylalanone; - No name attributed; * Different peptides with similar designations; | | | | | | | | | | | | | | | |

**Table S3. Features of published cell-penetrating peptides (cont.).**

| **Peptide** | **Typical sequence** | **Main cargoes** | **Cell model** | **Diseases** | **Proposed translocation** | **Physicochemical properties** | | | | | | | | | **Ref.** |
| --- | --- | --- | --- | --- | --- | --- | --- | --- | --- | --- | --- | --- | --- | --- | --- |
|  |  |  |  |  |  | **Molecular weight**  **(g/mol)** | **Extinction coefficient**  **(M^-1^.cm^-1^)** | **Hydrophobic**  **(%)** | **Isoelectric point** | **Net charge**  **(pH 7)** | **Charge** | **Average hydrophobicity** | **Hydrophobicity**  **(pH 7)** | **Ratio hydrophilic residues**  **(%)** |  |
|  |  |  |  |  |  |  |  |  |  |  |  |  |  |  |  |
| stEK | LKKLLKLRRRRRRRRKKLLKLSSSSSG | Nucleic acids | - HeLa - Mice |  | Endocytosis | 3321,26 | 0 | 25,93 | 13,54 | 15 | 15 | 1,1 | 15,74 | 70 | [579] |
| SV40 | PKKKRKV | Proteins, small drugs, nucleic acids | - EBTr, HeLa, RAW 264.7, PC-3M, A431, U87, HCT116, SK-BR-3 - Mice | Cancer | Direct internalization | 883,18 | 0 | 14,29 | 11,91 | 6 | 6 | 1,9 | -10,86 | 71 | [156,190,253,289,290,495,558] |
| SVS-1 | KVKVKVKVPTKVKVKVK | NPs | - HeLa | Cancer | Direct internalization | 1935,66 | 0 | 41,18 | 11,61 | 9 | 9 | 0,8 | 18,53 | 47 | [580] |
| SynB1 | RGGRLSYSRRRFSTSTGR | Proteins, small drugs | - A431, SK-BR-3, HCE - Rabbits, Mice | Cancer, ocular disorders | AMT | 2100,4 | 1280 | 16,67 | 12,81 | 7 | 7 | 0,7 | 10,11 | 56 | [290,420,581] |
| Tat | YGRKKRRQRRR | Proteins, NPs, small drugs, nucleic acids | - A431, A-745, Caco-2, EBTr, HeLa, HepG22, Huh7, HT1080, HT29, SAOS-2, MCF-7, US-OS, SKLU-1, H4IIE, BNL, RT101, T-36274, KLN 205, Jurkat –T, P388D.1, RAW264.7, COS-1, HUVECs, HEK-293T, K562, SDC, MDA-MB-231, A549, Namalwa cells, RBL-2H3, SK-BR-3, SH-SY5Y, U87MG, Vero E6, U-2OS, CHO, MDCK, HaCaT, S3, PC3, LNCaP, U118MG, D65MG, Neuron, CaSki, NIH 3T3, MSTO, rat 9L, SJSA-1, SW480, BE(2)-C, A2780, THK320, Vero, L02, HUVEC, ECV304, MDA-MB-435, MDA-MB-231, HCM¸ HK-2, A375, C2C12, Neonatal cardiac cells, MSCs, Dendritic cells, C6Bu1, CT26, B16F10, Swiss 3T3, Bovine spermatozoa, Human semen, Skin fibroblasts, Astrocyte-like neural stem cells, FaDu, Lovo, U2OS, HepG2, K562, NHDF- 1064SK, mel526  - Parasites, Virus, Fungus, Bacteria, Drosophila  - Rats, Mice, Rabbits, Mini Pigs  - Humans (Phase I) | Cancer, infectious Diseases, cardiovascular disorders, ocular disorders, metabolic disorders, inflammatory disorders, muscular disorders | Endocytosis, macropinocytosis, clathrin-mediated endocytosis, caveolae-mediated endocytosis, direct internalization | 1559,89 | 1280 | 9,09 | 12,82 | 9 | 9 | 2 | -7 | 82 | [109,142,149,156,158,159,161-163,170,173,174,177,189,196,197,199,202,204,206,212,214,215,218,219,224,236,241,247,248,251-253,255,256,260,265,282-284,289-291,308,310,312,330,342,344,345,347,348,353,355,365,367,369,372,373,382,387-389,391,395,399,400,404,405,407,409,411-414,416,418,420,422,423,430,452,470,516,525,527,543,558,570,582-649] |
| Aca, Aminocaproic acid; AEC, Aortic endothelial cells; Ahx, 6-aminohexanoic acid; Aib, 2-aminoisobutyric acid; B-ALL, B acute lymphoblastic leukemia; BBMVEC, Bovine brain microvascular endothelial cells; BCECs, Brain capillary endothelial cells; BHK, Baby hamster kidney; BMB, 3,5-bis(mercaptomethyl)benzoyl; BMDCs, Bone marrow-derived dendritic cells; BMEC, Brain microvascular endothelial cells; Cha, Cyclohexylalanine; CHO, Chinese hamster ovary; Dap, Diaminopimelic acid; DC2.4, Mouse dendritic cells; DMECs, Dermal microvessel endothelial cells; Dmt, *N,N*-Dimethyltryptamine; ES, Embryonic stem cells; F2, Diphenyl; GICs, Glioma-initiating cells; HASMCs, primary human aortic smooth muscle cells; HCE, Human corneal epithelial; HCEC, Human corneal epithelial cells; HCM, Human cardiac myocytes; HEK, Human embryonic kidney; HEL, Human erythroleukemia cell; Hex, Hexyl; hfRPE, Human fetal retinal pigment epithelial cells; hMSCs, Human mesenchymal stem cells; HMVEC, Human lung microvascular endothelial cells; HUVECs, Human umbilical vein endothelial cells; MDCK, Madin-Darby canine kidney; Met, Methylation; Nap, Naphthylalanine; NHC, Human conjunctival epithelial cells; NHDF, Normal human dermal fibroblasts; NPs, Nanoparticles; RAECM, Rat alveolar epithelial cell monolayers; SDHCEC, Spontaneously derived human corneal epithelial cells; Sip, Silaproline; X, Cyclohexylalanine; , Naphthylalanone; - No name attributed; * Different peptides with similar designations; | | | | | | | | | | | | | | | |

**Table S3. Features of published cell-penetrating peptides (cont.).**

| **Peptide** | **Typical sequence** | **Main cargoes** | **Cell model** | **Diseases** | **Proposed translocation** | **Physicochemical properties** | | | | | | | | | **Ref.** |
| --- | --- | --- | --- | --- | --- | --- | --- | --- | --- | --- | --- | --- | --- | --- | --- |
|  |  |  |  |  |  | **Molecular weight**  **(g/mol)** | **Extinction coefficient**  **(M^-1^.cm^-1^)** | **Hydrophobic**  **(%)** | **Isoelectric point** | **Net charge**  **(pH 7)** | **Charge** | **Average hydrophobicity** | **Hydrophobicity**  **(pH 7)** | **Ratio hydrophilic residues**  **(%)** |  |
| SynB3 | RRLSYSRRRF | Small drugs, nucleic acids | - HeLa  - Mice | Cancer | AMT | 1396,65 | 1280 | 30 | 12,68 | 6 | 6 | 0,9 | 18 | 70 | [289,581] |
| D-SynB3 | RRLSYSRRRF | Small drugs | - Mice | Cancer | AMT | 1396,65 | 1280 | 30 | 12,68 | 6 | 6 | 0,9 | 18 | 70 | [581] |
| D-Tat | Ygrkkrrqrrr | Proteins, nucleic acids | - NIH-3T3, C6, PANC-1, HeLa, CCL-13, RAW 264.7, C2C12  - Lipid vesicles |  |  | 1559,89 | 1280 | 9,09 | 12,82 | 9 | 9 | 2 | -7 | 82 | [162,201,474,495] |
| cTat | rRrQrRkKrG |  | - C2C12 |  |  | 1396,71 | 0 | 0 | 13,33 | 9 | 9 | 2,4 | -14 | 90 | [474] |
| RI-Tat | RRRQRRKKRGY | Nucleic acids | - TA3/St, H1299 | Cancer |  | 1559,89 | 1280 | 9,09 | 12,82 | 9 | 9 | 2 | -7 | 82 | [650] |
| dTat | RKKRRQRRRHRRKKR | NPs, nucleic acids | - A549, TPC-1, 3T3 - Rats | Cancer | Macropinocytosis | 2201,73 | 0 | 0 | 13,6 | 14,11 | 15 | 2,6 | -14,67 | 93 | [651-653] |
| Tat_2_ | RKKRRQRRRRKKRRQRRR | NPs | - HeLa, HCE - Fungus | Infectious Diseases, ocular disorders | Endocytosis | 2746,41 | 0 | 0 | 13,74 | 17 | 17 | 2,7 | -15,56 | 100 | [326] |
| Tat2 | CGGGYGRKKRCGGGYGRKKRRQRRR | Small drugs | - A549 - Mice | Cancer | Endocytosis | 2897.51 | 1280 | 9,09 | 12,82 | 9 | 9 | 2 | -7 | 82 | [465] |
| Tat-HA2 | CRRRQRRKKRGGDIMGEWGNEIFGAIAGFLG | Nucleic acids | - HEK293T, HepG2 - Mice |  |  | 3621,39 | 5810 | 35,48 | 11,77 | 5,91 | 6 | 0,5 | 19,19 | 42 | [301] |
| TB27 | YGRKKRRQRRRGGRFKRFRKKFKKLFKKLS | Proteins | - L02, HUVEC, ECV304, HeLa, MDA-MB-435, MDA-MB-231, HCM, MCF-7, HK-2, A375 | Cancer |  | 4027,1 | 1280 | 23,33 | 13,07 | 19 | 19 | 1,3 | 10,1 | 67 | [158] |
| TB28 | YGRKKRRQRRRGGLRSLGRKILRAWKKYG | Proteins | - L02, HUVEC, ECV304, HeLa, MDA-MB-435, MDA-MB-231, HCM, MCF-7, HK-2, A375 | Cancer |  | 3686,6 | 8250 | 27,59 | 12,82 | 15 | 15 | 0,9 | 14,03 | 55 | [158] |
| TCPP C6H | KRKKKGKGLGKKRDPCLRKYK | Proteins | - HeLa, MGC-803, Bcap-37, 95D, A549, BxPC-3, 5637, MRC-5, SMMC-7721, NCI-H460, A375, T24, 293T, Lovo, MDA-MB-231 | Cancer |  | 2601,37 | 1400 | 19,05 | 11,49 | 11,91 | 12 | 1,5 | -1,81 | 62 | [636] |
| TFIIE-b | lNSAGYLLGKINLKALAALAKKIL |  | - MCF-7, HT29, PC3, KB, MIAOACA2 |  | Endocytosis | 1031,36 | 0 | 12,5 | 11,3 | 6 | 6 | 1,7 | -3,88 | 75 | [331] |
| TH | AGYLLGHINLHHLAHL(Aib)HHIL | NPs | - C2, HepG2 - Mice | Cancer | Endocytosis | 2364,96 | 1280 | 55 | 8,25 | 1,67 | 7 | -1 | 47,5 | 5 | [654] |
| TI | KWCFRVCYRGICYRRCR | Proteins, nucleic acids | - HeLa, MCF-7, MDA-MB-435S, HaCaT, MM96L, HT144, WM164, U-87MG, T98G - Lipid vesicles | Cancer |  | 2268,83 | 8730 | 58,82 | 10,18 | 6,63 | 7 | 0 | 35,18 | 35 | [141,655] |
| cTI | (KWCFRVCYRGICYRRCR)c | Proteins | - HeLa, MCF-7, MDA-MB-435S, HaCaT, MM96L, HT144, WM164, U-87MG, T98G - Lipid vesicles | Cancer |  | 2268,83 | 8730 | 58,82 | 10,18 | 6,63 | 7 | 0 | 35,18 | 35 | [141] |
| Aca, Aminocaproic acid; AEC, Aortic endothelial cells; Ahx, 6-aminohexanoic acid; Aib, 2-aminoisobutyric acid; B-ALL, B acute lymphoblastic leukemia; BBMVEC, Bovine brain microvascular endothelial cells; BCECs, Brain capillary endothelial cells; BHK, Baby hamster kidney; BMB, 3,5-bis(mercaptomethyl)benzoyl; BMDCs, Bone marrow-derived dendritic cells; BMEC, Brain microvascular endothelial cells; Cha, Cyclohexylalanine; CHO, Chinese hamster ovary; Dap, Diaminopimelic acid; DC2.4, Mouse dendritic cells; DMECs, Dermal microvessel endothelial cells; Dmt, *N,N*-Dimethyltryptamine; ES, Embryonic stem cells; F2, Diphenyl; GICs, Glioma-initiating cells; HASMCs, primary human aortic smooth muscle cells; HCE, Human corneal epithelial; HCEC, Human corneal epithelial cells; HCM, Human cardiac myocytes; HEK, Human embryonic kidney; HEL, Human erythroleukemia cell; Hex, Hexyl; hfRPE, Human fetal retinal pigment epithelial cells; hMSCs, Human mesenchymal stem cells; HMVEC, Human lung microvascular endothelial cells; HUVECs, Human umbilical vein endothelial cells; MDCK, Madin-Darby canine kidney; Met, Methylation; Nap, Naphthylalanine; NHC, Human conjunctival epithelial cells; NHDF, Normal human dermal fibroblasts; NPs, Nanoparticles; RAECM, Rat alveolar epithelial cell monolayers; SDHCEC, Spontaneously derived human corneal epithelial cells; Sip, Silaproline; X, Cyclohexylalanine; , Naphthylalanone; - No name attributed; * Different peptides with similar designations; | | | | | | | | | | | | | | | |

**Table S3. Features of published cell-penetrating peptides (cont.).**

| **Peptide** | **Typical sequence** | **Main cargoes** | **Cell model** | **Diseases** | **Proposed translocation** | **Physicochemical properties** | | | | | | | | | **Ref.** |
| --- | --- | --- | --- | --- | --- | --- | --- | --- | --- | --- | --- | --- | --- | --- | --- |
|  |  |  |  |  |  | **Molecular weight**  **(g/mol)** | **Extinction coefficient**  **(M^-1^.cm^-1^)** | **Hydrophobic**  **(%)** | **Isoelectric point** | **Net charge**  **(pH 7)** | **Charge** | **Average hydrophobicity** | **Hydrophobicity**  **(pH 7)** | **Ratio hydrophilic residues**  **(%)** |  |
| TK | YGRKKRRQRRRGGKLAKLAKKLAKLAK | Proteins | - L02, HUVEC, ECV304, HeLa, MDA-MB-435, MDA-MB-231, HCM, MCF-7, HK-2, A375 | Cancer |  | 3180,09 | 1280 | 33,33 | 12,83 | 15 | 15 | 1,1 | 12,93 | 56 | [158] |
| tLyP-1 | CGNKRTRGC | NPs | - PC-3, MDA-MB-231 - Mice | Cancer |  | 994,19 | 240 | 22,22 | 10,27 | 3,82 | 4 | 0,8 | 2,11 | 44 | [656] |
| tLyp-1 (Tyr) | YGGNKRTR |  | - A549, MDA-MB-231, HL-7702 - Mice | Cancer |  | 951,09 | 1280 | 12,5 | 11,46 | 4 | 4 | 0,8 | -2 | 50 | [657] |
| TM9 | PLIYLRLLR | Proteins | - CHO-K1, PgsA-747 - Lipid vesicles |  | Direct internalization | 1156,54 | 1280 | 66,67 | 11,21 | 3 | 3 | -0,6 | 54,22 | 22 | [658] |
| TP1  (WWSP) | PLILLRLLRGQF | Proteins | - CHO, HEK293, HeLa  - Bacteria - Lipid vesicles | Infectious Diseases | Endocytosis | 1438,91 | 0 | 58,33 | 12,49 | 3 | 3 | -0,6 | 51 | 25 | [217,546] |
| TP2 | PLIYLRLLRGQF |  | - CHO - Lipid vesicles |  | Endocytosis | 1488,92 | 1280 | 58,33 | 11,21 | 3 | 3 | -0,6 | 47,92 | 25 | [546] |
| D-TP2 | Pliylrllrgqf |  | - CHO - Lipid vesicles |  | Endocytosis | 1488,92 | 1280 | 58,33 | 11,21 | 3 | 3 | -0,6 | 47,92 | 25 | [546] |
| TP3 | RRILLQLLRGQF |  | - CHO - Lipid vesicles |  | Endocytosis | 1512,95 | 0 | 50 | 12,81 | 4 | 4 | -0,2 | 44,5 | 39,83 | [546] |
| Transportan | GWTLNSAGYLLGKINLKALAALAKKIL | Proteins, small drugs, nucleic acids | - Bowes melanoma, HeLa, HT29, HCT116, PepLook method, C166, EOMA  - Bacteria, Virus  - Lipid vesicles | Cancer, infectious Diseases | Endocytosis, caveolae-mediated endocytosis, endocytosis-independent | 2841,65 | 6970 | 59,26 | 10,73 | 5 | 5 | -0,4 | 40,63 | 26 | [153,214,289,375,384,421,601,659-663] |
| Transportan 7 | LNSAGYLLGKINLKALAALAKKIL |  | - Bowes melanoma, PepLook method |  | Endocytosis | 2497,26 | 1280 | 62,5 | 10,73 | 5 | 5 | -0,3 | 41,12 | 29 | [659,664] |
| Transportan 9 | GWTLNSAGYLLGKLKALAALAKKIL |  | - Bowes melanoma |  | Endocytosis | 2614,37 | 6970 | 60 | 10,73 | 5 | 5 | -0,4 | 41,56 | 24 | [659] |
| Transportan 9 (dR) | GWTLNSAGYLLGKINLKALAALAKKILdRdRdRdRdRdRdRdRdR | Nucleic acids | - 293T, MDCK, RAW, A549 - Virus | Infectious Diseases | Clathrin-mediated endocytosis | 5283,17 | 6970 | 35,56 | 10,66 | 5 | 5 | 0,9 | 10,58 | 56 | [665] |
| Transportan 10  (NickFect15 or PepFect3) | AGYLLGKINLKALAALAKKIL | Proteins, small drugs, nucleic acids | - Bowes melanoma, PepLook method, HeLa, U373MG, RBL-2H3, MCF-7, MDA-MB-231, A2780, 4T1, HT29, HCT116, Swiss 3T3, Bovine spermatozoa, Human semen, HEK293, UOS, N2a, SH-SY5Y, Hepalclc7, HepG2, U87, RD, MEF  - Mice  - Lipid vesicles | Cancer, infectious Diseases | Endocytosis, macropinocytosis | 2182,9 | 1280 | 66,67 | 10,73 | 5 | 5 | -0,3 | 44,43 | 24 | [154,168,173,174,204,205,214,311,312,320,391,430,472,504,601,659,662,666-670] |
| Transportan 10K | AGYLLGKINKLKALAALAKKIL | Proteins | - HeLa, CHO |  | Endocytosis | 2311,08 | 1280 | 63,64 | 10,85 | 6 | 6 | -0,2 | 41,36 | 27 | [391] |
| Aca, Aminocaproic acid; AEC, Aortic endothelial cells; Ahx, 6-aminohexanoic acid; Aib, 2-aminoisobutyric acid; B-ALL, B acute lymphoblastic leukemia; BBMVEC, Bovine brain microvascular endothelial cells; BCECs, Brain capillary endothelial cells; BHK, Baby hamster kidney; BMB, 3,5-bis(mercaptomethyl)benzoyl; BMDCs, Bone marrow-derived dendritic cells; BMEC, Brain microvascular endothelial cells; Cha, Cyclohexylalanine; CHO, Chinese hamster ovary; Dap, Diaminopimelic acid; DC2.4, Mouse dendritic cells; DMECs, Dermal microvessel endothelial cells; Dmt, *N,N*-Dimethyltryptamine; ES, Embryonic stem cells; F2, Diphenyl; GICs, Glioma-initiating cells; HASMCs, primary human aortic smooth muscle cells; HCE, Human corneal epithelial; HCEC, Human corneal epithelial cells; HCM, Human cardiac myocytes; HEK, Human embryonic kidney; HEL, Human erythroleukemia cell; Hex, Hexyl; hfRPE, Human fetal retinal pigment epithelial cells; hMSCs, Human mesenchymal stem cells; HMVEC, Human lung microvascular endothelial cells; HUVECs, Human umbilical vein endothelial cells; MDCK, Madin-Darby canine kidney; Met, Methylation; Nap, Naphthylalanine; NHC, Human conjunctival epithelial cells; NHDF, Normal human dermal fibroblasts; NPs, Nanoparticles; RAECM, Rat alveolar epithelial cell monolayers; SDHCEC, Spontaneously derived human corneal epithelial cells; Sip, Silaproline; X, Cyclohexylalanine; , Naphthylalanone; - No name attributed; * Different peptides with similar designations; | | | | | | | | | | | | | | | |

**Table S3. Features of published cell-penetrating peptides (cont.).**

| **Peptide** | **Typical sequence** | **Main cargoes** | **Cell model** | **Diseases** | **Proposed translocation** | **Physicochemical properties** | | | | | | | | | **Ref.** |
| --- | --- | --- | --- | --- | --- | --- | --- | --- | --- | --- | --- | --- | --- | --- | --- |
|  |  |  |  |  |  | **Molecular weight**  **(g/mol)** | **Extinction coefficient**  **(M^-1^.cm^-1^)** | **Hydrophobic**  **(%)** | **Isoelectric point** | **Net charge**  **(pH 7)** | **Charge** | **Average hydrophobicity** | **Hydrophobicity**  **(pH 7)** | **Ratio hydrophilic residues**  **(%)** |  |
| vCPP0275 | KKRYKKKYKAYKPYKKKKKF | Nucleic acids | - HEK293  - Bacteria | Infectious Diseases |  | 2680,46 | 5120 | 30 | 10,95 | 14 | 14 | 1,3 | 2,7 | 65 | [439,671] |
| vCPP0417 | SPRRRTPSPRRRRSQSPRRR | Nucleic acids | - HEK293  - Bacteria | Infectious Diseases |  | 2545,96 | 0 | 0 | 13,66 | 11 | 11 | 1,5 | -17,05 | 75 | [439,671] |
| vCPP0667 | RPRRRATTRRRITTGTRRRR | Nucleic acids | - HEK293  - Bacteria | Infectious Diseases |  | 2580,08 | 0 | 10 | 13,7 | 12 | 12 | 1,4 | 0,25 | 55 | [439,671] |
| vCPP0769 | RRLTLRQLLGLGSRRRRRSR | Nucleic acids | - HEK293  - Bacteria | Infectious Diseases |  | 2507,1 | 0 | 25 | 13,6 | 10 | 10 | 0,9 | 18,35 | 60 | [439,671] |
| vCPP1779 | GRRGPRRANQNGTRRRRRRT | Nucleic acids | - HEK293  - Bacteria | Infectious Diseases |  | 2477,87 | 0 | 5 | 13,66 | 11 | 11 | 1,5 | -10,55 | 65 | [439,671] |
| vCPP2319 | WRRRYRRWRRRRRWRRRPRR | Nucleic acids | - HEK293  - Bacteria | Infectious Diseases |  | 3179,82 | 18350 | 20 | 13,49 | 16 | 16 | 1,6 | 4,9 | 75 | [439,671] |
| Vectocell | CVKRGLKLRHVRPRVTRMDV | Small drugs | - HCT 116, HCT-29, LS 147T, NCI-H460, MDA-MB-231 - Dogs, Mice | Cancer |  | 2420,09 | 120 | 40 | 12,32 | 6,02 | 7 | 0,6 | 21,55 | 40 | [672] |
| Vectocell 15b | GAYDLRRRERQSRLRRRERQSR | Proteins | - HeLa, CHO-K1, PgsA-745 |  | Direct internalization | 2901,34 | 1280 | 18,18 | 12,49 | 7 | 7 | 1,5 | 0,77 | 77 | [215] |
| VG-21 | VTPHHVLVDEYTGEWVDSQFK | NPs | - Hep-2, HeLa, Cos-7 | Infectious Diseases |  | 2486,84 | 6970 | 38,1 | 4,54 | -2,77 | -1 | -0,1 | 21,29 | 33 | [673] |
| VP22 | DAATATRGRSAASRPTERPRAPARSASRPRPRRPVE | Proteins, NPs, nucleic acids | - HepG22, Huh7, HT1080, HT29, SAOS-2, MCF-7, US-OS, SKLU-1, H4IIE, BNL, RT101, T-36274, HeLa, KLN 205, Jurkat –T, P388D.1, RAW264.7, COS-1, 293, A549, MDA-MB-231, A375, MSTO, rat 9L, U87, LN18, AsPC-1, Pan89, Colo357, COS-1, Vero, HeLa, Y79, RPE-J, Chang C.  - Mice | Cancer, infectious Diseases, metabolic disorders, ocular disorders | Endocytosis, endocytosis-independent | 3912,45 | 0 | 25 | 12,68 | 7 | 7 | 0,9 | -3,06 | 47 | [202,367,405,674-677] |
| VPMLK | VPMLK | NPs | - HeLa | Cancer |  | 586,82 | 0 | 60 | 10,28 | 1 | 1 | -0,3 | 36,2 | 20 | [413] |
| VT5 | DPKGDPKGVTVTVTVTVTGKGDPKPD | Proteins | - AEC |  | Endocytosis | 2609,06 | 0 | 19,23 | 7,03 | 0 | 0 | 0,6 | -1,96 | 31 | [678] |
| W4R3K | FWWWWRRRK |  | - CCRF-CEM, HT-29, MDA-MB-48, SK-OV-3 | Cancer |  | 1506,82 | 22760 | 55,56 | 12,82 | 4 | 4 | -0,5 | 46,67 | 44 | [679] |
| W4R8 | WWWWRRRRRRRR |  | - CHO-K1 - Lipid vesicles |  | Endocytosis | 2012,41 | 22760 | 33,33 | 13,53 | 8 | 8 | 0,9 | 23 | 67 | [565] |
| W5R4K | FWWWWWRRRRK |  | - CCRF-CEM, HT-29, MDA-MB-48, SK-OV-3 | Cancer |  | 1849,23 | 28450 | 54,55 | 12,97 | 5 | 5 | -0,4 | 45,73 | 45 | [679] |
| Aca, Aminocaproic acid; AEC, Aortic endothelial cells; Ahx, 6-aminohexanoic acid; Aib, 2-aminoisobutyric acid; B-ALL, B acute lymphoblastic leukemia; BBMVEC, Bovine brain microvascular endothelial cells; BCECs, Brain capillary endothelial cells; BHK, Baby hamster kidney; BMB, 3,5-bis(mercaptomethyl)benzoyl; BMDCs, Bone marrow-derived dendritic cells; BMEC, Brain microvascular endothelial cells; Cha, Cyclohexylalanine; CHO, Chinese hamster ovary; Dap, Diaminopimelic acid; DC2.4, Mouse dendritic cells; DMECs, Dermal microvessel endothelial cells; Dmt, *N,N*-Dimethyltryptamine; ES, Embryonic stem cells; F2, Diphenyl; GICs, Glioma-initiating cells; HASMCs, primary human aortic smooth muscle cells; HCE, Human corneal epithelial; HCEC, Human corneal epithelial cells; HCM, Human cardiac myocytes; HEK, Human embryonic kidney; HEL, Human erythroleukemia cell; Hex, Hexyl; hfRPE, Human fetal retinal pigment epithelial cells; hMSCs, Human mesenchymal stem cells; HMVEC, Human lung microvascular endothelial cells; HUVECs, Human umbilical vein endothelial cells; MDCK, Madin-Darby canine kidney; Met, Methylation; Nap, Naphthylalanine; NHC, Human conjunctival epithelial cells; NHDF, Normal human dermal fibroblasts; NPs, Nanoparticles; RAECM, Rat alveolar epithelial cell monolayers; SDHCEC, Spontaneously derived human corneal epithelial cells; Sip, Silaproline; X, Cyclohexylalanine; , Naphthylalanone; - No name attributed; * Different peptides with similar designations; | | | | | | | | | | | | | | | |

**Table S3. Features of published cell-penetrating peptides (cont.).**

| **Peptide** | **Typical sequence** | **Main cargoes** | **Cell model** | **Diseases** | **Proposed translocation** | **Physicochemical properties** | | | | | | | | | **Ref.** |
| --- | --- | --- | --- | --- | --- | --- | --- | --- | --- | --- | --- | --- | --- | --- | --- |
|  |  |  |  |  |  | **Molecular weight**  **(g/mol)** | **Extinction coefficient**  **(M^-1^.cm^-1^)** | **Hydrophobic**  **(%)** | **Isoelectric point** | **Net charge**  **(pH 7)** | **Charge** | **Average hydrophobicity** | **Hydrophobicity**  **(pH 7)** | **Ratio hydrophilic residues**  **(%)** |  |
| WLRCPP | WLRRIKAWLRRIKA | Proteins | - THP-1 human monocytes | Inflammatory disorders |  | 1866,41 | 11380 | 57,14 | 12,97 | 6 | 6 | 0,2 | 40,86 | 43 | [680] |
| YARACPP | YARAAARQARA | Proteins | - THP-1 human monocytes | Inflammatory disorders |  | 1204,38 | 1280 | 63,64 | 12,18 | 3 | 3 | 0,4 | 23,36 | 36 | [681] |
| YDEGE | YDEEGGGE | Proteins, nucleic acids | - HeLa, CHO |  | Macropinocytosis | 854,82 | 1280 | 12,5 | 2,79 | -4 | -4 | 1,2 | -10,62 | 50 | [311] |
| YTA2 | YTAIAWVKAFIRKLRK | Small drugs | - MDA-MB-231, MCF-7 | Cancer |  | 1964,51 | 6970 | 62,5 | 11,71 | 5 | 5 | -0,1 | 41,88 | 31 | [682] |
| YTA4 | IAWVKAFIRKLRKGPLG | NPs, small drugs | - MDA-MB-231, MCF-7  - Lipid vesicles | Cancer |  | 1953,55 | 5690 | 52,94 | 12,54 | 5 | 5 | 0 | 35,71 | 29 | [682,683] |
| YY23R1 | APPLPPRNRGEDASPEELSRYYRSLRHYLNLVTRQRY | Proteins | - HeLa |  | Endocytosis | 4472,18 | 5120 | 32,43 | 10,27 | 3,11 | 4 | 0,3 | 9,41 | 46 | [153] |
| YY24R1 | APPLPPRNRGEDASPRELSRYYRSLRHYLNLVTRQRY | Proteins | - HeLa |  | Endocytosis | 4499,25 | 5120 | 32,43 | 11,12 | 5,11 | 6 | 0,3 | 9,86 | 46 | [153] |
| YY25R1 | APPLPPRNRGEDASPRELRRYYRSLRHYLNLVTRQRY | Proteins | - HeLa |  | Endocytosis | 4568,36 | 5120 | 32,43 | 11,51 | 6,11 | 7 | 0,4 | 9,62 | 46 | [153] |
| Aca, Aminocaproic acid; AEC, Aortic endothelial cells; Ahx, 6-aminohexanoic acid; Aib, 2-aminoisobutyric acid; B-ALL, B acute lymphoblastic leukemia; BBMVEC, Bovine brain microvascular endothelial cells; BCECs, Brain capillary endothelial cells; BHK, Baby hamster kidney; BMB, 3,5-bis(mercaptomethyl)benzoyl; BMDCs, Bone marrow-derived dendritic cells; BMEC, Brain microvascular endothelial cells; Cha, Cyclohexylalanine; CHO, Chinese hamster ovary; Dap, Diaminopimelic acid; DC2.4, Mouse dendritic cells; DMECs, Dermal microvessel endothelial cells; Dmt, *N,N*-Dimethyltryptamine; ES, Embryonic stem cells; F2, Diphenyl; GICs, Glioma-initiating cells; HASMCs, primary human aortic smooth muscle cells; HCE, Human corneal epithelial; HCEC, Human corneal epithelial cells; HCM, Human cardiac myocytes; HEK, Human embryonic kidney; HEL, Human erythroleukemia cell; Hex, Hexyl; hfRPE, Human fetal retinal pigment epithelial cells; hMSCs, Human mesenchymal stem cells; HMVEC, Human lung microvascular endothelial cells; HUVECs, Human umbilical vein endothelial cells; MDCK, Madin-Darby canine kidney; Met, Methylation; Nap, Naphthylalanine; NHC, Human conjunctival epithelial cells; NHDF, Normal human dermal fibroblasts; NPs, Nanoparticles; RAECM, Rat alveolar epithelial cell monolayers; SDHCEC, Spontaneously derived human corneal epithelial cells; Sip, Silaproline; X, Cyclohexylalanine; , Naphthylalanone; - No name attributed; * Different peptides with similar designations; | | | | | | | | | | | | | | | |

**Table S4. Limits selected for each parameter using the BBB peptide shuttle database.**

| **Physicochemical property** | **Limits** | |
| --- | --- | --- |
|  | **Minimum** | **Maximum** |
| Molecular weight (g.mol^-1^) | 852.0 | 3065.0 |
| Extinction coefficient (M^-1^.cm^-1^) | 0.0 | 7786.0 |
| Hydrophobic nature (%) | 18.8 | 50.0 |
| Isoelectric point | 8.0 | 13.3 |
| Net charge (pH 7.0) | -1.0 | 4.7 |
| Charge | 0.0 | 6.0 |
| Average hydrophobicity | -0.6 | 0.8 |
| Hydrophobicity (pH 7.0) | 5.4 | 34.6 |
| Hydrophilic residues ratio (%) | 17.0 | 56.0 |


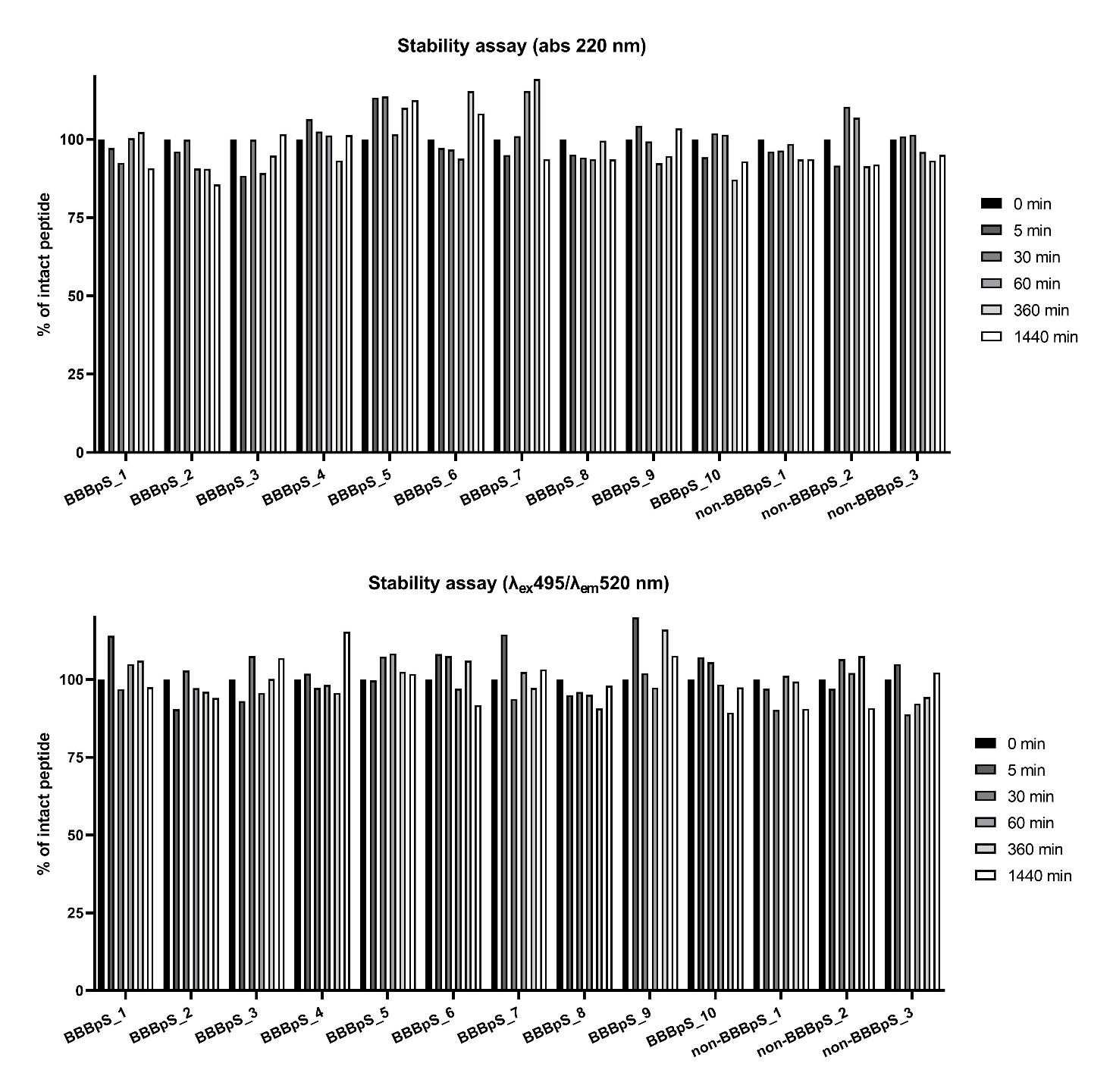


A

B

**Figure S1. Stability of peptides in culturing conditions.** 500 µM 5(6)-CF-peptides solution in DMEM:F12 without phenol-red (Gibco/Thermo Fischer (USA) were incubated at 37°C and 5% CO2. Then, 150 µl aliquots were taken at different timepoints and analyzed by analytical RP-HPLC using linear gratients of solvent B (0.1% TFA in ACN) into solvent A (0.1% TFA in H2O). The detection was made using a (**A**) SPD-M40 PDA detection (λ 220 nm) and (**B**) RF-20Axs Spectrofluorometric detector (λ_ex_ 495/λ_em_ 520 nm) (Shimadzu, Japan). Percent of intact peptide was calculated by peak integration, expressed as percent of the amount at t0.


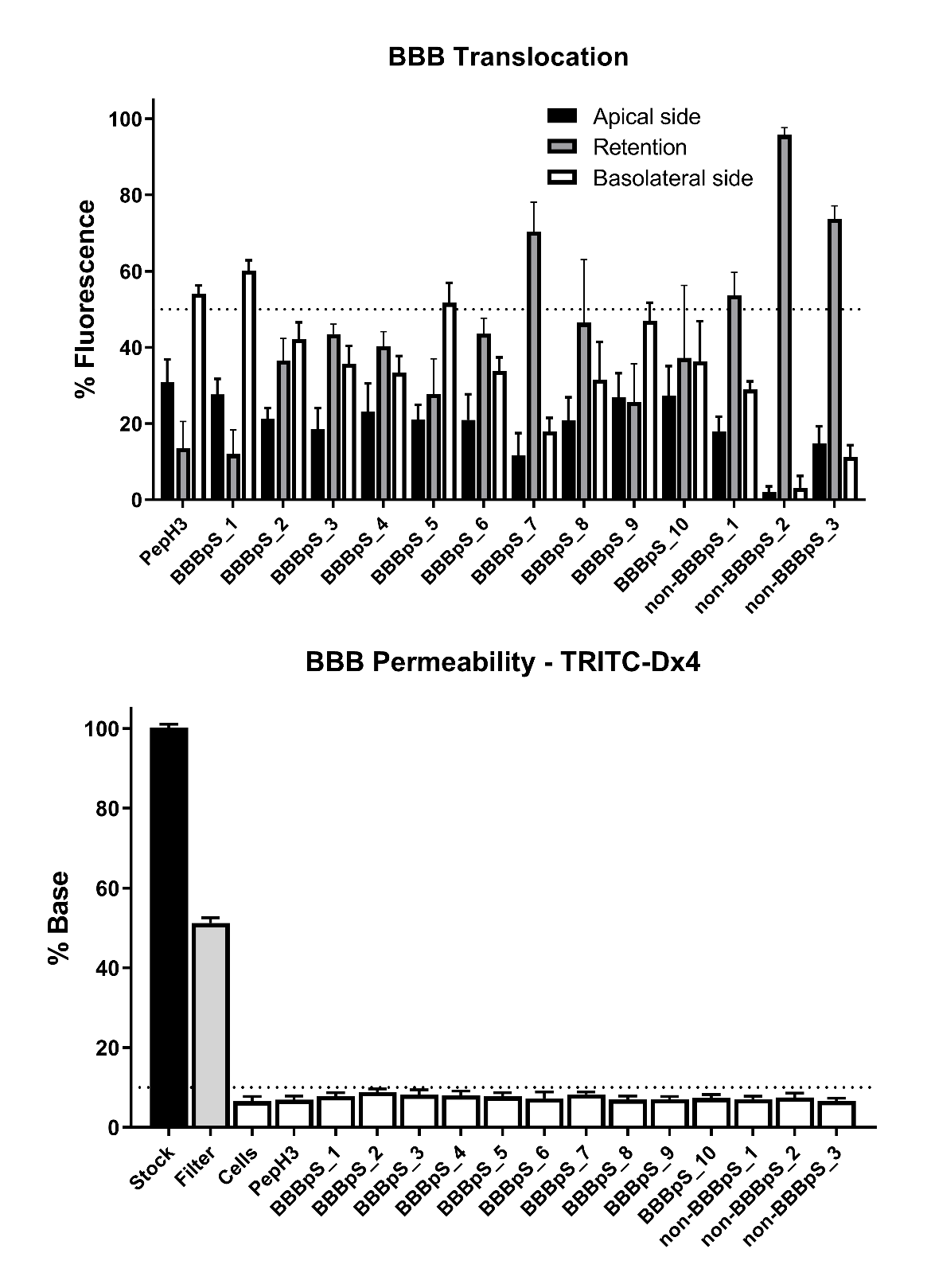


A

B

**Figure S2.** **Translocation of 5(6)-peptides across an in vitro BBB model and the TRITX-Dx4 permeability study. (A)** Percentage of translocation of 5(6)-CF-peptides (5.0 µM). **(B)** Fluorescence intensity of TRITC-Dx4 as a measured of BBB integrity after peptide exposure after translocation assay. The values were obtained from triplicates of three independent experiments. PepH3 was used as a BBBpS control. Error bars, S.D.


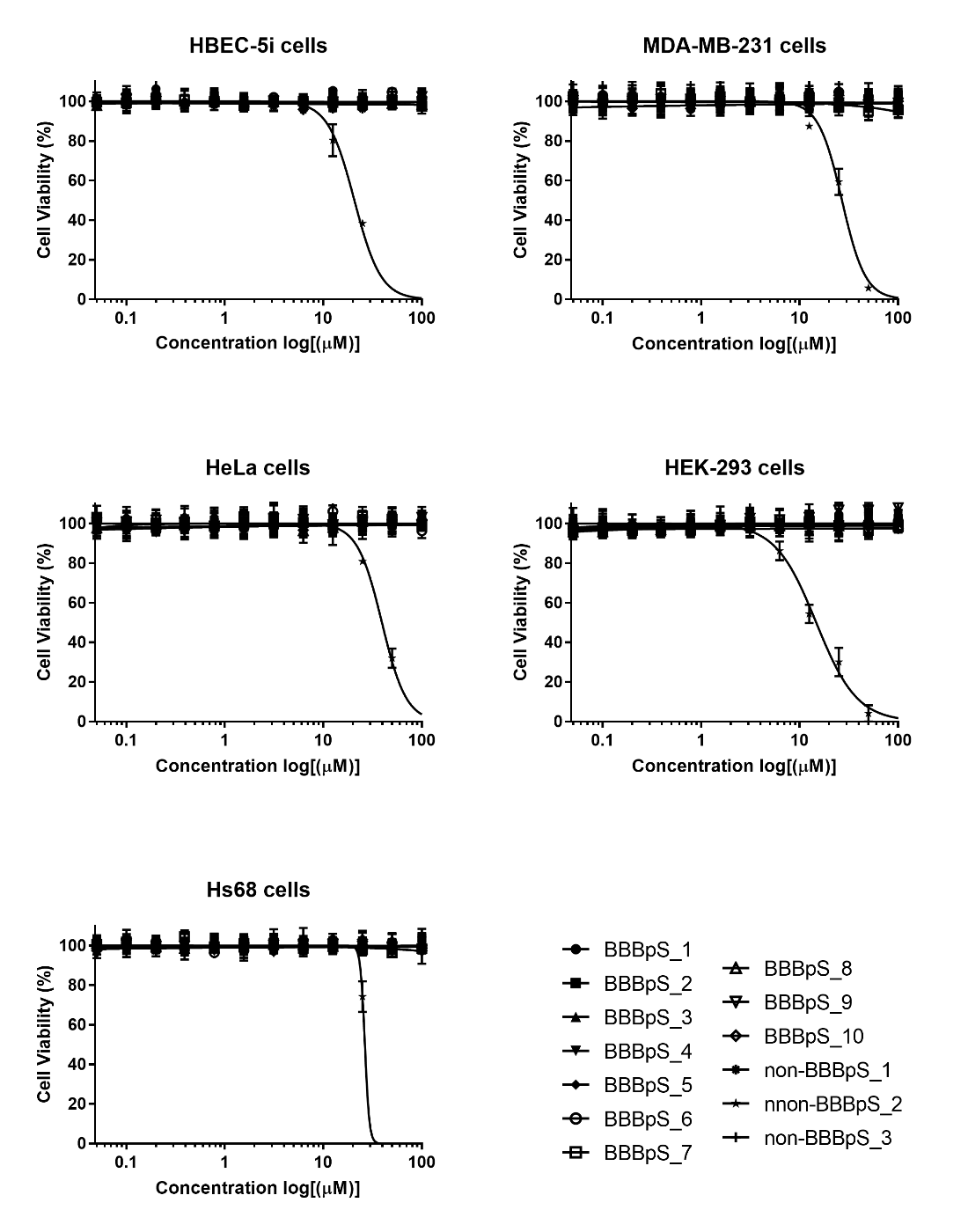


**Figure S3.** **in vitro cytotoxicity of all peptides towards a panel of different cell lines**. HBEC-5i, Hs68, HeLa, MDA-MB-231, and HEK-293 cells treated with different concentrations of peptides (0.05 – 100.0 µM) for 24h. The percentage of viable cells was determined using CellTiter-Blue® reagent assay. The values were obtained from triplicates of three independent experiments. Error bars, S.D.


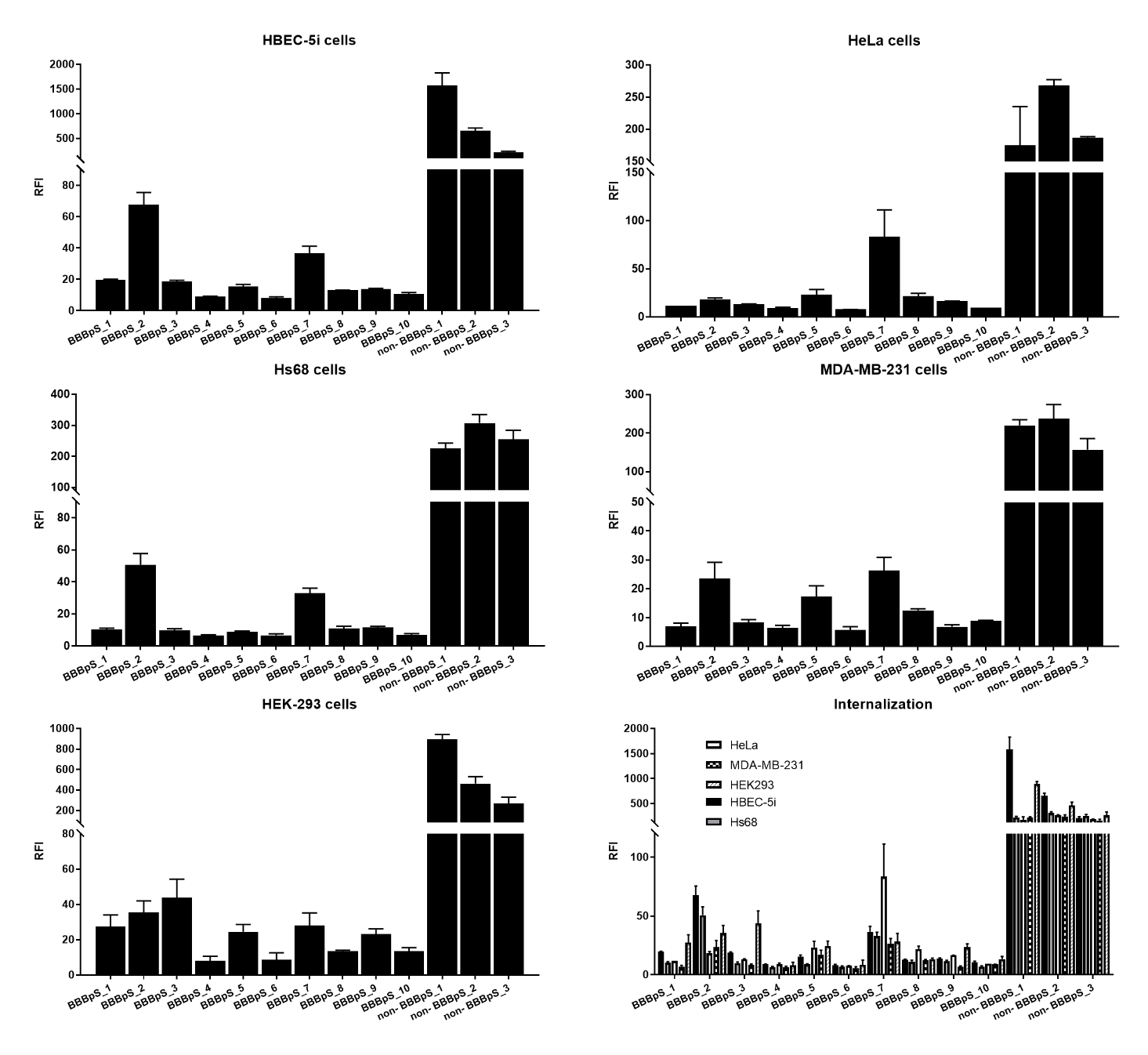


**Figure S4.** **in vitro internalization of all 5(6)-peptides towards a panel of different cell lines.** HBEC-5i, Hs68, HeLa, MDA-MB-231, and HEK-293 cells exposed to 5(6)-CF-peptides (5.0 µM) for 24h. The relative fluorescence intensity (RFI) was determined using flow cytometry. The values were obtained from triplicates of three independent experiments. Error bars, S.D.

**References**

1. Majerova, P.; Hanes, J.; Olesova, D.; Sinsky, J.; Pilipcinec, E.; Kovac, A. Novel Blood–Brain Barrier Shuttle Peptides Discovered through the Phage Display Method. *Molecules* **2020**, *25*.

2. Lin, T.; Zhao, P.; Jiang, Y.; Tang, Y.; Jin, H.; Pan, Z.; He, H.; Yang, V.C.; Huang, Y. Blood–Brain-Barrier-Penetrating Albumin Nanoparticles for Biomimetic Drug Delivery via Albumin-Binding Protein Pathways for Antiglioma Therapy. *ACS Nano* **2016**, *10*, 9999-10012, doi:10.1021/acsnano.6b04268.

3. Urich, E.; Schmucki, R.; Ruderisch, N.; Kitas, E.; Certa, U.; Jacobsen, H.; Schweitzer, C.; Bergadano, A.; Ebeling, M.; Loetscher, H., et al. Cargo Delivery into the Brain by in vivo identified Transport Peptides. *Scientific reports* **2015**, *5*, 14104-14104, doi:10.1038/srep14104.

4. Di Mauro, P.P.; Cascante, A.; Brugada Vilà, P.; Gómez-Vallejo, V.; Llop, J.; Borrós, S. Peptide-functionalized and high drug loaded novel nanoparticles as dual-targeting drug delivery system for modulated and controlled release of paclitaxel to brain glioma. *International Journal of Pharmaceutics* **2018**, *553*, 169-185, doi:<https://doi.org/10.1016/j.ijpharm.2018.10.022>.

5. Endo-Takahashi, Y.; Ooaku, K.; Ishida, K.; Suzuki, R.; Maruyama, K.; Negishi, Y. Preparation of Angiopep-2 Peptide-Modified Bubble Liposomes for Delivery to the Brain. *Biological and Pharmaceutical Bulletin* **2016**, *39*, 977-983, doi:10.1248/bpb.b15-00994.

6. Demeule, M.; Régina, A.; Ché, C.; Poirier, J.; Nguyen, T.; Gabathuler, R.; Castaigne, J.-P.; Béliveau, R. Identification and Design of Peptides as a New Drug Delivery System for the Brain. *Journal of Pharmacology and Experimental Therapeutics* **2008**, *324*, 1064, doi:10.1124/jpet.107.131318.

7. Wang, L.; Hao, Y.; Li, H.; Zhao, Y.; Meng, D.; Li, D.; Shi, J.; Zhang, H.; Zhang, Z.; Zhang, Y. Co-delivery of doxorubicin and siRNA for glioma therapy by a brain targeting system: angiopep-2-modified poly(lactic-co-glycolic acid) nanoparticles. *Journal of Drug Targeting* **2015**, *23*, 832-846, doi:10.3109/1061186X.2015.1025077.

8. Regina, A.; Demeule, M.; Tripathy, S.; Lord-Dufour, S.; Currie, J.-C.; Iddir, M.; Annabi, B.; Castaigne, J.-P.; Lachowicz, J.E. ANG4043, a Novel Brain-Penetrant Peptide–mAb Conjugate, Is Efficacious against HER2-Positive Intracranial Tumors in Mice. *Mol Cancer Ther* **2014**, *9*.

9. Hoyos-Ceballos, P.G.; Ruozi, B.; Ottonelli, I.; Da Ros, F.; Vandelli, A.M.; Forni, F.; Daini, E.; Vilella, A.; Zoli, M.; Tosi, G., et al. PLGA-PEG-ANG-2 Nanoparticles for Blood–Brain Barrier Crossing: Proof-of-Concept Study. *Pharmaceutics* **2020**, *12*, doi:10.3390/pharmaceutics12010072.

10. Böckenhoff, A.; Cramer, S.; Wölte, P.; Knieling, S.; Wohlenberg, C.; Gieselmann, V.; Galla, H.-J.; Matzner, U. Comparison of Five Peptide Vectors for Improved Brain Delivery of the Lysosomal Enzyme Arylsulfatase A. *The Journal of Neuroscience* **2014**, *34*, 3122, doi:10.1523/JNEUROSCI.4785-13.2014.

11. Srimanee, A.; Regberg, J.; Hallbrink, M.; Kurrikoff, K.; Veiman, K.-L.; Vajragupta, O.; Langel, Ü. Peptide-Based Delivery of Oligonucleotides Across Blood–Brain Barrier Model. *International Journal of Peptide Research and Therapeutics* **2014**, *20*, 169-178, doi:10.1007/s10989-013-9378-4.

12. Thomas, F.C.; Taskar, K.; Rudraraju, V.; Goda, S.; Thorsheim, H.R.; Gaasch, J.A.; Mittapalli, R.K.; Palmieri, D.; Steeg, P.S.; Lockman, P.R., et al. Uptake of ANG1005, A Novel Paclitaxel Derivative, Through the Blood-Brain Barrier into Brain and Experimental Brain Metastases of Breast Cancer. *Pharmaceutical Research* **2009**, *26*, 2486-2494, doi:10.1007/s11095-009-9964-5.

13. Li, F.; Tang, S.-C. Targeting metastatic breast cancer with ANG1005, a novel peptide-paclitaxel conjugate that crosses the blood-brain-barrier (BBB). *Genes & diseases* **2017**, *4*, 1-3, doi:10.1016/j.gendis.2017.01.004.

14. Beard, R.; Gaboriau, D.C.A.; Gee, A.D.; Tate, E.W. Chemical biology tools for probing transcytosis at the blood–brain barrier. *Chemical Science* **2019**, *10*, 10772-10778, doi:10.1039/C9SC04024B.

15. Ché, C.; Yang, G.; Thiot, C.; Lacoste, M.-C.; Currie, J.-C.; Demeule, M.; Régina, A.; Béliveau, R.; Castaigne, J.-P. New Angiopep-Modified Doxorubicin (ANG1007) and Etoposide (ANG1009) Chemotherapeutics With Increased Brain Penetration. *Journal of Medicinal Chemistry* **2010**, *53*, 2814-2824, doi:10.1021/jm9016637.

16. Régina, A.; Demeule, M.; Ché, C.; Lavallée, I.; Poirier, J.; Gabathuler, R.; Béliveau, R.; Castaigne, J.P. Antitumour activity of ANG1005, a conjugate between paclitaxel and the new brain delivery vector Angiopep-2. *British Journal of Pharmacology* **2008**, *155*, 185-197, doi:10.1038/bjp.2008.260.

17. Demeule, M.; Beaudet, N.; Régina, A.; Besserer-Offroy, É.; Murza, A.; Tétreault, P.; Belleville, K.; Ché, C.; Larocque, A.; Thiot, C., et al. Conjugation of a brain-penetrant peptide with neurotensin provides antinociceptive properties. *The Journal of Clinical Investigation* **2014**, *124*, 1199-1213, doi:10.1172/JCI70647.

18. Bertrand, Y.; Currie, J.C.; Poirier, J.; Demeule, M.; Abulrob, A.; Fatehi, D.; Stanimirovic, D.; Sartelet, H.; Castaigne, J.P.; Béliveau, R. Influence of glioma tumour microenvironment on the transport of ANG1005 via low-density lipoprotein receptor-related protein 1. *British Journal of Cancer* **2011**, *105*, 1697-1707, doi:10.1038/bjc.2011.427.

19. Thomas, A.; Pommier, Y. Targeting Topoisomerase I in the Era of Precision Medicine. *Clinical Cancer Research* **2019**, 10.1158/1078-0432.CCR-19-1089, doi:10.1158/1078-0432.CCR-19-1089.

20. Yang, Z.-Z.; Li, J.-Q.; Wang, Z.-Z.; Dong, D.-W.; Qi, X.-R. Tumor-targeting dual peptides-modified cationic liposomes for delivery of siRNA and docetaxel to gliomas. *Biomaterials* **2014**, *35*, 5226-5239, doi:<https://doi.org/10.1016/j.biomaterials.2014.03.017>.

21. Ren, J.; Shen, S.; Wang, D.; Xi, Z.; Guo, L.; Pang, Z.; Qian, Y.; Sun, X.; Jiang, X. The targeted delivery of anticancer drugs to brain glioma by PEGylated oxidized multi-walled carbon nanotubes modified with angiopep-2. *Biomaterials* **2012**, *33*, 3324-3333, doi:<https://doi.org/10.1016/j.biomaterials.2012.01.025>.

22. Yan, H.; Wang, L.; Wang, J.; Weng, X.; Lei, H.; Wang, X.; Jiang, L.; Zhu, J.; Lu, W.; Wei, X., et al. Two-Order Targeted Brain Tumor Imaging by Using an Optical/Paramagnetic Nanoprobe across the Blood Brain Barrier. *ACS Nano* **2012**, *6*, 410-420, doi:10.1021/nn203749v.

23. Yan, H.; Wang, J.; Yi, P.; Lei, H.; Zhan, C.; Xie, C.; Feng, L.; Qian, J.; Zhu, J.; Lu, W., et al. Imaging brain tumor by dendrimer-based optical/paramagnetic nanoprobe across the blood-brain barrier. *Chemical Communications* **2011**, *47*, 8130-8132, doi:10.1039/C1CC12007G.

24. Gao, X.; Qian, J.; Zheng, S.; Xiong, Y.; Man, J.; Cao, B.; Wang, L.; Ju, S.; Li, C. Up-regulating Blood Brain Barrier Permeability of Nanoparticles via Multivalent Effect. *Pharmaceutical Research* **2013**, *30*, 2538-2548, doi:10.1007/s11095-013-1004-9.

25. Huang, S.; Li, J.; Han, L.; Liu, S.; Ma, H.; Huang, R.; Jiang, C. Dual targeting effect of Angiopep-2-modified, DNA-loaded nanoparticles for glioma. *Biomaterials* **2011**, *32*, 6832-6838, doi:<https://doi.org/10.1016/j.biomaterials.2011.05.064>.

26. Huang, R.; Ma, H.; Guo, Y.; Liu, S.; Kuang, Y.; Shao, K.; Li, J.; Liu, Y.; Han, L.; Huang, S., et al. Angiopep-Conjugated Nanoparticles for Targeted Long-Term Gene Therapy of Parkinson’s Disease. *Pharmaceutical Research* **2013**, *30*, 2549-2559, doi:10.1007/s11095-013-1005-8.

27. Gao, H.; Zhang, S.; Cao, S.; Yang, Z.; Pang, Z.; Jiang, X. Angiopep-2 and Activatable Cell-Penetrating Peptide Dual-Functionalized Nanoparticles for Systemic Glioma-Targeting Delivery. *Molecular Pharmaceutics* **2014**, *11*, 2755-2763, doi:10.1021/mp500113p.

28. Xin, H.; Jiang, X.; Gu, J.; Sha, X.; Chen, L.; Law, K.; Chen, Y.; Wang, X.; Jiang, Y.; Fang, X. Angiopep-conjugated poly(ethylene glycol)-co-poly(ε-caprolactone) nanoparticles as dual-targeting drug delivery system for brain glioma. *Biomaterials* **2011**, *32*, 4293-4305, doi:<https://doi.org/10.1016/j.biomaterials.2011.02.044>.

29. Xin, H.; Sha, X.; Jiang, X.; Zhang, W.; Chen, L.; Fang, X. Anti-glioblastoma efficacy and safety of paclitaxel-loading Angiopep-conjugated dual targeting PEG-PCL nanoparticles. *Biomaterials* **2012**, *33*, 8167-8176, doi:10.1016/j.biomaterials.2012.07.046.

30. Shen, J.; Zhan, C.; Xie, C.; Meng, Q.; Gu, B.; Li, C.; Zhang, Y.; Lu, W. Poly(ethylene glycol)-block-poly(d,l-lactide acid) micelles anchored with angiopep-2 for brain-targeting delivery. *Journal of Drug Targeting* **2011**, *19*, 197-203, doi:10.3109/1061186X.2010.483517.

31. Ying, X.; Wang, Y.; Liang, J.; Yue, J.; Xu, C.; Lu, L.; Xu, Z.; Gao, J.; Du, Y.; Chen, Z. Angiopep-Conjugated Electro-Responsive Hydrogel Nanoparticles: Therapeutic Potential for Epilepsy. *Angewandte Chemie International Edition* **2014**, *53*, 12436-12440, doi:10.1002/anie.201403846.

32. Ruan, S.; Qian, J.; Shen, S.; Chen, J.; Zhu, J.; Jiang, X.; He, Q.; Yang, W.; Gao, H. Fluorescent Carbonaceous Nanodots for Noninvasive Glioma Imaging after Angiopep-2 Decoration. *Bioconjugate Chemistry* **2014**, *25*, 2252-2259, doi:10.1021/bc500474p.

33. Ruan, S.; Yuan, M.; Zhang, L.; Hu, G.; Chen, J.; Cun, X.; Zhang, Q.; Yang, Y.; He, Q.; Gao, H. Tumor microenvironment sensitive doxorubicin delivery and release to glioma using angiopep-2 decorated gold nanoparticles. *Biomaterials* **2015**, *37*, 425-435, doi:<https://doi.org/10.1016/j.biomaterials.2014.10.007>.

34. Morales-Zavala, F.; Arriagada, H.; Hassan, N.; Velasco, C.; Riveros, A.; Álvarez, A.R.; Minniti, A.N.; Rojas-Silva, X.; Muñoz, L.L.; Vasquez, R., et al. Peptide multifunctionalized gold nanorods decrease toxicity of β-amyloid peptide in a Caenorhabditis elegans model of Alzheimer's disease. *Nanomedicine: Nanotechnology, Biology and Medicine* **2017**, *13*, 2341-2350, doi:<https://doi.org/10.1016/j.nano.2017.06.013>.

35. Wei, X.; Zhan, C.; Chen, X.; Hou, J.; Xie, C.; Lu, W. Retro-Inverso Isomer of Angiopep-2: A Stable d-Peptide Ligand Inspires Brain-Targeted Drug Delivery. *Molecular Pharmaceutics* **2014**, *11*, 3261-3268, doi:10.1021/mp500086e.

36. Oller-Salvia, B.; Teixidó, M.; Giralt, E. From venoms to BBB shuttles: Synthesis and blood–brain barrier transport assessment of apamin and a nontoxic analog. *Peptide Science* **2013**, *100*, 675-686, doi:10.1002/bip.22257.

37. Wu, J.; Jiang, H.; Bi, Q.; Luo, Q.; Li, J.; Zhang, Y.; Chen, Z.; Li, C. Apamin-Mediated Actively Targeted Drug Delivery for Treatment of Spinal Cord Injury: More Than Just a Concept. *Molecular Pharmaceutics* **2014**, *11*, 3210-3222, doi:10.1021/mp500393m.

38. Spencer, B.J.; Vera, I.M. Targeted delivery of proteins across the blood–brain barrier. *PNAS* **2007**, *104*, 7594-7599.

39. Sorrentino, N.C.; D'Orsi, L.; Sambri, I.; Nusco, E.; Monaco, C.; Spampanato, C.; Polishchuk, E.; Saccone, P.; De Leonibus, E.; Ballabio, A., et al. A highly secreted sulphamidase engineered to cross the blood-brain barrier corrects brain lesions of mice with mucopolysaccharidoses type IIIA. *EMBO Molecular Medicine* **2013**, *5*, 675-690, doi:10.1002/emmm.201202083.

40. Wang, D.; El-Amouri, S.S.; Dai, M.; Kuan, C.-Y.; Hui, D.Y.; Brady, R.O.; Pan, D. Engineering a lysosomal enzyme for brain delivery. *PNAS* **2013**, *110*, 2999-3004.

41. Re, F.; Cambianica, I.; Zona, C.; Sesana, S.; Gregori, M.; Rigolio, R.; La Ferla, B.; Nicotra, F.; Forloni, G.; Cagnotto, A., et al. Functionalization of liposomes with ApoE-derived peptides at different density affects cellular uptake and drug transport across a blood-brain barrier model. *Nanomedicine: Nanotechnology, Biology and Medicine* **2011**, *7*, 551-559, doi:<https://doi.org/10.1016/j.nano.2011.05.004>.

42. Re, F.; Cambianica, I.; Sesana, S.; Salvati, E.; Cagnotto, A.; Salmona, M.; Couraud, P.-O.; Moghimi, S.M.; Masserini, M.; Sancini, G. Functionalization with ApoE-derived peptides enhances the interaction with brain capillary endothelial cells of nanoliposomes binding amyloid-beta peptide. *Journal of biotechnology* **2011**, *156*, 341-346, doi:10.1016/j.jbiotec.2011.06.037.

43. Portioli, C.; Bovi, M.; Benati, D.; Donini, M.; Perduca, M.; Romeo, A.; Dusi, S.; Monaco, H.L.; Bentivoglio, M. Novel functionalization strategies of polymeric nanoparticles as carriers for brain medications. *Journal of Biomedical Materials Research Part A* **2017**, *105*, 847-858, doi:10.1002/jbm.a.35961.

44. Sarkar, G.; Curran, G.L.; Sarkaria, J.N.; Lowe, V.J.; Jenkins, R.B. Peptide Carrier-Mediated Non-Covalent Delivery of Unmodified Cisplatin, Methotrexate and Other Agents via Intravenous Route to the Brain. *PLOS ONE* **2014**, *9*, e97655, doi:10.1371/journal.pone.0097655.

45. Yin, T.; Yang, L.; Liu, Y.; Zhou, X.; Sun, J.; Liu, J. Sialic acid (SA)-modified selenium nanoparticles coated with a high blood–brain barrier permeability peptide-B6 peptide for potential use in Alzheimer’s disease. *Acta Biomaterialia* **2015**, *25*, 172-183, doi:<https://doi.org/10.1016/j.actbio.2015.06.035>.

46. Liu, Z.; Gao, X.; Kang, T.; Jiang, M.; Miao, D.; Gu, G.; Hu, Q.; Song, Q.; Yao, L.; Tu, Y., et al. B6 Peptide-Modified PEG-PLA Nanoparticles for Enhanced Brain Delivery of Neuroprotective Peptide. *Bioconjugate Chemistry* **2013**, *24*, 997-1007, doi:10.1021/bc400055h.

47. Xia, H.; Anderson, B.; Mao, Q.; Davidson, B.L. Recombinant Human Adenovirus: Targeting to the Human Transferrin Receptor Improves Gene Transfer to Brain Microcapillary Endothelium. *Journal of Virology* **2000**, *74*, 11359, doi:10.1128/JVI.74.23.11359-11366.2000.

48. Javed, H.; Menon, S.A.; Al-Mansoori, K.M.; Al-Wandi, A.; Majbour, N.K.; Ardah, M.T.; Varghese, S.; Vaikath, N.N.; Haque, M.E.; Azzouz, M., et al. Development of Nonviral Vectors Targeting the Brain as a Therapeutic Approach For Parkinson's Disease and Other Brain Disorders. *Molecular therapy : the journal of the American Society of Gene Therapy* **2016**, *24*, 746-758, doi:10.1038/mt.2015.232.

49. Fan, X.; Venegas, R.; Fey, R.; van der Heyde, H.; Bernard, M.A.; Lazarides, E.; Woods, C.M. An in vivo approach to structure activity relationship analysis of peptide ligands. *Pharmaceutical research* **2007**, *24*, 868-879, doi:10.1007/s11095-007-9238-z.

50. Toome, K.; Willmore, A.-M.A.; Paiste, P.; Tobi, A.; Sugahara, K.N.; Kirsimäe, K.; Ruoslahti, E.; Braun, G.B.; Teesalu, T. Ratiometric in vivo auditioning of targeted silver nanoparticles. *Nanoscale* **2017**, *9*, 10094-10100, doi:10.1039/C7NR04056C.

51. Wei, X.; Zhan, C.; Shen, Q.; Fu, W.; Xie, C.; Gao, J.; Peng, C.; Zheng, P.; Lu, W. A D-Peptide Ligand of Nicotine Acetylcholine Receptors for Brain-Targeted Drug Delivery. *Angewandte Chemie International Edition* **2015**, *54*, 3023-3027, doi:10.1002/anie.201411226.

52. Ying, M.; Zhan, C.; Wang, S.; Yao, B.; Hu, X.; Song, X.; Zhang, M.; Wei, X.; Xiong, Y.; Lu, W. Liposome-Based Systemic Glioma-Targeted Drug Delivery Enabled by All-d Peptides. *ACS Applied Materials & Interfaces* **2016**, *8*, 29977-29985, doi:10.1021/acsami.6b10146.

53. Zhan, C.; Li, B.; Hu, L.; Wei, X.; Feng, L.; Fu, W.; Lu, W. Micelle-Based Brain-Targeted Drug Delivery Enabled by a Nicotine Acetylcholine Receptor Ligand. *Angewandte Chemie International Edition* **2011**, *50*, 5482-5485, doi:10.1002/anie.201100875.

54. Wei, X.; Gao, J.; Zhan, C.; Xie, C.; Chai, Z.; Ran, D.; Ying, M.; Zheng, P.; Lu, W. Liposome-based glioma targeted drug delivery enabled by stable peptide ligands. *Journal of Controlled Release* **2015**, *218*, 13-21, doi:<https://doi.org/10.1016/j.jconrel.2015.09.059>.

55. Huang, N.; Lu, S.; Liu, X.-G.; Zhu, J.; Wang, Y.-J.; Liu, R.-T. PLGA nanoparticles modified with a BBB-penetrating peptide co-delivering Aβ generation inhibitor and curcumin attenuate memory deficits and neuropathology in Alzheimer’s disease mice. *Oncotarget; Vol 8, No 46* **2017**.

56. Staquicini, F.I.; Ozawa, M.G.; Moya, C.A.; Driessen, W.H.P.; Barbu, E.M.; Nishimori, H.; Soghomonyan, S.; Flores, L.G., 2nd; Liang, X.; Paolillo, V., et al. Systemic combinatorial peptide selection yields a non-canonical iron-mimicry mechanism for targeting tumors in a mouse model of human glioblastoma. *The Journal of Clinical Investigation* **2011**, *121*, 161-173, doi:10.1172/JCI44798.

57. Kang, T.; Jiang, M.; Jiang, D.; Feng, X.; Yao, J.; Song, Q.; Chen, H.; Gao, X.; Chen, J. Enhancing Glioblastoma-Specific Penetration by Functionalization of Nanoparticles with an Iron-Mimic Peptide Targeting Transferrin/Transferrin Receptor Complex. *Molecular Pharmaceutics* **2015**, *12*, 2947-2961, doi:10.1021/acs.molpharmaceut.5b00222.

58. Lim, S.; Kim, W.-J.; Kim, Y.-H.; Lee, S.; Koo, J.-H.; Lee, J.-A.; Yoon, H.; Kim, D.-H.; Park, H.-J.; Kim, H.-M., et al. dNP2 is a blood–brain barrier-permeable peptide enabling ctCTLA-4 protein delivery to ameliorate experimental autoimmune encephalomyelitis. *Nature Communications* **2015**, *6*, 8244, doi:10.1038/ncomms9244.

59. Georgieva, J.V.; Brinkhuis, R.P.; Stojanov, K.; Weijers, C.A.G.M.; Zuilhof, H.; Rutjes, F.P.J.T.; Hoekstra, D.; van Hest, J.C.M.; Zuhorn, I.S. Peptide-Mediated Blood–Brain Barrier Transport of Polymersomes. *Angewandte Chemie International Edition* **2012**, *51*, 8339-8342, doi:10.1002/anie.201202001.

60. Lundin, P.; Johansson, H.; Guterstam, P.; Holm, T.; Hansen, M.; Langel, Ü.; El Andaloussi, S. Distinct Uptake Routes of Cell-Penetrating Peptide Conjugates. *Bioconjugate Chemistry* **2008**, *19*, 2535-2542, doi:10.1021/bc800212j.

61. van Rooy, I.; Cakir-Tascioglu, S.; Couraud, P.-O.; Romero, I.A.; Weksler, B.; Storm, G.; Hennink, W.E.; Schiffelers, R.M.; Mastrobattista, E. Identification of Peptide Ligands for Targeting to the Blood-Brain Barrier. *Pharmaceutical Research* **2010**, *27*, 673-682, doi:10.1007/s11095-010-0053-6.

62. Gaillard, P.J.; Appeldoorn, C.C.M.; Rip, J.; Dorland, R.; van der Pol, S.M.A.; Kooij, G.; de Vries, H.E.; Reijerkerk, A. Enhanced brain delivery of liposomal methylprednisolone improved therapeutic efficacy in a model of neuroinflammation. *Journal of controlled release : official journal of the Controlled Release Society* **2012**, *164*, 364-369, doi:10.1016/j.jconrel.2012.06.022.

63. Mdzinarishvili, A.; Sutariya, V.; Talasila, P.K.; Geldenhuys, W.J.; Sadana, P. Engineering triiodothyronine (T3) nanoparticle for use in ischemic brain stroke. *Drug Delivery and Translational Research* **2013**, *3*, 309-317, doi:10.1007/s13346-012-0117-8.

64. Lee, D.-H.; Rötger, C.; Appeldoorn, C.C.M.; Reijerkerk, A.; Gladdines, W.; Gaillard, P.J.; Linker, R.A. Glutathione PEGylated liposomal methylprednisolone (2B3-201) attenuates CNS inflammation and degeneration in murine myelin oligodendrocyte glycoprotein induced experimental autoimmune encephalomyelitis. *Journal of Neuroimmunology* **2014**, *274*, 96-101, doi:<https://doi.org/10.1016/j.jneuroim.2014.06.025>.

65. Geldenhuys, W.; Mbimba, T.; Bui, T.; Harrison, K.; Sutariya, V. Brain-targeted delivery of paclitaxel using glutathione-coated nanoparticles for brain cancers. *Journal of Drug Targeting* **2011**, *19*, 837-845, doi:10.3109/1061186X.2011.589435.

66. Lindqvist, A.; Rip, J.; Gaillard, P.J.; Björkman, S.; Hammarlund-Udenaes, M. Enhanced Brain Delivery of the Opioid Peptide DAMGO in Glutathione PEGylated Liposomes: A Microdialysis Study. *Molecular Pharmaceutics* **2013**, *10*, 1533-1541, doi:10.1021/mp300272a.

67. Rotman, M.; Welling, M.M.; Bunschoten, A.; de Backer, M.E.; Rip, J.; Nabuurs, R.J.A.; Gaillard, P.J.; van Buchem, M.A.; van der Maarel, S.M.; van der Weerd, L. Enhanced glutathione PEGylated liposomal brain delivery of an anti-amyloid single domain antibody fragment in a mouse model for Alzheimer's disease. *Journal of Controlled Release* **2015**, *203*, 40-50, doi:<https://doi.org/10.1016/j.jconrel.2015.02.012>.

68. Zheng, X.; Shao, X.; Zhang, C.; Tan, Y.; Liu, Q.; Wan, X.; Zhang, Q.; Xu, S.; Jiang, X. Intranasal H102 Peptide-Loaded Liposomes for Brain Delivery to Treat Alzheimer’s Disease. *Pharmaceutical Research* **2015**, *32*, 3837-3849, doi:10.1007/s11095-015-1744-9.

69. Tabanor, K.; Lee, P.; Kiptoo, P.; Choi, I.-Y.; Sherry, E.B.; Eagle, C.S.; Williams, T.D.; Siahaan, T.J. Brain Delivery of Drug and MRI Contrast Agent: Detection and Quantitative Determination of Brain Deposition of CPT-Glu Using LC–MS/MS and Gd-DTPA Using Magnetic Resonance Imaging. *Molecular Pharmaceutics* **2016**, *13*, 379-390, doi:10.1021/acs.molpharmaceut.5b00607.

70. Barrett, G.L.; Trieu, J.; Naim, T. The identification of leptin-derived peptides that are taken up by the brain. *Regulatory Peptides* **2009**, *155*, 55-61, doi:<https://doi.org/10.1016/j.regpep.2009.02.008>.

71. Sabbatini, M.E. Natriuretic peptides as regulatory mediators of secretory activity in the digestive system. *Regulatory peptides* **2009**, *154*, 5-15, doi:10.1016/j.regpep.2009.02.009.

72. Kralisch, S.; Weise, S.; Sommer, G.; Lipfert, J.; Lossner, U.; Bluher, M.; Stumvoll, M.; Fasshauer, M. Interleukin-1beta induces the novel adipokine chemerin in adipocytes in vitro. *Regulatory peptides* **2009**, *154*, 102-106, doi:10.1016/j.regpep.2009.02.010.

73. Liu, Y.; Li, J.; Shao, K.; Huang, R.; Ye, L.; Lou, J.; Jiang, C. A leptin derived 30-amino-acid peptide modified pegylated poly-l-lysine dendrigraft for brain targeted gene delivery. *Biomaterials* **2010**, *31*, 5246-5257, doi:<https://doi.org/10.1016/j.biomaterials.2010.03.011>.

74. Chen, L.; Zeng, D.; Xu, N.; Li, C.; Zhang, W.; Zhu, X.; Gao, Y.; Chen, P.R.; Lin, J. Blood–Brain Barrier- and Blood–Brain Tumor Barrier-Penetrating Peptide-Derived Targeted Therapeutics for Glioma and Malignant Tumor Brain Metastases. *ACS Applied Materials & Interfaces* **2019**, *11*, 41889-41897, doi:10.1021/acsami.9b14046.

75. Han, L.; Kong, D.K.; Zheng, M.-q.; Murikinati, S.; Ma, C.; Yuan, P.; Li, L.; Tian, D.; Cai, Q.; Ye, C., et al. Increased Nanoparticle Delivery to Brain Tumors by Autocatalytic Priming for Improved Treatment and Imaging. *ACS Nano* **2016**, *10*, 4209-4218, doi:10.1021/acsnano.5b07573.

76. Oller-Salvia, B.; Sánchez-Navarro, M.; Ciudad, S.; Guiu, M.; Arranz-Gibert, P.; Garcia, C.; Gomis, R.R.; Cecchelli, R.; García, J.; Giralt, E., et al. MiniAp-4: A Venom-Inspired Peptidomimetic for Brain Delivery. *Angewandte Chemie International Edition* **2016**, *55*, 572-575, doi:10.1002/anie.201508445.

77. Huang, N.; Cheng, S.; Zhang, X.; Tian, Q.; Pi, J.; Tang, J.; Huang, Q.; Wang, F.; Chen, J.; Xie, Z., et al. Efficacy of NGR peptide-modified PEGylated quantum dots for crossing the blood–brain barrier and targeted fluorescence imaging of glioma and tumor vasculature. *Nanomedicine: Nanotechnology, Biology and Medicine* **2017**, *13*, 83-93, doi:<https://doi.org/10.1016/j.nano.2016.08.029>.

78. Yao, H.; Wang, K.; Wang, Y.; Wang, S.; Li, J.; Lou, J.; Ye, L.; Yan, X.; Lu, W.; Huang, R. Enhanced blood–brain barrier penetration and glioma therapy mediated by a new peptide modified gene delivery system. *Biomaterials* **2015**, *37*, 345-352, doi:<https://doi.org/10.1016/j.biomaterials.2014.10.034>.

79. Yin, T.; Xie, W.; Sun, J.; Yang, L.; Liu, J. Penetratin Peptide-Functionalized Gold Nanostars: Enhanced BBB Permeability and NIR Photothermal Treatment of Alzheimer’s Disease Using Ultralow Irradiance. *ACS Applied Materials & Interfaces* **2016**, *8*, 19291-19302, doi:10.1021/acsami.6b05089.

80. Rousselle, C.; Clair, P.; Lefauconnier, J.-M.; Kaczorek, M.; Scherrmann, J.-M.; Temsamani, J. New Advances in the Transport of Doxorubicin through the Blood-Brain Barrier by a Peptide Vector-Mediated Strategy. *Molecular Pharmacology* **2000**, *57*, 679, doi:10.1124/mol.57.4.679.

81. Li, J.; Zhang, Q.; Pang, Z.; Wang, Y.; Liu, Q.; Guo, L.; Jiang, X. Identification of peptide sequences that target to the brain using in vivo phage display. *Amino Acids* **2012**, *42*, 2373-2381, doi:10.1007/s00726-011-0979-y.

82. Neves, V.; Aires-da-Silva, F.; Morais, M.; Gano, L.; Ribeiro, E.; Pinto, A.; Aguiar, S.; Gaspar, D.; Fernandes, C.; Correia, J.D.G., et al. Novel Peptides Derived from Dengue Virus Capsid Protein Translocate Reversibly the Blood–Brain Barrier through a Receptor-Free Mechanism. *ACS Chemical Biology* **2017**, *12*, 1257-1268, doi:10.1021/acschembio.7b00087.

83. Neves-Coelho, S.; Eleutério, R.P.; Enguita, F.J.; Neves, V.; Castanho, M.A.R.B. A New Noncanonical Anionic Peptide That Translocates a Cellular Blood-Brain Barrier Model. *Molecules (Basel, Switzerland)* **2017**, *22*, 1753, doi:10.3390/molecules22101753.

84. Marco Calvinho, C.; Javier Valle, G.; Ruben Diogo Marques da, S.; João Domingos Galamba, C.; Miguel Augusto Rico Botas, C.; David Andreu Martinez and Vera Luísa Santos, N. DPepH3, an Improved Peptide Shuttle for Receptor-independent Transport Across the Blood-Brain Barrier. *Current Pharmaceutical Design* **2020**, *26*, 1-12, doi:<http://dx.doi.org/10.2174/1381612826666200213094556>.

85. Costantino, L.; Gandolfi, F.; Tosi, G.; Rivasi, F.; Vandelli, M.A.; Forni, F. Peptide-derivatized biodegradable nanoparticles able to cross the blood-brain barrier. *Journal of controlled release : official journal of the Controlled Release Society* **2005**, *108*, 84-96, doi:10.1016/j.jconrel.2005.07.013.

86. Malcor, J.-D.; Payrot, N.; David, M.; Faucon, A.; Abouzid, K.; Jacquot, G.; Floquet, N.; Debarbieux, F.; Rougon, G.; Martinez, J., et al. Chemical Optimization of New Ligands of the Low-Density Lipoprotein Receptor as Potential Vectors for Central Nervous System Targeting. *Journal of Medicinal Chemistry* **2012**, *55*, 2227-2241, doi:10.1021/jm2014919.

87. Zhang, B.; Sun, X.; Mei, H.; Wang, Y.; Liao, Z.; Chen, J.; Zhang, Q.; Hu, Y.; Pang, Z.; Jiang, X. LDLR-mediated peptide-22-conjugated nanoparticles for dual-targeting therapy of brain glioma. *Biomaterials* **2013**, *34*, 9171-9182, doi:<https://doi.org/10.1016/j.biomaterials.2013.08.039>.

88. Chen, C.; Duan, Z.; Yuan, Y.; Li, R.; Pang, L.; Liang, J.; Xu, X.; Wang, J. Peptide-22 and Cyclic RGD Functionalized Liposomes for Glioma Targeting Drug Delivery Overcoming BBB and BBTB. *ACS Applied Materials & Interfaces* **2017**, *9*, 5864-5873, doi:10.1021/acsami.6b15831.

89. Jia, G.; Han, Y.; An, Y.; Ding, Y.; He, C.; Wang, X.; Tang, Q. NRP-1 targeted and cargo-loaded exosomes facilitate simultaneous imaging and therapy of glioma in vitro and in vivo. *Biomaterials* **2018**, *178*, 302-316, doi:<https://doi.org/10.1016/j.biomaterials.2018.06.029>.

90. Kumar, P.; Wu, H.; McBride, J.L.; Jung, K.-E.; Hee Kim, M.; Davidson, B.L.; Kyung Lee, S.; Shankar, P.; Manjunath, N. Transvascular delivery of small interfering RNA to the central nervous system. *Nature* **2007**, *448*, 39-43, doi:10.1038/nature05901.

91. Liu, Y.; Huang, R.; Han, L.; Ke, W.; Shao, K.; Ye, L.; Lou, J.; Jiang, C. Brain-targeting gene delivery and cellular internalization mechanisms for modified rabies virus glycoprotein RVG29 nanoparticles. *Biomaterials* **2009**, *30*, 4195-4202, doi:10.1016/j.biomaterials.2009.02.051.

92. dos Santos Rodrigues, B.; Arora, S.; Kanekiyo, T.; Singh, J. Efficient neuronal targeting and transfection using RVG and transferrin-conjugated liposomes. *Brain Research* **2020**, *1734*, 146738, doi:<https://doi.org/10.1016/j.brainres.2020.146738>.

93. Cook, R.L.; Householder, K.T.; Chung, E.P.; Prakapenka, A.V.; DiPerna, D.M.; Sirianni, R.W. A critical evaluation of drug delivery from ligand modified nanoparticles: Confounding small molecule distribution and efficacy in the central nervous system. *Journal of controlled release : official journal of the Controlled Release Society* **2015**, *220*, 89-97, doi:10.1016/j.jconrel.2015.10.013.

94. Zadran, S.; Akopian, G.; Zadran, H.; Walsh, J.; Baudry, M. RVG-Mediated Calpain2 Gene Silencing in the Brain Impairs Learning and Memory. *NeuroMolecular Medicine* **2013**, *15*, 74-81, doi:10.1007/s12017-012-8196-8.

95. Hwang, D.W.; Son, S.; Jang, J.; Youn, H.; Lee, S.; Lee, D.; Lee, Y.-S.; Jeong, J.M.; Kim, W.J.; Lee, D.S. A brain-targeted rabies virus glycoprotein-disulfide linked PEI nanocarrier for delivery of neurogenic microRNA. *Biomaterials* **2011**, *32*, 4968-4975, doi:<https://doi.org/10.1016/j.biomaterials.2011.03.047>.

96. Huo, H.; Gao, Y.; Wang, Y.; Zhang, J.; Wang, Z.-y.; Jiang, T.; Wang, S. Polyion complex micelles composed of pegylated polyasparthydrazide derivatives for siRNA delivery to the brain. *Journal of Colloid and Interface Science* **2015**, *447*, 8-15, doi:<https://doi.org/10.1016/j.jcis.2015.01.043>.

97. Alvarez-Erviti, L.; Seow, Y.; Yin, H.; Betts, C.; Lakhal, S.; Wood, M.J.A. Delivery of siRNA to the mouse brain by systemic injection of targeted exosomes. *Nature Biotechnology* **2011**, *29*, 341-345, doi:10.1038/nbt.1807.

98. Liu, Y.; Guo, Y.; An, S.; Kuang, Y.; He, X.; Ma, H.; Li, J.; Lv, J.; Zhang, N.; Jiang, C. Targeting Caspase-3 as Dual Therapeutic Benefits by RNAi Facilitating Brain-Targeted Nanoparticles in a Rat Model of Parkinson’s Disease. *PLOS ONE* **2013**, *8*, e62905, doi:10.1371/journal.pone.0062905.

99. Gong, C.; Li, X.; Xu, L.; Zhang, Y.-H. Target delivery of a gene into the brain using the RVG29-oligoarginine peptide. *Biomaterials* **2012**, *33*, 3456-3463, doi:<https://doi.org/10.1016/j.biomaterials.2011.12.017>.

100. Díaz-Perlas, C.; Sánchez-Navarro, M.; Oller-Salvia, B.; Moreno, M.; Teixidó, M.; Giralt, E. Phage display as a tool to discover blood–brain barrier (BBB)-shuttle peptides: panning against a human BBB cellular model. *Peptide Science* **2017**, *108*, e22928, doi:10.1002/bip.22928.

101. Smith, M.W.; Al-Jayyoussi, G.; Gumbleton, M. Peptide sequences mediating tropism to intact blood–brain barrier: An in vivo biodistribution study using phage display. *Peptides* **2012**, *38*, 172-180, doi:<https://doi.org/10.1016/j.peptides.2012.06.019>.

102. Bi, Y.; Liu, L.; Lu, Y.; Sun, T.; Shen, C.; Chen, X.; Chen, Q.; An, S.; He, X.; Ruan, C., et al. T7 Peptide-Functionalized PEG-PLGA Micelles Loaded with Carmustine for Targeting Therapy of Glioma. *ACS Applied Materials & Interfaces* **2016**, *8*, 27465-27473, doi:10.1021/acsami.6b05572.

103. Cui, Y.; Zhang, M.; Zeng, F.; Jin, H.; Xu, Q.; Huang, Y. Dual-Targeting Magnetic PLGA Nanoparticles for Codelivery of Paclitaxel and Curcumin for Brain Tumor Therapy. *ACS applied materials & interfaces* **2016**, *8*, 32159-32169, doi:10.1021/acsami.6b10175.

104. Zhang, Y.; Zhai, M.; Chen, Z.; Han, X.; Yu, F.; Li, Z.; Xie, X.; Han, C.; Yu, L.; Yang, Y., et al. Dual-modified liposome codelivery of doxorubicin and vincristine improve targeting and therapeutic efficacy of glioma. *Drug Delivery* **2017**, *24*, 1045-1055, doi:10.1080/10717544.2017.1344334.

105. Arranz-Gibert, P.; Prades, R.; Guixer, B.; Guerrero, S.; Araya, E.; Ciudad, S.; Kogan, M.J.; Giralt, E.; Teixidó, M. HAI Peptide and Backbone Analogs—Validation and Enhancement of Biostability and Bioactivity of BBB Shuttles. *Scientific Reports* **2018**, *8*.

106. Lee, J.H.; Engler, J.A.; Collawn, J.F.; Moore, B.A. Receptor mediated uptake of peptides that bind the human transferrin receptor. *European Journal of Biochemistry* **2001**, *268*, 2004-2012, doi:10.1046/j.1432-1327.2001.02073.x.

107. Zong, T.; Mei, L.; Gao, H.; Cai, W.; Zhu, P.; Shi, K.; Chen, J.; Wang, Y.; Gao, F.; He, Q. Synergistic Dual-Ligand Doxorubicin Liposomes Improve Targeting and Therapeutic Efficacy of Brain Glioma in Animals. *Molecular Pharmaceutics* **2014**, *11*, 2346-2357, doi:10.1021/mp500057n.

108. Wang, M.; Zhi, D.; Wang, H.; Ru, Y.; Ren, H.; Wang, N.; Liu, Y.; Li, Y.; Li, H. TAT-HSA-α-MSH fusion protein with extended half-life inhibits tumor necrosis factor-α in brain inflammation of mice. *Applied Microbiology and Biotechnology* **2016**, *100*, 5353-5361, doi:10.1007/s00253-015-7251-4.

109. Schwarze, S.R.; Ho, A.; Vocero-Akbani, A.; Dowdy, S.F. In Vivo Protein Transduction: Delivery of a Biologically Active Protein into the Mouse. *Science* **1999**, *285*, 1569, doi:10.1126/science.285.5433.1569.

110. Maderna, E.; Colombo, L.; Cagnotto, A.; Di Fede, G.; Indaco, A.; Tagliavini, F.; Salmona, M.; Giaccone, G. In Situ Tissue Labeling of Cerebral Amyloid Using HIV-Related Tat Peptide. *Molecular Neurobiology* **2018**, *55*, 6834-6840, doi:10.1007/s12035-018-0870-x.

111. Liu, L.; Venkatraman, S.S.; Yang, Y.-Y.; Guo, K.; Lu, J.; He, B.; Moochhala, S.; Kan, L. Polymeric micelles anchored with TAT for delivery of antibiotics across the blood–brain barrier. *Peptide Science* **2008**, *90*, 617-623, doi:10.1002/bip.20998.

112. Dos Santos Rodrigues, B.; Lakkadwala, S.; Kanekiyo, T.; Singh, J. Development and screening of brain-targeted lipid-based nanoparticles with enhanced cell penetration and gene delivery properties. *International journal of nanomedicine* **2019**, *14*, 6497-6517, doi:10.2147/IJN.S215941.

113. Moochhala and D. Luo, L.L.a.Y.-Y.Y.a.S.S.V.a.K.G.a.J.L.a.E.-A.L.a.K.C.N.a.S. Biologically active core/shell nanoparticles self-assembled from cholesterol-terminated PEG-TAT for drug delivery across the blood-brain barrier. 2008.

114. Kilic, E.; Dietz, G.P.H.; Hermann, D.M.; Bähr, M. Intravenous TAT–Bcl-Xl is protective after middle cerebral artery occlusion in mice. *Annals of Neurology* **2002**, *52*, 617-622, doi:10.1002/ana.10356.

115. Aarts, M.; Liu, Y.; Liu, L.; Besshoh, S.; Arundine, M.; Gurd, J.W.; Wang, Y.-T.; Salter, M.W.; Tymianski, M. Treatment of Ischemic Brain Damage by Perturbing NMDA Receptor- PSD-95 Protein Interactions. *Science* **2002**, *298*, 846, doi:10.1126/science.1072873.

116. Elliger, S.S.; Elliger, C.A.; Lang, C.; Watson, G.L. Enhanced Secretion and Uptake of β-Glucuronidase Improves Adeno-associated Viral-Mediated Gene Therapy of Mucopolysaccharidosis Type VII Mice. *Molecular Therapy* **2002**, *5*, 617-626, doi:<https://doi.org/10.1006/mthe.2002.0594>.

117. Kilic, E.; Kilic, Ü.; Hermann, D.M. TAT-GDNF in Neurodegeneration and Ischemic Stroke. *CNS Drug Reviews* **2005**, *11*, 369-378, doi:10.1111/j.1527-3458.2005.tb00054.x.

118. Qin, Y.; Chen, H.; Zhang, Q.; Wang, X.; Yuan, W.; Kuai, R.; Tang, J.; Zhang, L.; Zhang, Z.; Zhang, Q., et al. Liposome formulated with TAT-modified cholesterol for improving brain delivery and therapeutic efficacy on brain glioma in animals. *International Journal of Pharmaceutics* **2011**, *420*, 304-312, doi:<https://doi.org/10.1016/j.ijpharm.2011.09.008>.

119. Wang, H.; Xu, K.; Liu, L.; Tan, J.P.K.; Chen, Y.; Li, Y.; Fan, W.; Wei, Z.; Sheng, J.; Yang, Y.-Y., et al. The efficacy of self-assembled cationic antimicrobial peptide nanoparticles against Cryptococcus neoformans for the treatment of meningitis. *Biomaterials* **2010**, *31*, 2874-2881, doi:<https://doi.org/10.1016/j.biomaterials.2009.12.042>.

120. Tian, X.-H.; Wang, Z.-G.; Meng, H.; Wang, Y.-H.; Feng, W.; Wei, F.; Huang, Z.-C.; Lin, X.-N.; Ren, L. Tat peptide-decorated gelatin-siloxane nanoparticles for delivery of CGRP transgene in treatment of cerebral vasospasm. *International journal of nanomedicine* **2013**, *8*, 865-876, doi:10.2147/IJN.S39951.

121. Rao, K.S.; Reddy, M.K.; Horning, J.L.; Labhasetwar, V. TAT-conjugated nanoparticles for the CNS delivery of anti-HIV drugs. *Biomaterials* **2008**, *29*, 4429-4438, doi:10.1016/j.biomaterials.2008.08.004.

122. Zhao, X.; Shang, T.; Zhang, X.; Ye, T.; Wang, D.; Rei, L. Passage of Magnetic Tat-Conjugated Fe3O4@SiO2 Nanoparticles Across In Vitro Blood-Brain Barrier. *Nanoscale Research Letters* **2016**, *11*, 451, doi:10.1186/s11671-016-1676-2.

123. Gregori, M.; Taylor, M.; Salvati, E.; Re, F.; Mancini, S.; Balducci, C.; Forloni, G.; Zambelli, V.; Sesana, S.; Michael, M., et al. Retro-inverso peptide inhibitor nanoparticles as potent inhibitors of aggregation of the Alzheimer's Aβ peptide. *Nanomedicine: Nanotechnology, Biology and Medicine* **2017**, *13*, 723-732, doi:<https://doi.org/10.1016/j.nano.2016.10.006>.

124. Li, J.; Feng, L.; Fan, L.; Zha, Y.; Guo, L.; Zhang, Q.; Chen, J.; Pang, Z.; Wang, Y.; Jiang, X., et al. Targeting the brain with PEG-PLGA nanoparticles modified with phage-displayed peptides. *Biomaterials* **2011**, *32*, 4943-4950, doi:10.1016/j.biomaterials.2011.03.031.

125. Li, J.; Zhang, C.; Li, J.; Fan, L.; Jiang, X.; Chen, J.; Pang, Z.; Zhang, Q. Brain Delivery of NAP with PEG-PLGA Nanoparticles Modified with Phage Display Peptides. *Pharmaceutical Research* **2013**, *30*, 1813-1823, doi:10.1007/s11095-013-1025-4.

126. Gao, H.; Qian, J.; Cao, S.; Yang, Z.; Pang, Z.; Pan, S.; Fan, L.; Xi, Z.; Jiang, X.; Zhang, Q. Precise glioma targeting of and penetration by aptamer and peptide dual-functioned nanoparticles. *Biomaterials* **2012**, *33*, 5115-5123, doi:10.1016/j.biomaterials.2012.03.058.

127. Prest, T.A.; Yeager, E.; LoPresti, S.T.; Zygelyte, E.; Martin, M.J.; Dong, L.; Gibson, A.; Olutoye, O.O.; Brown, B.N.; Cheetham, J. Nerve-specific, xenogeneic extracellular matrix hydrogel promotes recovery following peripheral nerve injury. *Journal of biomedical materials research. Part A* **2018**, *106*, 450-459, doi:10.1002/jbm.a.36235.

128. Zhang, C.; Zheng, X.; Wan, X.; Shao, X.; Liu, Q.; Zhang, Z.; Zhang, Q. The potential use of H102 peptide-loaded dual-functional nanoparticles in the treatment of Alzheimer's disease. *Journal of Controlled Release* **2014**, *192*, 317-324, doi:<https://doi.org/10.1016/j.jconrel.2014.07.050>.

129. Ma, H. A dual functional fluorescent probe for glioma imaging mediated by Blood-brain barrier penetration and glioma cell targeting. *Biochemical and biophysical research communications* **2014**, *v. 449*, pp. 44-48-2014 v.2449, doi:10.1016/j.bbrc.2014.04.148.

130. Nag, O.K.; Delehanty, J.B. Active Cellular and Subcellular Targeting of Nanoparticles for Drug Delivery. *Pharmaceutics* **2019**, *11*, 543, doi:10.3390/pharmaceutics11100543.

131. Prades, R. Delivery of gold nanoparticles to the brain by conjugation with a peptide that recognizes the transferrin receptor. *Biomaterials* **2012**, *v. 33*, pp. 7194-7205-2012 v.7133 no.7129, doi:10.1016/j.biomaterials.2012.06.063.

132. Prades, R.; Oller-Salvia, B.; Schwarzmaier, S.M.; Selva, J.; Moros, M.; Balbi, M.; Grazú, V.; de La Fuente, J.M.; Egea, G.; Plesnila, N., et al. Applying the Retro-Enantio Approach To Obtain a Peptide Capable of Overcoming the Blood–Brain Barrier. *Angewandte Chemie International Edition* **2015**, *54*, 3967-3972, doi:10.1002/anie.201411408.

133. Zhang, X.; He, T.; Chai, Z.; Samulski, R.J.; Li, C. Blood-brain barrier shuttle peptides enhance AAV transduction in the brain after systemic administration. *Biomaterials* **2018**, *176*, 71-83, doi:10.1016/j.biomaterials.2018.05.041.

134. Wängler, C.; Nada, D.; Höfner, G.; Maschauer, S.; Wängler, B.; Schneider, S.; Schirrmacher, E.; Wanner, K.T.; Schirrmacher, R.; Prante, O. In Vitro and Initial In Vivo Evaluation of 68Ga-Labeled Transferrin Receptor (TfR) Binding Peptides as Potential Carriers for Enhanced Drug Transport into TfR Expressing Cells. *Molecular Imaging and Biology* **2011**, *13*, 332-341, doi:10.1007/s11307-010-0329-6.

135. Youn, P.; Chen, Y.; Furgeson, D.Y. A Myristoylated Cell-Penetrating Peptide Bearing a Transferrin Receptor-Targeting Sequence for Neuro-Targeted siRNA Delivery. *Molecular Pharmaceutics* **2014**, *11*, 486-495, doi:10.1021/mp400446v.

136. Díaz-Perlas, C.; Oller-Salvia, B.; Sánchez-Navarro, M.; Teixidó, M.; Giralt, E. Branched BBB-shuttle peptides: chemoselective modification of proteins to enhance blood–brain barrier transport. *Chemical Science* **2018**, *9*, 8409-8415, doi:10.1039/C8SC02415D.

137. Guo, Q.; Xu, S.; Yang, P.; Wang, P.; Lu, S.; Sheng, D.; Qian, K.; Cao, J.; Lu, W.; Zhang, Q. A dual-ligand fusion peptide improves the brain-neuron targeting of nanocarriers in Alzheimer's disease mice. *Journal of Controlled Release* **2020**, *320*, 347-362, doi:<https://doi.org/10.1016/j.jconrel.2020.01.039>.

138. Wang, K.; Yang, Y.; Xue, W.; Liu, Z. Cell Penetrating Peptide-Based Redox-Sensitive Vaccine Delivery System for Subcutaneous Vaccination. *Molecular Pharmaceutics* **2018**, *15*, 975-984, doi:10.1021/acs.molpharmaceut.7b00905.

139. Kokotidou, C.; Jonnalagadda, V.R.S.; Orr, A.A.; Vrentzos, G.; Kretsovali, A.; Tamamis, P.; Mitraki, A. Designer Amyloid Cell-Penetrating Peptides for Potential Use as Gene Transfer Vehicles. *Biomolecules* **2019**, *10*, doi:10.3390/biom10010007.

140. Liu, Y.; He, X.; Kuang, Y.; An, S.; Wang, C.; Guo, Y.; Ma, H.; Lou, J.; Jiang, C. A Bacteria Deriving Peptide Modified Dendrigraft Poly-l-lysines (DGL) Self-Assembling Nanoplatform for Targeted Gene Delivery. *Molecular Pharmaceutics* **2014**, *11*, 3330-3341, doi:10.1021/mp500084s.

141. Vernen, F.; Craik, D.J.; Lawrence, N.; Troeira Henriques, S. Cyclic Analogues of Horseshoe Crab Peptide Tachyplesin I with Anticancer and Cell Penetrating Properties. *ACS Chemical Biology* **2019**, *14*, 2895-2908, doi:10.1021/acschembio.9b00782.

142. Young Kim, H.; Young Yum, S.; Jang, G.; Ahn, D.-R. Discovery of a non-cationic cell penetrating peptide derived from membrane-interacting human proteins and its potential as a protein delivery carrier. *Scientific Reports* **2015**, *5*, 11719, doi:10.1038/srep11719.

143. van Duijnhoven, S.M.J.; Robillard, M.S.; Nicolay, K.; Grüll, H. Tumor Targeting of MMP-2/9 Activatable Cell-Penetrating Imaging Probes Is Caused by Tumor-Independent Activation. *Journal of Nuclear Medicine* **2011**.

144. Xiang, B.; Jia, X.-L.; Qi, J.-L.; Yang, L.-P.; Sun, W.-H.; Yan, X.; Yang, S.-K.; Cao, D.-Y.; Du, Q.; Qi, X.-R. Enhancing siRNA-based cancer therapy using a new pH-responsive activatable cell-penetrating peptide-modified liposomal system. In *International journal of nanomedicine*, 2017; Vol. 12, pp 2385-2405.

145. Olson, E.S.; Jiang, T.; Aguilera, T.A.; Nguyen, Q.T.; Ellies, L.G.; Scadeng, M.; Tsien, R.Y. Activatable cell penetrating peptides linked to nanoparticles as dual probes for in vivo fluorescence and MR imaging of proteases. *Proceedings of the National Academy of Sciences* **2010**, 10.1073/pnas.0910283107, 200910283, doi:10.1073/pnas.0910283107.

146. Unkart, J.T.; Chen, S.L.; Wapnir, I.L.; González, J.E.; Harootunian, A.; Wallace, A.M. Intraoperative Tumor Detection Using a Ratiometric Activatable Fluorescent Peptide: A First-in-Human Phase 1 Study. *Annals of Surgical Oncology* **2017**, *24*, 3167-3173, doi:10.1245/s10434-017-5991-3.

147. Aguilera, T.A.; Olson, E.S.; Timmers, M.M.; Jiang, T.; Tsien, R.Y. Systemic in vivo distribution of activatable cell penetrating peptides is superior to that of cell penetrating peptides. *Integrative biology : quantitative biosciences from nano to macro* **2009**, *1*, 371-381, doi:10.1039/b904878b.

148. van Duijnhoven, S.M.J.; Robillard, M.S.; Nicolay, K.; Grüll, H. In vivo biodistribution of radiolabeled MMP-2/9 activatable cell-penetrating peptide probes in tumor-bearing mice. *Contrast Media & Molecular Imaging* **2015**, *10*, 59-66, doi:10.1002/cmmi.1605.

149. Wang, Y.F.; Xu, X.; Fan, X.; Zhang, C.; Wei, Q.; Wang, X.; Guo, W.; Xing, W.; Yu, J.; Yan, J.-L., et al. A cell-penetrating peptide suppresses inflammation by inhibiting NF-κB signaling. *Molecular therapy : the journal of the American Society of Gene Therapy* **2011**, *19*, 1849-1857, doi:10.1038/mt.2011.82.

150. Suckfuell, M.; Lisowska, G.; Domka, W.; Kabacinska, A.; Morawski, K.; Bodlaj, R.; Klimak, P.; Kostrica, R.; Meyer, T. Efficacy and Safety of AM-111 in the Treatment of Acute Sensorineural Hearing Loss: A Double-Blind, Randomized, Placebo-Controlled Phase II Study. *Otology & Neurotology* **2014**, *35*.

151. Qian, Z.; Rhodes, C.A.; McCroskey, L.C.; Wen, J.; Appiah-Kubi, G.; Wang, D.J.; Guttridge, D.C.; Pei, D. Enhancing the Cell Permeability and Metabolic Stability of Peptidyl Drugs by Reversible Bicyclization. *Angewandte Chemie International Edition* **2017**, *56*, 1525-1529, doi:10.1002/anie.201610888.

152. Prochiantz, A. Getting hydrophilic compounds into cells: lessons from homeopeptides. *Current Opinion in Neurobiology* **1996**, *6*, 629-634, doi:<https://doi.org/10.1016/S0959-4388(96)80095-X>.

153. Smith, B.A.; Daniels, D.S.; Coplin, A.E.; Jordan, G.E.; McGregor, L.M.; Schepartz, A. Minimally Cationic Cell-Permeable Miniature Proteins via α-Helical Arginine Display. *Journal of the American Chemical Society* **2008**, *130*, 2948-2949, doi:10.1021/ja800074v.

154. Johansson, H.J.; El-Andaloussi, S.; Holm, T.; Mäe, M.; Jänes, J.; Maimets, T.; Langel, Ü. Characterization of a Novel Cytotoxic Cell‐penetrating Peptide Derived From p14ARF Protein. *Molecular Therapy* **2008**, *16*, 115-123, doi:10.1038/sj.mt.6300346.

155. Raheem, N.; Kumar, P.; Lee, E.; Cheng, J.T.J.; Hancock, R.E.W.; Straus, S.K. Insights into the mechanism of action of two analogues of aurein 2.2. *Biochimica et Biophysica Acta (BBA) - Biomembranes* **2020**, *1862*, 183262, doi:<https://doi.org/10.1016/j.bbamem.2020.183262>.

156. Langedijk, J.P.M.; Olijhoek, T.; Meloen, R.H. Application, efficiency and cargo-dependence of transport peptides. *International Congress Series* **2005**, *1277*, 95-107, doi:<https://doi.org/10.1016/j.ics.2005.02.016>.

157. Lopes, L.B.; Furnish, E.J.; Komalavilas, P.; Flynn, C.R.; Ashby, P.; Hansen, A.; Ly, D.P.; Yang, G.P.; Longaker, M.T.; Panitch, A., et al. Cell Permeant Peptide Analogues of the Small Heat Shock Protein, HSP20, Reduce TGF-β1-Induced CTGF Expression in Keloid Fibroblasts. *Journal of Investigative Dermatology* **2009**, *129*, 590-598, doi:<https://doi.org/10.1038/jid.2008.264>.

158. Yang, H.; Liu, S.; Cai, H.; Wan, L.; Li, S.; Li, Y.; Cheng, J.; Lu, X. Chondroitin sulfate as a molecular portal that preferentially mediates the apoptotic killing of tumor cells by penetratin-directed mitochondria-disrupting peptides. *The Journal of biological chemistry* **2010**, *285*, 25666-25676, doi:10.1074/jbc.M109.089417.

159. Sadler, K.; Eom, K.D.; Yang, J.-L.; Dimitrova, Y.; Tam, J.P. Translocating Proline-Rich Peptides from the Antimicrobial Peptide Bactenecin 7. *Biochemistry* **2002**, *41*, 14150-14157, doi:10.1021/bi026661l.

160. Bird, G.H.; Mazzola, E.; Opoku-Nsiah, K.; Lammert, M.A.; Godes, M.; Neuberg, D.S.; Walensky, L.D. Biophysical determinants for cellular uptake of hydrocarbon-stapled peptide helices. *Nature chemical biology* **2016**, *12*, 845-852, doi:10.1038/nchembio.2153.

161. Nakase, I.; Hirose, H.; Tanaka, G.; Tadokoro, A.; Kobayashi, S.; Takeuchi, T.; Futaki, S. Cell-surface Accumulation of Flock House Virus-derived Peptide Leads to Efficient Internalization via Macropinocytosis. *Molecular Therapy* **2009**, *17*, 1868-1876, doi:<https://doi.org/10.1038/mt.2009.192>.

162. Futaki, S.; Nakase, I. Cell-Surface Interactions on Arginine-Rich Cell-Penetrating Peptides Allow for Multiplex Modes of Internalization. *Accounts of Chemical Research* **2017**, *50*, 2449-2456, doi:10.1021/acs.accounts.7b00221.

163. Reynolds, F.; Weissleder, R.; Josephson, L. Protamine as an efficient membrane-translocating peptide. *Bioconjugate chemistry* **2005**, *16*, 1240-1245, doi:10.1021/bc0501451.

164. Soler, M.; González-Bártulos, M.; Soriano-Castell, D.; Ribas, X.; Costas, M.; Tebar, F.; Massaguer, A.; Feliu, L.; Planas, M. Identification of BP16 as a non-toxic cell-penetrating peptide with highly efficient drug delivery properties. *Organic & Biomolecular Chemistry* **2014**, *12*, 1652-1663, doi:10.1039/C3OB42422G.

165. Thagun, C.; Chuah, J.-A.; Numata, K. Targeted Gene Delivery into Various Plastids Mediated by Clustered Cell-Penetrating and Chloroplast-Targeting Peptides. *Advanced Science* **2019**, *6*, 1902064, doi:10.1002/advs.201902064.

166. Guo, B.; Itami, J.; Oikawa, K.; Motoda, Y.; Kigawa, T.; Numata, K. Native protein delivery into rice callus using ionic complexes of protein and cell-penetrating peptides. In *PloS one*, 2019; Vol. 14, p e0214033.

167. Magzoub, M.; Sandgren, S.; Lundberg, P.; Oglecka, K.; Lilja, J.; Wittrup, A.; Göran Eriksson, L.E.; Langel, U.; Belting, M.; Gräslund, A. N-terminal peptides from unprocessed prion proteins enter cells by macropinocytosis. *Biochemical and biophysical research communications* **2006**, *348*, 379-385, doi:10.1016/j.bbrc.2006.07.065.

168. Lundberg, P.; El-Andaloussi, S.; Sütlü, T.; Johansson, H.; Langel, Ü. Delivery of short interfering RNA using endosomolytic cell-penetrating peptides. *The FASEB Journal* **2007**, *21*, 2664-2671, doi:10.1096/fj.06-6502com.

169. Lee, Y.; Hwang, Y.; Lee, J.; Sohn, J.-H.; Sung, B.; Kim, S. VEGF siRNA Delivery by a Cancer-Specific Cell-Penetrating Peptide. *J. Microbiol. Biotechnol.* **2018**, *28*, 367–374, doi:10.4014/jmb.1711.11025.

170. Lim, K.J.; Sung, B.H.; Shin, J.R.; Lee, Y.W.; Kim, D.J.; Yang, K.S.; Kim, S.C. A Cancer Specific Cell-Penetrating Peptide, BR2, for the Efficient Delivery of an scFv into Cancer Cells. *PLOS ONE* **2013**, *8*, e66084, doi:10.1371/journal.pone.0066084.

171. Kobayashi, S.; Takeshima, K.; Park, C.B.; Kim, S.C.; Matsuzaki, K. Interactions of the Novel Antimicrobial Peptide Buforin 2 with Lipid Bilayers:  Proline as a Translocation Promoting Factor. *Biochemistry* **2000**, *39*, 8648-8654, doi:10.1021/bi0004549.

172. Rhee, M.; Davis, P. Mechanism of Uptake of C105Y, a Novel Cell-penetrating Peptide. *Journal of Biological Chemistry* **2006**, *281*, 1233-1240.

173. Howl, J.; Jones, S. Cell penetrating peptide-mediated transport enables the regulated secretion of accumulated cargoes from mast cells. *Journal of controlled release : official journal of the Controlled Release Society* **2015**, *202*, 108-117, doi:10.1016/j.jconrel.2015.02.005.

174. Jones, S.; Lukanowska, M.; Suhorutsenko, J.; Oxenham, S.; Barratt, C.; Publicover, S.; Copolovici, D.M.; Langel, Ü.; Howl, J. Intracellular translocation and differential accumulation of cell-penetrating peptides in bovine spermatozoa: evaluation of efficient delivery vectors that do not compromise human sperm motility. *Human Reproduction* **2013**, *28*, 1874-1889, doi:10.1093/humrep/det064.

175. Qian, Z.; Liu, T.; Liu, Y.-Y.; Briesewitz, R.; Barrios, A.M.; Jhiang, S.M.; Pei, D. Efficient Delivery of Cyclic Peptides into Mammalian Cells with Short Sequence Motifs. *ACS Chemical Biology* **2013**, *8*, 423-431, doi:10.1021/cb3005275.

176. Qian, Z.; Xu, X.; Amacher, J.F.; Madden, D.R.; Cormet-Boyaka, E.; Pei, D. Intracellular Delivery of Peptidyl Ligands by Reversible Cyclization: Discovery of a PDZ Domain Inhibitor that Rescues CFTR Activity. *Angewandte Chemie (International ed. in English)* **2015**, *54*, 5874-5878, doi:10.1002/anie.201411594.

177. Qian, Z.; Martyna, A.; Hard, R.L.; Wang, J.; Appiah-Kubi, G.; Coss, C.; Phelps, M.A.; Rossman, J.S.; Pei, D. Discovery and Mechanism of Highly Efficient Cyclic Cell-Penetrating Peptides. *Biochemistry* **2016**, *55*, 2601-2612, doi:10.1021/acs.biochem.6b00226.

178. Trinh, T.B.; Upadhyaya, P.; Qian, Z.; Pei, D. Discovery of a Direct Ras Inhibitor by Screening a Combinatorial Library of Cell-Permeable Bicyclic Peptides. *ACS Combinatorial Science* **2016**, *18*, 75-85, doi:10.1021/acscombsci.5b00164.

179. Song, J.; Qian, Z.; Sahni, A.; Chen, K.; Pei, D. Cyclic Cell-Penetrating Peptides with Single Hydrophobic Groups. *ChemBioChem* **2019**, *20*, 2085-2088, doi:10.1002/cbic.201900370.

180. Yang, W.; Xia, Y.; Fang, Y.; Meng, F.; Zhang, J.; Cheng, R.; Deng, C.; Zhong, Z. Selective Cell Penetrating Peptide-Functionalized Polymersomes Mediate Efficient and Targeted Delivery of Methotrexate Disodium to Human Lung Cancer In Vivo. *Advanced Healthcare Materials* **2018**, *7*, 1701135, doi:10.1002/adhm.201701135.

181. Qiu, M.; Ouyang, J.; Wei, Y.; Zhang, J.; Lan, Q.; Deng, C.; Zhong, Z. Selective Cell Penetrating Peptide-Functionalized Envelope-Type Chimeric Lipopepsomes Boost Systemic RNAi Therapy for Lung Tumors. *Advanced Healthcare Materials* **2019**, *8*, 1900500, doi:10.1002/adhm.201900500.

182. Tian, R.; Wang, H.; Niu, R.; Ding, D. Drug delivery with nanospherical supramolecular cell penetrating peptide-taxol conjugates containing a high drug loading. *Journal of colloid and interface science* **2015**, *453*, 15-20, doi:10.1016/j.jcis.2015.04.028.

183. Zhang, W.; Taheri-Ledari, R.; Hajizadeh, Z.; Zolfaghari, E.; Ahghari, M.R.; Maleki, A.; Hamblin, M.R.; Tian, Y. Enhanced activity of vancomycin by encapsulation in hybrid magnetic nanoparticles conjugated to a cell-penetrating peptide. *Nanoscale* **2020**, *12*, 3855-3870, doi:10.1039/C9NR09687F.

184. Asai, T.; Tsuzuku, T.; Takahashi, S.; Okamoto, A.; Dewa, T.; Nango, M.; Hyodo, K.; Ishihara, H.; Kikuchi, H.; Oku, N. Cell-penetrating peptide-conjugated lipid nanoparticles for siRNA delivery. *Biochemical and Biophysical Research Communications* **2014**, *444*, 599-604, doi:<https://doi.org/10.1016/j.bbrc.2014.01.107>.

185. Nakayama, F.; Yasuda, T.; Umeda, S.; Asada, M.; Imamura, T.; Meineke, V.; Akashi, M. Fibroblast growth factor-12 translocation into intestinal epithelial cells is dependent on a novel cell-penetrating peptide domain: Involvement of internalization in the in Vivo role of exogenous FGF12. *Journal of Biological Chemistry* **2011**.

186. Subia, B.; Reinisalo, M.; Dey, N.; Tavakoli, S.; Subrizi, A.; Ganguli, M.; Ruponen, M. Nucleic acid delivery to differentiated retinal pigment epithelial cells using cell-penetrating peptide as a carrier. *European Journal of Pharmaceutics and Biopharmaceutics* **2019**, *140*, 91-99, doi:<https://doi.org/10.1016/j.ejpb.2019.05.003>.

187. Philippe, G.J.B.; Gaspar, D.; Sheng, C.; Huang, Y.-H.; Benfield, A.H.; Condon, N.D.; Weidmann, J.; Lawrence, N.; Löwer, A.; Castanho, M.A.R.B., et al. Cell Membrane Composition Drives Selectivity and Toxicity of Designed Cyclic Helix–Loop–Helix Peptides with Cell Penetrating and Tumor Suppressor Properties. *ACS Chemical Biology* **2019**, *14*, 2071-2087, doi:10.1021/acschembio.9b00593.

188. Vallespí, M.G.; Pimentel, G.; Cabrales-Rico, A.; Garza, J.; Oliva, B.; Mendoza, O.; Gomez, Y.; Basaco, T.; Sánchez, I.; Calderón, C., et al. Antitumor efficacy, pharmacokinetic and biodistribution studies of the anticancer peptide CIGB-552 in mouse models. *Journal of peptide science : an official publication of the European Peptide Society* **2014**, *20*, 850-859, doi:10.1002/psc.2676.

189. Read, S.P.; Cashman, S.M.; Kumar-Singh, R. A poly(ethylene) glycolylated peptide for ocular delivery compacts DNA into nanoparticles for gene delivery to post-mitotic tissues in vivo. *The Journal of Gene Medicine* **2010**, *12*, 86-96, doi:10.1002/jgm.1415.

190. Soudah, T.; Mogilevsky, M.; Karni, R.; Yavin, E. CLIP6-PNA-Peptide Conjugates: Non-Endosomal Delivery of Splice Switching Oligonucleotides. *Bioconjugate Chemistry* **2017**, *28*, 3036-3042, doi:10.1021/acs.bioconjchem.7b00638.

191. Wu, H.; Zhuang, Q.; Xu, J.; Xu, L.; Zhao, Y.; Wang, C.; Yang, Z.; Shen, F.; Liu, Z.; Peng, R. Cell-Penetrating Peptide Enhanced Antigen Presentation for Cancer Immunotherapy. *Bioconjugate Chemistry* **2019**, *30*, 2115-2126, doi:10.1021/acs.bioconjchem.9b00245.

192. Liu, F.; Lou, J.; Hristov, D. X-Ray responsive nanoparticles with triggered release of nitrite, a precursor of reactive nitrogen species, for enhanced cancer radiosensitization. *Nanoscale* **2017**, *9*, 14627-14634, doi:10.1039/C7NR04684G.

193. Mi, Z.; Lu, X.; Mai, J.C.; Ng, B.G.; Wang, G.; Lechman, E.R.; Watkins, S.C.; Rabinowich, H.; Robbins, P.D. Identification of a synovial fibroblast-specific protein transduction domain for delivery of apoptotic agents to hyperplastic synovium. *Molecular Therapy* **2003**, *8*, 295-305, doi:<https://doi.org/10.1016/S1525-0016(03)00181-3>.

194. Rodrigues, M.; Santos, A.; de la Torre, B.G.; Rádis-Baptista, G.; Andreu, D.; Santos, N.C. Molecular characterization of the interaction of crotamine-derived nucleolar targeting peptides with lipid membranes. *Biochimica et biophysica acta* **2012**, *1818*, 2707-2717, doi:10.1016/j.bbamem.2012.06.014.

195. Kerkis, A.; Kerkis, I.; RÁDis-Baptista, G.; Oliveira, E.B.; Vianna-Morgante, A.M.; Pereira, L.V.; Yamane, T. Crotamine is a novel cell-penetrating protein from the venom of rattlesnake Crotalus durissus terrificus. *The FASEB Journal* **2004**, *18*, 1407-1409, doi:10.1096/fj.03-1459fje.

196. Chen, H.-Z.; Wu, C.P.; Chao, Y.-C.; Liu, C.Y.-Y. Membrane penetrating peptides greatly enhance baculovirus transduction efficiency into mammalian cells. *Biochemical and Biophysical Research Communications* **2011**, *405*, 297-302, doi:<https://doi.org/10.1016/j.bbrc.2011.01.032>.

197. Zahid, M.; Feldman, S.K.; Garcia-Borrero, G.; Feinstein, N.T.; Pogodzinski, N.; Xu, X.; Yurko, R.; Czachowski, M.; Wu, L.Y.; Mason, S.N., et al. Cardiac Targeting Peptide, a Novel Cardiac Vector: Studies in Bio-Distribution, Imaging Application, and Mechanism of Transduction. *Biomolecules* **2018**, *8*, doi:10.3390/biom8040147.

198. Zahid, M.; Phillips, B.E.; Albers, S.M.; Giannoukakis, N.; Watkins, S.C.; Robbins, P.D. Identification of a Cardiac Specific Protein Transduction Domain by In Vivo Biopanning Using a M13 Phage Peptide Display Library in Mice. *PLOS ONE* **2010**, *5*, e12252, doi:10.1371/journal.pone.0012252.

199. Hu, G.; Zheng, W.; Li, A.; Mu, Y.; Shi, M.; Li, T.; Zou, H.; Shao, H.; Qin, A.; Ye, J. A novel CAV derived cell-penetrating peptide efficiently delivers exogenous molecules through caveolae-mediated endocytosis. *Veterinary Research* **2018**, *49*, 16, doi:10.1186/s13567-018-0513-2.

200. Wu, Y.; Sun, J.; Li, A.; Chen, D. The promoted delivery of RRM2 siRNA to vascular smooth muscle cells through liposome-polycation-DNA complex conjugated with cell penetrating peptides. *Biomedicine & Pharmacotherapy* **2018**, *103*, 982-988, doi:<https://doi.org/10.1016/j.biopha.2018.03.068>.

201. Jha, D.; Mishra, R.; Gottschalk, S.; Wiesmüller, K.-H.; Ugurbil, K.; Maier, M.E.; Engelmann, J. CyLoP-1: A Novel Cysteine-Rich Cell-Penetrating Peptide for Cytosolic Delivery of Cargoes. *Bioconjugate Chemistry* **2011**, *22*, 319-328, doi:10.1021/bc100045s.

202. Carnevale, K.J.F.; Muroski, M.E.; Vakil, P.N.; Foley, M.E.; Laufersky, G.; Kenworthy, R.; Zorio, D.A.R.; Morgan, T.J.; Levenson, C.W.; Strouse, G.F. Selective Uptake Into Drug Resistant Mammalian Cancer by Cell Penetrating Peptide-Mediated Delivery. *Bioconjugate Chemistry* **2018**, *29*, 3273-3284, doi:10.1021/acs.bioconjchem.8b00429.

203. Kurzawa, L.; Pellerano, M.; Morris, M.C. PEP and CADY-mediated delivery of fluorescent peptides and proteins into living cells. *Biochimica et Biophysica Acta (BBA) - Biomembranes* **2010**, *1798*, 2274-2285, doi:<https://doi.org/10.1016/j.bbamem.2010.02.027>.

204. Eiríksdóttir, E.; Konate, K.; Langel, Ü.; Divita, G.; Deshayes, S. Secondary structure of cell-penetrating peptides controls membrane interaction and insertion. *Biochimica et Biophysica Acta (BBA) - Biomembranes* **2010**, *1798*, 1119-1128, doi:<https://doi.org/10.1016/j.bbamem.2010.03.005>.

205. Urgard, E.; Lorents, A.; Klaas, M.; Padari, K.; Viil, J.; Runnel, T.; Langel, K.; Kingo, K.; Tkaczyk, E.; Langel, Ü., et al. Pre-administration of PepFect6-microRNA-146a nanocomplexes inhibits inflammatory responses in keratinocytes and in a mouse model of irritant contact dermatitis. *Journal of Controlled Release* **2016**, *235*, 195-204, doi:<https://doi.org/10.1016/j.jconrel.2016.06.006>.

206. Grasso, G.; Mercuri, S.; Danani, A.; Deriu, M.A. Biofunctionalization of Silica Nanoparticles with Cell-Penetrating Peptides: Adsorption Mechanism and Binding Energy Estimation. *The Journal of Physical Chemistry B* **2019**, *123*, 10622-10630, doi:10.1021/acs.jpcb.9b08106.

207. Konate, K.; Crombez, L.; Deshayes, S.; Decaffmeyer, M.; Thomas, A.; Brasseur, R.; Aldrian, G.; Heitz, F.; Divita, G. Insight into the Cellular Uptake Mechanism of a Secondary Amphipathic Cell-Penetrating Peptide for siRNA Delivery. *Biochemistry* **2010**, *49*, 3393-3402, doi:10.1021/bi901791x.

208. Crombez, L.; Aldrian-Herrada, G.; Konate, K.; Nguyen, Q.N.; McMaster, G.K.; Brasseur, R.; Heitz, F.; Divita, G. A new potent secondary amphipathic cell-penetrating peptide for siRNA delivery into mammalian cells. *Molecular therapy : the journal of the American Society of Gene Therapy* **2009**, *17*, 95-103, doi:10.1038/mt.2008.215.

209. Vaissière, A.; Aldrian, G.; Konate, K.; Lindberg, M.F.; Jourdan, C.; Telmar, A.; Seisel, Q.; Fernandez, F.; Viguier, V.; Genevois, C., et al. A retro-inverso cell-penetrating peptide for siRNA delivery. *Journal of Nanobiotechnology* **2017**, *15*, 34, doi:10.1186/s12951-017-0269-2.

210. Shahbazi, S.; Bolhassani, A. Comparison of six cell penetrating peptides with different properties for in vitro and in vivo delivery of HPV16 E7 antigen in therapeutic vaccines. *International immunopharmacology* **2018**, *62*, 170-180, doi:10.1016/j.intimp.2018.07.006.

211. Chen, C.; Liu, K.; Xu, Y.; Zhang, P.; Suo, Y.; Lu, Y.; Zhang, W.; Su, L.; Gu, Q.; Wang, H., et al. Anti-angiogenesis through noninvasive to minimally invasive intraocular delivery of the peptide CC12 identified by in vivo-directed evolution. *Biomaterials* **2017**, *112*, 218-233, doi:10.1016/j.biomaterials.2016.09.022.

212. Kondo, E.; Saito, K.; Tashiro, Y.; Kamide, K.; Uno, S.; Furuya, T.; Mashita, M.; Nakajima, K.; Tsumuraya, T.; Kobayashi, N., et al. Tumour lineage-homing cell-penetrating peptides as anticancer molecular delivery systems. *Nature Communications* **2012**, *3*, 951, doi:10.1038/ncomms1952.

213. Li, T.; Liu, Q.; Chen, H.; Li, J. Antibacterial activity and mechanism of the cell-penetrating peptide CF-14 on the gram-negative bacteria, Escherichia coli. *Fish & Shellfish Immunology* **2020**, <https://doi.org/10.1016/j.fsi.2020.03.038>, doi:<https://doi.org/10.1016/j.fsi.2020.03.038>.

214. Aguiar, L.; Biosca, A.; Lantero, E.; Gut, J.; Vale, N.; Rosenthal, J.P.; Nogueira, F.; Andreu, D.; Fernàndez-Busquets, X.; Gomes, P. Coupling the Antimalarial Cell Penetrating Peptide TP10 to Classical Antimalarial Drugs Primaquine and Chloroquine Produces Strongly Hemolytic Conjugates. *Molecules* **2019**, *24*, doi:10.3390/molecules24244559.

215. de Coupade, C.; Fittipaldi, A.; Chagnas, V.; Michel, M.; Carlier, S.; Tasciotti, E.; Darmon, A.; Ravel, D.; Kearsey, J.; Giacca, M., et al. Novel human-derived cell-penetrating peptides for specific subcellular delivery of therapeutic biomolecules. *Biochemical Journal* **2005**, *390*, 407-418, doi:10.1042/BJ20050401.

216. Dong, C.; Lyu, S.-C.; Krensky, A.; Clayberger, C. DQ 65–79, A Peptide Derived from HLA Class II, Mimics p21 to Block T Cell Proliferation. *The Journal of Immunology* **2003**, *171*, 5064-5070, doi:10.4049/jimmunol.171.10.5064.

217. Klein, M.J.; Schmidt, S.; Wadhwani, P.; Bürck, J.; Reichert, J.; Afonin, S.; Berditsch, M.; Schober, T.; Brock, R.; Kansy, M., et al. Lactam-Stapled Cell-Penetrating Peptides: Cell Uptake and Membrane Binding Properties. *Journal of Medicinal Chemistry* **2017**, *60*, 8071-8082, doi:10.1021/acs.jmedchem.7b00813.

218. Akkarawongsa, R.; Cullinan, A.E.; Zinkel, A.; Clarin, J.; Brandt, C.R. Corneal Toxicity of Cell-Penetrating Peptides That Inhibit Herpes simplex Virus Entry. *Journal of Ocular Pharmacology and Therapeutics* **2006**, *22*, 279-289, doi:10.1089/jop.2006.22.279.

219. Kamei, N.; Morishita, M.; Eda, Y.; Ida, N.; Nishio, R.; Takayama, K. Usefulness of cell-penetrating peptides to improve intestinal insulin absorption. *Journal of Controlled Release* **2008**, *132*, 21-25, doi:<https://doi.org/10.1016/j.jconrel.2008.08.001>.

220. Forsman, H.; Bylund, J.; Oprea, T.I.; Karlsson, A.; Boulay, F.; Rabiet, M.-J.; Dahlgren, C. The leukocyte chemotactic receptor FPR2, but not the closely related FPR1, is sensitive to cell-penetrating pepducins with amino acid sequences descending from the third intracellular receptor loop. *Biochimica et Biophysica Acta (BBA) - Molecular Cell Research* **2013**, *1833*, 1914-1923, doi:<https://doi.org/10.1016/j.bbamcr.2013.03.026>.

221. Erratum: Cell penetrating peptide-modified nanoparticles for tumor targeted imaging and synergistic effect of sonodynamic/HIFU therapy [Corrigendum]. In *International Journal of Nanomedicine*, 2019; Vol. 14, p 6867.

222. Brugnano, J.L.; Chan, B.K.; Seal, B.L.; Panitch, A. Cell-penetrating peptides can confer biological function: Regulation of inflammatory cytokines in human monocytes by MK2 inhibitor peptides. *Journal of Controlled Release* **2011**, *155*, 128-133, doi:<https://doi.org/10.1016/j.jconrel.2011.05.007>.

223. Yamashita, H.; Demizu, Y.; Shoda, T.; Sato, Y.; Oba, M.; Tanaka, M.; Kurihara, M. Amphipathic short helix-stabilized peptides with cell-membrane penetrating ability. *Bioorganic & Medicinal Chemistry* **2014**, *22*, 2403-2408, doi:<https://doi.org/10.1016/j.bmc.2014.03.005>.

224. Futaki, S.; Ohashi, W.; Suzuki, T.; Niwa, M.; Tanaka, S.; Ueda, K.; Harashima, H.; Sugiura, Y. Stearylated Arginine-Rich Peptides:  A New Class of Transfection Systems. *Bioconjugate Chemistry* **2001**, *12*, 1005-1011, doi:10.1021/bc015508l.

225. Ueda, Y.; Wei, F.-Y.; Hide, T.-i.; Michiue, H.; Takayama, K.; Kaitsuka, T.; Nakamura, H.; Makino, K.; Kuratsu, J.-i.; Futaki, S., et al. Induction of autophagic cell death of glioma-initiating cells by cell-penetrating d-isomer peptides consisting of Pas and the p53 C-terminus. *Biomaterials* **2012**, *33*, 9061-9069, doi:<https://doi.org/10.1016/j.biomaterials.2012.09.003>.

226. Tan, Y.-X.; Chen, C.; Wang, Y.-L.; Lin, S.; Wang, Y.; Li, S.-B.; Jin, X.-P.; Gao, H.-W.; Du, F.-S.; Gong, F., et al. Truncated peptides from melittin and its analog with high lytic activity at endosomal pH enhance branched polyethylenimine-mediated gene transfection. *The Journal of Gene Medicine* **2012**, *14*, 241-250, doi:10.1002/jgm.2609.

227. Fernández-Carneado, J.; Kogan, M.J.; Van Mau, N.; Pujals, S.; López-Iglesias, C.; Heitz, F.; Giralt, E. Fatty acyl moieties: improving Pro-rich peptide uptake inside HeLa cells. *The Journal of Peptide Research* **2005**, *65*, 580-590, doi:10.1111/j.1399-3011.2005.00253.x.

228. Váňová, J.; Hejtmánková, A.; Žáčková Suchanová, J.; Sauerová, P.; Forstová, J.; Hubálek Kalbáčová, M.; Španielová, H. Influence of cell-penetrating peptides on the activity and stability of virus-based nanoparticles. *International Journal of Pharmaceutics* **2020**, *576*, 119008, doi:<https://doi.org/10.1016/j.ijpharm.2019.119008>.

229. Li, W.; Nicol, F.; Szoka, F.C. GALA: a designed synthetic pH-responsive amphipathic peptide with applications in drug and gene delivery. *Advanced Drug Delivery Reviews* **2004**, *56*, 967-985, doi:<https://doi.org/10.1016/j.addr.2003.10.041>.

230. Schach, D.K.; Rock, W.; Franz, J.; Bonn, M.; Parekh, S.H.; Weidner, T. Reversible Activation of a Cell-Penetrating Peptide in a Membrane Environment. *Journal of the American Chemical Society* **2015**, *137*, 12199-12202, doi:10.1021/jacs.5b06720.

231. Santiwarangkool, S.; Akita, H.; Nakatani, T.; Kusumoto, K.; Kimura, H.; Suzuki, M.; Nishimura, M.; Sato, Y.; Harashima, H. PEGylation of the GALA Peptide Enhances the Lung-Targeting Activity of Nanocarriers That Contain Encapsulated siRNA. *Journal of pharmaceutical sciences* **2017**, *106*, 2420-2427, doi:10.1016/j.xphs.2017.04.075.

232. Raftery, R.M.; Walsh, D.P.; Blokpoel Ferreras, L.; Mencía Castaño, I.; Chen, G.; LeMoine, M.; Osman, G.; Shakesheff, K.M.; Dixon, J.E.; O'Brien, F.J. Highly versatile cell-penetrating peptide loaded scaffold for efficient and localised gene delivery to multiple cell types: From development to application in tissue engineering. *Biomaterials* **2019**, *216*, 119277, doi:<https://doi.org/10.1016/j.biomaterials.2019.119277>.

233. Galdiero, S.; Falanga, A.; Vitiello, M.; Browne, H.; Pedone, C.; Galdiero, M. Fusogenic Domains in Herpes Simplex Virus Type 1 Glycoprotein H. *Journal of Biological Chemistry* **2005**, *280*, 28632-28643.

234. Perillo, E.; Hervé-Aubert, K.; Allard-Vannier, E.; Falanga, A.; Galdiero, S.; Chourpa, I. Synthesis and in vitro evaluation of fluorescent and magnetic nanoparticles functionalized with a cell penetrating peptide for cancer theranosis. *Journal of Colloid and Interface Science* **2017**, *499*, 209-217, doi:<https://doi.org/10.1016/j.jcis.2017.03.106>.

235. Perillo, E.; Allard-Vannier, E.; Falanga, A.; Stiuso, P.; Vitiello, M.T.; Galdiero, M.; Galdiero, S.; Chourpa, I. Quantitative and qualitative effect of gH625 on the nanoliposome-mediated delivery of mitoxantrone anticancer drug to HeLa cells. *International journal of pharmaceutics* **2015**, *488*, 59-66, doi:10.1016/j.ijpharm.2015.04.039.

236. Falanga, A.; Vitiello, M.T.; Cantisani, M.; Tarallo, R.; Guarnieri, D.; Mignogna, E.; Netti, P.; Pedone, C.; Galdiero, M.; Galdiero, S. A peptide derived from herpes simplex virus type 1 glycoprotein H: membrane translocation and applications to the delivery of quantum dots. *Nanomedicine: Nanotechnology, Biology and Medicine* **2011**, *7*, 925-934, doi:<https://doi.org/10.1016/j.nano.2011.04.009>.

237. Ben Djemaa, S.; Hervé-Aubert, K.; Lajoie, L.; Falanga, A.; Galdiero, S.; Nedellec, S.; Soucé, M.; Munnier, E.; Chourpa, I.; David, S., et al. gH625 Cell-Penetrating Peptide Promotes the Endosomal Escape of Nanovectorized siRNA in a Triple-Negative Breast Cancer Cell Line. *Biomacromolecules* **2019**, *20*, 3076-3086, doi:10.1021/acs.biomac.9b00637.

238. Ben Djemaa, S.; David, S.; Hervé-Aubert, K.; Falanga, A.; Galdiero, S.; Allard-Vannier, E.; Chourpa, I.; Munnier, E. Formulation and in vitro evaluation of a siRNA delivery nanosystem decorated with gH625 peptide for triple negative breast cancer theranosis. *European Journal of Pharmaceutics and Biopharmaceutics* **2018**, *131*, 99-108, doi:<https://doi.org/10.1016/j.ejpb.2018.07.024>.

239. Galdiero, S.; Russo, L.; Falanga, A.; Cantisani, M.; Vitiello, M.; Fattorusso, R.; Malgieri, G.; Galdiero, M.; Isernia, C. Structure and Orientation of the gH625–644 Membrane Interacting Region of Herpes Simplex Virus Type 1 in a Membrane Mimetic System. *Biochemistry* **2012**, *51*, 3121-3128, doi:10.1021/bi201589m.

240. Tarallo, R.; Accardo, A.; Falanga, A.; Guarnieri, D.; Vitiello, G.; Netti, P.; D'Errico, G.; Morelli, G.; Galdiero, S. Clickable Functionalization of Liposomes with the gH625 Peptide from Herpes simplex Virus Type I for Intracellular Drug Delivery. *Chemistry – A European Journal* **2011**, *17*, 12659-12668, doi:10.1002/chem.201101425.

241. Smaldone, G.; Falanga, A.; Capasso, D.; Guarnieri, D.; Correale, S.; Galdiero, M.; Netti, P.A.; Zollo, M.; Galdiero, S.; Di Gaetano, S., et al. gH625 is a viral derived peptide for effective delivery of intrinsically disordered proteins. *International journal of nanomedicine* **2013**, *8*, 2555-2565, doi:10.2147/IJN.S44186.

242. Hayashi, T.; Shinagawa, M.; Kawano, T.; Iwasaki, T. Drug delivery using polyhistidine peptide-modified liposomes that target endogenous lysosome. *Biochemical and Biophysical Research Communications* **2018**, *501*, 648-653, doi:<https://doi.org/10.1016/j.bbrc.2018.05.037>.

243. Leng, Q.; Scaria, P.; Lu, P.; Woodle, M.C.; Mixson, A.J. Systemic delivery of HK Raf-1 siRNA polyplexes inhibits MDA-MB-435 xenografts. *Cancer Gene Therapy* **2008**, *15*, 485-495, doi:10.1038/cgt.2008.29.

244. Lee, J.Y.; Suh, J.S.; Kim, J.M.; Kim, J.H.; Park, H.J.; Park, Y.J.; Chung, C.P. Identification of a cell-penetrating peptide domain from human beta-defensin 3 and characterization of its anti-inflammatory activity. *International journal of nanomedicine* **2015**, *10*, 5423-5434, doi:10.2147/IJN.S90014.

245. Bhattacharya, P.; Basak, A.; Campbell, A.; Alabugin, I.V. Photochemical Activation of Enediyne Warheads: A Potential Tool for Targeted Antitumor Therapy. *Molecular Pharmaceutics* **2018**, *15*, 768-797, doi:10.1021/acs.molpharmaceut.7b00911.

246. Schmidt, M.C.; Rothen-Rutishauser, B.; Rist, B.; Beck-Sickinger, A.; Wunderli-Allenspach, H.; Rubas, W.; Sadée, W.; Merkle, H.P. Translocation of Human Calcitonin in Respiratory Nasal Epithelium Is Associated with Self-Assembly in Lipid Membrane. *Biochemistry* **1998**, *37*, 16582-16590, doi:10.1021/bi981219h.

247. Tréhin, R.; Krauss, U.; Muff, R.; Meinecke, M.; Beck-Sickinger, A.G.; Merkle, H.P. Cellular Internalization of Human Calcitonin Derived Peptides in MDCK Monolayers: A Comparative Study with Tat(47-57) and Penetratin(43-58). *Pharmaceutical Research* **2004**, *21*, 33-42, doi:10.1023/B:PHAM.0000012149.83119.bf.

248. Neundorf, I.; Rennert, R.; Hoyer, J.; Schramm, F.; Löbner, K.; Kitanovic, I.; Wölfl, S. Fusion of a Short HA2-Derived Peptide Sequence to Cell-Penetrating Peptides Improves Cytosolic Uptake, but Enhances Cytotoxic Activity. *Pharmaceuticals* **2009**, *2*, doi:10.3390/ph2020049.

249. Richter, S.; Bouvet, V.; Wuest, M.; Bergmann, R.; Steinbach, J.; Pietzsch, J.; Neundorf, I.; Wuest, F. 18F-Labeled phosphopeptide-cell-penetrating peptide dimers with enhanced cell uptake properties in human cancer cells. *Nuclear Medicine and Biology* **2012**, *39*, 1202-1212, doi:<https://doi.org/10.1016/j.nucmedbio.2012.06.003>.

250. Duchardt, F.; Ruttekolk, I.R.; Verdurmen, W.P.; Lortat-Jacob, H.; Burck, J.; Hufnagel, H.; Fischer, R.; van den Heuvel, M.; Lowik, D.W.P.M.; Vuister, G.W., et al. A cell-penetrating peptide derived from human lactoferrin with conformation-dependent uptake efficiency. *Journal of Biological Chemistry* **2009**.

251. Youn, J.I.; Park, S.H.; Jin, H.T.; Lee, C.G.; Seo, S.H.; Song, M.Y.; Lee, C.W.; Sung, Y.C. Enhanced delivery efficiency of recombinant adenovirus into tumor and mesenchymal stem cells by a novel PTD. *Cancer Gene Therapy* **2008**, *15*, 703-712, doi:10.1038/cgt.2008.45.

252. Park, S.H.; Doh, J.; Park, S.I.; Lim, J.Y.; Kim, S.M.; Youn, J.I.; Jin, H.T.; Seo, S.H.; Song, M.Y.; Sung, S.Y., et al. Branched oligomerization of cell-permeable peptides markedly enhances the transduction efficiency of adenovirus into mesenchymal stem cells. *Gene therapy* **2010**, *17*, 1052-1061, doi:10.1038/gt.2010.58.

253. Lim, J.; Kim, J.; Duong, T.; Lee, G.; Kim, J.; Yoon, J.; Kim, J.; Kim, H.; Ruley, H.E.; El-Rifai, W., et al. Antitumor activity of cell-permeable p18(INK4c) with enhanced membrane and tissue penetration. *Molecular therapy : the journal of the American Society of Gene Therapy* **2012**, *20*, 1540-1549, doi:10.1038/mt.2012.102.

254. Li, H.; Zheng, X.; Koren, V.; Vashist, Y.K.; Tsui, T.Y. Highly efficient delivery of siRNA to a heart transplant model by a novel cell penetrating peptide-dsRNA binding domain. *International journal of pharmaceutics* **2014**, *469*, 206-213, doi:10.1016/j.ijpharm.2014.04.050.

255. Koo, J.-H.; Yoon, H.; Kim, W.-J.; Cha, D.; Choi, J.-M. Cell-Penetrating Function of the Poly(ADP-Ribose) (PAR)-Binding Motif Derived from the PAR-Dependent E3 Ubiquitin Ligase Iduna. *International Journal of Molecular Sciences* **2018**, *19*, doi:10.3390/ijms19030779.

256. Wang, H.; Ma, J.; Yang, Y.; Zeng, F.; Liu, C. Highly Efficient Delivery of Functional Cargoes by a Novel Cell-Penetrating Peptide Derived from SP140-Like Protein. *Bioconjugate Chemistry* **2016**, *27*, 1373-1381, doi:10.1021/acs.bioconjchem.6b00161.

257. Alizadeh, S.; Irani, S.; Bolhassani, A.; Sadat, S.M. HR9: An Important Cell Penetrating Peptide for Delivery of HCV NS3 DNA into HEK-293T Cells. *Avicenna journal of medical biotechnology* **2020**, *12*, 44-51.

258. Anderson, D.S.; Hobbs, J.R.; Gwenin, V.V.; Ball, P.; Bennie, A.L.; Coulter, A.J.; Gwenin, D.C. Cell-Penetrating Peptides as a Tool for the Cellular Uptake of a Genetically Modified Nitroreductase for use in Directed Enzyme Prodrug Therapy. *Journal of Functional Biomaterials* **2019**, *10*, doi:10.3390/jfb10040045.

259. Dai, Y.; Yue, N.; Gong, J.; Liu, C.; Li, Q.; Zhou, J.; Huang, W.; Qian, H. Development of cell-permeable peptide-based PROTACs targeting estrogen receptor α. *European Journal of Medicinal Chemistry* **2020**, *187*, 111967, doi:<https://doi.org/10.1016/j.ejmech.2019.111967>.

260. Gautam, A.; Nanda, J.S.; Samuel, J.S.; Kumari, M.; Priyanka, P.; Bedi, G.; Nath, S.K.; Mittal, G.; Khatri, N.; Raghava, G.P.S. Topical Delivery of Protein and Peptide Using Novel Cell Penetrating Peptide IMT-P8. *Scientific Reports* **2016**, *6*, 26278, doi:10.1038/srep26278.

261. Wolfert, M.A.; Seymour, L.W. Chloroquine and amphipathic peptide helices show synergistic transfection in vitro. *Gene Therapy* **1998**, *5*, 409-414, doi:10.1038/sj.gt.3300606.

262. Puig-Saus, C.; Rojas, L.A.; Laborda, E.; Figueras, A.; Alba, R.; Fillat, C.; Alemany, R. iRGD tumor-penetrating peptide-modified oncolytic adenovirus shows enhanced tumor transduction, intratumoral dissemination and antitumor efficacy. *Gene therapy* **2014**, *21*, 767-774, doi:10.1038/gt.2014.52.

263. Yu, K.-F.; Zhang, W.-Q.; Luo, L.-M.; Song, P.; Li, D.; Du, R.; Ren, W.; Huang, D.; Lu, W.-L.; Zhang, X., et al. The antitumor activity of a doxorubicin loaded, iRGD-modified sterically-stabilized liposome on B16-F10 melanoma cells: in vitro and in vivo evaluation. *International journal of nanomedicine* **2013**, *8*, 2473-2485, doi:10.2147/IJN.S46962.

264. Du, R.; Zhong, T.; Zhang, W.-Q.; Song, P.; Song, W.-D.; Zhao, Y.; Wang, C.; Tang, Y.-Q.; Zhang, X.; Zhang, Q. Antitumor effect of iRGD-modified liposomes containing conjugated linoleic acid-paclitaxel (CLA-PTX) on B16-F10 melanoma. *International journal of nanomedicine* **2014**, *9*, 3091-3105, doi:10.2147/IJN.S65664.

265. Chu, Y.; Chen, N.; Yu, H.; Mu, H.; He, B.; Hua, H.; Wang, A.; Sun, K. Topical ocular delivery to laser-induced choroidal neovascularization by dual internalizing RGD and TAT peptide-modified nanoparticles. *International journal of nanomedicine* **2017**, *12*, 1353-1368, doi:10.2147/IJN.S126865.

266. Russo, M.A.; Paolillo, M.; Sanchez-Hernandez, Y.; Curti, D.; Ciusani, E.; Serra, M.; Colombo, L.; Schinelli, S. A small-molecule RGD-integrin antagonist inhibits cell adhesion, cell migration and induces anoikis in glioblastoma cells. *International journal of oncology* **2013**, *42*, 83-92, doi:10.3892/ijo.2012.1708.

267. Zhang, Q.; Zhang, Y.; Li, K.; Wang, H.; Li, H.; Zheng, J. A Novel Strategy to Improve the Therapeutic Efficacy of Gemcitabine for Non-Small Cell Lung Cancer by the Tumor-Penetrating Peptide iRGD. *PLOS ONE* **2015**, *10*, e0129865, doi:10.1371/journal.pone.0129865.

268. Lao, X.; Li, B.; Liu, M.; Chen, J.; Gao, X.; Zheng, H. Increased antitumor activity of tumor-specific peptide modified thymopentin. *Biochimie* **2014**, *107*, 277-285, doi:<https://doi.org/10.1016/j.biochi.2014.09.013>.

269. Li, M.; Tang, Z.; Zhang, D.; Sun, H.; Liu, H.; Zhang, Y.; Zhang, Y.; Chen, X. Doxorubicin-loaded polysaccharide nanoparticles suppress the growth of murine colorectal carcinoma and inhibit the metastasis of murine mammary carcinoma in rodent models. *Biomaterials* **2015**, *51*, 161-172, doi:<https://doi.org/10.1016/j.biomaterials.2015.02.002>.

270. Song, W.; Li, M.; Tang, Z.; Li, Q.; Yang, Y.; Liu, H.; Duan, T.; Hong, H.; Chen, X. Methoxypoly(ethylene glycol)-block-Poly(L-glutamic acid)-Loaded Cisplatin and a Combination With iRGD for the Treatment of Non-Small-Cell Lung Cancers. *Macromolecular Bioscience* **2012**, *12*, 1514-1523, doi:10.1002/mabi.201200145.

271. Mao, X.; Liu, J.; Gong, Z.; Zhang, H.; Lu, Y.; Zou, H.; Yu, Y.; Chen, Y.; Sun, Z.; Li, W., et al. iRGD-conjugated DSPE-PEG2000 nanomicelles for targeted delivery of salinomycin for treatment of both liver cancer cells and cancer stem cells. *Nanomedicine (London, England)* **2015**, *10*, 2677-2695, doi:10.2217/nnm.15.106.

272. Sugahara, K.N.; Teesalu, T.; Karmali, P.P.; Kotamraju, V.R.; Agemy, L.; Greenwald, D.R.; Ruoslahti, E. Coadministration of a tumor-penetrating peptide enhances the efficacy of cancer drugs. *Science (New York, N.Y.)* **2010**, *328*, 1031-1035, doi:10.1126/science.1183057.

273. Werfel, T.A.; Elion, D.L.; Rahman, B.; Hicks, D.J.; Sanchez, V.; Gonzalez-Ericsson, P.I.; Nixon, M.J.; James, J.L.; Balko, J.M.; Scherle, P., et al. Treatment-induced tumor cell apoptosis and secondary necrosis drive tumor progression in the residual tumor microenvironment through MerTK and IDO-1. *Cancer Research* **2018**, 10.1158/0008-5472.CAN-18-1106, canres.1106.2018, doi:10.1158/0008-5472.CAN-18-1106.

274. Sha, H.; Zou, Z.; Xin, K.; Bian, X.; Cai, X.; Lu, W.; Chen, J.; Chen, G.; Huang, L.; Blair, A.M., et al. Tumor-penetrating peptide fused EGFR single-domain antibody enhances cancer drug penetration into 3D multicellular spheroids and facilitates effective gastric cancer therapy. *Journal of Controlled Release* **2015**, *200*, 188-200, doi:<https://doi.org/10.1016/j.jconrel.2014.12.039>.

275. De, G.; Ko, J.-K.; Lin, P.; Kaumaya, P.; Li, H.; Ma, J. Amphipathic Tail-Anchoring Peptide is a Promising Therapeutic Agent for Cancer Treatment. *Biophysical Journal* **2014**, *106*, 186a, doi:10.1016/j.bpj.2013.11.1049.

276. Cheng, W.; Feng, F.; Ma, C.; Wang, H. The effect of antagonizing RGD-binding integrin activity in papillary thyroid cancer cell lines. *OncoTargets and therapy* **2016**, *9*, 1415-1423, doi:10.2147/OTT.S99166.

277. Ji, X.; Lv, H.; Guo, J.; Ding, C.; Luo, X. A DNA Nanotube–Peptide Biocomplex for mRNA Detection and Its Application in Cancer Diagnosis and Targeted Therapy. *Chemistry – A European Journal* **2018**, *24*, 10171-10177, doi:10.1002/chem.201801347.

278. Mai, J.C.; Shen, H.; Watkins, S.C.; Cheng, T.; Robbins, P.D. Efficiency of Protein Transduction Is Cell Type-dependent and Is Enhanced by Dextran Sulfate. *Journal of Biological Chemistry* **2002**, *277*, 30208-30218.

279. Ndeboko, B.; Ramamurthy, N.; Lemamy, G.J.; Jamard, C.; Nielsen, P.E.; Cova, L. Role of Cell-Penetrating Peptides in Intracellular Delivery of Peptide Nucleic Acids Targeting Hepadnaviral Replication. *Molecular Therapy - Nucleic Acids* **2017**, *9*, 162-169, doi:<https://doi.org/10.1016/j.omtn.2017.09.003>.

280. Xu, D.; Dustin, D.; Jiang, L.; Samways, D.S.K.; Dong, H. Designed filamentous cell penetrating peptides: probing supramolecular structure-dependent membrane activity and transfection efficiency. *Chemical Communications* **2015**, *51*, 11757-11760, doi:10.1039/C5CC02699G.

281. Zhang, W.; Song, J.; Liang, R.; Zheng, X.; Chen, J.; Li, G.; Zhang, B.; Wang, K.; Yan, X.; Wang, R. Stearylated antimicrobial peptide [D]-K6L9 with cell penetrating property for efficient gene transfer. *Peptides* **2013**, *46*, 33-39, doi:<https://doi.org/10.1016/j.peptides.2013.05.011>.

282. Barkowsky, G.; Lemster, A.-L.; Pappesch, R.; Jacob, A.; Krüger, S.; Schröder, A.; Kreikemeyer, B.; Patenge, N. Influence of Different Cell-Penetrating Peptides on the Antimicrobial Efficiency of PNAs in Streptococcus pyogenes. *Molecular Therapy - Nucleic Acids* **2019**, *18*, 444-454, doi:<https://doi.org/10.1016/j.omtn.2019.09.010>.

283. Park, J.; Ryu, J.; Kim, K.-A.; Lee, H.J.; Bahn, J.H.; Han, K.; Choi, E.Y.; Lee, K.S.; Kwon, H.Y.; Choi, S.Y. Mutational analysis of a human immunodeficiency virus type 1 Tat protein transduction domain which is required for delivery of an exogenous protein into mammalian cells. *Journal of General Virology* **2002**, *83*.

284. Patel, L.N.; Wang, J.; Kim, K.-J.; Borok, Z.; Crandall, E.D.; Shen, W.-C. Conjugation with Cationic Cell-Penetrating Peptide Increases Pulmonary Absorption of Insulin. *Molecular Pharmaceutics* **2009**, *6*, 492-503, doi:10.1021/mp800174g.

285. Ferreira, A.J.; Cemlyn-Jones, J.; Robalo Cordeiro, C. Nanoparticles, nanotechnology and pulmonary nanotoxicology. *Revista portuguesa de pneumologia* **2013**, *19*, 28-37, doi:10.1016/j.rppneu.2012.09.003.

286. Bartlett, R.L.; Panitch, A. Thermosensitive Nanoparticles with pH-Triggered Degradation and Release of Anti-inflammatory Cell-Penetrating Peptides. *Biomacromolecules* **2012**, *13*, 2578-2584, doi:10.1021/bm300826v.

287. Guo, L.; Fan, L.; Pang, Z.; Ren, J.; Ren, Y.; Li, J.; Chen, J.; Wen, Z.; Jiang, X. TRAIL and doxorubicin combination enhances anti-glioblastoma effect based on passive tumor targeting of liposomes. *Journal of Controlled Release* **2011**, *154*, 93-102, doi:<https://doi.org/10.1016/j.jconrel.2011.05.008>.

288. Wyman, T.B.; Nicol, F.; Zelphati, O.; Scaria, P.V.; Plank, C.; Szoka, F.C. Design, Synthesis, and Characterization of a Cationic Peptide That Binds to Nucleic Acids and Permeabilizes Bilayers. *Biochemistry* **1997**, *36*, 3008-3017, doi:10.1021/bi9618474.

289. Bendifallah, N.; Rasmussen, F.W.; Zachar, V.; Ebbesen, P.; Nielsen, P.E.; Koppelhus, U. Evaluation of Cell-Penetrating Peptides (CPPs) as Vehicles for Intracellular Delivery of Antisense Peptide Nucleic Acid (PNA). *Bioconjugate Chemistry* **2006**, *17*, 750-758, doi:10.1021/bc050283q.

290. Sauter, M.; Strieker, M.; Kleist, C.; Wischnjow, A.; Daniel, V.; Altmann, A.; Haberkorn, U.; Mier, W. Improving antibody-based therapies by chemical engineering of antibodies with multimeric cell-penetrating peptides for elevated intracellular delivery. *Journal of Controlled Release* **2020**, *322*, 200-208, doi:<https://doi.org/10.1016/j.jconrel.2020.03.005>.

291. Monreal, I.A.; Liu, Q.; Tyson, K.; Bland, T.; Dalisay, D.S.; Adams, E.V.; Wayman, G.A.; Aguilar, H.C.; Saludes, J.P. Branched dimerization of Tat peptide improves permeability to HeLa and hippocampal neuronal cells. *Chemical Communications* **2015**, *51*, 5463-5466, doi:10.1039/C5CC00882D.

292. Scheller, A.; Oehlke, J.; Wiesner, B.; Dathe, M.; Krause, E.; Beyermann, M.; Melzig, M.; Bienert, M. Structural requirements for cellular uptake of α-helical amphipathic peptides. *Journal of Peptide Science* **1999**, *5*, 185-194, doi:10.1002/(SICI)1099-1387(199904)5:4<185::AID-PSC184>3.0.CO;2-9.

293. Oehlke, J.; Scheller, A.; Janek, K.; Wiesner, B.; Krause, E.; Beyermann, M.; Bienert, M. Rapid translocation of amphipathic βhelical and β-sheet-forming peptides through plasma membranes of endothelial cells. In *Peptide Science — Present and Future: Proceedings of the 1st International Peptide Symposium*, Shimonishi, Y., Ed. Springer Netherlands: Dordrecht, 2002; 10.1007/0-306-46864-6_268pp. 782-783.

294. Yishay-Safranchik, E.; Golan, M.; David, A. Controlled release of doxorubicin and Smac-derived pro-apoptotic peptide from self-assembled KLD-based peptide hydrogels. *Polymers for Advanced Technologies* **2014**, *25*, 539-544, doi:10.1002/pat.3300.

295. Balayssac, S.; Burlina, F.; Convert, O.; Bolbach, G.; Chassaing, G.; Lequin, O. Comparison of Penetratin and Other Homeodomain-Derived Cell-Penetrating Peptides:  Interaction in a Membrane-Mimicking Environment and Cellular Uptake Efficiency. *Biochemistry* **2006**, *45*, 1408-1420, doi:10.1021/bi0518390.

296. Maraming, P.; Klaynongsruang, S.; Boonsiri, P.; Peng, S.-F.; Daduang, S.; Leelayuwat, C.; Pientong, C.; Chung, J.-G.; Daduang, J. The cationic cell-penetrating KT2 peptide promotes cell membrane defects and apoptosis with autophagy inhibition in human HCT 116 colon cancer cells. *Journal of Cellular Physiology* **2019**, *234*, 22116-22129, doi:10.1002/jcp.28774.

297. Akishiba, M.; Takeuchi, T.; Kawaguchi, Y.; Sakamoto, K.; Yu, H.-H.; Nakase, I.; Takatani-Nakase, T.; Madani, F.; Gräslund, A.; Futaki, S. Cytosolic antibody delivery by lipid-sensitive endosomolytic peptide. *Nature Chemistry* **2017**, *9*, 751-761, doi:10.1038/nchem.2779.

298. Gomes dos Reis, L.; Lee, W.-H.; Svolos, M.; Moir, L.M.; Jaber, R.; Engel, A.; Windhab, N.; Young, P.M.; Traini, D. Delivery of pDNA to lung epithelial cells using PLGA nanoparticles formulated with a cell-penetrating peptide: understanding the intracellular fate. *Drug Development and Industrial Pharmacy* **2020**, *46*, 427-442, doi:10.1080/03639045.2020.1724134.

299. Jamali, A.; Kapitza, L.; Schaser, T.; Johnston, I.C.D.; Buchholz, C.J.; Hartmann, J. Highly Efficient and Selective CAR-Gene Transfer Using CD4- and CD8-Targeted Lentiviral Vectors. *Molecular therapy. Methods & clinical development* **2019**, *13*, 371-379, doi:10.1016/j.omtm.2019.03.003.

300. Piovan, C.; Marin, V.; Scavullo, C.; Corna, S.; Giuliani, E.; Bossi, S.; Galy, A.; Fenard, D.; Bordignon, C.; Rizzardi, G.P., et al. Vectofusin-1 Promotes RD114-TR-Pseudotyped Lentiviral Vector Transduction of Human HSPCs and T Lymphocytes. *Molecular therapy. Methods & clinical development* **2017**, *5*, 22-30, doi:10.1016/j.omtm.2017.02.003.

301. Liu, Y.; Kim, Y.J.; Ji, M.; Fang, J.; Siriwon, N.; Zhang, L.I.; Wang, P. Enhancing gene delivery of adeno-associated viruses by cell-permeable peptides. *Molecular Therapy - Methods & Clinical Development* **2014**, *1*, 12, doi:<https://doi.org/10.1038/mtm.2013.12>.

302. Fenard, D.; Ingrao, D.; Seye, A.; Buisset, J.; Genries, S.; Martin, S.; Kichler, A.; Galy, A. Vectofusin-1, a new viral entry enhancer, strongly promotes lentiviral transduction of human hematopoietic stem cells. *Molecular therapy. Nucleic acids* **2013**, *2*, e90-e90, doi:10.1038/mtna.2013.17.

303. Fenard, D.; Genries, S.; Scherman, D.; Galy, A.; Martin, S.; Kichler, A. Infectivity enhancement of different HIV-1-based lentiviral pseudotypes in presence of the cationic amphipathic peptide LAH4-L1. *Journal of Virological Methods* **2013**, *189*, 375-378, doi:<https://doi.org/10.1016/j.jviromet.2013.02.005>.

304. Zhang, Y.; Li, L.; Chang, L.; Liu, H.; Song, J.; Liu, Y.; Bao, H.; Liu, B.; Wang, R.; Ni, J. Design of a new pH-activatable cell-penetrating peptide for drug delivery into tumor cells. *Chemical Biology & Drug Design* **2019**, *94*, 1884-1893, doi:10.1111/cbdd.13537.

305. Simón-Gracia, L.; Scodeller, P.; Fuentes, S.S.; Vallejo, V.G.; Ríos, X.; San Sebastián, E.; Sidorenko, V.; Di Silvio, D.; Suck, M.; De Lorenzi, F., et al. Application of polymersomes engineered to target p32 protein for detection of small breast tumors in mice. *Oncotarget* **2018**, *9*, 18682-18697, doi:10.18632/oncotarget.24588.

306. Kim, Y.; Hwang, S.; Khalmuratova, R.; Kang, S.; Lee, M.; Song, Y.; Park, J.-W.; Yu, J.; Shin, H.-W.; Lee, Y. α-Helical cell-penetrating peptide-mediated nasal delivery of resveratrol for inhibition of epithelial-to-mesenchymal transition. *Journal of Controlled Release* **2020**, *317*, 181-194, doi:<https://doi.org/10.1016/j.jconrel.2019.11.034>.

307. Hyun, S.; Li, L.; Yoon, K.C.; Yu, J. An amphipathic cell penetrating peptide aids cell penetration of cyclosporin A and increases its therapeutic effect in an in vivo mouse model for dry eye disease. *Chemical Communications* **2019**, *55*, 13657-13660, doi:10.1039/C9CC05960A.

308. Lim, S.; Kim, W.-j.; Kim, Y.-h.; Choi, J.-M. Identification of a novel cell-penetrating peptide from human phosphatidate phosphatase LPIN3. *Molecules and Cells* **2012**, *34*, 577-582, doi:10.1007/s10059-012-0284-y.

309. Yang, S.; Wang, D.; Sun, Y.; Zheng, B. Delivery of antisense oligonucleotide using polyethylenimine-based lipid nanoparticle modified with cell penetrating peptide. *Drug delivery* **2019**, *26*, 965-974, doi:10.1080/10717544.2019.1667453.

310. Dekiwadia, C.D.; Lawrie, A.C.; Fecondo, J.V. Peptide-mediated cell penetration and targeted delivery of gold nanoparticles into lysosomes. *Journal of peptide science : an official publication of the European Peptide Society* **2012**, *18*, 527-534, doi:10.1002/psc.2430.

311. El-Andaloussi, S.; Johansson, H.J.; Holm, T.; Langel, Ü. A Novel Cell-penetrating Peptide, M918, for Efficient Delivery of Proteins and Peptide Nucleic Acids. *Molecular Therapy* **2007**, *15*, 1820-1826, doi:10.1038/sj.mt.6300255.

312. Guterstam, P.; Madani, F.; Hirose, H.; Takeuchi, T.; Futaki, S.; El Andaloussi, S.; Gräslund, A.; Langel, U. Elucidating cell-penetrating peptide mechanisms of action for membrane interaction, cellular uptake, and translocation utilizing the hydrophobic counter-anion pyrenebutyrate. *Biochimica et biophysica acta* **2009**, *1788*, 2509-2517, doi:10.1016/j.bbamem.2009.09.014.

313. Bocsik, A.; Gróf, I.; Kiss, L.; Ötvös, F.; Zsíros, O.; Daruka, L.; Fülöp, L.; Vastag, M.; Kittel, Á.; Imre, N., et al. Dual Action of the PN159/KLAL/MAP Peptide: Increase of Drug Penetration across Caco-2 Intestinal Barrier Model by Modulation of Tight Junctions and Plasma Membrane Permeability. *Pharmaceutics* **2019**, *11*, doi:10.3390/pharmaceutics11020073.

314. Oehlke, J.; Birth, P.; Klauschenz, E.; Wiesner, B.; Beyermann, M.; Oksche, A.; Bienert, M. Cellular uptake of antisense oligonucleotides after complexing or conjugation with cell-penetrating model peptides. *European Journal of Biochemistry* **2002**, *269*, 4025-4032, doi:10.1046/j.1432-1033.2002.03093.x.

315. Wada, S.-i.; Hashimoto, Y.; Kawai, Y.; Miyata, K.; Tsuda, H.; Nakagawa, O.; Urata, H. Effect of Ala replacement with Aib in amphipathic cell-penetrating peptide on oligonucleotide delivery into cells. *Bioorganic & Medicinal Chemistry* **2013**, *21*, 7669-7673, doi:<https://doi.org/10.1016/j.bmc.2013.10.029>.

316. Oehlke, J.; Scheller, A.; Wiesner, B.; Krause, E.; Beyermann, M.; Klauschenz, E.; Melzig, M.; Bienert, M. Cellular uptake of an α-helical amphipathic model peptide with the potential to deliver polar compounds into the cell interior non-endocytically. *Biochimica et Biophysica Acta (BBA) - Biomembranes* **1998**, *1414*, 127-139, doi:<https://doi.org/10.1016/S0005-2736(98)00161-8>.

317. Perret, P.; Ahmadi, M.; Riou, L.; Bacot, S.; Pecher, J.; Poillot, C.; Broisat, A.; Ghezzi, C.; De Waard, M. Biodistribution, Stability, and Blood Distribution of the Cell Penetrating Peptide Maurocalcine in Mice. *International Journal of Molecular Sciences* **2015**, *16*, doi:10.3390/ijms161126054.

318. Aroui, S.; Dardevet, L.; Ajmia, W.B.; de Boisvilliers, M.; Perrin, F.; Laajimi, A.; Boumendjel, A.; Kenani, A.; Muller, J.M.; De Waard, M. A Novel Platinum–Maurocalcine Conjugate Induces Apoptosis of Human Glioblastoma Cells by Acting through the ROS-ERK/AKT-p53 Pathway. *Molecular Pharmaceutics* **2015**, *12*, 4336-4348, doi:10.1021/acs.molpharmaceut.5b00531.

319. Crombez, L.; Morris, M.C.; Dufort, S.; Aldrian-Herrada, G.; Nguyen, Q.; Mc Master, G.; Coll, J.-L.; Heitz, F.; Divita, G. Targeting cyclin B1 through peptide-based delivery of siRNA prevents tumour growth. *Nucleic Acids Research* **2009**, *37*, 4559-4569, doi:10.1093/nar/gkp451.

320. Mäe, M.; El Andaloussi, S.; Lundin, P.; Oskolkov, N.; Johansson, H.J.; Guterstam, P.; Langel, U. A stearylated CPP for delivery of splice correcting oligonucleotides using a non-covalent co-incubation strategy. *Journal of controlled release : official journal of the Controlled Release Society* **2009**, *134*, 221-227, doi:10.1016/j.jconrel.2008.11.025.

321. Kwon, S.-J.; Han, K.; Jung, S.; Lee, J.-E.; Park, S.; Cheon, Y.-P.; Lim, H.J. Transduction of the MPG-tagged fusion protein into mammalian cells and oocytes depends on amiloride-sensitive endocytic pathway. *BMC Biotechnology* **2009**, *9*, 73, doi:10.1186/1472-6750-9-73.

322. Morris, M.C.; Vidal, P.; Chaloin, L.; Heitz, F.; Divita, G. A new peptide vector for efficient delivery of oligonucleotides into mammalian cells. *Nucleic acids research* **1997**, *25*, 2730-2736, doi:10.1093/nar/25.14.2730.

323. Simeoni, F.; Morris, M.C.; Heitz, F.; Divita, G. Insight into the mechanism of the peptide‐based gene delivery system MPG: implications for delivery of siRNA into mammalian cells. *Nucleic Acids Research* **2003**, *31*, 2717-2724, doi:10.1093/nar/gkg385.

324. Veldhoen, S.; Laufer, S.D.; Trampe, A.; Restle, T. Cellular delivery of small interfering RNA by a non-covalently attached cell-penetrating peptide: quantitative analysis of uptake and biological effect. *Nucleic Acids Research* **2006**, *34*, 6561-6573, doi:10.1093/nar/gkl941.

325. Delaroche, D.; Aussedat, B.; Aubry, S.; Chassaing, G.; Burlina, F.; Clodic, G.; Bolbach, G.; Lavielle, S.; Sagan, S. Tracking a New Cell-Penetrating (W/R) Nonapeptide, through an Enzyme-Stable Mass Spectrometry Reporter Tag. *Analytical Chemistry* **2007**, *79*, 1932-1938, doi:10.1021/ac061108l.

326. Jain, A.; Shah, S.G.; Chugh, A. Cell Penetrating Peptides as Efficient Nanocarriers for Delivery of Antifungal Compound, Natamycin for the Treatment of Fungal Keratitis. *Pharmaceutical Research* **2015**, *32*, 1920-1930, doi:10.1007/s11095-014-1586-x.

327. Cerrato, C.P.; Pirisinu, M.; Vlachos, E.N.; Langel, Ü. Novel cell-penetrating peptide targeting mitochondria. *The FASEB Journal* **2015**, *29*, 4589-4599, doi:10.1096/fj.14-269225.

328. Jo, D.; Liu, D.; Yao, S.; Collins, R.D.; Hawiger, J. Intracellular protein therapy with SOCS3 inhibits inflammation and apoptosis. *Nature Medicine* **2005**, *11*, 892-898, doi:10.1038/nm1269.

329. Rojas, M.; Donahue, J.P.; Tan, Z.; Lin, Y.-Z. Genetic engineering of proteins with cell membrane permeability. *Nature Biotechnology* **1998**, *16*, 370-375, doi:10.1038/nbt0498-370.

330. Binder, C.; Read, S.P.; Cashman, S.M.; Kumar-Singh, R. Nuclear targeted delivery of macromolecules to retina and cornea. *The Journal of Gene Medicine* **2011**, *13*, 158-170, doi:10.1002/jgm.1548.

331. Ragin, A.D.; Morgan, R.A.; Chmielewski, J. Cellular Import Mediated by Nuclear Localization Signal Peptide Sequences. *Chemistry & Biology* **2002**, *9*, 943-948, doi:<https://doi.org/10.1016/S1074-5521(02)00189-8>.

332. Curnis, F.; Sacchi, A.; Borgna, L.; Magni, F.; Gasparri, A.; Corti, A. Enhancement of tumor necrosis factor α antitumor immunotherapeutic properties by targeted delivery to aminopeptidase N (CD13). *Nature Biotechnology* **2000**, *18*, 1185-1190, doi:10.1038/81183.

333. Sacchi, A.; Gasparri, A.; Gallo-Stampino, C.; Toma, S.; Curnis, F.; Corti, A. Synergistic antitumor activity of cisplatin, paclitaxel, and gemcitabine with tumor vasculature-targeted tumor necrosis factor-alpha. *Clinical cancer research : an official journal of the American Association for Cancer Research* **2006**, *12*, 175-182, doi:10.1158/1078-0432.ccr-05-1147.

334. Gregorc, V.; De Braud, F.G.; De Pas, T.M.; Scalamogna, R.; Citterio, G.; Milani, A.; Boselli, S.; Catania, C.; Donadoni, G.; Rossoni, G., et al. Phase I Study of NGR-hTNF, a Selective Vascular Targeting Agent, in Combination with Cisplatin in Refractory Solid Tumors. *Clinical Cancer Research* **2011**, 10.1158/1078-0432.CCR-10-1376, doi:10.1158/1078-0432.CCR-10-1376.

335. Pastorino, F.; Brignole, C.; Marimpietri, D.; Cilli, M.; Gambini, C.; Ribatti, D.; Longhi, R.; Allen, T.M.; Corti, A.; Ponzoni, M. Vascular Damage and Anti-angiogenic Effects of Tumor Vessel-Targeted Liposomal Chemotherapy. *Cancer Research* **2003**, *63*, 7400.

336. Pastorino, F.; Brignole, C.; Di Paolo, D.; Nico, B.; Pezzolo, A.; Marimpietri, D.; Pagnan, G.; Piccardi, F.; Cilli, M.; Panzoni, M. Targeting Liposomal Chemotherapy via Both Tumor Cell–Specific and Tumor Vasculature–Specific Ligands Potentiates Therapeutic Efficacy. *Cancer Res* **2006**, *66*, 10073-10082, doi:10.1158/0008-5472.CAN-06-2117.

337. Corti, A.; Ponzoni, M. Tumor Vascular Targeting with Tumor Necrosis Factor α and Chemotherapeutic Drugs. *Annals of the New York Academy of Sciences* **2004**, *1028*, 104-112, doi:10.1196/annals.1322.011.

338. Curnis, F.; Fiocchi, M.; Sacchi, A.; Gori, A.; Gasparri, A.; Corti, A. NGR-tagged nano-gold: A new CD13-selective carrier for cytokine delivery to tumors. *Nano Research* **2016**, *9*, 1393-1408, doi:10.1007/s12274-016-1035-8.

339. Garde, S.V.; Forté, A.J.; Ge, M.; Lepekhin, E.A.; Panchal, C.J.; Rabbani, S.A.; Wu, J.J. Binding and internalization of NGR-peptide-targeted liposomal doxorubicin (TVT-DOX) in CD13-expressing cells and its antitumor effects. *Anti-Cancer Drugs* **2007**, *18*.

340. Zarovni, N.; Monaco, L.; Corti, A. Inhibition of Tumor Growth by Intramuscular Injection of cDNA Encoding Tumor Necrosis Factor α Coupled to NGR and RGD Tumor-Homing Peptides. *Human Gene Therapy* **2004**, *15*, 373-382, doi:10.1089/104303404322959524.

341. Moede, T.; Leibiger, B.; Pour, H.G.; Berggren, P.-O.; Leibiger, I.B. Identification of a nuclear localization signal, RRMKWKK, in the homeodomain transcription factor PDX-1. *FEBS Letters* **1999**, *461*, 229-234, doi:10.1016/S0014-5793(99)01446-5.

342. Rodrigues, M.; Andreu, D.; Santos, N.C. Uptake and cellular distribution of nucleolar targeting peptides (NrTPs) in different cell types. *Peptide Science* **2015**, *104*, 101-109, doi:10.1002/bip.22610.

343. Rodrigues, M.; de la Torre, B.G.; Rádis-Baptista, G.; Santos, N.C.; Andreu, D. Efficient Cellular Delivery of β-Galactosidase Mediated by NrTPs, a New Family of Cell-Penetrating Peptides. *Bioconjugate Chemistry* **2011**, *22*, 2339-2344, doi:10.1021/bc200421z.

344. Sehgal, I.; Sibrian-Vazquez, M.; Vicente, M.G.H. Photoinduced Cytotoxicity and Biodistribution of Prostate Cancer Cell-Targeted Porphyrins. *Journal of Medicinal Chemistry* **2008**, *51*, 6014-6020, doi:10.1021/jm800444c.

345. Lewis, H.D.; Husain, A.; Donnelly, R.J.; Barlos, D.; Riaz, S.; Ginjupalli, K.; Shodeinde, A.; Barton, B.E. Creation of a novel peptide with enhanced nuclear localization in prostate and pancreatic cancer cell lines. *BMC Biotechnology* **2010**, *10*, 79, doi:10.1186/1472-6750-10-79.

346. Lelle, M.; Frick, S.U.; Steinbrink, K.; Peneva, K. Novel cleavable cell-penetrating peptide–drug conjugates: synthesis and characterization. *Journal of Peptide Science* **2014**, *20*, 323-333, doi:10.1002/psc.2617.

347. Daniels, D.S.; Schepartz, A. Intrinsically Cell-Permeable Miniature Proteins Based on a Minimal Cationic PPII Motif. *Journal of the American Chemical Society* **2007**, *129*, 14578-14579, doi:10.1021/ja0772445.

348. Hoffmann, K.; Milech, N.; Juraja, S.M.; Cunningham, P.T.; Stone, S.R.; Francis, R.W.; Anastasas, M.; Hall, C.M.; Heinrich, T.; Bogdawa, H.M., et al. A platform for discovery of functional cell-penetrating peptides for efficient multi-cargo intracellular delivery. *Scientific Reports* **2018**, *8*, 12538, doi:10.1038/s41598-018-30790-2.

349. Śmiłowicz, D.; Metzler-Nolte, N. Bioconjugates of Co(III) complexes with Schiff base ligands and cell penetrating peptides: Solid phase synthesis, characterization and antiproliferative activity. *Journal of Inorganic Biochemistry* **2020**, *206*, 111041, doi:<https://doi.org/10.1016/j.jinorgbio.2020.111041>.

350. Covic, L.; Misra, M.; Badar, J.; Singh, C.; Kuliopulos, A. Pepducin-based intervention of thrombin-receptor signaling and systemic platelet activation. *Nature Medicine* **2002**, *8*, 1161-1165, doi:10.1038/nm760.

351. Jirka, S.M.G.; Heemskerk, H.; Tanganyika-de Winter, C.L.; Muilwijk, D.; Pang, K.H.; de Visser, P.C.; Janson, A.; Karnaoukh, T.G.; Vermue, R.; ‘t Hoen, P.A.C., et al. Peptide Conjugation of 2′-O-methyl Phosphorothioate Antisense Oligonucleotides Enhances Cardiac Uptake and Exon Skipping in mdx Mice. *Nucleic Acid Therapeutics* **2013**, *24*, 25-36, doi:10.1089/nat.2013.0448.

352. Li, L.; Shi, Y.; Cheng, X.; Xia, S.; Cheserek, M.J.; Le, G. A cell-penetrating peptide analogue, P7, exerts antimicrobial activity against Escherichia coli ATCC25922 via penetrating cell membrane and targeting intracellular DNA. *Food Chemistry* **2015**, *166*, 231-239, doi:<https://doi.org/10.1016/j.foodchem.2014.05.113>.

353. Fillon, Y.A.; Anderson, J.P.; Chmielewski, J. Cell Penetrating Agents Based on a Polyproline Helix Scaffold. *Journal of the American Chemical Society* **2005**, *127*, 11798-11803, doi:10.1021/ja052377g.

354. Brezden, A.; Mohamed, M.F.; Nepal, M.; Harwood, J.S.; Kuriakose, J.; Seleem, M.N.; Chmielewski, J. Dual Targeting of Intracellular Pathogenic Bacteria with a Cleavable Conjugate of Kanamycin and an Antibacterial Cell-Penetrating Peptide. *Journal of the American Chemical Society* **2016**, *138*, 10945-10949, doi:10.1021/jacs.6b04831.

355. Zhang, P.; Moreno, R.; Lambert, P.F.; DiMaio, D. Cell-penetrating peptide inhibits retromer-mediated human papillomavirus trafficking during virus entry. *Proceedings of the National Academy of Sciences* **2020**, *117*, 6121, doi:10.1073/pnas.1917748117.

356. Taylor, B.N.; Mehta, R.R.; Yamada, T.; Lekmine, F.; Christov, K.; Chakrabarty, A.M.; Green, A.; Bratescu, L.; Shilkaitis, A.; Beattie, C.W., et al. Noncationic Peptides Obtained From Azurin Preferentially Enter Cancer Cells. *Cancer Research* **2009**, *69*, 537, doi:10.1158/0008-5472.CAN-08-2932.

357. Warso, M.A.; Richards, J.M.; Mehta, D.; Christov, K.; Schaeffer, C.; Rae Bressler, L.; Yamada, T.; Majumdar, D.; Kennedy, S.A.; Beattie, C.W., et al. A first-in-class, first-in-human, phase I trial of p28, a non-HDM2-mediated peptide inhibitor of p53 ubiquitination in patients with advanced solid tumours. *British Journal of Cancer* **2013**, *108*, 1061-1070, doi:10.1038/bjc.2013.74.

358. Yamada, T.; Christov, K.; Shilkaitis, A.; Bratescu, L.; Green, A.; Santini, S.; Bizzarri, A.R.; Cannistraro, S.; Gupta, T.K.D.; Beattie, C.W. p28, A first in class peptide inhibitor of cop1 binding to p53. *British Journal of Cancer* **2013**, *108*, 2495-2504, doi:10.1038/bjc.2013.266.

359. Mehta, R.R.; Yamada, T.; Taylor, B.N.; Christov, K.; King, M.L.; Majumdar, D.; Lekmine, F.; Tiruppathi, C.; Shilkaitis, A.; Bratescu, L., et al. A cell penetrating peptide derived from azurin inhibits angiogenesis and tumor growth by inhibiting phosphorylation of VEGFR-2, FAK and Akt. *Angiogenesis* **2011**, *14*, 355-369, doi:10.1007/s10456-011-9220-6.

360. Yamada, T.; Das Gupta, T.K.; Beattie, C.W. p28, an Anionic Cell-Penetrating Peptide, Increases the Activity of Wild Type and Mutated p53 without Altering Its Conformation. *Molecular Pharmaceutics* **2013**, *10*, 3375-3383, doi:10.1021/mp400221r.

361. Yamada, T.; Signorelli, S.; Cannistraro, S.; Beattie, C.W.; Bizzarri, A.R. Chirality Switching within an Anionic Cell-Penetrating Peptide Inhibits Translocation without Affecting Preferential Entry. *Molecular Pharmaceutics* **2015**, *12*, 140-149, doi:10.1021/mp500495u.

362. Lulla, R.R.; Goldman, S.; Yamada, T.; Beattie, C.W.; Bressler, L.; Pacini, M.; Pollack, I.F.; Fisher, P.G.; Packer, R.J.; Dunkel, I.J., et al. Phase I trial of p28 (NSC745104), a non-HDM2-mediated peptide inhibitor of p53 ubiquitination in pediatric patients with recurrent or progressive central nervous system tumors: A Pediatric Brain Tumor Consortium Study. *Neuro-Oncology* **2016**, *18*, 1319-1325, doi:10.1093/neuonc/now047.

363. Sardan, M.; Kilinc, M.; Genc, R.; Tekinay, A.B.; Guler, M.O. Cell penetrating peptide amphiphile integrated liposomal systems for enhanced delivery of anticancer drugs to tumor cells. *Faraday Discussions* **2013**, *166*, 269-283, doi:10.1039/C3FD00058C.

364. Bundó, M.; Shi, X.; Vernet, M.; Marcos, J.F.; López-García, B.; Coca, M. Rice Seeds as Biofactories of Rationally Designed and Cell-Penetrating Antifungal PAF Peptides. *Frontiers in Plant Science* **2019**, *10*, 731.

365. Wang, X.; Huang, H.; Zhang, L.; Bai, Y.; Chen, H. PCM and TAT co-modified liposome with improved myocardium delivery: in vitro and in vivo evaluations. *Drug Delivery* **2017**, *24*, 339-345, doi:10.1080/10717544.2016.1253121.

366. Noguchi, H.; Matsushita, M.; Matsumoto, S.; Lu, Y.-F.; Matsui, H.; Bonner-Weir, S. Mechanism of PDX-1 protein transduction. *Biochemical and biophysical research communications* **2005**, *332*, 68-74, doi:10.1016/j.bbrc.2005.04.092.

367. Kühnel, F.; Schulte, B.; Wirth, T.; Woller, N.; Schäfers, S.; Zender, L.; Manns, M.; Kubicka, S. Protein Transduction Domains Fused to Virus Receptors Improve Cellular Virus Uptake and Enhance Oncolysis by Tumor-Specific Replicating Vectors. *Journal of Virology* **2004**, *78*, 13743, doi:10.1128/JVI.78.24.13743-13754.2004.

368. Deshayes, S.; Decaffmeyer, M.; Brasseur, R.; Thomas, A. Structural polymorphism of two CPP: an important parameter of activity. *Biochimica et biophysica acta* **2008**, *1778*, 1197-1205, doi:10.1016/j.bbamem.2008.01.027.

369. Letoha, T.; Keller-Pintér, A.; Kusz, E.; Kolozsi, C.; Bozsó, Z.; Tóth, G.; Vizler, C.; Oláh, Z.; Szilák, L. Cell-penetrating peptide exploited syndecans. *Biochimica et Biophysica Acta (BBA) - Biomembranes* **2010**, *1798*, 2258-2265, doi:<https://doi.org/10.1016/j.bbamem.2010.01.022>.

370. Alves, I.D.; Carré, M.; Montero, M.-P.; Castano, S.; Lecomte, S.; Marquant, R.; Lecorché, P.; Burlina, F.; Schatz, C.; Sagan, S., et al. A proapoptotic peptide conjugated to penetratin selectively inhibits tumor cell growth. *Biochimica et biophysica acta* **2014**, *1838*, 2087-2098, doi:10.1016/j.bbamem.2014.04.025.

371. Khafagy, E.-S.; Morishita, M.; Isowa, K.; Imai, J.; Takayama, K. Effect of cell-penetrating peptides on the nasal absorption of insulin. *Journal of controlled release : official journal of the Controlled Release Society* **2009**, *133*, 103-108, doi:10.1016/j.jconrel.2008.09.076.

372. Nakase, I.; Konishi, Y.; Ueda, M.; Saji, H.; Futaki, S. Accumulation of arginine-rich cell-penetrating peptides in tumors and the potential for anticancer drug delivery in vivo. *Journal of controlled release : official journal of the Controlled Release Society* **2012**, *159*, 181-188, doi:10.1016/j.jconrel.2012.01.016.

373. Suda, K.; Murakami, T.; Gotoh, N.; Fukuda, R.; Hashida, Y.; Hashida, M.; Tsujikawa, A.; Yoshimura, N. High-density lipoprotein mutant eye drops for the treatment of posterior eye diseases. *Journal of controlled release : official journal of the Controlled Release Society* **2017**, *266*, 301-309, doi:10.1016/j.jconrel.2017.09.036.

374. Jiang, K.; Gao, X.; Shen, Q.; Zhan, C.; Zhang, Y.; Xie, C.; Wei, G.; Lu, W. Discerning the composition of penetratin for safe penetration from cornea to retina. *Acta biomaterialia* **2017**, *63*, 123-134, doi:10.1016/j.actbio.2017.09.023.

375. Magzoub, M.; Eriksson, L.E.G.; Gräslund, A. Comparison of the interaction, positioning, structure induction and membrane perturbation of cell-penetrating peptides and non-translocating variants with phospholipid vesicles. *Biophysical Chemistry* **2003**, *103*, 271-288, doi:<https://doi.org/10.1016/S0301-4622(02)00321-6>.

376. Khafagy, E.-S.; Morishita, M.; Kamei, N.; Eda, Y.; Ikeno, Y.; Takayama, K. Efficiency of cell-penetrating peptides on the nasal and intestinal absorption of therapeutic peptides and proteins. *International journal of pharmaceutics* **2009**, *381*, 49-55, doi:10.1016/j.ijpharm.2009.07.022.

377. Tai, L.; Liu, C.; Jiang, K.; Chen, X.; Feng, L.; Pan, W.; Wei, G.; Lu, W. A novel penetratin-modified complex for noninvasive intraocular delivery of antisense oligonucleotides. *International Journal of Pharmaceutics* **2017**, *529*, 347-356, doi:<https://doi.org/10.1016/j.ijpharm.2017.06.090>.

378. Derossi, D.; Chassaing, G.; Prochiantz, A. Trojan peptides: the penetratin system for intracellular delivery. *Trends in Cell Biology* **1998**, *8*, 84-87, doi:10.1016/S0962-8924(98)80017-2.

379. Peterson, J.M.; Kline, W.; Canan, B.D.; Ricca, D.J.; Kaspar, B.; Delfín, D.A.; DiRienzo, K.; Clemens, P.R.; Robbins, P.D.; Baldwin, A.S., et al. Peptide-Based Inhibition of NF-κB Rescues Diaphragm Muscle Contractile Dysfunction in a Murine Model of Duchenne Muscular Dystrophy. *Molecular Medicine* **2011**, *17*, 508-515, doi:10.2119/molmed.2010.00263.

380. Kamei, N.; Shigei, C.; Hasegawa, R.; Takeda-Morishita, M. Exploration of the Key Factors for Optimizing the <i>in Vivo</i> Oral Delivery of Insulin by Using a Noncovalent Strategy with Cell-Penetrating Peptides. *Biological and Pharmaceutical Bulletin* **2018**, *41*, 239-246, doi:10.1248/bpb.b17-00798.

381. Joliot, A.; Pernelle, C.; Deagostini-Bazin, H.; Prochiantz, A. Antennapedia homeobox peptide regulates neural morphogenesis. *Proceedings of the National Academy of Sciences of the United States of America* **1991**, *88*, 1864-1868, doi:10.1073/pnas.88.5.1864.

382. Jain, M.; Chauhan, S.C.; Singh, A.P.; Venkatraman, G.; Colcher, D.; Batra, S.K. Penetratin Improves Tumor Retention of Single-Chain Antibodies: A Novel Step toward Optimization of Radioimmunotherapy of Solid Tumors. *Cancer Research* **2005**, *65*, 7840, doi:10.1158/0008-5472.CAN-05-0662.

383. Kristensen, M.; Franzyk, H.; Klausen, M.T.; Iversen, A.; Bahnsen, J.S.; Skyggebjerg, R.B.; Foderà, V.; Nielsen, H.M. Penetratin-Mediated Transepithelial Insulin Permeation: Importance of Cationic Residues and pH for Complexation and Permeation. *The AAPS Journal* **2015**, *17*, 1200-1209, doi:10.1208/s12248-015-9747-3.

384. Muratovska, A.; Eccles, M.R. Conjugate for efficient delivery of short interfering RNA (siRNA) into mammalian cells. *FEBS Letters* **2004**, *558*, 63-68, doi:10.1016/S0014-5793(03)01505-9.

385. de Mello, L.R.; Hamley, I.W.; Castelletto, V.; Garcia, B.B.M.; Han, S.W.; de Oliveira, C.L.P.; da Silva, E.R. Nanoscopic Structure of Complexes Formed between DNA and the Cell-Penetrating Peptide Penetratin. *The Journal of Physical Chemistry B* **2019**, *123*, 8861-8871, doi:10.1021/acs.jpcb.9b05512.

386. Daimon, Y.; Kamei, N.; Kawakami, K.; Takeda-Morishita, M.; Izawa, H.; Takechi-Haraya, Y.; Saito, H.; Sakai, H.; Abe, M.; Ariga, K. Dependence of Intestinal Absorption Profile of Insulin on Carrier Morphology Composed of β-Cyclodextrin-Grafted Chitosan. *Molecular Pharmaceutics* **2016**, *13*, 4034-4042, doi:10.1021/acs.molpharmaceut.6b00561.

387. Som, A.; Reuter, A.; Tew, G.N. Protein Transduction Domain Mimics: The Role of Aromatic Functionality. *Angewandte Chemie International Edition* **2012**, *51*, 980-983, doi:10.1002/anie.201104624.

388. Moulton, H.M.; Nelson, M.H.; Hatlevig, S.A.; Reddy, M.T.; Iversen, P.L. Cellular Uptake of Antisense Morpholino Oligomers Conjugated to Arginine-Rich Peptides. *Bioconjugate Chemistry* **2004**, *15*, 290-299, doi:10.1021/bc034221g.

389. Bechara, C.; Pallerla, M.; Burlina, F.; Illien, F.; Cribier, S.; Sagan, S. Massive glycosaminoglycan-dependent entry of Trp-containing cell-penetrating peptides induced by exogenous sphingomyelinase or cholesterol depletion. *Cellular and Molecular Life Sciences* **2015**, *72*, 809-820, doi:10.1007/s00018-014-1696-y.

390. Ferreira, A.; Lapa, R.; Vale, N. Combination of Gemcitabine with Cell-Penetrating Peptides: A Pharmacokinetic Approach Using in Silico Tools. *Biomolecules* **2019**, *9*, doi:10.3390/biom9110693.

391. Eggimann, G.A.; Buschor, S.; Darbre, T.; Reymond, J.-L. Convergent synthesis and cellular uptake of multivalent cell penetrating peptides derived from Tat, Antp, pVEC, TP10 and SAP. *Organic & Biomolecular Chemistry* **2013**, *11*, 6717-6733, doi:10.1039/C3OB41023D.

392. Child, H.W.; del Pino, P.A.; De La Fuente, J.M.; Hursthouse, A.S.; Stirling, D.; Mullen, M.; McPhee, G.M.; Nixon, C.; Jayawarna, V.; Berry, C.C. Working Together: The Combined Application of a Magnetic Field and Penetratin for the Delivery of Magnetic Nanoparticles to Cells in 3D. *ACS Nano* **2011**, *5*, 7910-7919, doi:10.1021/nn202163v.

393. Dutot, L.; Lécorché, P.; Burlina, F.; Marquant, R.; Point, V.; Sagan, S.; Chassaing, G.; Mallet, J.-M.; Lavielle, S. Glycosylated cell-penetrating peptides and their conjugates to a proapoptotic peptide: preparation by click chemistry and cell viability studies. *Journal of chemical biology* **2009**, *3*, 51-65, doi:10.1007/s12154-009-0031-9.

394. Elmquist, A.; Lindgren, M.; Bartfai, T.; Langel, Ü. VE-Cadherin-Derived Cell-Penetrating Peptide, pVEC, with Carrier Functions. *Experimental Cell Research* **2001**, *269*, 237-244, doi:<https://doi.org/10.1006/excr.2001.5316>.

395. Zhu, S.; Chen, S.; Gao, Y.; Guo, F.; Li, F.; Xie, B.; Zhou, J.; Zhong, H. Enhanced oral bioavailability of insulin using PLGA nanoparticles co-modified with cell-penetrating peptides and Engrailed secretion peptide (Sec). *Drug Delivery* **2016**, *23*, 1980-1991, doi:10.3109/10717544.2015.1043472.

396. Jobin, M.-L.; Vamparys, L.; Deniau, R.; Grélard, A.; Mackereth, D.C.; Fuchs, F.J.P.; Alves, D.I. Biophysical Insight on the Membrane Insertion of an Arginine-Rich Cell-Penetrating Peptide. *International Journal of Molecular Sciences* **2019**, *20*, doi:10.3390/ijms20184441.

397. Alsulays, B.B.; Anwer, M.K.; Soliman, G.A.; Alshehri, S.M.; Khafagy, E.-S. Impact Of Penetratin Stereochemistry On The Oral Bioavailability Of Insulin-Loaded Solid Lipid Nanoparticles. *International journal of nanomedicine* **2019**, *14*, 9127-9138, doi:10.2147/IJN.S225086.

398. Derossi, D.; Joliot, A.H.; Chassaing, G.; Prochiantz, A. The third helix of the Antennapedia homeodomain translocates through biological membranes. *The Journal of biological chemistry* **1994**, *269*, 10444-10450.

399. Fischer, R.; Köhler, K.; Fotin-Mleczek, M.; Brock, R. A Stepwise Dissection of the Intracellular Fate of Cationic Cell-penetrating Peptides. *Journal of Biological Chemistry* **2004**, *279*, 12625-12635.

400. Duchardt, F.; Fotin-Mleczek, M.; Schwarz, H.; Fischer, R.; Brock, R. A Comprehensive Model for the Cellular Uptake of Cationic Cell-penetrating Peptides. *Traffic* **2007**, *8*, 848-866, doi:10.1111/j.1600-0854.2007.00572.x.

401. Kamei, N.; Kikuchi, S.; Takeda-Morishita, M.; Terasawa, Y.; Yasuda, A.; Yamamoto, S.; Ida, N.; Nishio, R.; Takayama, K. Determination of the optimal cell-penetrating peptide sequence for intestinal insulin delivery based on molecular orbital analysis with self-organizing maps. *Journal of Pharmaceutical Sciences* **2013**, *102*, 469-479, doi:10.1002/jps.23364.

402. Khafagy, E.-S.; Morishita, M.; Ida, N.; Nishio, R.; Isowa, K.; Takayama, K. Structural requirements of penetratin absorption enhancement efficiency for insulin delivery. *Journal of controlled release : official journal of the Controlled Release Society* **2010**, *143*, 302-310, doi:10.1016/j.jconrel.2010.01.019.

403. Kilk, K.; Magzoub, M.; Pooga, M.; Eriksson, L.E.G.; Langel, Ü.; Gräslund, A. Cellular Internalization of a Cargo Complex with a Novel Peptide Derived from the Third Helix of the Islet-1 Homeodomain. Comparison with the Penetratin Peptide. *Bioconjugate Chemistry* **2001**, *12*, 911-916, doi:10.1021/bc0100298.

404. Kim, D.; Lee, Y.; Dreher, T.W.; Cho, T.-J. Empty Turnip yellow mosaic virus capsids as delivery vehicles to mammalian cells. *Virus research* **2018**, *252*, 13-21, doi:10.1016/j.virusres.2018.05.004.

405. Kristensen, M.; de Groot, A.M.; Berthelsen, J.; Franzyk, H.; Sijts, A.; Nielsen, H.M. Conjugation of Cell-Penetrating Peptides to Parathyroid Hormone Affects Its Structure, Potency, and Transepithelial Permeation. *Bioconjugate Chemistry* **2015**, *26*, 477-488, doi:10.1021/bc5005763.

406. Liu, C.; Jiang, K.; Tai, L.; Liu, Y.; Wei, G.; Lu, W.; Pan, W. Facile Noninvasive Retinal Gene Delivery Enabled by Penetratin. *ACS Applied Materials & Interfaces* **2016**, *8*, 19256-19267, doi:10.1021/acsami.6b04551.

407. Algayer, B.; O’Brien, A.; Momose, A.; Murphy, J.D.; Procopio, W.; Tellers, M.D.; Tucker, J.T. Novel pH Selective, Highly Lytic Peptides Based on a Chimeric Influenza Hemagglutinin Peptide/Cell Penetrating Peptide Motif. *Molecules* **2019**, *24*, doi:10.3390/molecules24112079.

408. Zhu, X.; Shan, W.; Zhang, P.; Jin, Y.; Guan, S.; Fan, T.; Yang, Y.; Zhou, Z.; Huang, Y. Penetratin Derivative-Based Nanocomplexes for Enhanced Intestinal Insulin Delivery. *Molecular Pharmaceutics* **2014**, *11*, 317-328, doi:10.1021/mp400493b.

409. Liu, C.; Tai, L.; Zhang, W.; Wei, G.; Pan, W.; Lu, W. Penetratin, a Potentially Powerful Absorption Enhancer for Noninvasive Intraocular Drug Delivery. *Molecular Pharmaceutics* **2014**, *11*, 1218-1227, doi:10.1021/mp400681n.

410. Nielsen, E.J.B.; Yoshida, S.; Kamei, N.; Iwamae, R.; Khafagy, E.-S.; Olsen, J.; Rahbek, U.L.; Pedersen, B.L.; Takayama, K.; Takeda-Morishita, M. In vivo proof of concept of oral insulin delivery based on a co-administration strategy with the cell-penetrating peptide penetratin. *Journal of Controlled Release* **2014**, *189*, 19-24, doi:<https://doi.org/10.1016/j.jconrel.2014.06.022>.

411. Nigatu, A.S.; Vupputuri, S.; Flynn, N.; Ramsey, J.D. Effects of cell-penetrating peptides on transduction efficiency of PEGylated adenovirus. *Biomedicine & pharmacotherapy = Biomedecine & pharmacotherapie* **2015**, *71*, 153-160, doi:10.1016/j.biopha.2015.02.015.

412. Gao, S.; Simon, M.J.; Hue, C.D.; Morrison, B., 3rd; Banta, S. An unusual cell penetrating peptide identified using a plasmid display-based functional selection platform. *ACS chemical biology* **2011**, *6*, 484-491, doi:10.1021/cb100423u.

413. Böhmová, E.; Pola, R.; Pechar, M.; Parnica, J.; Machová, D.; Janoušková, O.; Etrych, T. Polymer Cancerostatics Containing Cell-Penetrating Peptides: Internalization Efficacy Depends on Peptide Type and Spacer Length. *Pharmaceutics* **2020**, *12*, doi:10.3390/pharmaceutics12010059.

414. Mi, Z.; Mai, J.; Lu, X.; Robbins, P.D. Characterization of a Class of Cationic Peptides Able to Facilitate Efficient Protein Transduction in Vitro and in Vivo. *Molecular Therapy* **2000**, *2*, 339-347, doi:<https://doi.org/10.1006/mthe.2000.0137>.

415. Shan, W.; Zhu, X.; Liu, M.; Li, L.; Zhong, J.; Sun, W.; Zhang, Z.; Huang, Y. Overcoming the Diffusion Barrier of Mucus and Absorption Barrier of Epithelium by Self-Assembled Nanoparticles for Oral Delivery of Insulin. *ACS Nano* **2015**, *9*, 2345-2356, doi:10.1021/acsnano.5b00028.

416. Guo, F.; Ouyang, T.; Peng, T.; Zhang, X.; Xie, B.; Yang, X.; Liang, D.; Zhong, H. Enhanced oral absorption of insulin using colon-specific nanoparticles co-modified with amphiphilic chitosan derivatives and cell-penetrating peptides. *Biomaterials Science* **2019**, *7*, 1493-1506, doi:10.1039/C8BM01485J.

417. Tan, X.; Zhang, Y.; Wang, Q.; Ren, T.; Gou, J.; Guo, W.; Yin, T.; He, H.; Zhang, Y.; Tang, X. Cell-penetrating peptide together with PEG-modified mesostructured silica nanoparticles promotes mucous permeation and oral delivery of therapeutic proteins and peptides. *Biomaterials Science* **2019**, *7*, 2934-2950, doi:10.1039/C9BM00274J.

418. Takeuchi, T.; Kosuge, M.; Tadokoro, A.; Sugiura, Y.; Nishi, M.; Kawata, M.; Sakai, N.; Matile, S.; Futaki, S. Direct and Rapid Cytosolic Delivery Using Cell-Penetrating Peptides Mediated by Pyrenebutyrate. *ACS Chemical Biology* **2006**, *1*, 299-303, doi:10.1021/cb600127m.

419. Prezma, T.; Shteinfer, A.; Admoni, L.; Raviv, Z.; Sela, I.; Levi, I.; Shoshan-Barmatz, V. VDAC1-based peptides: novel pro-apoptotic agents and potential therapeutics for B-cell chronic lymphocytic leukemia. *Cell Death & Disease* **2013**, *4*, e809-e809, doi:10.1038/cddis.2013.316.

420. George, E.M.; Mahdi, F.; Logue, O.C.; Robinson, G.G.; Bidwell, G.L. Corneal Penetrating Elastin-Like Polypeptide Carriers. *Journal of Ocular Pharmacology and Therapeutics* **2015**, *32*, 163-171, doi:10.1089/jop.2015.0082.

421. Pooga, M.; Soomets, U.; Hällbrink, M.; Valkna, A.; Saar, K.; Rezaei, K.; Kahl, U.; Hao, J.-X.; Xu, X.-J.; Wiesenfeld-Hallin, Z., et al. Cell penetrating PNA constructs regulate galanin receptor levels and modify pain transmission in vivo. *Nature Biotechnology* **1998**, *16*, 857-861, doi:10.1038/nbt0998-857.

422. Gratton, J.-P.; Yu, J.; Griffith, J.W.; Babbitt, R.W.; Scotland, R.S.; Hickey, R.; Giordano, F.J.; Sessa, W.C. Cell-permeable peptides improve cellular uptake and therapeutic gene delivery of replication-deficient viruses in cells and in vivo. *Nature Medicine* **2003**, *9*, 357-362, doi:10.1038/nm835.

423. Han, K.; Jeon, M.-J.; Kim, K.-A.; Park, J.; Choi, S.Y. Efficient Intracellular Delivery of GFP by Homeodomains of Drosophila Fushi-tarazu and Engrailed Proteins. *Molecules and Cells* **2000**, *10*, 728-732, doi:10.1007/s10059-000-0728-7.

424. Jean, S.R.; Ahmed, M.; Lei, E.K.; Wisnovsky, S.P.; Kelley, S.O. Peptide-Mediated Delivery of Chemical Probes and Therapeutics to Mitochondria. *Accounts of Chemical Research* **2016**, *49*, 1893-1902, doi:10.1021/acs.accounts.6b00277.

425. Lein, M.; deRonde, B.M.; Sgolastra, F.; Tew, G.N.; Holden, M.A. Protein transport across membranes: Comparison between lysine and guanidinium-rich carriers. *Biochimica et Biophysica Acta (BBA) - Biomembranes* **2015**, *1848*, 2980-2984, doi:<https://doi.org/10.1016/j.bbamem.2015.09.004>.

426. Almarwani, B.; Phambu, E.N.; Alexander, C.; Nguyen, H.A.T.; Phambu, N.; Sunda-Meya, A. Vesicles mimicking normal and cancer cell membranes exhibit differential responses to the cell-penetrating peptide Pep-1. *Biochimica et Biophysica Acta (BBA) - Biomembranes* **2018**, *1860*, 1394-1402, doi:<https://doi.org/10.1016/j.bbamem.2018.03.022>.

427. Huang, G.-Q.; Wang, J.-N.; Tang, J.-M.; Zhang, L.; Zheng, F.; Yang, J.-Y.; Guo, L.-Y.; Kong, X.; Huang, Y.-Z.; Liu, Y., et al. The combined transduction of copper, zinc-superoxide dismutase and catalase mediated by cell-penetrating peptide, PEP-1, to protect myocardium from ischemia-reperfusion injury. *Journal of translational medicine* **2011**, *9*, 73-73, doi:10.1186/1479-5876-9-73.

428. Pescina, S.; Sala, M.; Padula, C.; Scala, M.C.; Spensiero, A.; Belletti, S.; Gatti, R.; Novellino, E.; Campiglia, P.; Santi, P., et al. Design and Synthesis of New Cell Penetrating Peptides: Diffusion and Distribution Inside the Cornea. *Molecular Pharmaceutics* **2016**, *13*, 3876-3883, doi:10.1021/acs.molpharmaceut.6b00658.

429. Deshayes, S.; Heitz, A.; Morris, M.C.; Charnet, P.; Divita, G.; Heitz, F. Insight into the Mechanism of Internalization of the Cell-Penetrating Carrier Peptide Pep-1 through Conformational Analysis. *Biochemistry* **2004**, *43*, 1449-1457, doi:10.1021/bi035682s.

430. Di Pisa, M.; Chassaing, G.; Swiecicki, J.-M. Translocation Mechanism(s) of Cell-Penetrating Peptides: Biophysical Studies Using Artificial Membrane Bilayers. *Biochemistry* **2015**, *54*, 194-207, doi:10.1021/bi501392n.

431. Kim, D.W.; Lee, S.H.; Shin, M.J.; Kim, K.; Ku, S.K.; Youn, J.K.; Cho, S.B.; Park, J.H.; Lee, C.H.; Son, O., et al. PEP-1-FK506BP inhibits alkali burn-induced corneal inflammation on the rat model of corneal alkali injury. *BMB reports* **2015**, *48*, 618-623, doi:10.5483/BMBRep.2015.48.11.041.

432. Kim, M.J.; Park, M.; Kim, D.W.; Shin, M.J.; Son, O.; Jo, H.S.; Yeo, H.J.; Cho, S.B.; Park, J.H.; Lee, C.H., et al. Transduced PEP-1-PON1 proteins regulate microglial activation and dopaminergic neuronal death in a Parkinson's disease model. *Biomaterials* **2015**, *64*, 45-56, doi:<https://doi.org/10.1016/j.biomaterials.2015.06.015>.

433. Morris, M.C.; Depollier, J.; Mery, J.; Heitz, F.; Divita, G. A peptide carrier for the delivery of biologically active proteins into mammalian cells. *Nature Biotechnology* **2001**, *19*, 1173-1176, doi:10.1038/nbt1201-1173.

434. Sun, Y.; Li, J.; Sun, Y.; Zhao, R.; Wang, L.; Song, W.; Wang, Z.; Wang, J.; Wei, L.; Zhao, Y., et al. A Stable Pep2-proapoptotic Peptide Inducing Apoptosis of Acute Myeloid Leukemia Cells by Down-Regulating EZH2. In *Cellular and molecular bioengineering*, 2020; Vol. 13, pp 165-177.

435. Morris, M.C.; Gros, E.; Aldrian-Herrada, G.; Choob, M.; Archdeacon, J.; Heitz, F.; Divita, G. A non-covalent peptide-based carrier for in vivo delivery of DNA mimics. *Nucleic Acids Research* **2007**, *35*, e49-e49, doi:10.1093/nar/gkm053.

436. Morris, M.C.; Chaloin, L.; Choob, M.; Archdeacon, J.; Heitz, F.; Divita, G. Combination of a new generation of PNAs with a peptide-based carrier enables efficient targeting of cell cycle progression. *Gene Therapy* **2004**, *11*, 757-764, doi:10.1038/sj.gt.3302235.

437. Gao, C.; Mao, S.; Ditzel, H.J.; Farnaes, L.; Wirsching, P.; Lerner, R.A.; Janda, K.D. A cell-penetrating peptide from a novel pVII–pIX phage-displayed random peptide library. *Bioorganic & Medicinal Chemistry* **2002**, *10*, 4057-4065, doi:<https://doi.org/10.1016/S0968-0896(02)00340-1>.

438. Freire, J.M.; Veiga, A.S.; Rego de Figueiredo, I.; de la Torre, B.G.; Santos, N.C.; Andreu, D.; Da Poian, A.T.; Castanho, M.A.R.B. Nucleic acid delivery by cell penetrating peptides derived from dengue virus capsid protein: design and mechanism of action. *The FEBS Journal* **2014**, *281*, 191-215, doi:10.1111/febs.12587.

439. Freire, J.M.; Almeida Dias, S.; Flores, L.; Veiga, A.S.; Castanho, M.A.R.B. Mining viral proteins for antimicrobial and cell-penetrating drug delivery peptides. *Bioinformatics* **2015**, *31*, 2252-2256, doi:10.1093/bioinformatics/btv131.

440. Mnif, S.; Jardak, M.; Graiet, I.; Abid, S.; Driss, D.; Kharrat, N. The novel cationic cell-penetrating peptide PEP-NJSM is highly active against Staphylococcus epidermidis biofilm. *International Journal of Biological Macromolecules* **2019**, *125*, 262-269, doi:<https://doi.org/10.1016/j.ijbiomac.2018.12.008>.

441. Veiman, K.-L.; Mäger, I.; Ezzat, K.; Margus, H.; Lehto, T.; Langel, K.; Kurrikoff, K.; Arukuusk, P.; Suhorutšenko, J.; Padari, K., et al. PepFect14 Peptide Vector for Efficient Gene Delivery in Cell Cultures. *Molecular Pharmaceutics* **2013**, *10*, 199-210, doi:10.1021/mp3003557.

442. Veiman, K.-L.; Künnapuu, K.; Lehto, T.; Kiisholts, K.; Pärn, K.; Langel, Ü.; Kurrikoff, K. PEG shielded MMP sensitive CPPs for efficient and tumor specific gene delivery in vivo. *Journal of Controlled Release* **2015**, *209*, 238-247, doi:<https://doi.org/10.1016/j.jconrel.2015.04.038>.

443. Ezzat, K.; El Andaloussi, S.; Zaghloul, E.M.; Lehto, T.; Lindberg, S.; Moreno, P.M.D.; Viola, J.R.; Magdy, T.; Abdo, R.; Guterstam, P., et al. PepFect 14, a novel cell-penetrating peptide for oligonucleotide delivery in solution and as solid formulation. *Nucleic Acids Research* **2011**, *39*, 5284-5298, doi:10.1093/nar/gkr072.

444. Srimanee, A.; Arvanitidou, M.; Kim, K.; Hällbrink, M.; Langel, Ü. Cell-penetrating peptides for siRNA delivery to glioblastomas. *Peptides* **2018**, *104*, 62-69, doi:<https://doi.org/10.1016/j.peptides.2018.04.015>.

445. Javanmard, Z.; Kalani, B.S.; Razavi, S.; Farahani, N.N.; Mohammadzadeh, R.; Javanmard, F.; Irajian, G. Evaluation of cell-penetrating peptide–peptide nucleic acid effect in the inhibition of cagA in Helicobacter pylori. *Acta Microbiologica et Immunologica Hungarica* **2020**, 10.1556/030.66.2019.032, 1-7, doi:10.1556/030.66.2019.032.

446. Zhao, H.; Jiang, Y.; Tian, Y.; Yang, D.; Qin, X.; Li, Z. Improving cell penetration of helical peptides stabilized by N-terminal crosslinked aspartic acids. *Organic & Biomolecular Chemistry* **2017**, *15*, 459-464, doi:10.1039/C6OB02501C.

447. Yamashita, H.; Kato, T.; Oba, M.; Misawa, T.; Hattori, T.; Ohoka, N.; Tanaka, M.; Naito, M.; Kurihara, M.; Demizu, Y. Development of a Cell-penetrating Peptide that Exhibits Responsive Changes in its Secondary Structure in the Cellular Environment. *Scientific Reports* **2016**, *6*, 33003, doi:10.1038/srep33003.

448. Liu, X.Y.; Timmons, S.; Lin, Y.Z.; Hawiger, J. Identification of a functionally important sequence in the cytoplasmic tail of integrin beta 3 by using cell-permeable peptide analogs. *Proceedings of the National Academy of Sciences of the United States of America* **1996**, *93*, 11819-11824, doi:10.1073/pnas.93.21.11819.

449. Gan, B.K.; Yong, C.Y.; Ho, K.L.; Omar, A.R.; Alitheen, N.B.; Tan, W.S. Targeted Delivery of Cell Penetrating Peptide Virus-like Nanoparticles to Skin Cancer Cells. *Scientific Reports* **2018**, *8*, 8499, doi:10.1038/s41598-018-26749-y.

450. Cai, D.; Gao, W.; He, B.; Dai, W.; Zhang, H.; Wang, X.; Wang, J.; Zhang, X.; Zhang, Q. Hydrophobic penetrating peptide PFVYLI-modified stealth liposomes for doxorubicin delivery in breast cancer therapy. *Biomaterials* **2014**, *35*, 2283-2294, doi:10.1016/j.biomaterials.2013.11.088.

451. Godfrey, C.; Muses, S.; McClorey, G.; Wells, K.E.; Coursindel, T.; Terry, R.L.; Betts, C.; Hammond, S.; O'Donovan, L.; Hildyard, J., et al. How much dystrophin is enough: the physiological consequences of different levels of dystrophin in the mdx mouse. *Human Molecular Genetics* **2015**, *24*, 4225-4237, doi:10.1093/hmg/ddv155.

452. Boisguerin, P.; Redt-Clouet, C.; Franck-Miclo, A.; Licheheb, S.; Nargeot, J.; Barrère-Lemaire, S.; Lebleu, B. Systemic delivery of BH4 anti-apoptotic peptide using CPPs prevents cardiac ischemia–reperfusion injuries in vivo. *Journal of Controlled Release* **2011**, *156*, 146-153, doi:<https://doi.org/10.1016/j.jconrel.2011.07.037>.

453. Yin, H.; Saleh, A.F.; Betts, C.; Camelliti, P.; Seow, Y.; Ashraf, S.; Arzumanov, A.; Hammond, S.; Merritt, T.; Gait, M.J., et al. Pip5 transduction peptides direct high efficiency oligonucleotide-mediated dystrophin exon skipping in heart and phenotypic correction in mdx mice. *Molecular therapy : the journal of the American Society of Gene Therapy* **2011**, *19*, 1295-1303, doi:10.1038/mt.2011.79.

454. Betts, C.; Saleh, A.F.; Arzumanov, A.A.; Hammond, S.M.; Godfrey, C.; Coursindel, T.; Gait, M.J.; Wood, M.J.A. Pip6-PMO, A New Generation of Peptide-oligonucleotide Conjugates With Improved Cardiac Exon Skipping Activity for DMD Treatment. *Molecular Therapy - Nucleic Acids* **2012**, *1*, doi:10.1038/mtna.2012.30.

455. Lehto, T.; Castillo Alvarez, A.; Gauck, S.; Gait, M.J.; Coursindel, T.; Wood, M.J.A.; Lebleu, B.; Boisguerin, P. Cellular trafficking determines the exon skipping activity of Pip6a-PMO in mdx skeletal and cardiac muscle cells. *Nucleic Acids Research* **2013**, *42*, 3207-3217, doi:10.1093/nar/gkt1220.

456. Miyatake, S.; Mizobe, Y.; Tsoumpra, M.K.; Lim, K.R.Q.; Hara, Y.; Shabanpoor, F.; Yokota, T.; Takeda, S.i.; Aoki, Y. Scavenger Receptor Class A1 Mediates Uptake of Morpholino Antisense Oligonucleotide into Dystrophic Skeletal Muscle. *Molecular therapy. Nucleic acids* **2019**, *14*, 520-535, doi:10.1016/j.omtn.2019.01.008.

457. Hammond, S.M.; Hazell, G.; Shabanpoor, F.; Saleh, A.F.; Bowerman, M.; Sleigh, J.N.; Meijboom, K.E.; Zhou, H.; Muntoni, F.; Talbot, K., et al. Systemic peptide-mediated oligonucleotide therapy improves long-term survival in spinal muscular atrophy. *Proceedings of the National Academy of Sciences of the United States of America* **2016**, *113*, 10962-10967, doi:10.1073/pnas.1605731113.

458. Betts, C.A.; Saleh, A.F.; Carr, C.A.; Hammond, S.M.; Coenen-Stass, A.M.L.; Godfrey, C.; McClorey, G.; Varela, M.A.; Roberts, T.C.; Clarke, K., et al. Prevention of exercised induced cardiomyopathy following Pip-PMO treatment in dystrophic mdx mice. *Scientific Reports* **2015**, *5*, 8986, doi:10.1038/srep08986.

459. Johnson, L.N.; Cashman, S.M.; Kumar-Singh, R. Cell-penetrating Peptide for Enhanced Delivery of Nucleic Acids and Drugs to Ocular Tissues Including Retina and Cornea. *Molecular Therapy* **2008**, *16*, 107-114, doi:<https://doi.org/10.1038/sj.mt.6300324>.

460. Johnson, L.N.; Cashman, S.M.; Read, S.P.; Kumar-Singh, R. Cell penetrating peptide POD mediates delivery of recombinant proteins to retina, cornea and skin. *Vision Research* **2010**, *50*, 686-697, doi:<https://doi.org/10.1016/j.visres.2009.08.028>.

461. Wu, B.; Moulton, H.M.; Iversen, P.L.; Jiang, J.; Li, J.; Li, J.; Spurney, C.F.; Sali, A.; Guerron, A.D.; Nagaraju, K., et al. Effective rescue of dystrophin improves cardiac function in dystrophin-deficient mice by a modified morpholino oligomer. *Proceedings of the National Academy of Sciences* **2008**, 10.1073/pnas.0805676105, doi:10.1073/pnas.0805676105.

462. Wesolowski, D.; Tae, H.S.; Gandotra, N.; Llopis, P.; Shen, N.; Altman, S. Basic peptide-morpholino oligomer conjugate that is very effective in killing bacteria by gene-specific and nonspecific modes. *Proceedings of the National Academy of Sciences* **2011**, *108*, 16582, doi:10.1073/pnas.1112561108.

463. Oess, S.; Hildt, E. Novel cell permeable motif derived from the PreS2-domain of hepatitis-B virus surface antigens. *Gene Therapy* **2000**, *7*, 750-758, doi:10.1038/sj.gt.3301154.

464. Xia, H.; Gu, G.; Hu, Q.; Liu, Z.; Jiang, M.; Kang, T.; Miao, D.; Song, Q.; Yao, L.; Tu, Y., et al. Activatable Cell Penetrating Peptide-Conjugated Nanoparticles with Enhanced Permeability for Site-Specific Targeting Delivery of Anticancer Drug. *Bioconjugate Chemistry* **2013**, *24*, 419-430, doi:10.1021/bc300520t.

465. Duan, Z.; Chen, C.; Qin, J.; Liu, Q.; Wang, Q.; Xu, X.; Wang, J. Cell-penetrating peptide conjugates to enhance the antitumor effect of paclitaxel on drug-resistant lung cancer. *Drug Delivery* **2017**, *24*, 752-764, doi:10.1080/10717544.2017.1321060.

466. Ye, J.; Liu, E.; Gong, J.; Wang, J.; Huang, Y.; He, H.; Yang, V.C. High-Yield Synthesis of Monomeric LMWP(CPP)-siRNA Covalent Conjugate for Effective Cytosolic Delivery of siRNA. *Theranostics* **2017**, *7*, 2495-2508, doi:10.7150/thno.19863.

467. Ye, J.; Pei, X.; Cui, H.; Yu, Z.; Lee, H.; Wang, J.; Wang, X.; Sun, L.; He, H.; Yang, V.C. Cellular uptake mechanism and comparative in vitro cytotoxicity studies of monomeric LMWP-siRNA conjugate. *Journal of Industrial and Engineering Chemistry* **2018**, *63*, 103-111, doi:<https://doi.org/10.1016/j.jiec.2018.02.005>.

468. Yu, Z.; Ye, J.; Pei, X.; Sun, L.; Liu, E.; Wang, J.; Huang, Y.; Lee, S.J.; He, H. Improved method for synthesis of low molecular weight protamine–siRNA conjugate. *Acta Pharmaceutica Sinica B* **2018**, *8*, 116-126, doi:<https://doi.org/10.1016/j.apsb.2017.11.011>.

469. Sun, Y.; Sun, Y.; Zhao, R.; Gao, K. Intracellular delivery of messenger RNA by recombinant PP7 virus-like particles carrying low molecular weight protamine. *BMC Biotechnology* **2016**, *16*, 46, doi:10.1186/s12896-016-0274-9.

470. Ho, A.; Schwarze, S.R.; Mermelstein, S.J.; Waksman, G.; Dowdy, S.F. Synthetic Protein Transduction Domains: Enhanced Transduction Potential <em>in Vitro</em> and <em>in Vivo</em>. *Cancer Research* **2001**, *61*, 474.

471. Michiue, H.; Eguchi, A.; Scadeng, M.; Dowdy, S.F. Induction of in vivo synthetic lethal RNAi responses to treat glioblastoma. *Cancer biology & therapy* **2009**, *8*, 2306-2313, doi:10.4161/cbt.8.23.10271.

472. Izabela, R.; Jarosław, R.; Magdalena, A.; Piotr, R.; Ivan, K. Transportan 10 improves the anticancer activity of cisplatin. *Naunyn-Schmiedeberg's Archives of Pharmacology* **2016**, *389*, 485-497, doi:10.1007/s00210-016-1219-5.

473. Wang, H.; Chen, X.; Chen, Y.; Sun, L.; Li, G.; Zhai, M.; Zhai, W.; Kang, Q.; Gao, Y.; Qi, Y. Antitumor activity of novel chimeric peptides derived from cyclinD/CDK4 and the protein transduction domain 4. *Amino Acids* **2013**, *44*, 499-510, doi:10.1007/s00726-012-1360-5.

474. Lättig-Tünnemann, G.; Prinz, M.; Hoffmann, D.; Behlke, J.; Palm-Apergi, C.; Morano, I.; Herce, H.D.; Cardoso, M.C. Backbone rigidity and static presentation of guanidinium groups increases cellular uptake of arginine-rich cell-penetrating peptides. *Nature Communications* **2011**, *2*, 453, doi:10.1038/ncomms1459.

475. Elmquist, A.; Hansen, M.; Langel, U. Structure-activity relationship study of the cell-penetrating peptide pVEC. *Biochimica et biophysica acta* **2006**, *1758*, 721-729, doi:10.1016/j.bbamem.2006.05.013.

476. Nan, Y.H.; Park, I.-S.; Hahm, K.-S.; Shin, S.Y. Antimicrobial activity, bactericidal mechanism and LPS-neutralizing activity of the cell-penetrating peptide pVEC and its analogs. *Journal of Peptide Science* **2011**, *17*, 812-817, doi:10.1002/psc.1408.

477. Alaybeyoglu, B.; Sariyar Akbulut, B.; Ozkirimli, E. pVEC hydrophobic N-terminus is critical for antibacterial activity. *Journal of Peptide Science* **2018**, *24*, e3083, doi:10.1002/psc.3083.

478. Gurbel Paul, A.; Bliden Kevin, P.; Turner Susan, E.; Tantry Udaya, S.; Gesheff Martin, G.; Barr Travis, P.; Covic, L.; Kuliopulos, A. Cell-Penetrating Pepducin Therapy Targeting PAR1 in Subjects With Coronary Artery Disease. *Arteriosclerosis, Thrombosis, and Vascular Biology* **2016**, *36*, 189-197, doi:10.1161/ATVBAHA.115.306777.

479. Geller, B.L.; Marshall-Batty, K.; Schnell, F.J.; McKnight, M.M.; Iversen, P.L.; Greenberg, D.E. Gene-Silencing Antisense Oligomers Inhibit Acinetobacter Growth In Vitro and In Vivo. *The Journal of Infectious Diseases* **2013**, *208*, 1553-1560, doi:10.1093/infdis/jit460.

480. Tian, Y.; Mi, G.; Chen, Q.; Chaurasiya, B.; Li, Y.; Shi, D.; Zhang, Y.; Webster, T.J.; Sun, C.; Shen, Y. Acid-Induced Activated Cell-Penetrating Peptide-Modified Cholesterol-Conjugated Polyoxyethylene Sorbitol Oleate Mixed Micelles for pH-Triggered Drug Release and Efficient Brain Tumor Targeting Based on a Charge Reversal Mechanism. *ACS Applied Materials & Interfaces* **2018**, *10*, 43411-43428, doi:10.1021/acsami.8b15147.

481. Cao, C.; Sheng, D.; Li, X.; Xue, F.; Liu, L.; Zhong, Y.; Wei, P.; Li, R.; Yi, T. Furin substrate as a novel cell-penetrating peptide: combining a delivery vector and an inducer of cargo release. *Chemical Communications* **2019**, *55*, 11872-11875, doi:10.1039/C9CC02353D.

482. Mussa Farkhani, S.; Asoudeh Fard, A.; Zakeri-Milani, P.; Shahbazi Mojarrad, J.; Valizadeh, H. Enhancing antitumor activity of silver nanoparticles by modification with cell-penetrating peptides. *Artificial Cells, Nanomedicine, and Biotechnology* **2017**, *45*, 1029-1035, doi:10.1080/21691401.2016.1200059.

483. Nasrolahi Shirazi, A.; Tiwari, R.; Chhikara, B.S.; Mandal, D.; Parang, K. Design and Biological Evaluation of Cell-Penetrating Peptide–Doxorubicin Conjugates as Prodrugs. *Molecular Pharmaceutics* **2013**, *10*, 488-499, doi:10.1021/mp3004034.

484. Burrer, R.; Neuman, B.W.; Ting, J.P.C.; Stein, D.A.; Moulton, H.M.; Iversen, P.L.; Kuhn, P.; Buchmeier, M.J. Antiviral Effects of Antisense Morpholino Oligomers in Murine Coronavirus Infection Models. *Journal of Virology* **2007**, *81*, 5637, doi:10.1128/JVI.02360-06.

485. Amantana, A.; Moulton, H.M.; Cate, M.L.; Reddy, M.T.; Whitehead, T.; Hassinger, J.N.; Youngblood, D.S.; Iversen, P.L. Pharmacokinetics, Biodistribution, Stability and Toxicity of a Cell-Penetrating Peptide−Morpholino Oligomer Conjugate. *Bioconjugate Chemistry* **2007**, *18*, 1325-1331, doi:10.1021/bc070060v.

486. Hosseini, A.; Lattanzio, F.A.; Samudre, S.S.; DiSandro, G.; Sheppard, J.D.; Williams, P.B. Efficacy of a Phosphorodiamidate Morpholino Oligomer Antisense Compound in the Inhibition of Corneal Transplant Rejection in a Rat Cornea Transplant Model. *Journal of Ocular Pharmacology and Therapeutics* **2011**, *28*, 194-201, doi:10.1089/jop.2011.0135.

487. Lai, S.-H.; Stein, D.A.; Guerrero-Plata, A.; Liao, S.-L.; Ivanciuc, T.; Hong, C.; Iversen, P.L.; Casola, A.; Garofalo, R.P. Inhibition of respiratory syncytial virus infections with morpholino oligomers in cell cultures and in mice. *Molecular therapy : the journal of the American Society of Gene Therapy* **2008**, *16*, 1120-1128, doi:10.1038/mt.2008.81.

488. Leger, A.J.; Mosquea, L.M.; Clayton, N.P.; Wu, I.H.; Weeden, T.; Nelson, C.A.; Phillips, L.; Roberts, E.; Piepenhagen, P.A.; Cheng, S.H., et al. Systemic Delivery of a Peptide-Linked Morpholino Oligonucleotide Neutralizes Mutant RNA Toxicity in a Mouse Model of Myotonic Dystrophy. *Nucleic Acid Therapeutics* **2013**, *23*, 109-117, doi:10.1089/nat.2012.0404.

489. Yin, H.; Moulton, H.M.; Seow, Y.; Boyd, C.; Boutilier, J.; Iverson, P.; Wood, M.J.A. Cell-penetrating peptide-conjugated antisense oligonucleotides restore systemic muscle and cardiac dystrophin expression and function. *Human Molecular Genetics* **2008**, *17*, 3909-3918, doi:10.1093/hmg/ddn293.

490. Yin, H.; Moulton, H.M.; Betts, C.; Seow, Y.; Boutilier, J.; Iverson, P.L.; Wood, M.J.A. A fusion peptide directs enhanced systemic dystrophin exon skipping and functional restoration in dystrophin-deficient mdx mice. *Human Molecular Genetics* **2009**, *18*, 4405-4414, doi:10.1093/hmg/ddp395.

491. Kinali, M.; Arechavala-Gomeza, V.; Feng, L.; Cirak, S.; Hunt, D.; Adkin, C.; Guglieri, M.; Ashton, E.; Abbs, S.; Nihoyannopoulos, P., et al. Local restoration of dystrophin expression with the morpholino oligomer AVI-4658 in Duchenne muscular dystrophy: a single-blind, placebo-controlled, dose-escalation, proof-of-concept study. *The Lancet Neurology* **2009**, *8*, 918-928, doi:10.1016/S1474-4422(09)70211-X.

492. Cirak, S.; Arechavala-Gomeza, V.; Guglieri, M.; Feng, L.; Torelli, S.; Anthony, K.; Abbs, S.; Garralda, M.E.; Bourke, J.; Wells, D.J., et al. Exon skipping and dystrophin restoration in patients with Duchenne muscular dystrophy after systemic phosphorodiamidate morpholino oligomer treatment: an open-label, phase 2, dose-escalation study. *The Lancet* **2011**, *378*, 595-605, doi:10.1016/S0140-6736(11)60756-3.

493. Jearawiriyapaisarn, N.; Moulton, H.M.; Buckley, B.; Roberts, J.; Sazani, P.; Fucharoen, S.; Iversen, P.L.; Kole, R. Sustained dystrophin expression induced by peptide-conjugated morpholino oligomers in the muscles of mdx mice. *Molecular therapy : the journal of the American Society of Gene Therapy* **2008**, *16*, 1624-1629, doi:10.1038/mt.2008.120.

494. Hitsuda, T.; Michiue, H.; Kitamatsu, M.; Fujimura, A.; Wang, F.; Yamamoto, T.; Han, X.-J.; Tazawa, H.; Uneda, A.; Ohmori, I., et al. A protein transduction method using oligo-arginine (3R) for the delivery of transcription factors into cell nuclei. *Biomaterials* **2012**, *33*, 4665-4672, doi:<https://doi.org/10.1016/j.biomaterials.2012.02.049>.

495. Futaki, S.; Suzuki, T.; Ohashi, W.; Yagami, T.; Tanaka, S.; Ueda, K.; Sugiura, Y. Arginine-rich Peptides: AN ABUNDANT SOURCE OF MEMBRANE-PERMEABLE PEPTIDES HAVING POTENTIAL AS CARRIERS FOR INTRACELLULAR PROTEIN DELIVERY. *Journal of Biological Chemistry* **2001**, *276*, 5836-5840.

496. Wu, Z.; Chen, K.; Yildiz, I.; Dirksen, A.; Fischer, R.; Dawson, P.E.; Steinmetz, N.F. Development of viral nanoparticles for efficient intracellular delivery. *Nanoscale* **2012**, *4*, 3567-3576, doi:10.1039/C2NR30366C.

497. Alhakamy, N.A.; Berkland, C.J. Polyarginine Molecular Weight Determines Transfection Efficiency of Calcium Condensed Complexes. *Molecular Pharmaceutics* **2013**, *10*, 1940-1948, doi:10.1021/mp3007117.

498. Shinde, A.; Feher, K.M.; Hu, C.; Slowinska, K. Peptide internalization enabled by folding: triple helical cell-penetrating peptides. *Journal of Peptide Science* **2015**, *21*, 77-84, doi:10.1002/psc.2725.

499. Morishita, M.; Kamei, N.; Ehara, J.; Isowa, K.; Takayama, K. A novel approach using functional peptides for efficient intestinal absorption of insulin. *Journal of controlled release : official journal of the Controlled Release Society* **2007**, *118*, 177-184, doi:10.1016/j.jconrel.2006.12.022.

500. de Cogan, F.; Hill, L.J.; Lynch, A.; Morgan-Warren, P.J.; Lechner, J.; Berwick, M.R.; Peacock, A.F.A.; Chen, M.; Scott, R.A.H.; Xu, H., et al. Topical Delivery of Anti-VEGF Drugs to the Ocular Posterior Segment Using Cell-Penetrating Peptides. *Investigative Ophthalmology & Visual Science* **2017**, *58*, 2578-2590, doi:10.1167/iovs.16-20072.

501. Zhang, Y.; Li, L.; Han, M.; Hu, J.; Zhang, L. Amphiphilic Lipopeptide-Mediated Transport of Insulin and Cell Membrane Penetration Mechanism. *Molecules* **2015**, *20*, doi:10.3390/molecules201219771.

502. Jiang, T.; Zhang, Z.; Zhang, Y.; Lv, H.; Zhou, J.; Li, C.; Hou, L.; Zhang, Q. Dual-functional liposomes based on pH-responsive cell-penetrating peptide and hyaluronic acid for tumor-targeted anticancer drug delivery. *Biomaterials* **2012**, *33*, 9246-9258, doi:10.1016/j.biomaterials.2012.09.027.

503. Rothbard, J.B.; Garlington, S.; Lin, Q.; Kirschberg, T.; Kreider, E.; McGrane, P.L.; Wender, P.A.; Khavari, P.A. Conjugation of arginine oligomers to cyclosporin A facilitates topical delivery and inhibition of inflammation. *Nature Medicine* **2000**, *6*, 1253-1257, doi:10.1038/81359.

504. Tints, K.; Prink, M.; Neuman, T.; Palm, K. LXXLL peptide converts transportan 10 to a potent inducer of apoptosis in breast cancer cells. *International journal of molecular sciences* **2014**, *15*, 5680-5698, doi:10.3390/ijms15045680.

505. Zhang, L.; Song, L.; Zhang, C.; Ren, Y. Improving intestinal insulin absorption efficiency through coadministration of cell-penetrating peptide and hydroxypropyl-β-cyclodextrin. *Carbohydrate Polymers* **2012**, *87*, 1822-1827, doi:<https://doi.org/10.1016/j.carbpol.2011.10.002>.

506. Opriessnig, T.; Patel, D.; Wang, R.; Halbur, P.G.; Meng, X.-J.; Stein, D.A.; Zhang, Y.-J. Inhibition of porcine reproductive and respiratory syndrome virus infection in piglets by a peptide-conjugated morpholino oligomer. *Antiviral research* **2011**, *91*, 36-42, doi:10.1016/j.antiviral.2011.04.012.

507. Ma, Y.; Gong, C.; Ma, Y.; Fan, F.; Luo, M.; Yang, F.; Zhang, Y.-H. Direct cytosolic delivery of cargoes in vivo by a chimera consisting of D- and L-arginine residues. *Journal of Controlled Release* **2012**, *162*, 286-294, doi:<https://doi.org/10.1016/j.jconrel.2012.07.022>.

508. Liu, X.; Liu, C.; Zhang, W.; Xie, C.; Wei, G.; Lu, W. Oligoarginine-modified biodegradable nanoparticles improve the intestinal absorption of insulin. *International Journal of Pharmaceutics* **2013**, *448*, 159-167, doi:<https://doi.org/10.1016/j.ijpharm.2013.03.033>.

509. Mei, L.; Zhang, Q.; Yang, Y.; He, Q.; Gao, H. Angiopep-2 and activatable cell penetrating peptide dual modified nanoparticles for enhanced tumor targeting and penetrating. *International Journal of Pharmaceutics* **2014**, *474*, 95-102, doi:<https://doi.org/10.1016/j.ijpharm.2014.08.020>.

510. Zhao, J.; Zhang, X.; Sun, X.; Zhao, M.; Yu, C.; Lee, R.J.; Sun, F.; Zhou, Y.; Li, Y.; Teng, L. Dual-functional lipid polymeric hybrid pH-responsive nanoparticles decorated with cell penetrating peptide and folate for therapy against rheumatoid arthritis. *European Journal of Pharmaceutics and Biopharmaceutics* **2018**, *130*, 39-47, doi:<https://doi.org/10.1016/j.ejpb.2018.06.020>.

511. Tanaka, G.; Nakase, I.; Fukuda, Y.; Masuda, R.; Oishi, S.; Shimura, K.; Kawaguchi, Y.; Takatani-Nakase, T.; Langel, Ü.; Gräslund, A., et al. CXCR4 Stimulates Macropinocytosis: Implications for Cellular Uptake of Arginine-Rich Cell-Penetrating Peptides and HIV. *Chemistry & Biology* **2012**, *19*, 1437-1446, doi:<https://doi.org/10.1016/j.chembiol.2012.09.011>.

512. Nomura, W.; Ohashi, N.; Mori, A.; Tamamura, H. An In-Cell Fluorogenic Tag–Probe System for Protein Dynamics Imaging Enabled by Cell-Penetrating Peptides. *Bioconjugate Chemistry* **2015**, *26*, 1080-1085, doi:10.1021/acs.bioconjchem.5b00131.

513. Hu, J.; Lou, Y.; Wu, F. Improved Intracellular Delivery of Polyarginine Peptides with Cargoes. *The Journal of Physical Chemistry B* **2019**, *123*, 2636-2644, doi:10.1021/acs.jpcb.8b10483.

514. Lin, R.; Zhang, P.; Cheetham, A.G.; Walston, J.; Abadir, P.; Cui, H. Dual Peptide Conjugation Strategy for Improved Cellular Uptake and Mitochondria Targeting. *Bioconjugate Chemistry* **2015**, *26*, 71-77, doi:10.1021/bc500408p.

515. El-Sayed, A.; Masuda, T.; Khalil, I.; Akita, H.; Harashima, H. Enhanced gene expression by a novel stearylated INF7 peptide derivative through fusion independent endosomal escape. *Journal of Controlled Release* **2009**, *138*, 160-167, doi:<https://doi.org/10.1016/j.jconrel.2009.05.018>.

516. Eto, Y.; Yoshioka, Y.; Asavatanabodee, R.; Kida, S.; Maeda, M.; Mukai, Y.; Mizuguchi, H.; Kawasaki, K.; Okada, N.; Nakagawa, S. Transduction of adenovirus vectors modified with cell-penetrating peptides. *Peptides* **2009**, *30*, 1548-1552, doi:10.1016/j.peptides.2009.05.017.

517. Deshpande, P.; Jhaveri, A.; Pattni, B.; Biswas, S.; Torchilin, V. Transferrin and octaarginine modified dual-functional liposomes with improved cancer cell targeting and enhanced intracellular delivery for the treatment of ovarian cancer. *Drug Delivery* **2018**, *25*, 517-532, doi:10.1080/10717544.2018.1435747.

518. Rompicharla, S.V.K.; Kumari, P.; Ghosh, B.; Biswas, S. Octa-arginine modified poly(amidoamine) dendrimers for improved delivery and cytotoxic effect of paclitaxel in cancer. *Artificial Cells, Nanomedicine, and Biotechnology* **2018**, *46*, 847-859, doi:10.1080/21691401.2018.1470527.

519. Qiu, Y.; Yu, Q.; Liu, Y.; Tang, J.; Wang, X.; Lu, Z.; Xu, Z.; He, Q. Dual Receptor Targeting Cell Penetrating Peptide Modified Liposome for Glioma and Breast Cancer Postoperative Recurrence Therapy. *Pharmaceutical Research* **2018**, *35*, 130, doi:10.1007/s11095-018-2399-0.

520. Hakata, Y.; Ishikawa, S.; Ohtsuki, T.; Miyazawa, M.; Kitamatsu, M. Intracellular delivery of a peptide nucleic acid-based hybrid of an autophagy inducing peptide with a cell-penetrating peptide. *Organic & Biomolecular Chemistry* **2020**, *18*, 1978-1986, doi:10.1039/C9OB02559F.

521. Niu, Z.; Samaridou, E.; Jaumain, E.; Coëne, J.; Ullio, G.; Shrestha, N.; Garcia, J.; Durán-Lobato, M.; Tovar, S.; Santander-Ortega, M.J., et al. PEG-PGA enveloped octaarginine-peptide nanocomplexes: An oral peptide delivery strategy. *Journal of Controlled Release* **2018**, *276*, 125-139, doi:<https://doi.org/10.1016/j.jconrel.2018.03.004>.

522. Nakase, I.; Katayama, M.; Hattori, Y.; Ishimura, M.; Inaura, S.; Fujiwara, D.; Takatani-Nakase, T.; Fujii, I.; Futaki, S.; Kirihata, M. Intracellular target delivery of cell-penetrating peptide-conjugated dodecaborate for boron neutron capture therapy (BNCT). *Chemical Communications* **2019**, *55*, 13955-13958, doi:10.1039/C9CC03924D.

523. Sakuma, S.; Suita, M.; Masaoka, Y.; Kataoka, M.; Nakajima, N.; Shinkai, N.; Yamauchi, H.; Hiwatari, K.-i.; Tachikawa, H.; Kimura, R., et al. Oligoarginine-linked polymers as a new class of penetration enhancers. *Journal of controlled release : official journal of the Controlled Release Society* **2010**, *148*, 187-196, doi:10.1016/j.jconrel.2010.08.022.

524. Kamei, N.; Morishita, M.; Takayama, K. Importance of intermolecular interaction on the improvement of intestinal therapeutic peptide/protein absorption using cell-penetrating peptides. *Journal of controlled release : official journal of the Controlled Release Society* **2009**, *136*, 179-186, doi:10.1016/j.jconrel.2009.02.015.

525. Rothbard, J.B.; Jessop, T.C.; Lewis, R.S.; Murray, B.A.; Wender, P.A. Role of Membrane Potential and Hydrogen Bonding in the Mechanism of Translocation of Guanidinium-Rich Peptides into Cells. *Journal of the American Chemical Society* **2004**, *126*, 9506-9507, doi:10.1021/ja0482536.

526. Jiang, T.; Wang, T.; Li, T.; Ma, Y.; Shen, S.; He, B.; Mo, R. Enhanced Transdermal Drug Delivery by Transfersome-Embedded Oligopeptide Hydrogel for Topical Chemotherapy of Melanoma. *ACS Nano* **2018**, *12*, 9693-9701, doi:10.1021/acsnano.8b03800.

527. Wender, P.A.; Mitchell, D.J.; Pattabiraman, K.; Pelkey, E.T.; Steinman, L.; Rothbard, J.B. The design, synthesis, and evaluation of molecules that enable or enhance cellular uptake: Peptoid molecular transporters. *Proceedings of the National Academy of Sciences* **2000**, *97*, 13003, doi:10.1073/pnas.97.24.13003.

528. Cantini, L.; Attaway, C.C.; Butler, B.; Andino, L.M.; Sokolosky, M.L.; Jakymiw, A. Fusogenic-Oligoarginine Peptide-Mediated Delivery of siRNAs Targeting the CIP2A Oncogene into Oral Cancer Cells. *PLOS ONE* **2013**, *8*, e73348, doi:10.1371/journal.pone.0073348.

529. Tünnemann, G.; Ter-Avetisyan, G.; Martin, R.M.; Stöckl, M.; Herrmann, A.; Cardoso, M.C. Live-cell analysis of cell penetration ability and toxicity of oligo-arginines. *Journal of Peptide Science* **2008**, *14*, 469-476, doi:10.1002/psc.968.

530. Liu, Q.; Lin, Z.; Du, J.; Lin, H.; Wang, J. Delivery of miRNA-29b Using R9-LK15, a Novel Cell-Penetrating Peptide, Promotes Osteogenic Differentiation of Bone Mesenchymal Stem Cells. *BioMed Research International* **2019**, *2019*, doi:10.1155/2019/3032158.

531. Walrant, A.; Correia, I.; Jiao, C.-Y.; Lequin, O.; Bent, E.H.; Goasdoué, N.; Lacombe, C.; Chassaing, G.; Sagan, S.; Alves, I.D. Different membrane behaviour and cellular uptake of three basic arginine-rich peptides. *Biochimica et biophysica acta* **2011**, *1808*, 382-393, doi:10.1016/j.bbamem.2010.09.009.

532. Khalil, I.A.; Kimura, S.; Sato, Y.; Harashima, H. Synergism between a cell penetrating peptide and a pH-sensitive cationic lipid in efficient gene delivery based on double-coated nanoparticles. *Journal of controlled release : official journal of the Controlled Release Society* **2018**, *275*, 107-116, doi:10.1016/j.jconrel.2018.02.016.

533. Gao, H.; Zhang, Q.; Yang, Y.; Jiang, X.; He, Q. Tumor homing cell penetrating peptide decorated nanoparticles used for enhancing tumor targeting delivery and therapy. *International journal of pharmaceutics* **2015**, *478*, 240-250, doi:10.1016/j.ijpharm.2014.11.029.

534. Alhakamy, N.A.; Alaofi, A.L.; Ahmed, O.A.A.; Fahmy, U.A.; Md, S.; Abdulaal, W.H.; Alfaleh, M.A.; Chakraborty, A.; Berkland, C.J.; Dhar, P. Development of lipid membrane based assays to accurately predict the transfection efficiency of cell-penetrating peptide-based gene nanoparticles. *International Journal of Pharmaceutics* **2020**, *580*, 119221, doi:<https://doi.org/10.1016/j.ijpharm.2020.119221>.

535. Tsai, C.-W.; Lin, Z.-W.; Chang, W.-F.; Chen, Y.-F.; Hu, W.-W. Development of an indolicidin-derived peptide by reducing membrane perturbation to decrease cytotoxicity and maintain gene delivery ability. *Colloids and Surfaces B: Biointerfaces* **2018**, *165*, 18-27, doi:<https://doi.org/10.1016/j.colsurfb.2018.02.007>.

536. Uhl, P.; Grundmann, C.; Sauter, M.; Storck, P.; Tursch, A.; Özbek, S.; Leotta, K.; Roth, R.; Witzigmann, D.; Kulkarni, J.A., et al. Coating of PLA-nanoparticles with cyclic, arginine-rich cell penetrating peptides enables oral delivery of liraglutide. *Nanomedicine: Nanotechnology, Biology and Medicine* **2020**, *24*, 102132, doi:<https://doi.org/10.1016/j.nano.2019.102132>.

537. Kim, D.; Lee, I.-H.; Kim, S.; Choi, M.; Kim, H.; Ahn, S.; Saw, P.E.; Jeon, H.; Lee, Y.; Jon, S. A Specific STAT3-Binding Peptide Exerts Antiproliferative Effects and Antitumor Activity by Inhibiting STAT3 Phosphorylation and Signaling. *Cancer Research* **2014**, 10.1158/0008-5472.CAN-13-2187, doi:10.1158/0008-5472.CAN-13-2187.

538. Guo, F.; Fu, Q.; Zhou, K.; Jin, C.; Wu, W.; Ji, X.; Yan, Q.; Yang, Q.; Wu, D.; Li, A., et al. Matrix metalloprotein-triggered, cell penetrating peptide-modified star-shaped nanoparticles for tumor targeting and cancer therapy. *Journal of Nanobiotechnology* **2020**, *18*, 48, doi:10.1186/s12951-020-00595-5.

539. Liu, A.-d.; Xu, H.; Gao, Y.-n.; Luo, D.-n.; Li, Z.-f.; Voss, C.; Li, S.S.C.; Cao, X. (Arg)9-SH2 superbinder: a novel promising anticancer therapy to melanoma by blocking phosphotyrosine signaling. *Journal of Experimental & Clinical Cancer Research* **2018**, *37*, 138, doi:10.1186/s13046-018-0812-5.

540. Chen, S.; Cui, J.; Jiang, T.; Olson, E.S.; Cai, Q.-Y.; Yang, M.; Wu, W.; Guthrie, J.M.; Robertson, J.D.; Lipton, S.A., et al. Gelatinase activity imaged by activatable cell-penetrating peptides in cell-based and in vivo models of stroke. *Journal of Cerebral Blood Flow & Metabolism* **2015**, *37*, 188-200, doi:10.1177/0271678X15621573.

541. Traboulsi, H.; Larkin, H.; Bonin, M.-A.; Volkov, L.; Lavoie, C.L.; Marsault, É. Macrocyclic Cell Penetrating Peptides: A Study of Structure-Penetration Properties. *Bioconjugate Chemistry* **2015**, *26*, 405-411, doi:10.1021/acs.bioconjchem.5b00023.

542. Mansur, A.A.P.; Carvalho, S.M.; Lobato, Z.I.P.; Leite, M.d.F.; Cunha, A.d.S.; Mansur, H.S. Design and Development of Polysaccharide-Doxorubicin-Peptide Bioconjugates for Dual Synergistic Effects of Integrin-Targeted and Cell-Penetrating Peptides for Cancer Chemotherapy. *Bioconjugate Chemistry* **2018**, *29*, 1973-2000, doi:10.1021/acs.bioconjchem.8b00208.

543. Dougherty, P.G.; Wen, J.; Pan, X.; Koley, A.; Ren, J.-G.; Sahni, A.; Basu, R.; Salim, H.; Appiah Kubi, G.; Qian, Z., et al. Enhancing the Cell Permeability of Stapled Peptides with a Cyclic Cell-Penetrating Peptide. *Journal of Medicinal Chemistry* **2019**, *62*, 10098-10107, doi:10.1021/acs.jmedchem.9b00456.

544. Melikov, K.; Hara, A.; Yamoah, K.; Zaitseva, E.; Zaitsev, E.; Chernomordik, Leonid V. Efficient entry of cell-penetrating peptide nona-arginine into adherent cells involves a transient increase in intracellular calcium. *Biochemical Journal* **2015**, *471*, 221-230, doi:10.1042/BJ20150272.

545. Bode, S.A.; Wallbrecher, R.; Brock, R.; van Hest, J.C.M.; Löwik, D.W.P.M. Activation of cell-penetrating peptides by disulfide bridge formation of truncated precursors. *Chemical Communications* **2014**, *50*, 415-417, doi:10.1039/C3CC46826G.

546. Marks, J.R.; Placone, J.; Hristova, K.; Wimley, W.C. Spontaneous Membrane-Translocating Peptides by Orthogonal High-Throughput Screening. *Journal of the American Chemical Society* **2011**, *133*, 8995-9004, doi:10.1021/ja2017416.

547. Simion, V.; Stan, D.; Constantinescu, C.A.; Deleanu, M.; Dragan, E.; Tucureanu, M.M.; Gan, A.-M.; Butoi, E.; Constantin, A.; Manduteanu, I., et al. Conjugation of curcumin-loaded lipid nanoemulsions with cell-penetrating peptides increases their cellular uptake and enhances the anti-inflammatory effects in endothelial cells. *Journal of Pharmacy and Pharmacology* **2016**, *68*, 195-207, doi:10.1111/jphp.12513.

548. Liu, Y.; Ibricevic, A.; Cohen, J.A.; Cohen, J.L.; Gunsten, S.P.; Fréchet, J.M.J.; Walter, M.J.; Welch, M.J.; Brody, S.L. Impact of Hydrogel Nanoparticle Size and Functionalization on In Vivo Behavior for Lung Imaging and Therapeutics. *Molecular Pharmaceutics* **2009**, *6*, 1891-1902, doi:10.1021/mp900215p.

549. Alexander-Bryant, A.A.; Zhang, H.; Attaway, C.C.; Pugh, W.; Eggart, L.; Sansevere, R.M.; Andino, L.M.; Dinh, L.; Cantini, L.P.; Jakymiw, A. Dual peptide-mediated targeted delivery of bioactive siRNAs to oral cancer cells in vivo. *Oral oncology* **2017**, *72*, 123-131, doi:10.1016/j.oraloncology.2017.07.004.

550. Jin, C.; Bai, L.; Lin, L.; Wang, S.; Yin, X. Paclitaxel-loaded nanoparticles decorated with bivalent fragment HAb18 F(ab’)2 and cell penetrating peptide for improved therapeutic effect on hepatocellular carcinoma. *Artificial Cells, Nanomedicine, and Biotechnology* **2018**, *46*, 1076-1084, doi:10.1080/21691401.2017.1360325.

551. Kim, W.J.; Christensen, L.V.; Jo, S.; Yockman, J.W.; Jeong, J.H.; Kim, Y.-H.; Kim, S.W. Cholesteryl Oligoarginine Delivering Vascular Endothelial Growth Factor siRNA Effectively Inhibits Tumor Growth in Colon Adenocarcinoma. *Molecular Therapy* **2006**, *14*, 343-350, doi:10.1016/j.ymthe.2006.03.022.

552. Ullah, I.; Chung, K.; Beloor, J.; Kim, J.; Cho, M.; Kim, N.; Lee, K.Y.; Kumar, P.; Lee, S.-K. Trileucine residues in a ligand-CPP-based siRNA delivery platform improve endosomal escape of siRNA. *Journal of Drug Targeting* **2017**, *25*, 320-329, doi:10.1080/1061186X.2016.1258566.

553. Breger, J.C.; Muttenthaler, M.; Delehanty, J.B.; Thompson, D.A.; Oh, E.; Susumu, K.; Deschamps, J.R.; Anderson, G.P.; Field, L.D.; Walper, S.A., et al. Nanoparticle cellular uptake by dendritic wedge peptides: achieving single peptide facilitated delivery. *Nanoscale* **2017**, *9*, 10447-10464, doi:10.1039/C7NR03362A.

554. Mathieu, E.; Bernard, A.-S.; Ching, H.Y.V.; Somogyi, A.; Medjoubi, K.; Fores, J.R.; Bertrand, H.C.; Vincent, A.; Trépout, S.; Guerquin-Kern, J.-L., et al. Anti-inflammatory activity of superoxide dismutase mimics functionalized with cell-penetrating peptides. *Dalton Transactions* **2020**, *49*, 2323-2330, doi:10.1039/C9DT04619D.

555. Yang, Y.; Xia, M.; Zhang, S.; Zhang, X. Cell-penetrating peptide-modified quantum dots as a ratiometric nanobiosensor for the simultaneous sensing and imaging of lysosomes and extracellular pH. *Chemical Communications* **2020**, *56*, 145-148, doi:10.1039/C9CC07596H.

556. Lee, H.; Lim, S.I.; Shin, S.-H.; Lim, Y.; Koh, J.W.; Yang, S. Conjugation of Cell-Penetrating Peptides to Antimicrobial Peptides Enhances Antibacterial Activity. *ACS Omega* **2019**, *4*, 15694-15701, doi:10.1021/acsomega.9b02278.

557. Candan, G.; Michiue, H.; Ishikawa, S.; Fujimura, A.; Hayashi, K.; Uneda, A.; Mori, A.; Ohmori, I.; Nishiki, T.-i.; Matsui, H., et al. Combining poly-arginine with the hydrophobic counter-anion 4-(1-pyrenyl)-butyric acid for protein transduction in transdermal delivery. *Biomaterials* **2012**, *33*, 6468-6475, doi:<https://doi.org/10.1016/j.biomaterials.2012.04.056>.

558. Michiue, H.; Sakurai, Y.; Kondo, N.; Kitamatsu, M.; Bin, F.; Nakajima, K.; Hirota, Y.; Kawabata, S.; Nishiki, T.-i.; Ohmori, I., et al. The acceleration of boron neutron capture therapy using multi-linked mercaptoundecahydrododecaborate (BSH) fused cell-penetrating peptide. *Biomaterials* **2014**, *35*, 3396-3405, doi:<https://doi.org/10.1016/j.biomaterials.2013.12.055>.

559. Ding, C.; Wu, K.; Wang, W.; Guan, Z.; Wang, L.; Wang, X.; Wang, R.; Liu, L.; Fan, J. Synthesis of a cell penetrating peptide modified superparamagnetic iron oxide and MRI detection of bladder cancer. *Oncotarget* **2017**, *8*, 4718-4729, doi:10.18632/oncotarget.13578.

560. Ichimizu, S.; Watanabe, H.; Maeda, H.; Hamasaki, K.; Nakamura, Y.; Chuang, V.T.G.; Kinoshita, R.; Nishida, K.; Tanaka, R.; Enoki, Y., et al. Design and tuning of a cell-penetrating albumin derivative as a versatile nanovehicle for intracellular drug delivery. *Journal of Controlled Release* **2018**, *277*, 23-34, doi:<https://doi.org/10.1016/j.jconrel.2018.02.037>.

561. Zhang, H.-T.; Yu, M.; Niu, Y.-J.; Liu, W.-Z.; Pang, W.-H.; Ding, J.; Wang, J.-C. Polyarginine-Mediated siRNA Delivery: A Mechanistic Study of Intracellular Trafficking of PCL-R15/siRNA Nanoplexes. *Molecular Pharmaceutics* **2020**, 10.1021/acs.molpharmaceut.0c00120, doi:10.1021/acs.molpharmaceut.0c00120.

562. Noguchi, K.; Hirano, M.; Hashimoto, T.; Yuba, E.; Takatani-Nakase, T.; Nakase, I. Effects of Lyophilization of Arginine-rich Cell-penetrating Peptide-modified Extracellular Vesicles on Intracellular Delivery. *Anticancer Research* **2019**, *39*, 6701-6709.

563. Yoon, H.Y.; Kwak, S.S.; Jang, M.H.; Kang, M.H.; Sung, S.W.; Kim, C.H.; Kim, S.R.; Yeom, D.W.; Kang, M.J.; Choi, Y.W. Docetaxel-loaded RIPL peptide (IPLVVPLRRRRRRRRC)-conjugated liposomes: Drug release, cytotoxicity, and antitumor efficacy. *International Journal of Pharmaceutics* **2017**, *523*, 229-237, doi:<https://doi.org/10.1016/j.ijpharm.2017.03.045>.

564. Chinak, A.O.; Shernyukov, V.A.; Ovcherenko, S.S.; Sviridov, A.E.; Golyshev, M.V.; Fomin, S.A.; Pyshnaya, A.I.; Kuligina, V.E.; Richter, A.V.; Bagryanskaya, G.E. Structural and Aggregation Features of a Human κ-Casein Fragment with Antitumor and Cell-Penetrating Properties. *Molecules* **2019**, *24*, doi:10.3390/molecules24162919.

565. Rydberg, H.A.; Matson, M.; Åmand, H.L.; Esbjörner, E.K.; Nordén, B. Effects of Tryptophan Content and Backbone Spacing on the Uptake Efficiency of Cell-Penetrating Peptides. *Biochemistry* **2012**, *51*, 5531-5539, doi:10.1021/bi300454k.

566. Sudo, K.; Niikura, K.; Iwaki, K.; Kohyama, S.; Fujiwara, K.; Doi, N. Human-derived fusogenic peptides for the intracellular delivery of proteins. *Journal of controlled release : official journal of the Controlled Release Society* **2017**, *255*, 1-11, doi:10.1016/j.jconrel.2017.03.398.

567. Mano, M.; Henriques, A.; Paiva, A.; Prieto, M.; Gavilanes, F.; Simões, S.; Pedroso de Lima, M.C. Cellular uptake of S413-PV peptide occurs upon conformational changes induced by peptide-membrane interactions. *Biochimica et biophysica acta* **2006**, *1758*, 336-346, doi:10.1016/j.bbamem.2006.01.014.

568. Pujals, S.; Fernández-Carneado, J.; Kogan, M.J.; Martinez, J.; Cavelier, F.; Giralt, E. Replacement of a Proline with Silaproline Causes a 20-Fold Increase in the Cellular Uptake of a Pro-Rich Peptide. *Journal of the American Chemical Society* **2006**, *128*, 8479-8483, doi:10.1021/ja060036c.

569. Pujals, S.; Sabidó, E.; Tarragó, T.; Giralt, E. all-D proline-rich cell-penetrating peptides: a preliminary in vivo internalization study. *Biochemical Society Transactions* **2007**, *35*, 794-796, doi:10.1042/BST0350794.

570. Martín, I.; Teixidó, M.; Giralt , E. Design, Synthesis and Characterization of a New Anionic Cell-Penetrating Peptide: SAP(E). *ChemBioChem* **2011**, *12*, 896-903, doi:10.1002/cbic.201000679.

571. Franz, J.; Lelle, M.; Peneva, K.; Bonn, M.; Weidner, T. SAP(E) – A cell-penetrating polyproline helix at lipid interfaces. *Biochimica et Biophysica Acta (BBA) - Biomembranes* **2016**, *1858*, 2028-2034, doi:<https://doi.org/10.1016/j.bbamem.2016.05.021>.

572. Li, L.; Yang, L.; Li, M.; Zhang, L. A cell-penetrating peptide mediated chitosan nanocarriers for improving intestinal insulin delivery. *Carbohydrate polymers* **2017**, *174*, 182-189, doi:10.1016/j.carbpol.2017.06.061.

573. Splith, K.; Neundorf, I.; Hu, W.; Peindy N'Dongo, H.W.; Vasylyeva, V.; Merz, K.; Schatzschneider, U. Influence of the metal complex-to-peptide linker on the synthesis and properties of bioactive CpMn(CO)3 peptide conjugates. *Dalton transactions (Cambridge, England : 2003)* **2010**, *39*, 2536-2545, doi:10.1039/b916907e.

574. Horn, M.; Reichart, F.; Natividad-Tietz, S.; Diaz, D.; Neundorf, I. Tuning the properties of a novel short cell-penetrating peptide by intramolecular cyclization with a triazole bridge. *Chemical Communications* **2016**, *52*, 2261-2264, doi:10.1039/C5CC08938G.

575. Lin, Y.-Z.; Yao, S.; Veach, R.A.; Torgerson, T.R.; Hawiger, J. Inhibition of Nuclear Translocation of Transcription Factor NF-κB by a Synthetic Peptide Containing a Cell Membrane-permeable Motif and Nuclear Localization Sequence. *Journal of Biological Chemistry* **1995**, *270*, 14255-14258.

576. Hsu, T.; Mitragotri, S. Delivery of siRNA and other macromolecules into skin and cells using a peptide enhancer. *Proceedings of the National Academy of Sciences* **2011**, *108*, 15816, doi:10.1073/pnas.1016152108.

577. Chen, M.; Kumar, S.; Anselmo, A.C.; Gupta, V.; Slee, D.H.; Muraski, J.A.; Mitragotri, S. Topical delivery of Cyclosporine A into the skin using SPACE-peptide. *Journal of controlled release : official journal of the Controlled Release Society* **2015**, *199*, 190-197, doi:10.1016/j.jconrel.2014.11.015.

578. Song, J.; Huang, S.; Zhang, Z.; Jia, B.; Xie, H.; Kai, M.; Zhang, W. SPA: a peptide antagonist that acts as a cell-penetrating peptide for drug delivery. *Drug Delivery* **2020**, *27*, 91-99, doi:10.1080/10717544.2019.1706669.

579. Hyun, S.; Choi, Y.; Lee, H.N.; Lee, C.; Oh, D.; Lee, D.-K.; Lee, C.; Lee, Y.; Yu, J. Construction of histidine-containing hydrocarbon stapled cell penetrating peptides for in vitro and in vivo delivery of siRNAs. *Chemical Science* **2018**, *9*, 3820-3827, doi:10.1039/C8SC00074C.

580. Kapur, A.; Medina, S.H.; Wang, W.; Palui, G.; Schneider, J.P.; Mattoussi, H. Intracellular Delivery of Gold Nanocolloids Promoted by a Chemically Conjugated Anticancer Peptide. *ACS Omega* **2018**, *3*, 12754-12762, doi:10.1021/acsomega.8b02276.

581. Rousselle, C.; Smirnova, M.; Clair, P.; Lefauconnier, J.-M.; Chavanieu, A.; Calas, B.; Scherrmann, J.-M.; Temsamani, J. Enhanced Delivery of Doxorubicin into the Brain via a Peptide-Vector-Mediated Strategy: Saturation Kinetics and Specificity. *Journal of Pharmacology and Experimental Therapeutics* **2001**, *296*, 124.

582. Frankel, A.D.; Pabo, C.O. Cellular uptake of the tat protein from human immunodeficiency virus. *Cell* **1988**, *55*, 1189-1193, doi:<https://doi.org/10.1016/0092-8674(88)90263-2>.

583. Liang, J.F.; Yang, V.C. Insulin-cell penetrating peptide hybrids with improved intestinal absorption efficiency. *Biochemical and Biophysical Research Communications* **2005**, *335*, 734-738, doi:<https://doi.org/10.1016/j.bbrc.2005.07.142>.

584. Kim, H.; Kitamatsu, M.; Ohtsuki, T. Enhanced intracellular peptide delivery by multivalent cell-penetrating peptide with bioreducible linkage. *Bioorganic & Medicinal Chemistry Letters* **2018**, *28*, 378-381, doi:<https://doi.org/10.1016/j.bmcl.2017.12.035>.

585. Zhu, Y.; Jiang, Y.; Meng, F.; Deng, C.; Cheng, R.; Zhang, J.; Feijen, J.; Zhong, Z. Highly efficacious and specific anti-glioma chemotherapy by tandem nanomicelles co-functionalized with brain tumor-targeting and cell-penetrating peptides. *Journal of Controlled Release* **2018**, *278*, 1-8, doi:<https://doi.org/10.1016/j.jconrel.2018.03.025>.

586. Hossain, M.K.; Cho, H.-Y.; Kim, K.-J.; Choi, J.-W. In situ monitoring of doxorubicin release from biohybrid nanoparticles modified with antibody and cell-penetrating peptides in breast cancer cells using surface-enhanced Raman spectroscopy. *Biosensors and Bioelectronics* **2015**, *71*, 300-305, doi:<https://doi.org/10.1016/j.bios.2015.04.053>.

587. Qian, Y.; Wang, Y.; Jia, F.; Wang, Z.; Yue, C.; Zhang, W.; Hu, Z.; Wang, W. Tumor-microenvironment controlled nanomicelles with AIE property for boosting cancer therapy and apoptosis monitoring. *Biomaterials* **2019**, *188*, 96-106, doi:<https://doi.org/10.1016/j.biomaterials.2018.10.003>.

588. Kaplan, I.M.; Wadia, J.S.; Dowdy, S.F. Cationic TAT peptide transduction domain enters cells by macropinocytosis. *Journal of controlled release : official journal of the Controlled Release Society* **2005**, *102*, 247-253, doi:10.1016/j.jconrel.2004.10.018.

589. Zhang, X.; Li, Y.; Cheng, Y.; Tan, H.; Li, Z.; Qu, Y.; Mu, G.; Wang, F. Tat PTD–endostatin: A novel anti-angiogenesis protein with ocular barrier permeability via eye-drops. *Biochimica et Biophysica Acta (BBA) - General Subjects* **2015**, *1850*, 1140-1149, doi:<https://doi.org/10.1016/j.bbagen.2015.01.019>.

590. Wu, H.; You, C.; Chen, F.; Jiao, J.; Gao, Z.; An, P.; Sun, B.; Chen, R. Enhanced cellular uptake of near-infrared triggered targeted nanoparticles by cell-penetrating peptide TAT for combined chemo/photothermal/photodynamic therapy. *Materials science & engineering. C, Materials for biological applications* **2019**, *103*, 109738, doi:10.1016/j.msec.2019.109738.

591. Ferrari, A.; Pellegrini, V.; Arcangeli, C.; Fittipaldi, A.; Giacca, M.; Beltram, F. Caveolae-Mediated internalization of extracellular HIV-1 tat fusion proteins visualized in real time. *Molecular Therapy* **2003**, *8*, 284-294, doi:<https://doi.org/10.1016/S1525-0016(03)00122-9>.

592. Green, M.; Loewenstein, P.M. Autonomous functional domains of chemically synthesized human immunodeficiency virus tat <em>trans</em>-activator protein. *Cell* **1988**, *55*, 1179-1188, doi:10.1016/0092-8674(88)90262-0.

593. Fawell, S.; Seery, J.; Daikh, Y.; Moore, C.; Chen, L.L.; Pepinsky, B.; Barsoum, J. Tat-mediated delivery of heterologous proteins into cells. *Proceedings of the National Academy of Sciences of the United States of America* **1994**, *91*, 664-668, doi:10.1073/pnas.91.2.664.

594. Orzechowska, E.J.; Kozlowska, E.; Czubaty, A.; Kozlowski, P.; Staron, K.; Trzcinska-Danielewicz, J. Controlled delivery of BID protein fused with TAT peptide sensitizes cancer cells to apoptosis. *BMC cancer* **2014**, *14*, 771-771, doi:10.1186/1471-2407-14-771.

595. Han, T.; Tang, Y.; Ugai, H.; Perry, L.E.; Siegal, G.P.; Contreras, J.L.; Wu, H. Genetic incorporation of the protein transduction domain of Tat into Ad5 fiber enhances gene transfer efficacy. *Virology Journal* **2007**, *4*, 103, doi:10.1186/1743-422X-4-103.

596. Defaus, S.; Gallo, M.; Abengózar, M.A.; Rivas, L.; Andreu, D. A Synthetic Strategy for Conjugation of Paromomycin to Cell-Penetrating Tat(48-60) for Delivery and Visualization into Leishmania Parasites. *International Journal of Peptides* **2017**, *2017*, doi:10.1155/2017/4213037.

597. Zheng, X.-S.; Zong, C.; Wang, X.; Ren, B. Cell-Penetrating Peptide Conjugated SERS Nanosensor for in Situ Intracellular pH Imaging of Single Living Cells during Cell Cycle. *Analytical Chemistry* **2019**, *91*, 8383-8389, doi:10.1021/acs.analchem.9b01191.

598. Patel, K.D.; De Zoysa, G.H.; Kanamala, M.; Patel, K.; Pilkington, L.I.; Barker, D.; Reynisson, J.; Wu, Z.; Sarojini, V. Novel Cell-Penetrating Peptide Conjugated Proteasome Inhibitors: Anticancer and Antifungal Investigations. *Journal of Medicinal Chemistry* **2020**, *63*, 334-348, doi:10.1021/acs.jmedchem.9b01694.

599. Morshed, R.A.; Muroski, M.E.; Dai, Q.; Wegscheid, M.L.; Auffinger, B.; Yu, D.; Han, Y.; Zhang, L.; Wu, M.; Cheng, Y., et al. Cell-Penetrating Peptide-Modified Gold Nanoparticles for the Delivery of Doxorubicin to Brain Metastatic Breast Cancer. *Molecular Pharmaceutics* **2016**, *13*, 1843-1854, doi:10.1021/acs.molpharmaceut.6b00004.

600. Li, J.; Liu, F.; Shao, Q.; Min, Y.; Costa, M.; Yeow, E.K.L.; Xing, B. Enzyme-Responsive Cell-Penetrating Peptide Conjugated Mesoporous Silica Quantum Dot Nanocarriers for Controlled Release of Nucleus-Targeted Drug Molecules and Real-Time Intracellular Fluorescence Imaging of Tumor Cells. *Advanced Healthcare Materials* **2014**, *3*, 1230-1239, doi:10.1002/adhm.201300613.

601. Säälik, P.; Padari, K.; Niinep, A.; Lorents, A.; Hansen, M.; Jokitalo, E.; Langel, Ü.; Pooga, M. Protein Delivery with Transportans Is Mediated by Caveolae Rather Than Flotillin-Dependent Pathways. *Bioconjugate Chemistry* **2009**, *20*, 877-887, doi:10.1021/bc800416f.

602. Liu, S.; Mao, Q.; Zhang, W.; Zheng, X.; Bian, Y.; Wang, D.; Li, H.; Chai, L.; Zhao, J.; Xia, H. Genetically modified adenoviral vector with the protein transduction domain of Tat improves gene transfer to CAR-deficient cells. *Bioscience Reports* **2009**, *29*, 103-109, doi:10.1042/BSR20080023.

603. Malhotra, M.; Tomaro-Duchesneau, C.; Saha, S.; Kahouli, I.; Prakash, S. Development and characterization of chitosan-PEG-TAT nanoparticles for the intracellular delivery of siRNA. *International journal of nanomedicine* **2013**, *8*, 2041-2052, doi:10.2147/IJN.S43683.

604. Martorana, F.; Brambilla, L.; Valori, C.F.; Bergamaschi, C.; Roncoroni, C.; Aronica, E.; Volterra, A.; Bezzi, P.; Rossi, D. The BH4 domain of Bcl-XL rescues astrocyte degeneration in amyotrophic lateral sclerosis by modulating intracellular calcium signals. *Human Molecular Genetics* **2011**, *21*, 826-840, doi:10.1093/hmg/ddr513.

605. Collard, R.; Majtan, T.; Park, I.; Kraus, J.P. Import of TAT-conjugated propionyl-CoA carboxylase using models of propionic acidemia. *Molecular and Cellular Biology* **2018**, 10.1128/MCB.00491-17, MCB.00491-00417, doi:10.1128/MCB.00491-17.

606. El Zaoui, I.; Touchard, E.; Berdugo, M.; Abadie, C.; Kowalczuk, L.; Deloche, C.; Zhao, M.; Naud, M.-C.; Combette, J.-M.; Behar-Cohen, F. Subconjunctival injection of XG-102, a c-Jun N-terminal kinase inhibitor peptide, in the treatment of endotoxin-induced uveitis in rats. *Journal of ocular pharmacology and therapeutics : the official journal of the Association for Ocular Pharmacology and Therapeutics* **2015**, *31*, 17-24, doi:10.1089/jop.2014.0019.

607. Vivès, E.; Brodin, P.; Lebleu, B. A Truncated HIV-1 Tat Protein Basic Domain Rapidly Translocates through the Plasma Membrane and Accumulates in the Cell Nucleus. *Journal of Biological Chemistry* **1997**, *272*, 16010-16017.

608. Vasconcelos, A.; Vega, E.; Pérez, Y.; Gómara, M.J.; García, M.L.; Haro, I. Conjugation of cell-penetrating peptides with poly(lactic-co-glycolic acid)-polyethylene glycol nanoparticles improves ocular drug delivery. *International journal of nanomedicine* **2015**, *10*, 609-631, doi:10.2147/IJN.S71198.

609. Shuai, Q.; Cai, Y.; Zhao, G.; Sun, X. Cell-Penetrating Peptide Modified PEG-PLA Micelles for Efficient PTX Delivery. *International Journal of Molecular Sciences* **2020**, *21*, doi:10.3390/ijms21051856.

610. Kamide, K.; Nakakubo, H.; Uno, S.; Fukamizu, A. Isolation of novel cell-penetrating peptides from a random peptide library using in vitro virus and their modifications. *International Journal of Molecular Medicine* **2010**, *25*, 41 - 51, doi:10.3892/ijmm_00000311.

611. Zheng, C.; Ma, C.; Bai, E.; Yang, K.; Xu, R. Transferrin and cell-penetrating peptide dual-functioned liposome for targeted drug delivery to glioma. *International journal of clinical and experimental medicine* **2015**, *8*, 1658-1668.

612. Jose, G.G.; Larsen, I.V.; Gauger, J.; Carballo, E.; Stern, R.; Brummel, R.; Brandt, C.R. A cationic peptide, TAT-Cd°, inhibits herpes simplex virus type 1 ocular infection in vivo. *Investigative ophthalmology & visual science* **2013**, *54*, 1070-1079, doi:10.1167/iovs.12-10250.

613. Mueller, N.H.; Ammar, D.A.; Petrash, J.M. Cell penetration peptides for enhanced entry of αB-crystallin into lens cells. *Investigative ophthalmology & visual science* **2013**, *54*, 2-8, doi:10.1167/iovs.12-10947.

614. Tegtbur, U.; Busse, M.W.; Jung, K.; Pethig, K.; Haverich, A. Time course of physical reconditioning during exercise rehabilitation late after heart transplantation. *The Journal of Heart and Lung Transplantation* **2005**, *24*, 270-274, doi:10.1016/j.healun.2003.12.010.

615. Green, M.; Ishino, M.; Loewenstein, P.M. Mutational analysis of HIV-1 Tat minimal domain peptides: Identification of <em>trans</em>-dominant mutants that suppress HIV-LTR-driven gene expression. *Cell* **1989**, *58*, 215-223, doi:10.1016/0092-8674(89)90417-0.

616. Eguchi, A.; Akuta, T.; Okuyama, H.; Senda, T.; Yokoi, H.; Inokuchi, H.; Fujita, S.; Hayakawa, T.; Takeda, K.; Hasegawa, M., et al. Protein Transduction Domain of HIV-1 Tat Protein Promotes Efficient Delivery of DNA into Mammalian Cells. *Journal of Biological Chemistry* **2001**, *276*, 26204-26210.

617. Fittipaldi, A.; Ferrari, A.; Zoppé, M.; Arcangeli, C.; Pellegrini, V.; Beltram, F.; Giacca, M. Cell Membrane Lipid Rafts Mediate Caveolar Endocytosis of HIV-1 Tat Fusion Proteins. *Journal of Biological Chemistry* **2003**, *278*, 34141-34149.

618. Richard, J.P.; Melikov, K.; Vives, E.; Ramos, C.; Verbeure, B.; Gait, M.J.; Chernomordik, L.V.; Lebleu, B. Cell-penetrating Peptides: A REEVALUATION OF THE MECHANISM OF CELLULAR UPTAKE. *Journal of Biological Chemistry* **2003**, *278*, 585-590.

619. Gondeau, C.; Gerbal-Chaloin, S.; Bello, P.; Aldrian-Herrada, G.; Morris, M.C.; Divita, G. Design of a Novel Class of Peptide Inhibitors of Cyclin-dependent Kinase/Cyclin Activation. *Journal of Biological Chemistry* **2005**, *280*, 13793-13800, doi:10.1074/jbc.M413690200.

620. Richard, J.P.; Melikov, K.; Brooks, H.; Prevot, P.; Lebleu, B.; Chernomordik, L.V. Cellular Uptake of Unconjugated TAT Peptide Involves Clathrin-dependent Endocytosis and Heparan Sulfate Receptors. *Journal of Biological Chemistry* **2005**, *280*, 15300-15306.

621. Pan, Y.; Zhang, Y.; Jia, T.; Zhang, K.; Li, J.; Wang, L. Development of a microRNA delivery system based on bacteriophage MS2 virus-like particles. *The FEBS Journal* **2012**, *279*, 1198-1208, doi:10.1111/j.1742-4658.2012.08512.x.

622. Doeppner, T.R.; Nagel, F.; Dietz, G.P.H.; Weise, J.; Tönges, L.; Schwarting, S.; Bähr, M. TAT-Hsp70-Mediated Neuroprotection and Increased Survival of Neuronal Precursor Cells after Focal Cerebral Ischemia in Mice. *Journal of Cerebral Blood Flow & Metabolism* **2009**, *29*, 1187-1196, doi:10.1038/jcbfm.2009.44.

623. Wang, Y.; Lin, H.; Lin, S.; Qu, J.; Xiao, J.; Huang, Y.; Xiao, Y.; Fu, X.; Yang, Y.; Li, X. Cell-penetrating peptide TAT-mediated delivery of acidic FGF to retina and protection against ischemia–reperfusion injury in rats. *Journal of Cellular and Molecular Medicine* **2010**, *14*, 1998-2005, doi:10.1111/j.1582-4934.2009.00786.x.

624. Ur Rahman, A.; Khan, S.; Khan, M. Transport of trans-activator of transcription (TAT) peptide in tumour tissue model: evaluation of factors affecting the transport of TAT evidenced by flow cytometry. *Journal of Pharmacy and Pharmacology* **2020**, *72*, 519-530, doi:10.1111/jphp.13221.

625. Kim, S.M.; Chae, M.K.; Lee, C.; Yim, M.S.; Bang, J.K.; Ryu, E.K. Enhanced cellular uptake of a TAT-conjugated peptide inhibitor targeting the polo-box domain of polo-like kinase 1. *Amino Acids* **2014**, *46*, 2595-2603, doi:10.1007/s00726-014-1798-8.

626. Huang, H.-L.; Lin, J.W. Dual Peptide-Modified Nanoparticles Improve Combination Chemotherapy of Etoposide and siPIK3CA Against Drug-Resistant Small Cell Lung Carcinoma. *Pharmaceutics* **2020**, *12*, doi:10.3390/pharmaceutics12030254.

627. Ma, N.; Liu, P.; He, N.; Gu, N.; Wu, F.-G.; Chen, Z. Action of Gold Nanospikes-Based Nanoradiosensitizers: Cellular Internalization, Radiotherapy, and Autophagy. *ACS Applied Materials & Interfaces* **2017**, *9*, 31526-31542, doi:10.1021/acsami.7b09599.

628. Kanazawa, T.; Morisaki, K.; Suzuki, S.; Takashima, Y. Prolongation of Life in Rats with Malignant Glioma by Intranasal siRNA/Drug Codelivery to the Brain with Cell-Penetrating Peptide-Modified Micelles. *Molecular Pharmaceutics* **2014**, *11*, 1471-1478, doi:10.1021/mp400644e.

629. Hingorani, D.V.; Chapelin, F.; Stares, E.; Adams, S.R.; Okada, H.; Ahrens, E.T. Cell penetrating peptide functionalized perfluorocarbon nanoemulsions for targeted cell labeling and enhanced fluorine-19 MRI detection. *Magnetic Resonance in Medicine* **2020**, *83*, 974-987, doi:10.1002/mrm.27988.

630. Inomata, K.; Ohno, A.; Tochio, H.; Isogai, S.; Tenno, T.; Nakase, I.; Takeuchi, T.; Futaki, S.; Ito, Y.; Hiroaki, H., et al. High-resolution multi-dimensional NMR spectroscopy of proteins in human cells. *Nature* **2009**, *458*, 106-109, doi:10.1038/nature07839.

631. Shin, M.C.; Zhang, J.; Ah Min, K.; Lee, K.; Moon, C.; Balthasar, J.P.; Yang, V.C. Combination of antibody targeting and PTD-mediated intracellular toxin delivery for colorectal cancer therapy. *Journal of controlled release : official journal of the Controlled Release Society* **2014**, *194*, 197-210, doi:10.1016/j.jconrel.2014.08.030.

632. Niu, J.; Chu, Y.; Huang, Y.-F.; Chong, Y.-S.; Jiang, Z.-H.; Mao, Z.-W.; Peng, L.-H.; Gao, J.-Q. Transdermal Gene Delivery by Functional Peptide-Conjugated Cationic Gold Nanoparticle Reverses the Progression and Metastasis of Cutaneous Melanoma. *ACS Applied Materials & Interfaces* **2017**, *9*, 9388-9401, doi:10.1021/acsami.6b16378.

633. Peng, J.; Rao, Y.; Yang, X.; Jia, J.; Wu, Y.; Lu, J.; Tao, Y.; Tu, W. Targeting neuronal nitric oxide synthase by a cell penetrating peptide Tat-LK15/siRNA bioconjugate. *Neuroscience Letters* **2017**, *650*, 153-160, doi:<https://doi.org/10.1016/j.neulet.2017.04.045>.

634. Wang, G.; Jia, T.; Xu, X.; Chang, L.; Zhang, R.; Fu, Y.; Li, Y.; Yang, X.; Zhang, K.; Lin, G., et al. Novel miR-122 delivery system based on MS2 virus like particle surface displaying cell-penetrating peptide TAT for hepatocellular carcinoma. *Oncotarget; Vol 7, No 37* **2016**.

635. Chiu, Y.-L.; Ali, A.; Chu, C.-y.; Cao, H.; Rana, T.M. Visualizing a Correlation between siRNA Localization, Cellular Uptake, and RNAi in Living Cells. *Chemistry & Biology* **2004**, *11*, 1165-1175, doi:<https://doi.org/10.1016/j.chembiol.2004.06.006>.

636. Cao, X.-W.; Yang, X.-Z.; Du, X.; Fu, L.-Y.; Zhang, T.-Z.; Shan, H.-W.; Zhao, J.; Wang, F.-J. Structure optimisation to improve the delivery efficiency and cell selectivity of a tumour-targeting cell-penetrating peptide. *Journal of Drug Targeting* **2018**, *26*, 777-792, doi:10.1080/1061186X.2018.1424858.

637. Yong, X.; Yang, X.; Emory, S.R.; Wang, J.; Dai, J.; Yu, X.; Mei, L.; Xie, J.; Ruan, G. A potent, minimally invasive and simple strategy of enhancing intracellular targeted delivery of Tat peptide-conjugated quantum dots: organic solvent-based permeation enhancer. *Biomaterials Science* **2018**, *6*, 3085-3095, doi:10.1039/C8BM00928G.

638. Meng, Z.; Kang, Z.; Sun, C.; Yang, S.; Zhao, B.; Feng, S.; Meng, Q.; Liu, K. Enhanced gene transfection efficiency by use of peptide vectors containing laminin receptor-targeting sequence YIGSR. *Nanoscale* **2018**, *10*, 1215-1227, doi:10.1039/C7NR05843H.

639. Yu, D.; Jin, C.; Leja, J.; Majdalani, N.; Nilsson, B.; Eriksson, F.; Essand, M. Adenovirus with Hexon Tat-Protein Transduction Domain Modification Exhibits Increased Therapeutic Effect in Experimental Neuroblastoma and Neuroendocrine Tumors. *Journal of Virology* **2011**, *85*, 13114, doi:10.1128/JVI.05759-11.

640. Zhang, L.; Liu, F.; Li, G.; Zhou, Y.; Yang, Y. Twin-Arginine Translocation Peptide Conjugated Epirubicin-Loaded Nanoparticles for Enhanced Tumor Penetrating and Targeting. *Journal of Pharmaceutical Sciences* **2015**, *104*, 4185-4196, doi:10.1002/jps.24649.

641. Touchard, E.; Omri, S.; Naud, M.-C.; Berdugo, M.; Deloche, C.; Abadie, C.; Jonet, L.; Jeanny, J.-C.; Crisanti, P.; de Kozak, Y., et al. A Peptide Inhibitor of c-Jun N-Terminal Kinase for the Treatment of Endotoxin-Induced Uveitis. *Investigative Ophthalmology & Visual Science* **2010**, *51*, 4683-4693, doi:10.1167/iovs.09-4733.

642. Yoshioka, Y.; Asavatanabodee, R.; Eto, Y.; Watanabe, H.; Morishige, T.; Yao, X.; Kida, S.; Maeda, M.; Mukai, Y.; Mizuguchi, H., et al. Tat conjugation of adenovirus vector broadens tropism and enhances transduction efficiency. *Life Sciences* **2008**, *83*, 747-755, doi:<https://doi.org/10.1016/j.lfs.2008.09.022>.

643. Yan, C.; Gu, J.; Hou, D.; Jing, H.; Wang, J.; Guo, Y.; Katsumi, H.; Sakane, T.; Yamamoto, A. Improved tumor targetability of Tat-conjugated PAMAM dendrimers as a novel nanosized anti-tumor drug carrier. *Drug development and industrial pharmacy* **2015**, *41*, 617-622, doi:10.3109/03639045.2014.891127.

644. Wei, B.; Wei, Y.; Zhang, K.; Wang, J.; Xu, R.; Zhan, S.; Lin, G.; Wang, W.; Liu, M.; Wang, L., et al. Development of an antisense RNA delivery system using conjugates of the MS2 bacteriophage capsids and HIV-1 TAT cell penetrating peptide. *Biomedicine & Pharmacotherapy* **2009**, *63*, 313-318, doi:<https://doi.org/10.1016/j.biopha.2008.07.086>.

645. Pang, H.-H.; Chen, P.-Y.; Wei, K.-C.; Huang, C.-W.; Shiue, Y.-L.; Huang, C.-Y.; Yang, H.-W. Convection-Enhanced Delivery of a Virus-Like Nanotherapeutic Agent with Dual-Modal Imaging for Besiegement and Eradication of Brain Tumors. In *Theranostics*, 2019; Vol. 9, pp 1752-1763.

646. Sun, Y.; Sun, Y.; Zhao, R. Establishment of MicroRNA delivery system by PP7 bacteriophage-like particles carrying cell-penetrating peptide. *Journal of bioscience and bioengineering* **2017**, *124*, 242-249, doi:10.1016/j.jbiosc.2017.03.012.

647. Li, X.; Zheng, L.; Xia, Q.; Liu, L.; Mao, M.; Zhou, H.; Zhao, Y.; Shi, J. A novel cell-penetrating peptide protects against neuron apoptosis after cerebral ischemia by inhibiting the nuclear translocation of annexin A1. *Cell Death & Differentiation* **2019**, *26*, 260-275, doi:10.1038/s41418-018-0116-5.

648. Deloche, C.; Lopez-Lazaro, L.; Mouz, S.; Perino, J.; Abadie, C.; Combette, J.-M. XG-102 administered to healthy male volunteers as a single intravenous infusion: a randomized, double-blind, placebo-controlled, dose-escalating study. *Pharmacology Research & Perspectives* **2014**, *2*, e00020, doi:10.1002/prp2.20.

649. Zhu, L.; Wang, T.; Perche, F.; Taigind, A.; Torchilin, V.P. Enhanced anticancer activity of nanopreparation containing an MMP2-sensitive PEG-drug conjugate and cell-penetrating moiety. *Proceedings of the National Academy of Sciences* **2013**, *110*, 17047, doi:10.1073/pnas.1304987110.

650. Snyder, E.L.; Meade, B.R.; Saenz, C.C.; Dowdy, S.F. Treatment of Terminal Peritoneal Carcinomatosis by a Transducible p53-Activating Peptide. *PLOS Biology* **2004**, *2*, e36, doi:10.1371/journal.pbio.0020036.

651. Wadia, J.S.; Stan, R.V.; Dowdy, S.F. Transducible TAT-HA fusogenic peptide enhances escape of TAT-fusion proteins after lipid raft macropinocytosis. *Nature medicine* **2004**, *10*, 310-315, doi:10.1038/nm996.

652. Baoum, A.; Ovcharenko, D.; Berkland, C. Calcium condensed cell penetrating peptide complexes offer highly efficient, low toxicity gene silencing. *International journal of pharmaceutics* **2012**, *427*, 134-142, doi:10.1016/j.ijpharm.2011.08.012.

653. Fan, Y.-X.; Liang, Z.-X.; Liu, Q.-Z.; Xiao, H.; Li, K.-B.; Wu, J.-Z. Cell penetrating peptide of sodium-iodide symporter effect on the I-131 radiotherapy on thyroid cancer. *Experimental and therapeutic medicine* **2017**, *13*, 989-994, doi:10.3892/etm.2017.4079.

654. Zhang, Q.; Tang, J.; Fu, L.; Ran, R.; Liu, Y.; Yuan, M.; He, Q. A pH-responsive α-helical cell penetrating peptide-mediated liposomal delivery system. *Biomaterials* **2013**, *34*, 7980-7993, doi:<https://doi.org/10.1016/j.biomaterials.2013.07.014>.

655. Jana, A.; Narula, P.; Chugh, A.; Kulshreshtha, R. Efficient delivery of anti-miR-210 using Tachyplesin, a cell penetrating peptide, for glioblastoma treatment. *International journal of pharmaceutics* **2019**, *572*, 118789, doi:10.1016/j.ijpharm.2019.118789.

656. Liang, D.-S.; Su, H.-T.; Liu, Y.-J.; Wang, A.-T.; Qi, X.-R. Tumor-specific penetrating peptides-functionalized hyaluronic acid-d-α-tocopheryl succinate based nanoparticles for multi-task delivery to invasive cancers. *Biomaterials* **2015**, *71*, 11-23, doi:<https://doi.org/10.1016/j.biomaterials.2015.08.035>.

657. Dong, P.; Cai, H.; Chen, L.; Li, Y.; Yuan, C.; Wu, X.; Shen, G.; Zhou, H.; Zhang, W.; Li, L. Biodistribution and evaluation of 131I-labeled neuropilin-binding peptide for targeted tumor imaging. *Contrast Media & Molecular Imaging* **2016**, *11*, 467-474, doi:10.1002/cmmi.1708.

658. Macchi, S.; Signore, G.; Boccardi, C.; Di Rienzo, C.; Beltram, F.; Cardarelli, F. Spontaneous membrane-translocating peptides: influence of peptide self-aggregation and cargo polarity. *Scientific Reports* **2015**, *5*, 16914, doi:10.1038/srep16914.

659. Soomets, U.; Lindgren, M.; Gallet, X.; Hällbrink, M.; Elmquist, A.; Balaspiri, L.; Zorko, M.; Pooga, M.; Brasseur, R.; Langel, Ü. Deletion analogues of transportan. *Biochimica et Biophysica Acta (BBA) - Biomembranes* **2000**, *1467*, 165-176, doi:<https://doi.org/10.1016/S0005-2736(00)00216-9>.

660. Pooga, M.; Hällbrink, M.; Zorko, M.; Langel, U.; lo. Cell penetration by transportan. *The FASEB Journal* **1998**, *12*, 67-77, doi:10.1096/fasebj.12.1.67.

661. Ruczyński, J.; Rusiecka, I.; Turecka, K.; Kozłowska, A.; Alenowicz, M.; Gągało, I.; Kawiak, A.; Rekowski, P.; Waleron, K.; Kocić, I. Transportan 10 improves the pharmacokinetics and pharmacodynamics of vancomycin. *Scientific reports* **2019**, *9*, 3247-3247, doi:10.1038/s41598-019-40103-w.

662. Piotr, M.W.A.U.M.K.-W.A.U.J.R.A.U.K.S.-K.A.U.L.K.A.U.A.R.A. Protein and siRNA delivery by transportan and transportan 10 into colorectal cancer cell lines. *Protein and siRNA delivery by transportan and transportan 10 into colorectal cancer cell lines* **2014**, *52*, 270-280-270-280, doi:10.5603/FHC.a2014.0035.

663. Groysman, N.; Orynbayeva, Z.; Katz, M.; Kolusheva, S.; Khanin, M.; Danilenko, M.; Jelinek, R. Membrane processes and biophysical characterization of living cells decorated with chromatic polydiacetylene vesicles. *Biochimica et Biophysica Acta (BBA) - Biomembranes* **2008**, *1778*, 1335-1343, doi:<https://doi.org/10.1016/j.bbamem.2008.01.028>.

664. Stoilova, T.B.; Kovalchuk, S.I.; Egorova, N.S.; Surovoy, A.Y.; Ivanov, V.T. Gramicidin A-based peptide vector for intracellular protein delivery. *Biochimica et Biophysica Acta (BBA) - Biomembranes* **2008**, *1778*, 2026-2031, doi:<https://doi.org/10.1016/j.bbamem.2008.01.029>.

665. Zhang, C.; Ren, W.; Liu, Q.; Tan, Z.; Li, J.; Tong, C. Transportan-derived cell-penetrating peptide delivers siRNA to inhibit replication of influenza virus in vivo. In *Drug Design, Development and Therapy*, 2019; Vol. 13, pp 1059-1068.

666. Arukuusk, P.; Pärnaste, L.; Oskolkov, N.; Copolovici, D.-M.; Margus, H.; Padari, K.; Möll, K.; Maslovskaja, J.; Tegova, R.; Kivi, G., et al. New generation of efficient peptide-based vectors, NickFects, for the delivery of nucleic acids. *Biochimica et biophysica acta* **2013**, *1828*, 1365-1373, doi:10.1016/j.bbamem.2013.01.011.

667. El Andaloussi, S.; Lehto, T.; Mäger, I.; Rosenthal-Aizman, K.; Oprea, I.I.; Simonson, O.E.; Sork, H.; Ezzat, K.; Copolovici, D.M.; Kurrikoff, K., et al. Design of a peptide-based vector, PepFect6, for efficient delivery of siRNA in cell culture and systemically in vivo. *Nucleic Acids Research* **2011**, *39*, 3972-3987, doi:10.1093/nar/gkq1299.

668. Anko, M.; Majhenc, J.; Kogej, K.; Sillard, R.; Langel, U.; Anderluh, G.; Zorko, M. Influence of stearyl and trifluoromethylquinoline modifications of the cell penetrating peptide TP10 on its interaction with a lipid membrane. *Biochimica et biophysica acta* **2012**, *1818*, 915-924, doi:10.1016/j.bbamem.2011.12.028.

669. Jones, S.; Farquhar, M.; Martin, A.; Howl, J. Intracellular translocation of the decapeptide carboxyl terminal of Gi3α induces the dual phosphorylation of p42/p44 MAP kinases. *Biochimica et Biophysica Acta (BBA) - Molecular Cell Research* **2005**, *1745*, 207-214, doi:<https://doi.org/10.1016/j.bbamcr.2005.05.006>.

670. Moghal, M.M.R.; Islam, M.Z.; Hossain, F.; Saha, S.K.; Yamazaki, M. Role of Membrane Potential on Entry of Cell-Penetrating Peptide Transportan 10 into Single Vesicles. *Biophysical Journal* **2020**, *118*, 57-69, doi:<https://doi.org/10.1016/j.bpj.2019.11.012>.

671. Dias, S.A.; Freire, J.M.; Pérez-Peinado, C.; Domingues, M.M.; Gaspar, D.; Vale, N.; Gomes, P.; Andreu, D.; Henriques, S.T.; Castanho, M.A.R.B., et al. New Potent Membrane-Targeting Antibacterial Peptides from Viral Capsid Proteins. *Front Microbiol* **2017**, *8*, 775, doi:10.3389/fmicb.2017.00775.

672. Meyer-Losic, F.; Nicolazzi, C.; Quinonero, J.; Ribes, F.; Michel, M.; Dubois, V.; de Coupade, C.; Boukaissi, M.; Chéné, A.-S.; Tranchant, I., et al. DTS-108, A Novel Peptidic Prodrug of SN38: <em>In vivo</em> Efficacy and Toxicokinetic Studies. *Clinical Cancer Research* **2008**, *14*, 2145, doi:10.1158/1078-0432.CCR-07-4580.

673. Tiwari, P.M.; Eroglu, E.; Bawage, S.S.; Vig, K.; Miller, M.E.; Pillai, S.; Dennis, V.A.; Singh, S.R. Enhanced intracellular translocation and biodistribution of gold nanoparticles functionalized with a cell-penetrating peptide (VG-21) from vesicular stomatitis virus. *Biomaterials* **2014**, *35*, 9484-9494, doi:<https://doi.org/10.1016/j.biomaterials.2014.07.032>.

674. Cashman, S.M.; Sadowski, S.L.; Morris, D.J.; Frederick, J.; Kumar-Singh, R. Intercellular trafficking of adenovirus-delivered HSV VP22 from the retinal pigment epithelium to the photoreceptors--implications for gene therapy. *Molecular therapy : the journal of the American Society of Gene Therapy* **2002**, *6*, 813-823, doi:10.1006/mthe.2002.0806.

675. Feldman, M.; Levy, D. Peptide inhibition of the SETD6 methyltransferase catalytic activity. *Oncotarget* **2017**, *9*, 4875-4885, doi:10.18632/oncotarget.23591.

676. Boenicke, L.; Chu, K.; Pauls, R.; Tams, C.; Kruse, M.-L.; Kurdow, R.; Schniewind, B.; Böhle, A.; Kremer, B.; Kalthoff, H. Efficient dose-dependent and time-dependent protein transduction of pancreatic carcinoma cells in vitro and in vivo using purified VP22-EGFP fusion protein. *Journal of Molecular Medicine* **2003**, *81*, 205-213, doi:10.1007/s00109-003-0421-3.

677. Elliott, G.; O'Hare, P. Intercellular Trafficking and Protein Delivery by a Herpesvirus Structural Protein. *Cell* **1997**, *88*, 223-233, doi:10.1016/S0092-8674(00)81843-7.

678. Oehlke, J.; Krause, E.; Wiesner, B.; Beyermann, M.; Bienert, M. Extensive cellular uptake into endothelial cells of an amphipathic β-sheet forming peptide. *FEBS Letters* **1997**, *415*, 196-199, doi:10.1016/S0014-5793(97)01123-X.

679. Mandal, D.; Nasrolahi Shirazi, A.; Parang, K. Cell-Penetrating Homochiral Cyclic Peptides as Nuclear-Targeting Molecular Transporters. *Angewandte Chemie International Edition* **2011**, *50*, 9633-9637, doi:10.1002/anie.201102572.

680. Wohlfart, S.; Khalansky, A.S.; Gelperina, S.; Begley, D.; Kreuter, J. Kinetics of transport of doxorubicin bound to nanoparticles across the blood–brain barrier. *Journal of Controlled Release* **2011**, *154*, 103-107, doi:<https://doi.org/10.1016/j.jconrel.2011.05.010>.

681. Park, K. Injectable hyaluronic acid hydrogel for bone augmentation. *Journal of Controlled Release* **2011**, *152*, 207, doi:<https://doi.org/10.1016/j.jconrel.2011.05.009>.

682. Lindgren, M.; Rosenthal-Aizman, K.; Saar, K.; Eiríksdóttir, E.; Jiang, Y.; Sassian, M.; Ostlund, P.; Hällbrink, M.; Langel, U. Overcoming methotrexate resistance in breast cancer tumour cells by the use of a new cell-penetrating peptide. *Biochemical pharmacology* **2006**, *71*, 416-425, doi:10.1016/j.bcp.2005.10.048.

683. Shi, Y.; Hu, Y.; Ochbaum, G.; Lin, R.; Bitton, R.; Cui, H.; Azevedo, H.S. Enzymatic activation of cell-penetrating peptides in self-assembled nanostructures triggers fibre-to-micelle morphological transition. *Chemical Communications* **2017**, *53*, 7037-7040, doi:10.1039/C7CC03512H.
